# Lack of shared neoantigens in prevalent mutations in cancer

**DOI:** 10.1186/s12967-024-05110-0

**Published:** 2024-04-10

**Authors:** Concetta Ragone, Beatrice Cavalluzzo, Angela Mauriello, Maria Tagliamonte, Luigi Buonaguro

**Affiliations:** Lab of Innovative Immunological Models Unit, Istituto Nazionale Tumori, IRCCS - “Fondazione Pascale”, Via Mariano Semmola, 52, 80131 Naples, Italy

**Keywords:** Mutations, Neoantigens, Tumor-associated antigens, Tumor-specific antigens, Cancer vaccines, Molecular mimicry, T cell immunity

## Abstract

Tumors are mostly characterized by genetic instability, as result of mutations in surveillance mechanisms, such as DNA damage checkpoint, DNA repair machinery and mitotic checkpoint. Defect in one or more of these mechanisms causes additive accumulation of mutations. Some of these mutations are drivers of transformation and are positively selected during the evolution of the cancer, giving a growth advantage on the cancer cells. If such mutations would result in mutated neoantigens, these could be actionable targets for cancer vaccines and/or adoptive cell therapies. However, the results of the present analysis show, for the first time, that the most prevalent mutations identified in human cancers do not express mutated neoantigens. The hypothesis is that this is the result of the selection operated by the immune system in the very early stages of tumor development. At that stage, the tumor cells characterized by mutations giving rise to highly antigenic non-self-mutated neoantigens would be efficiently targeted and eliminated. Consequently, the outgrowing tumor cells cannot be controlled by the immune system, with an ultimate growth advantage to form large tumors embedded in an immunosuppressive tumor microenvironment (TME). The outcome of such a negative selection operated by the immune system is that the development of off-the-shelf vaccines, based on shared mutated neoantigens, does not seem to be at hand. This finding represents the first demonstration of the key role of the immune system on shaping the tumor antigen presentation and the implication in the development of antitumor immunological strategies.

**Supplementary Information:**

The online version contains supplementary material available at 10.1186/s12967-024-05110-0.

## Introduction

Somatic mutations occur in the genomes of all normal and neoplastic dividing cells. They are the result of errors occurring during DNA replication as well as exposure to exogenous or endogenous mutagens. However, if most of these mutations are repaired by cellular mechanisms, a minority remains fixed in the cell genome. Most of such fixed mutations are biologically neutral and already present in the progenitor cell, before the transformation into the final clonal cancer cell (“passenger” mutations). The remaining ones are “driver” mutations that confer growth advantage on the cell, increasing survival or proliferation, and are selected. The accumulation of the driver mutations over the lifetime of an individual will induce cell transformation and cancer development [1–3]. The number of mutations required to drive a cancer significantly varies across tumor types [4]. Studies have shown that carcinogenesis may be driven by a small number of driver mutations. In particular, one driver mutation per patient is sufficient in sarcomas, thyroid, and testicular cancers; and about four driver mutations per patient are needed in bladder, endometrial, and colorectal cancers [1, 2, 5]. The different mutations in cancer cells show different rates. In particular, most cancers carry 1000 to 20,000 somatic point mutations and a few to hundreds of insertions, deletions, and rearrangements [1].

Such mutations in the genomic sequences of cancer cells may generate modified protein sequences, which may give rise to new epitopes unique to cancer cells. These mutated epitopes (“neoantigens”) are tumor-specific non-self-antigens efficiently recognized by the immune system. Therefore, therapeutic vaccines based on such neoantigens would elicit a T cell immune response that can exclusively target the tumor while sparing healthy tissue [6]. The presence and biological relevance of the T cell immunity against neoantigens in cancer patients is demonstrated by the higher clinical efficacy of Immune checkpoint inhibitors (ICI) in tumors with high tumor mutational burden (TMB) [7–9] and with neoantigen-specific CD8 + T cells [10].

However, mutations and neoantigens are strictly individual (private) and their identification requires a combination of high throughput omics bioinformatics pipeline for each cancer patient, whose reliability has not been fully proven yet. Indeed, a comprehensive meta-analysis of the literature showed that only < 2.7% of prioritized predicted neoantigens are recognized by patient-derived T cells [11]. This has been further confirmed by the tumor neoantigens selection alliance (TESLA) global consortium [12]. Neoantigens were predicted with different pipelines by each participating member from the same tumor sequencing data but only approximately 6% of such predicted neoantigens were recognized by the T cells.

In addition to the complexity and reliability of the approaches, which appear highly difficult to be applied on a large scale, this strictly personalized strategy may fail due to the high mutational rate of tumors, which drives a constant generation of new target mutated neoantigens in the same patient. This would require subsequent rounds of neoantigens identification and vaccine production. More than 100 active or completed clinical trials are listed in clinicaltrials.gov when searching for the terms ‘vaccine’ and ‘neoantigens’, but a clear clinical benefit has not been demonstrated [13]. Only recently, an early phase trial in pancreatic cancer has generated a clinical benefit in terms of prolonged recurrence free survival (RFS) [14].

In this framework, it would be of the highest priority to identify mutated neoantigens, derived from the most frequent mutations and shared among cancer patients, to develop off-the-shelf cancer vaccines.

The results of the present study show that, indeed, such shared mutated neoantigens are not predicted for the most frequent cancer mutations (substitutions and insertion/deletion) in association to the most frequent HLA alleles. This would strongly suggest that only cancer cells lacking immunogenic tumor-specific non-self neoantigens, “poorly-visible” to the immune system, have a growth advantage and proliferate to generate clinically visible tumors. Therefore, off-the-shelf cancer vaccines based on shared mutated neoantigens have low chance to be a feasible strategy.

## Materials and methods

### Selection of cancer mutations from TCGA

The first 100 mutations reported at the TCGA database were selected for the study. Collectively, they represent 55.8% of all mutations identified in human cancers.

### Prediction of mutated neoantigens

Each of the wild-type (wt) proteins were downloaded from the UniProt database (https://www.uniprot.org). The amino acid sequences were manually modified, introducing the described mutation (substitution or insertion/deletion). The paired wt and mutated sequences from each protein were analyzed using the NetMHCpan 4.1 algorithm (https://services.healthtech.dtu.dk/service.php?NetMHCpan-4.1) to predict the best nonamers with affinity values 0—400 nM to the 12 most frequent HLA-A and B alleles. Only those with an affinity value < 100 nM (strong binders – SB) were then selected for subsequent analyses.

### Homology search for neoantigens in literature

The mutated neoantigens, identified as SB according to the NetMHCpan 4.1 prediction tool, were submitted to the Immune Epitope Database & Tools (www.iedb.org) to verify whether the predicted epitopes have been already described and validated in literature. The analysis was performed setting the parameters to search for epitopes with exact match in any host.

### Statistical analysis

The statistical significance of the observed predicted neoantigens derived from either missense or InDel mutations, was calculated based on the observed predicted neoantigens in all samples at TCGA. The normal distribution was calculated as $Z=(X- \mu )/\sigma$, where X is the experimental result; μ is the mean value; σ is the standard deviation. P value was calculated as left-tailed. The confidence interval was calculated as $\mu \pm Z\frac{\sigma }{\surd n}$, where μ is the mean value; Z is the Z-score; σ is the standard deviation; n is the sample population.

## Results

### Most frequent mutations in cancers

The total number of somatic mutations reported in the TCGA database is 190,632. They have been identified in 14,254 cancer cases. The most frequent 100 mutations occur in 8074 cases, which represent 56.65% of all cases, and the top frequent mutation is the BRAF_V640E/V600E_, found in 619/14,254 cases (4.34%) (Additional file 1: Table S1).

**Fig. 1:**
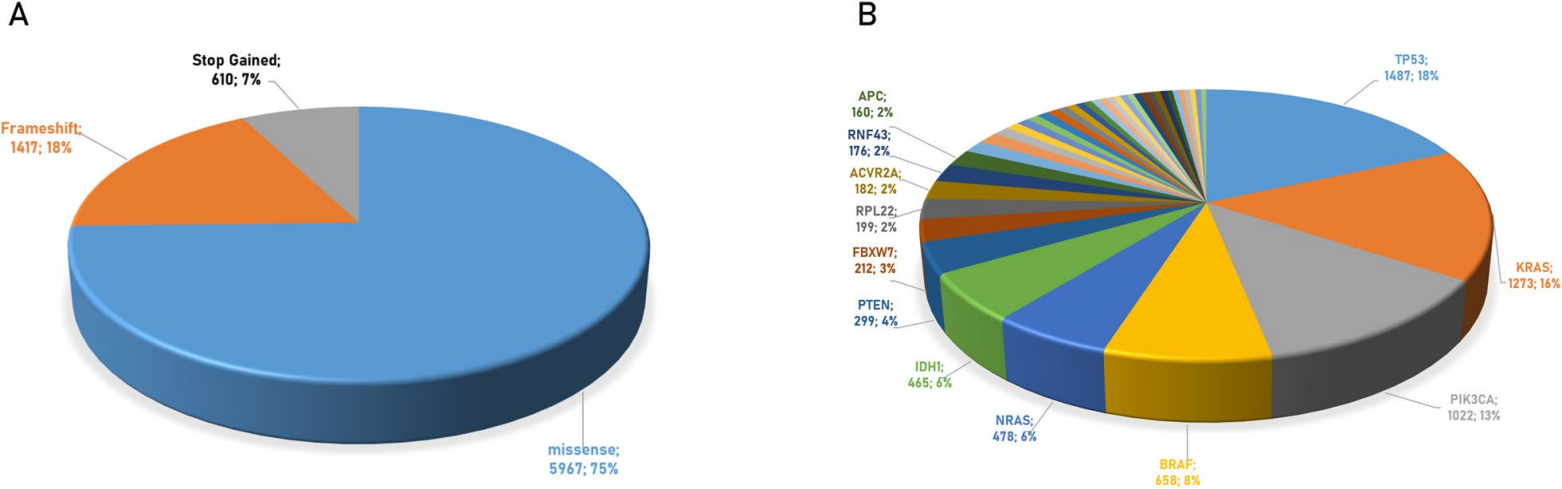
Top 100 mutations identified in cancers at TCGA database. **A** Percentage of type of mutations; **B** percentage of tumors presenting mutations of the indicated proteins

Among these 100 hot-spot mutations, 62% are missense mutations identified in 5967 cases (73.9%) and 23% are frameshift mutations identified in 1417 cases (17.55%). In addition, 13% are stop-gained mutations identified in 610 cases (7.56%) (Fig. 1A). The TP53 protein is characterized by the highest number of different mutations (nr. 20), which cumulatively are identified in the highest number of cases (1487 of the 8074 cases, 18%) (Fig. 1B; Additional file 1: Table S2). Among the 100 hot-spot mutations are included all the hot-spot mutations identified in each of the 51 primary cancer sites present in TCGA. The frequency of such hot-spot mutations in the different cancer sites is quite variable and broad, going from 3.10% of TP53_R175H_ identified in the retro peritoneum ca to 61.52% of BRAF_V640E/V600E_ in the thyroid ca. In particular, considering cancers with a high unmet clinical need (namely, < 20% 5 year overall survival — OS), the IDH1_R132H_ is found in 37.65% of brain ca; the KRAS_G12D_ is found in 32.87% of pancreas ca; the ACVR2A_K437Rfs*5_ is found in 14.13% of stomach ca (Additional file 1: Table S2).

### Selection of HLA alleles for epitope prediction from top 100 mutations.

**Fig. 2:**
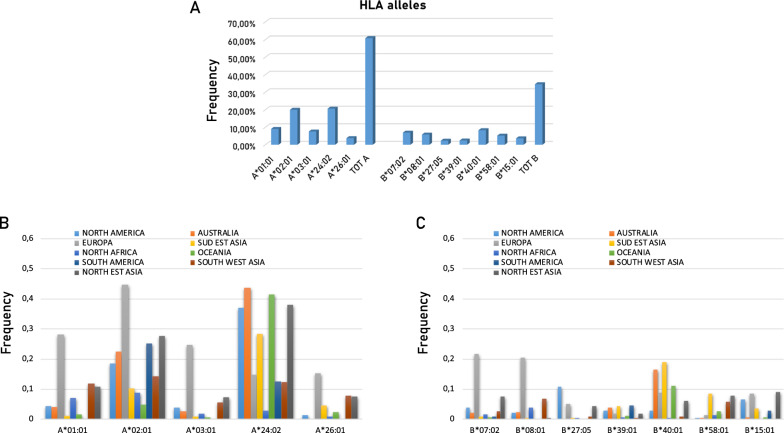
Frequency of HLA-A and B alleles considered in the study. **A** Frequency of individual alleles at global level; **B** and **C** Frequency of individual HLA-**A** and **B** alleles in each world population

In the quest for such shared TSAs, the peptide sequences including each of the top 62 missense mutations or derived from each of the 23 InDel mutations were analyzed for prediction of epitope binding to MHC class I molecules. Such analysis was performed including the 12 most frequent HLA-A and B alleles that, collectively, cover 60% (HLA-A alleles) and 35% (HLA-B alleles) of the world population (Fig. 2A). In particular, HLA-A*02:01 is present in 44% of the European population and in more than 10% in all other populations, with exception of Southeast Asian, North African and Oceania. The HLA-A*24:02 is present more than 10% in all populations, with exception of North African. Among the HLA-B alleles, the B*07:02 and 08:01 alleles show in Europeans a high prevalence of 21.8% and 20.6%, respectively. Furthermore, the B*40:01 allele shows a high prevalence in Australians (16.4%) and Southeast Asians (19.1%). All other HLA-A and B alleles show low prevalence (< 10%) across populations (Fig. 2B,C).

### Neoantigen prediction from the missense mutations.

**Fig. 3:**
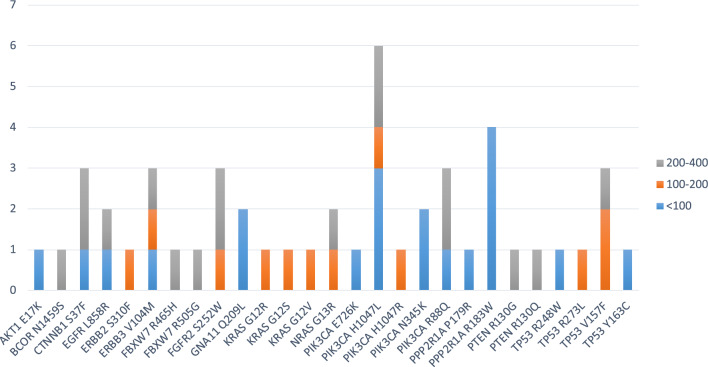
Number of predicted neoantigens from missense mutations. The number of predicted neoantigens for each missense mutations are reported. The predicted affinity of such neoantigens, expressed in nM, is indicated with color-code

In order to predict neoantigens from proteins with a single amino acid missense mutation, the amino acid sequence was downloaded from UniProt for each of the 62 proteins. A 17mer peptide was selected, centered around the mutated residue (from − 8 to + 8), and overlapping peptides were designed with the mutated residue at each of the 9 positions (Table 1). The wt and mutated peptides were subjected to the prediction analysis, to assess the affinity to the 12 HLA-A and B alleles. The results on the 945 peptides analyzed showed that only 49 mutated peptides (neoantigens) (5.18%) have an affinity < 400 nM (Fig. 3; Additional file 1: Table S3).

**Table 1:**
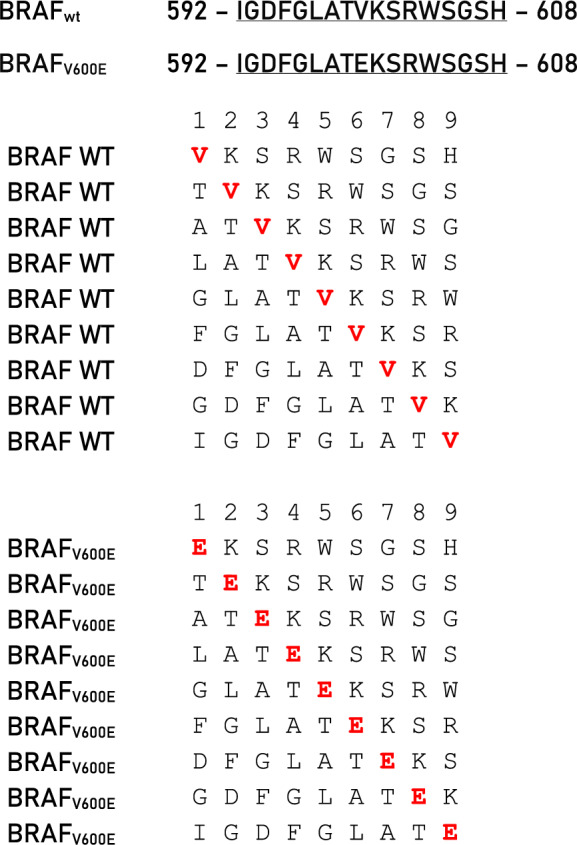
Example of overlapping peptides from wt and missense mutated protein sequences for neo-epitope prediction. Mutated aminoacid residue is indicated in bold. In each overlapping peptide, the residue involved in the missense mutation is indicated in red

**Fig. 4:**
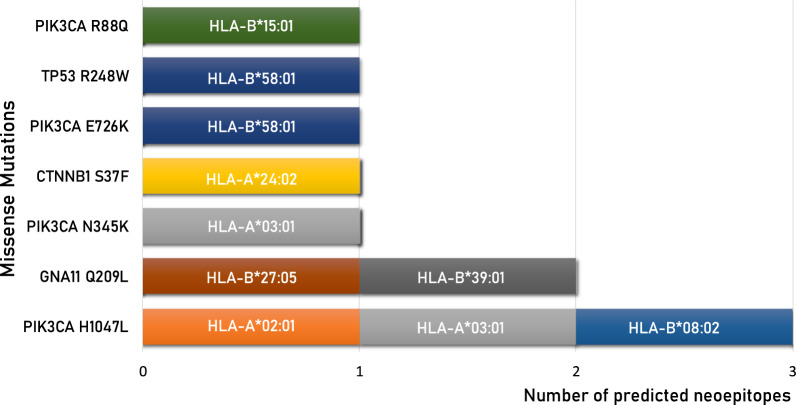
High-affinity predicted neoantigens from missense mutations and HLA restriction. The number of predicted neoantigens for each missense mutations are reported with indication of the HLA restriction

However, only 20 (2.11%) have an affinity value to the HLA alleles < 100 nM and only 10 (1.06%) can be considered optimal neoantigens. Indeed, only for these, the corresponding wt-epitope shows very low affinity values to the HLA alleles (102–41,900 nM) and are not antigenic (Table 2). Six of such neoantigens are strong binders to a single HLA allele; one epitope (GNA11_Q209L_ FRMVDVGG**L**) is a strong binder to two HLA alleles (B*27:05 and B*39:01); two epitopes derived from the same PIK3CA_H1047L_ mutation and are strong binders to three HLA alleles (FMKQMNDA**L,** A*02:01 and B*08:02; A**L**HGGWTTK, A*03:01) (Fig. 4).

**Table 2:**
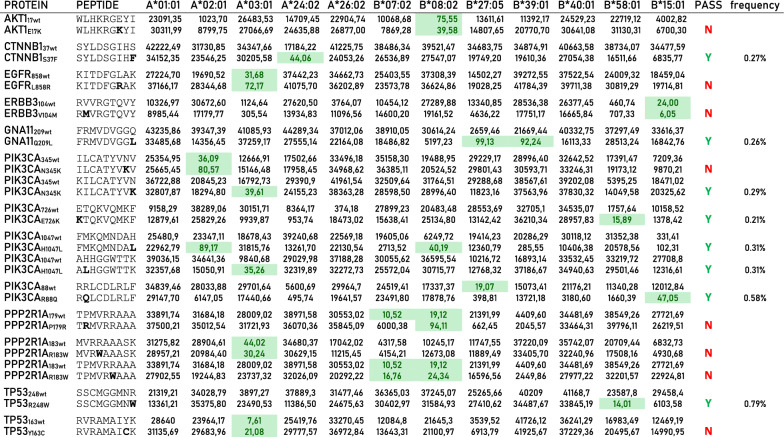
Predicted neo-epitopes derived from missense mutations with an affinity value to the HLA alleles < 100 nM (green highlighted).

The neo-epitopes pass the validation only when the corresponding wt epitope is a poor binder. The frequency of the validated neo-epitopes in the TCGA database is indicated. Identity to peptides in iedb is indicated

**Fig. 5:**
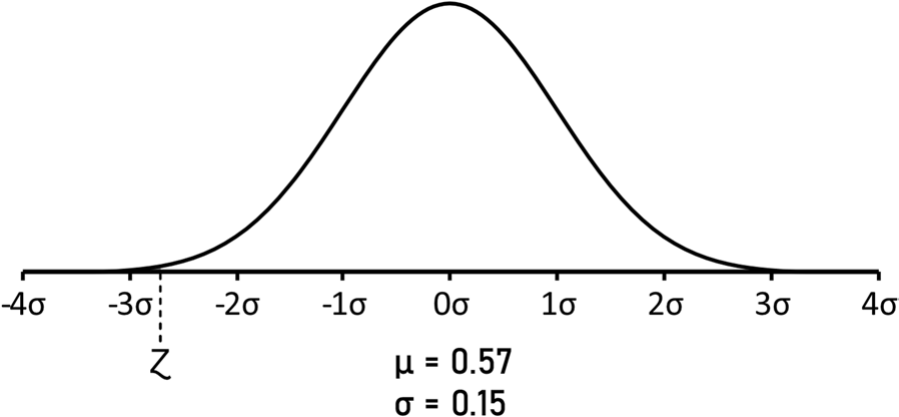
Z-score of the observed predicted neoantigens from the hot-spot missense mutations. The normal distribution of the percentage of predicted neoantigens from the 8547 samples present at TCGA. The Z-score of the observed predicted neoantigens from the hot-spot missense mutations is indicated. The result shows a statistically significant lower percentage than what expected (p-value = 0.006; 99.37% confidence level)

In order to verify the statistical significance of the observed low number of predicted neoantigens, we have considered all the mutations generating predicted epitopes in the 8547 samples present at TCGA (https://gdc.cancer.gov/about-data/publications/panimmune). Overall, 56.86% of all 1,327,063 missense mutations generate predicted epitopes. On the contrary, the 62 hot spot missense mutations analyzed in the present study generate only 10 mutations (16.13%). Therefore, the number of observed mutations is significantly lower than what expected, with a p-value = 0.006 and a 99.37% confidence level (Fig. 5).

No neoantigens were predicted for HLA-A*01:01, A*26:02, B*07:02 and B*40:01; one neoantigen was predicted for HLA-A*02.01, A*24:02, B*08:02, B*27:05, B*39:01 and B*15:01. Only for HLA-A*03:01 and B*58:01 were predicted more than a single neoantigen, three and two respectively.

The Immune Epitope Database & Tools (www.iedb.org) was interrogated in order to verify whether the predicted epitopes have been already described and validated in literature. The search returned only three peptides, the CTNNB1_S37F_ SYLDSGIH**F** peptide (PMID: 8642260; PMID:35122353), the PIK3CA_H1047L_ A**L**HGGWTTK peptide (PMID: 35484264; PMID: 37415627) and the PIK3CA_R88Q_ R**Q**LCDLRLF peptide (PMID: 37415627). The first two are confirmed to be restricted to HLA-A*24:02 and HLA-A*03:01, respectively. On the contrary, a discordance is observed for the PIK3CA_R88Q_ peptide, which has been reported as restricted to HLA-A*24:02 while our analysis predicted a very strong binding to HLA-B*15:01 (14.01 nM) and a low binding to HLA-A*24:02 (495.74 nM) (Table 2).

All the predicted neoantigens are identified in mutations identified in a very low percentage of tumor samples, ranging from 0.21% (PIK3CA_E726K_ **K**TQKVQMKF) to 0.79% (TP53_R248W_ SSCMGGMN**W**). Only the GNA11_Q209L_ FRMVDVGG**L** epitope, restricted to HLA-B*27:05 and B*39:01, is the most frequent mutation in uveal melanoma (42,50% of all cases reported in TCGA) (Additional file 2: Fig S1) (Table 3).

**Table 3:** Predicted neo-epitopes with an affinity value to the HLA alleles < 100 nM, derived from missense mutations, are listed with selected information

MISSENSE			TOT FREQ	TOP FREQ	TUMOR
PROTEIN	PEPTIDE	A*02:01			
PIK3CA_H1047L_	FMKQMNDA**L**	89.17	0.31%	N/A	N/A
**PROTEIN**	**PEPTIDE**	A*03:01			
PIK3CA_N345K_	KILCATYV**K**	39.61	0.29%	N/A	N/A
PIK3CA_H1047L_	A**L**HGGWTTK	35.26	0.31%	N/A	N/A
**PROTEIN**	**PEPTIDE**	A*24:02			
CTNNB1_S37F_	SYLDSGIHF	44.06	0.27%	N/A	N/A
**PROTEIN**	**PEPTIDE**	B*08:02			
PIK3CA_H1047L_	FMKQMNDA**L**	40.19	0.31%	N/A	N/A
**PROTEIN**	**PEPTIDE**	B*27:05			
GNA11_Q209L_	FRMVDVGG**L**	99.13	0.42%	42.50%	Eye & adnexa
**PROTEIN**	**PEPTIDE**	B*39:01			
GNA11_Q209L_	FRMVDVGG**L**	92.24	0.42%	42.50%	Eye & adnexa
**PROTEIN**	**PEPTIDE**	B*58:01			
PIK3CA_E726K_	**K**TQKVQMKF	15.89	0.21%	N/A	N/A
TP53_R248W_	SSCMGGMN**W**	14.01	1.05%	N/A	N/A
**PROTEIN**	**PEPTIDE**	B*15:01			
PIK3CA_R88Q_	R**Q**LCDLRLF	47.05	0.57%	N/A	N/A

The peptide sequences include the mutated aminoacid residue (bold & underlined). Values in the column of the haplotypes indicate the predicted affinity (nM). TOT FREQ: frequency in the TCGA database; TOP FREQ: top frequency in specific tumor; TUMOR: tumor type in which the top frequency is reported

### Neoantigen prediction from the frameshift mutations.

**Fig. 6:**
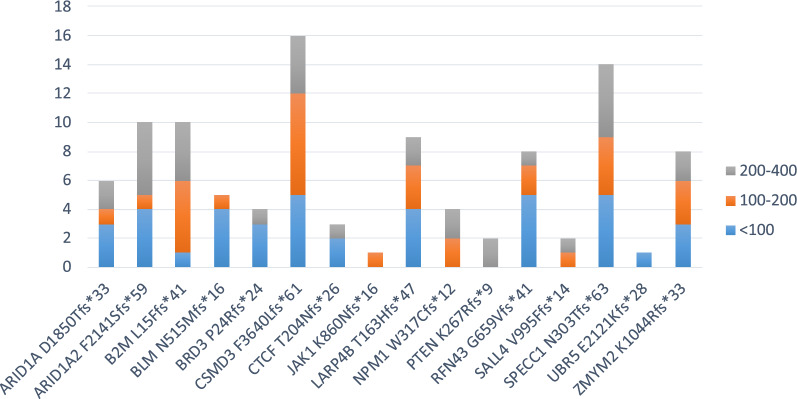
Number of predicted neoantigens from frameshift mutations. The number of predicted neoantigens for each frameshift mutations are reported. The predicted affinity of such neoantigens, expressed in nM, is indicated with color-code

Similarly, neoantigen predictions from the 23 proteins with a frameshift mutation were carried out. The amino acid sequence was downloaded from UniProt for each of the proteins but, in this case, the selection of peptides for neoantigen prediction was different for the wt and mutated sequences. Indeed, as for the missense mutations, the prediction of wt-peptides was based on a 17mer peptide, centered around the mutated residue (from − 8 to + 8), and overlapping peptides were designed with the mutated residue at each of the 9 positions (Table 4). On the contrary, the prediction of the mutated-peptides was based on a sequence starting at position − 8 from the mutated amino acid residue and including the entire downstream protein sequence. The number of mutated peptides ranged from 4 to 62, according to the position of the newly generated stop codon along the shifted reading frame. The wt and mutated peptides were subjected to the prediction analysis, to assess the affinity to the 12 HLA-A and B alleles. The results on the 686 peptides analyzed showed that 103 mutated peptides (neoantigens) (15.01%) have an affinity < 400 nM (Fig. 6; Additional file 1: Table S4).

**Table 4:**
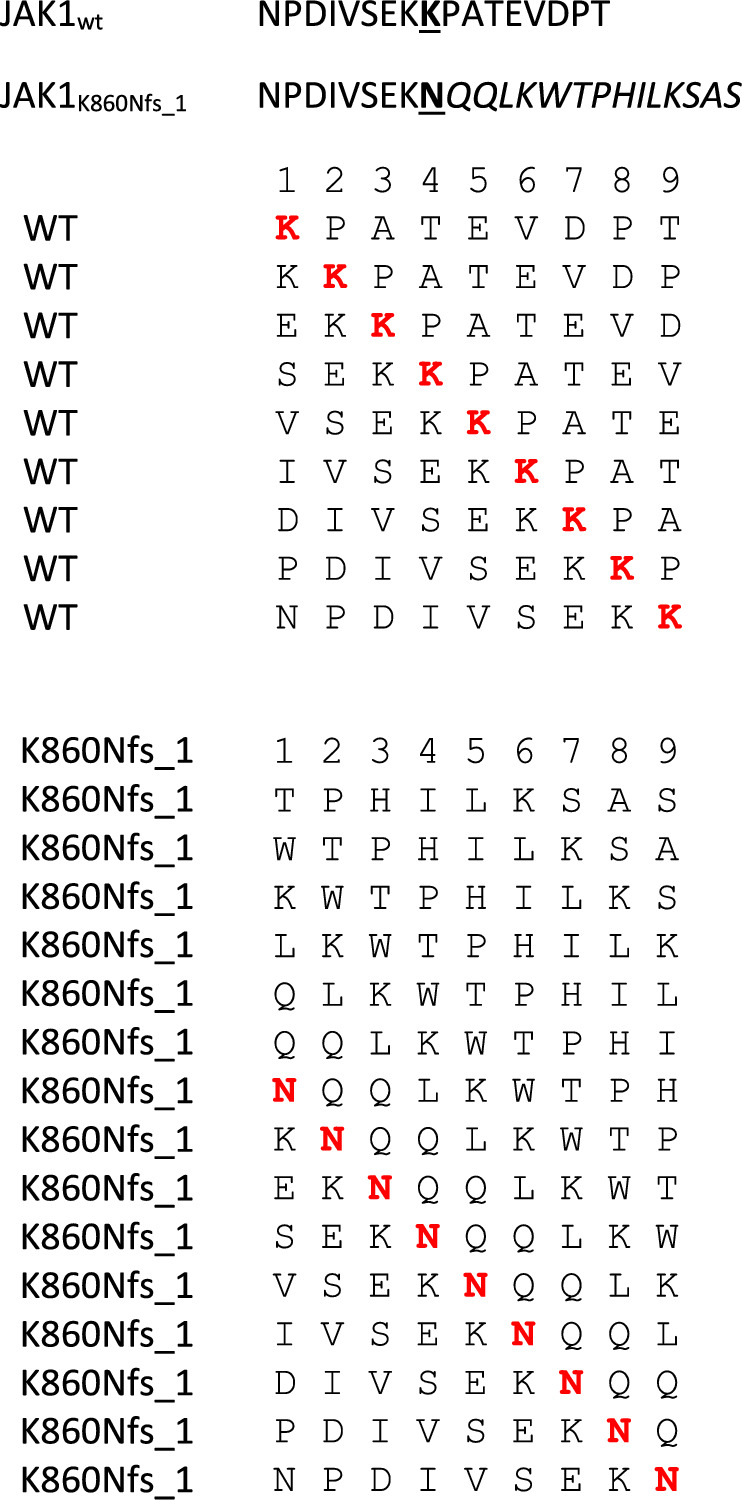
Example of overlapping peptides from wt and frameshift mutated protein sequences for neo-epitope prediction.

Mutated aminoacid residue is indicated in bold and the downstream sequence from the alternative reading frame is indicated in italics. In each overlapping peptide in the wt and mutated sequence, the mutated residue is indicated in red

**Fig. 7:**
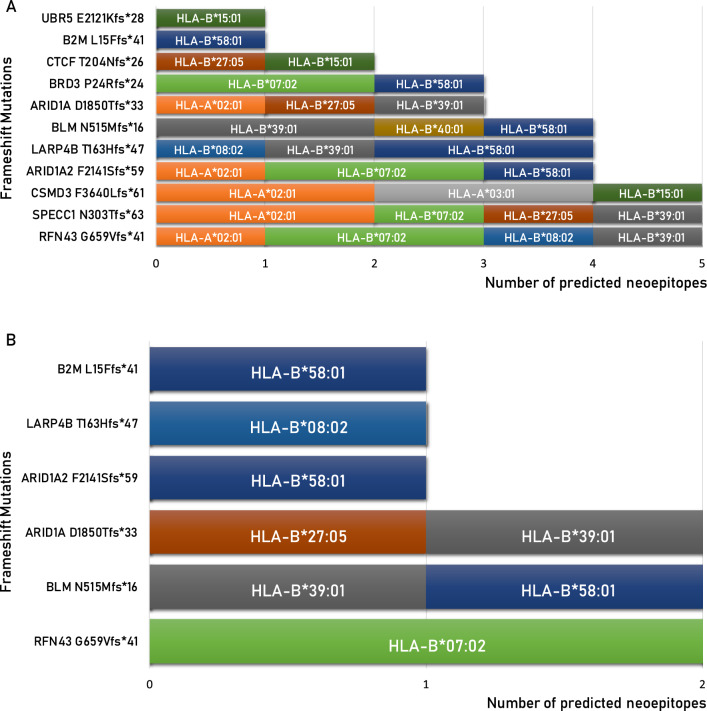
High-affinity predicted neoantigens from frameshift mutations and HLA restriction. The number of predicted neoantigens for each frameshift mutations are reported with indication of the HLA restriction, considering the total number of mutations (**A**) or only those not including the product of “abnormal” mRNA (**B**)

Of these, 40 have an affinity value to the HLA alleles < 100 nM (5.83%) and only 9 (1.31%) include the mutated residue from which the frameshift starts (Table 5). The remaining 31 mutated epitopes cover the new sequence generated by the alternative open reading frame. All of them can be considered optimal neoantigens given that the corresponding wt-epitopes either show very low affinity values to the HLA alleles (> 1000 nM), and are not antigenic, or are a completely different sequence and cannot be considered a “corresponding” epitope (Table 5). Only two of such neoantigens are strong binders to more than a single HLA allele: (RFN43_G659Vfs*41_ TQLARFFPI) is a strong binder to three HLA alleles (A*02:01, B*08:02 and B*39:01); (ARID1A_D1850Tfs*33_ WRIGGGTPL) is a strong binder to two HLA alleles (B*27:05 and B*39:01). All other epitopes are strong binders to a single HLA allele (Fig. 7A).

**Table 5:**
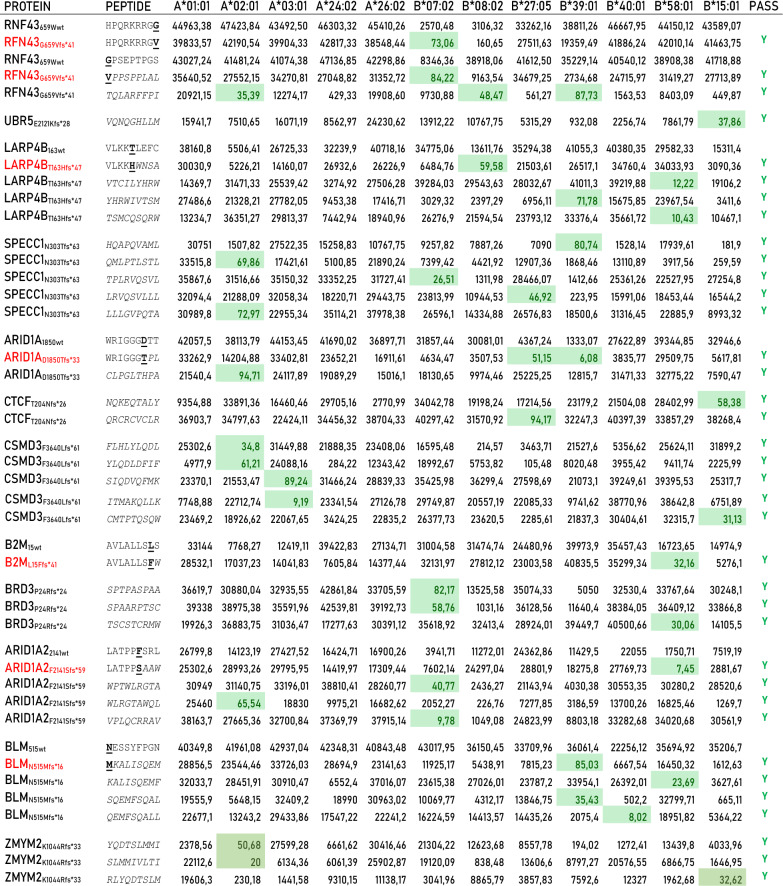
Predicted neo-epitopes derived from frameshift mutations with an affinity value to the HLA alleles < 100 nM (green highlighted)

The neo-epitopes pass the validation only when the corresponding wt epitope is a poor binder

However, the “abnormal” mRNAs generated by the frameshift contain premature termination codons (PTCs), which are recognized and degraded by nonsense-mediated mRNA decay (NMD). [15, 16] Moreover, even when PTC-containing mRNAs escape from NMD, truncated proteins are not generated due to a translational repression [17]. Therefore, these epitopes have very low or no real chance to be presented by cancer cells, implying that only 9 neoantigens (1.31%) derived from InDels could be taken into consideration (Fig. 7B).

**Fig. 8:**
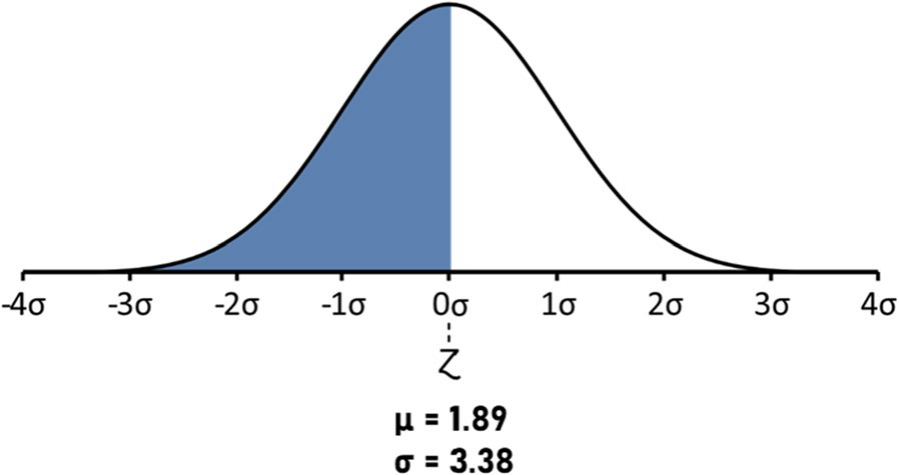
Z-score of the observed predicted neoantigens from the hot-spot InDel mutations. The normal distribution of the number of predicted neoantigens from InDel mutations in the 6610 samples present at TCGA. The Z-score of the observed predicted neoantigens from the hot-spot InDel mutations is indicated. It falls in the normal distribution of the expected values with a confidence level of 99.99% (p-value = 0.49)

Indeed, the 23 hot spot InDel mutations analyzed in the present study generate a total of 40 predicted neoantigens (1.74 per InDel), which falls in the normal distribution of the expected values derived from the 6610 samples at TCGA with a confidence level of 99.99% (Fig. 8).

No neoantigens were predicted for HLA-A*01:01, A*02:01, A*03:01, A*24:02, A*26:02, B*40:01 and B*15:01. The HLA alleles with predicted neoantigens were HLA-B*07:02, B*08:02, B*27:05, B*39:01 and B*58:01.

None of the predicted epitopes derived from the frameshift mutations were found in the Immune Epitope Database & Tools (www.iedb.org), indicating that they have not been already described and validated in literature. Moreover, all the predicted neoantigens are identified in a very low percentage of tumor samples, ranging from 0.22% (ARID1A2_F2141Sfs*59_ WLRGTAWQL, VPLQCRRAV and LATPPSAAW; BLM_N515Mfs*16_ MKALISQEM, SQEMFSQAL, QEMFSQALL and KALISQEMF); to 1.23% (RFN43_G659Vfs*41_ TQLARFFPI) (Table 6) (Additional file 1: Fig S2).

**Table 6:** Predicted neo-epitopes with an affinity value to the HLA alleles < 100 nM, derived from missense mutations, are listed with selected information.

FRAMESHIFT			TOT FREQ	TOP FREQ	TUMOR
PROTEIN	PEPTIDE	A*02:01			
RFN43 G659Vfs*41	*TQLARFFPI*	35.39	1.23%	N/A	N/A
SPECC1 N303Tfs*63	*QMLPTLSTL*	69.86	0.36%	N/A	N/A
SPECC1 N303Tfs*63	*LLLGVPQTA*	72.97	0.36%	N/A	N/A
ARID1A D1850Tfs*33	*CLPGLTHPA*	94.71	0.34%	N/A	N/A
CSMD3 F3640Lfs*61	*FLHLYLQDL*	34.80	0.28%	N/A	N/A
CSMD3 F3640Lfs*61	*YLQDLDFIF*	61.21	0.28%	N/A	N/A
ARID1A2 F2141Sfs*59	*WLRGTAWQL*	65.54	0.22%	N/A	N/A
**PROTEIN**	**PEPTIDE**	A*03:01			
CSMD3 F3640Lfs*61	*SIQDVQFMK*	89.24	0.28%	N/A	N/A
CSMD3 F3640Lfs*61	*ITMAKQLLK*	9.19	0.28%	N/A	N/A
**PROTEIN**	**PEPTIDE**	A*24:02			
N/A			N/A	N/A	N/A
**PROTEIN**	**PEPTIDE**	B*07:02			
RFN43 G659Vfs*41	HPQRKRRG**V**	73.06	1.23%	N/A	N/A
RFN43 G659Vfs*41	**V***PPSPPLAL*	84.22	1.23%	N/A	N/A
SPECC1 N303Tfs*63	*TPLRVQSVL*	26.51	0.36%	N/A	N/A
BRD3 P24Rfs*24	*SPTPASPAA*	82.17	0.24%	N/A	N/A
BRD3 P24Rfs*24	*SPAARPTSC*	58.76	0.24%	N/A	N/A
ARID1A2 F2141Sfs*59	*WPTWLRGTA*	40.77	0.22%	N/A	N/A
ARID1A2 F2141Sfs*59	*VPLQCRRAV*	9.78	0.22%	N/A	N/A
**PROTEIN**	**PEPTIDE**	B*08:02			
RFN43 G659Vfs*41	*TQLARFFPI*	48.47	1.23%	N/A	N/A
LARP4B T163Hfs*47	VLKK**H***WNSA*	59.58	0.43%	N/A	N/A
**PROTEIN**	**PEPTIDE**	B*27:05			
SPECC1 N303Tfs*63	*LRVQSVLLL*	46.92	0.36%	N/A	N/A
ARID1A D1850Tfs*33	WRIGGG**T***PL*	51.15	0.34%	N/A	N/A
CTCF T204Nfs*26	*QRCRCVCLR*	94.17	0.32%	N/A	N/A
**PROTEIN**	**PEPTIDE**	B*39:01			
RFN43 G659Vfs*41	*TQLARFFPI*	87.73	1.23%	N/A	N/A
LARP4B T163Hfs*47	*YHRWIVTSM*	71.78	0.43%	N/A	N/A
SPECC1 N303Tfs*63	*HQAPQVAML*	80.74	0.36%	N/A	N/A
ARID1A D1850Tfs*33	WRIGGG**T***PL*	6.08	0.34%	N/A	N/A
BLM N515Mfs*16	**M***KALISQEM*	85.03	0.22%	N/A	N/A
BLM N515Mfs*16	*SQEMFSQAL*	35.43	0.22%	N/A	N/A
**PROTEIN**	**PEPTIDE**	B*40:01			
BLM N515Mfs*16	*QEMFSQALL*	8.02	0.22%	N/A	N/A
**PROTEIN**	**PEPTIDE**	B*58:01			
LARP4B T163Hfs*47	*VTCILYHRW*	12.22	0.43%	N/A	N/A
LARP4B T163Hfs*47	*TSMCQSQRW*	10.43	0.43%	N/A	N/A
B2M L15Ffs*41	AVLALLS**F***W*	32.16	0.25%	N/A	N/A
BRD3 P24Rfs*24	*TSCSTCRMW*	30.06	0.24%	N/A	N/A
ARID1A2 F2141Sfs*59	LATPP**S***AAW*	7.45	0.22%	N/A	N/A
BLM N515Mfs*16	*KALISQEMF*	23.69	0.22%	N/A	N/A
**PROTEIN**	**PEPTIDE**	B*15:01			
UBR5 E2121Kfs*28	*VQNQGHLLM*	37.86	0.49%	N/A	N/A
CTCF T204Nfs*26	*NQKEQTALY*	58.38	0.32%	N/A	N/A
CSMD3 F3640Lfs*61	*CMTPTQSQW*	31.13	0.28%	N/A	N/A

The peptide sequences include the mutated aminoacid residue (bold & underlined) or the newly generated sequence downstream of the frameshift (italics). Values in the column of the haplotypes indicate the predicted affinity (nM). TOT FREQ: frequency in the TCGA database; TOP FREQ: top frequency in specific tumor; TUMOR: tumor type in which the top frequency is reported

### HLA polymorphism and neoantigen prediction in cancers.

The polymorphism of the HLA molecules taken into consideration in the present study greatly influences the array of peptides binding the HLA pocket.

**Fig. 9:**
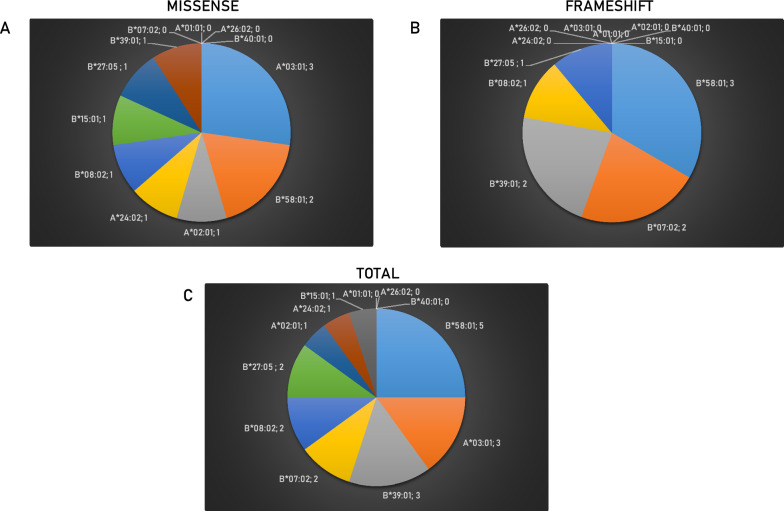
Number of predicted neoantigens for each haplotype. The number of predicted neoantigens is indicated for each of the 12 haplotypes taken into consideration. The numbers are indicated in a top-down listing in a clockwise direction. Neoantigens derived from missense mutations are listed in panel **A**; those derived from frameshift mutations are listed in panel **B**; the total neoantigens are listed in panel **C**

Considering the missense mutations, the HLA-A*03:01 and B*58:01 alleles are predicted to bind and present 3 and 2 mutated neoantigens, respectively. The HLA-A*01:01, A*26:02, B*07:02 and B*40:01 alleles do not bind and present any mutated neoantigens. The remaining ones bind and present a single mutated neoantigen (Fig. 9A). Considering the frameshift mutations, the HLA-B*58:01 binds and presents 3 mutated neoantigens while the HLA-A*01:01, A*02:01, A*03:01, A*24:02, A*26:02, B*15:01 and B*40:01 alleles do not bind and present any mutated neoantigens. The remaining ones are predicted to bind 1 or 2 mutated neoantigens (Fig. 9B). Overall, considering both types of mutations, the HLA allele predicted to bind and present the highest number of mutated neoantigens is the B*58:01 (5 neoantigens), followed by the A*03:01 and B*39:01 (3 neoantigens). The HLA-A*01:01, A*26:02, B*40:01 alleles do not bind and present any mutated neoantigens. The remaining ones are predicted to bind 1 or 2 mutated neoantigens (Fig. 9C).

**Fig. 10:**
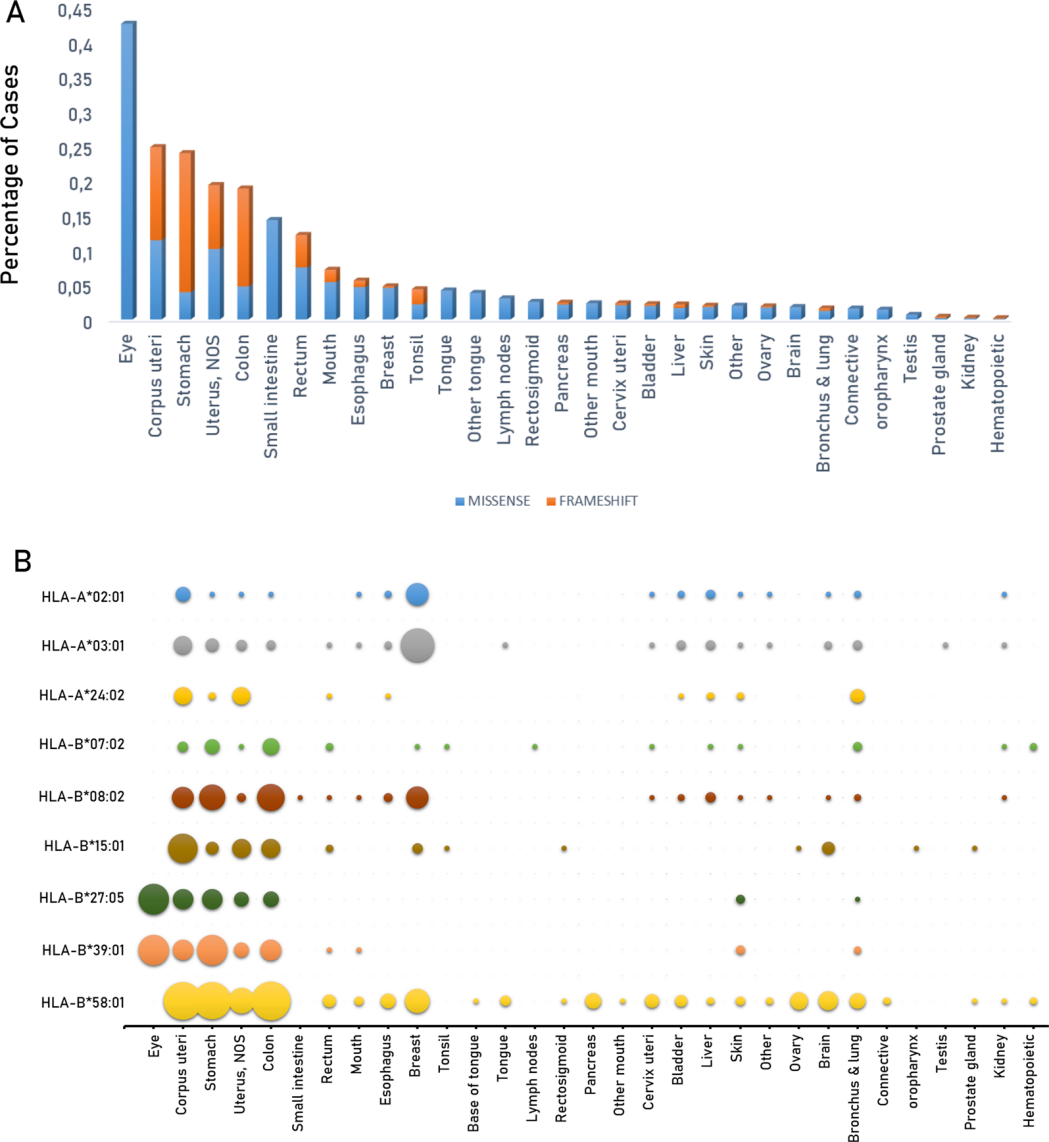
Predicted neoantigens in each tumor and haplotype. The percentage of cases with mutations predicting for neoantigens are indicated for each cancer (**A**); the percentage of neoantigens associated to each haplotype are indicated for each cancer (**B**)

Furthermore, the HLA alleles do influence the mutated proteins for which neoantigens are predicted. Indeed, 50 out of the 62 top missense mutation (80.6%) as well as 12 out of the 23 top frameshift mutations (52.2%) are not predicted to include neoantigens sequences binding to the most frequent HLA alleles. Most importantly, none of the missense and frameshift mutations identified in a relevant percentage of a specific tumor type, is predicted to include neoantigens sequences (Table 7). Looking the other way around, the percentage of tumor cases characterized by missense or frameshift mutations, generating neoantigens in specific HLA alleles, is extremely variable, ranging from 42.5% (eye) to 0.17% (hematopoietic) with an average of 6.97% and a median of 2.42%. Considering the so-called big killers, the percentage range from 18.8% (colon) to 0.4% (prostate). Furthermore, for those with a high-unmet medical need, the percentage is 2.4% for pancreatic ca and 1.78 for brain ca (Fig. 10A).

**Table 7:** Missense and frameshift mutations for which neo-epitopes have not been predicted in any of the 12 haplotypes considered in the study

MISSENSE	TOP FREQ	FRAMESHIFT	TOP FREQ
BCOR_N1459S_		ACVR2A_K437Rfs*5_	Stomach
BRAF_V640E/V600E_	Colon, Skin, Thyroid	APC_T1556Nfs*3_	
BRAF_V640M_		BCORL1_P1681Qfs*20_	
ERBB2_S310F_		GLI1_G274Afs*6_	
FBXW7_R465C_		JAK1_K860Nfs*16_	
FBXW7_R465H_		JAK1_P430Rfs*2_	
FBXW7_R479Q_		NPM1_W317Cfs*12_	
FBXW7_R505C_		PTEN_K267Rfs*9_	
FBXW7_R505G_		PTEN_T319*_	
FGFR2_S252W_		RPL22_K15Rfs*5_	Corpus Uteri
FGFR3_S249C_	Bladder	SALL4_V995Ffs*14_	
GNAQ_Q209P_		ZMYM2_K1044Rfs*33_	
HRAS_Q61R_	Adrenal gland		
IDH1_R132C_			
IDH1_R132H_	Brain		
KRAS_A146T_			
KRAS_G12A_			
KRAS_G12C_	Lung		
KRAS_G12D_	Pancreas, Rectum		
KRAS_G12R_			
KRAS_G12S_			
KRAS_G12V_			
KRAS_G13D_			
KRAS _Q61H_			
NRAS_G12D_			
NRAS_G13D_			
NRAS_G13R_			
NRAS_Q61K_			
NRAS_Q61L_			
NRAS_Q61R_	Hematopoietic		
PIK3CA_C420R_			
PIK3CA_E542K_			
PIK3CA_E545K_	Cervix uteri, Larynx		
PIK3CA_H1047R_	Breast		
POLE_P286R_			
PTEN_R130G_	Uterus		
PTEN_R130Q_			
TP53_G245S_			
TP53_H179R_			
TP53_H193R_			
TP53_I195T_			
TP53_R175H_	Ovary, Esophagus		
TP53_R248Q_			
TP53_R248W_	Bones		
TP53_R273C_			
TP53_R273H_			
TP53_R273L_			
TP53_R282W_			
TP53_V157F_			
TP53_Y220C_			

For those, which are reported as top frequent in a specific tumor, the tumor types are listed (TOP FREQ.)

However, the alleles more prevalently associated to the predicted neoantigens are not from the A locus, which overall has a 60% frequency in the general population. Indeed, most of them are predicted to be linked to alleles of the B locus, in particular HLA-B*58:01, which are among the less frequent and not equally distributed in the global population (Fig. 10B).

## Discussion

The first 100 most frequent cancer mutations reported in the TCGA database were selected to predict shared mutated neoantigens that could be useful for developing off-the-shelf cancer vaccines and/or T cell therapies. Such a selection is significantly representative of all cancer mutations. Indeed, although the first 100 mutations represent a large minority of all somatic mutations in the database (100/193,061 = 0.005%), they cover 56.65% of all identified cancer mutations. Moreover, from the 100th mutation on, each of them is identified in a number of cases lower than 29/14,254 cases and, from the 19,000th mutation, in a single case.

The majority of mutations considered for the study are missense mutations (62%). The top 100 mutations contain the most prevalent ones in the different cancer types, including those with a high unmet medical need (e.g. brain ca, pancreas ca, stomach ca). Indeed, the IDH1_R132H_ is the most prevalent mutation in brain tumors, the KRAS_G12D_ in pancreatic cancer and the ACVR2A_K437Rfs*5_ in gastric cancer, which have a 5 year relative survival rates of almost 36%, 12% and 33%, respectively. Therefore, if such mutations would generate shared tumor specific antigens (TSAs), they would be the optimal antigens for developing specific “off-the-shelf” immunotherapies for about one third of patients affected by these difficult-to treat cancers.

To perform the prediction analyses, the proteins present in the top 100 mutations were manually modified, according to the specific mutations. For the missense mutations, peptides were selected in order to have the mutated residue in each the nine positions (P_1_ to P_9_); for the frameshift mutations, peptides were selected also with the sequence downstream of the shifted reading frame. Consequently, while the 945 mutated peptides derived from the missense mutations diverged from the corresponding wt peptides only for a single amino acid, the 686 derived from the InDels included also peptides with a sequence completely different from the wt peptides.

The number of mutated peptides (neoantigens) with affinity < 400 nM to one of the 12 HLA alleles considered in the study is very low, 49 (5.18%) for the ones derived from the missense mutations and 103 (15.01%) for the ones derived from the frameshift mutations. However, the number significantly drops to 20 (2.11%) and 40 (5.83%), respectively, when considering a higher affinity of < 100 nM. Indeed, only peptides with a predicted affinity < 100 nM have been previously shown to have a 100% concordance with ex vivo binding assay [18]. Considering that a neoantigen can be classified as optimal only if the corresponding wt peptide is not antigenic, only 10 neoantigens (1.05%) are identified from the missense mutations. Likewise, also the number of neoantigens derived from the frameshift mutations with a real chance to be presented by cancer cells drops to 9 (1.31%) given that the “abnormal” mRNAs generated by the frameshift contain premature termination codons (PTCs) are recognized and degraded by nonsense-mediated mRNA decay (NMD) [15, 16]. Moreover, even when PTC-containing mRNAs escape from NMD, truncated proteins are not generated due to a translational repression [17].

Considering both types of mutations, the HLA alleles associated with the highest number of predicted neoantigens are from the B loci, namely the B*58:01 (5 epitopes), B*03:01 and B*39:01 (3 epitopes each), B*07:02, B*08:01 and B*27:05 (2 epitopes each). The HLA-A*02:01, A*24:02 and B*15:01 are associated with 1 epitope each. Of interest, three of the HLA alleles (HLA-A*01:01; A*26:02 and B*40:01) are predicted to present no mutated neoantigens. The HLA-A*02:01 and 24:02 are two of the most frequent alleles at global scale (about 40%), these findings imply that the vast majority of cancer patients at global level (> 50%) cannot benefit from tumor-specific shared mutated neoantigens.

Overall, the percentage of tumor cases characterized by missense or frameshift mutations generating neoantigens in specific HLA alleles is low, variable and associated to low-frequent HLA alleles. Indeed, the average of tumor cases is 6.97% and a median of 2.42% with a wide range going from 42.5% (eye) to 0.17% (hematopoietic). 22 out of 31 tumors (71%) show a percentage of cases characterized by mutations generating neoantigens lower than 5% and most of the big killers (e.g. breast, lung, prostate, liver ca) as well as those with a high unmet medical need (e.g. pancreas and brain ca) are in the lower part of the list (< 5%). The number of observed predicted neoantigens from the hot-spot missense mutations is significantly lower than the expected ones. On the contrary, the number of observed predicted neoantigens from the hot-spot InDel mutations is perfectly comparable to the expected ones. This supports the hypothesis that, the first ones are selected by the immunological pressure, while the latter are not because they are not translated and not presented to the immune system.

However, also the few cancers with a relevant percentage of cases (> 10%) with mutations generating neoantigens, these are associated to low prevalent HLA alleles. The GNA11_Q209L_ missense mutation, giving rise to the FRMVDVGG**L** epitope, is the most frequent mutation in uveal melanoma (UM) (42,50% of all cases reported in TCGA). Unfortunately, the clinical impact of this neoantigen appears to be very limited because UM is a rare tumor, with an average incidence rate of 5 per million globally [19] and the binding B*27:05 and B*39:01 HLA alleles are among the least frequent in the World. The two big killers colon and stomach cancers show 18.8% and 23.9% of cases which are characterized by mutations giving rise to shared neoantigens and could benefit from cancer vaccines based on TSAs. Considering that they show and age-standardized rate (ASR) of 6.2 and 6.1, respectively, this would be a huge advancement in cancer therapy. Unfortunately, such neoantigens are mostly associated to the B loci of the HLA, that show a prevalence much lower than 10%, and even lower than 1%, in world populations. This drastically reduce the potential application of such therapy. The only exception is represented by the predicted neoantigens derived from PIK3CA_H1047L_ (FMKQMNDAL) and LARP4BT163Hfs*47 (VLKKHWNSA), linked to HLA-B*08:01, as well as the one derived from RFN43G659Vfs*41 (HPQRKRRGV), linked to HLA-B*07:02. Indeed, these two alleles cover 21.8% and 20.5% of the European population and, therefore, could represent a great opportunity for providing an additional therapeutic opportunity to European patients affected by such deadly cancers.

**Fig. 11:**
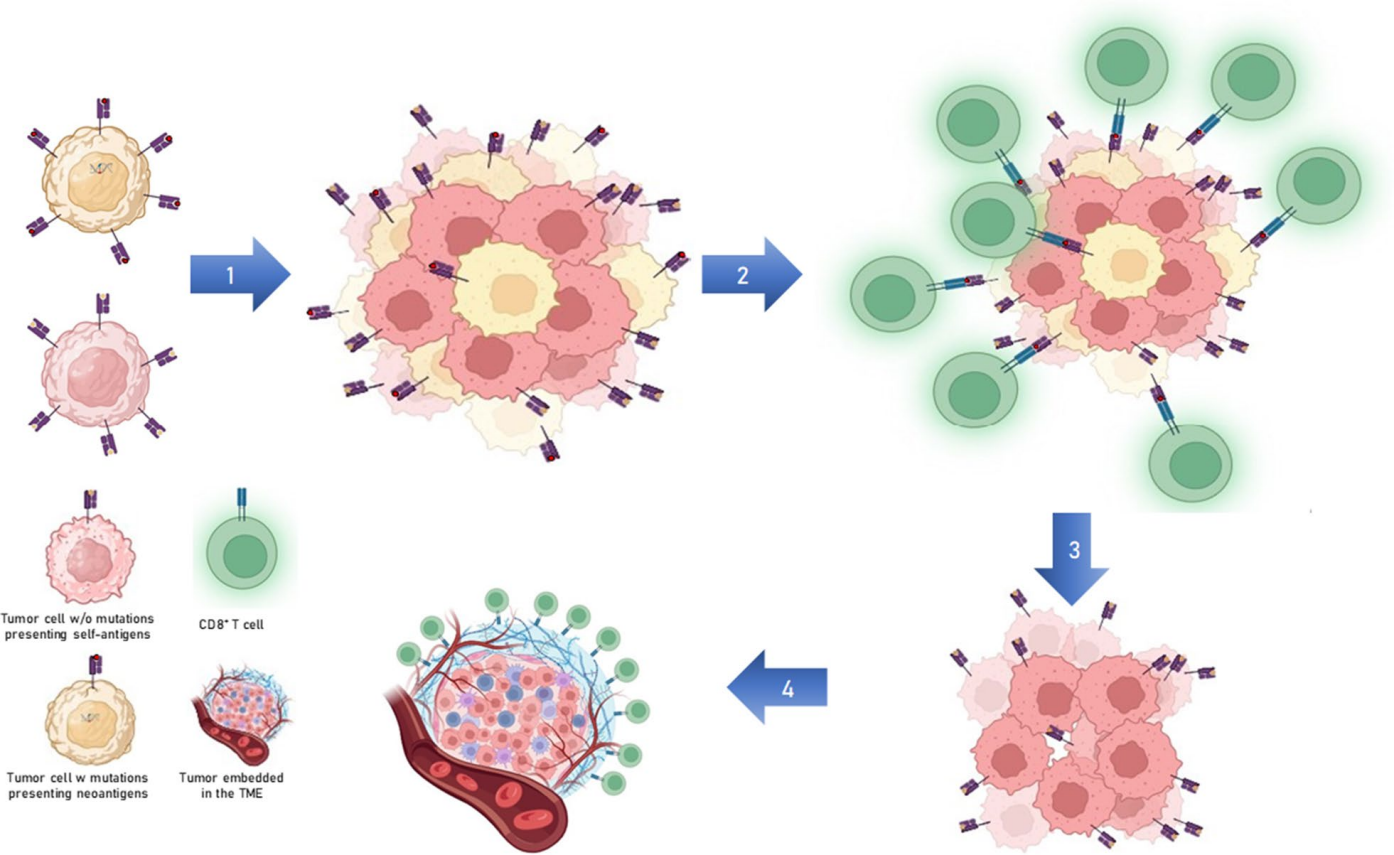
Prospected evolution of tumor lesions. Cells with mutations presenting shared neoantigens are eliminated in the very early stages of tumor lesions, when a full TME is not present. Cells not expressing shared neoantigens can outgrow without immunological control to form a tumor lesion embedded in the TME, difficult to be attacked by T cells

In conclusions, the search for shared mutated tumor-specific neoantigens for developing off-the-shelf highly specific immunotherapies results in an unfortunate failure. The most frequent mutations, either missense or InDel, do not give rise to any predicted neoantigen with high affinity to the most frequent HLA-A and B alleles. Such evidence is likely to be the result of a very strong selection by the immune system in the very early stages of tumor development, which eliminates cancer cells expressing mutated immunogenic neoantigens. At that stage, the tumor cells characterized by mutations giving rise to highly antigenic non-self-mutated neoantigens would be efficiently targeted and eliminated. The result is the selection of cancer cells expressing only wild type self-antigens and, consequently, able to escape the immune control. Finally, they will form tumor lesions embedded in a very immune-suppressive microenvironment, which is difficult to be accessed by T cells (Fig. 11).

Therefore, cancer vaccines may only rely upon personalized mutated neoantigens, with all the caveats and limitations, or upon wild type over-expressed tumor-associated antigens (TAAs), which may suffer from immunological tolerance. In order to overcome the latter drawback, non-self-antigens mimicking the TAAs (molecular mimicry), and able to elicit cross-reactive T cells, should be actively searched (i.e. antigens derived from microorganisms). This will provide the essential tool for developing off-the-shelf vaccines with the optimal immunogenicity to elicit an efficient anti-tumor T cell immune response [20–23].

### Supplementary Information

**Additional file 1: Table. S1.** List of the 100 most frequent mutations in the 14254 tumor cases reported in TCGA. **Table S2.** List of the 100 most frequent mutations reported in TCGA. **Table. S3.** List of all peptides from the wt and mutated sequences derived from proteins with missense mutations. **Table. S4.** List of all peptides from the wt and mutated sequences derived from proteins with frameshift mutations.

**Additional file 2: Fig. S1.** Percentage of mutated samples, for each tumor type, presenting the indicated missense mutation giving rise to neoantigens. **Fig. S2.** Percentage of mutated samples, for each tumor type, presenting the indicated frameshift mutation giving rise to neoantigens.

## Data Availability

Data and material will be deposited and publicly available.
