# Supplementary material for: Lack of shared neoantigens in prevalent mutations in cancer
Source: J Transl Med. 2024 Apr 10;22:344. doi: 10.1186/s12967-024-05110-0 (PMC11005154; doi:10.1186/s12967-024-05110-0)
Supplement: Supplementary file 1 — Additional file 1: Table. S1. List of the 100 most frequent mutations in the 14254 tumor cases reported in TCGA. Table S2. List of the 100 most frequent mutations reported in TCGA. Table. S3. List of all peptides from the wt and mutated sequences derived from proteins with missense mutations. Table. S4. List of all peptides from the wt and mutated sequences derived from proteins with frameshift mutations. [file 12967_2024_5110_MOESM1_ESM.docx]

**SUPPL. TABLE. 1** List of the 100 most frequent mutations in the 14254 tumor cases reported in TCGA.

| **TYPE OF MUTATION** | **MUTATION** | **CASES** | **FREQUENCY** |
| --- | --- | --- | --- |
| Missense | BRAF_V640E/V600E_ | 619 | 4.34% |
| Missense | IDH1_R132H_ | 391 | 2.74% |
| Missense | KRAS_G12D_ | 381 | 2.67% |
| Missense | PIK3CA_E545K_ | 313 | 2.20% |
| Missense | KRAS_G12V_ | 287 | 2.01% |
| Missense | PIK3CA_H1047R_ | 287 | 2.01% |
| Frameshift | RPL22_K15Rfs*5_ | 199 | 1.40% |
| Missense | PIK3CA_E542K_ | 196 | 1.38% |
| Missense | TP53_R175H_ | 195 | 1.37% |
| Missense | NRAS_Q61R_ | 184 | 1.29% |
| Frameshift | ACVR2A_K437Rfs*5_ | 182 | 1.28% |
| Frameshift | RNF43_G659Vfs*41_ | 176 | 1.23% |
| Missense | KRAS_G12C_ | 156 | 1.09% |
| Missense | TP53_R248Q_ | 149 | 1.05% |
| Missense | KRAS_G13D_ | 145 | 1.02% |
| Missense | TP53_R273C_ | 138 | 0.97% |
| Missense | NRAS_Q61K_ | 132 | 0.93% |
| Missense | TP53_R273H_ | 119 | 0.83% |
| Missense | TP53_R248W_ | 112 | 0.79% |
| Missense | TP53_R282W_ | 109 | 0.76% |
| Stop Gained | TP53_R213*_ | 89 | 0.62% |
| Missense | KRAS_Q61H_ | 82 | 0.58% |
| Missense | TP53_Y220C_ | 82 | 0.58% |
| Missense | KRAS_G12R_ | 81 | 0.57% |
| Missense | PIK3CA_R88Q_ | 81 | 0.57% |
| Frameshift | JAK1_K860Nfs*16_ | 80 | 0.56% |
| Missense | IDH1_R132C_ | 74 | 0.52% |
| Frameshift | UBR5_E2121Kfs*28_ | 70 | 0.49% |
| Missense | PTEN_R130G_ | 69 | 0.48% |
| Missense | AKT1_E17K_ | 68 | 0.48% |
| Stop Gained | TP53_R196*_ | 66 | 0.46% |
| Missense | PTEN_R130Q_ | 65 | 0.46% |
| Frameshift | BCORL1_P1681Qfs*20_ | 63 | 0.44% |
| Frameshift | LARP4B_T163Hfs*47_ | 62 | 0.43% |
| Missense | FBXW7_R465C_ | 62 | 0.43% |
| Stop Gained | APC_R1450*_ | 58 | 0.41% |
| Stop Gained | CDKN2A_R80*_ | 57 | 0.40% |
| Missense | KRAS_G12A_ | 57 | 0.40% |
| Missense | TP53_G245S_ | 56 | 0.39% |
| Missense | EGFR_L858R_ | 56 | 0.39% |
| Missense | NRAS_G12D_ | 55 | 0.39% |
| Stop Gained | PTEN_R233*_ | 52 | 0.36% |
| Frameshift | SPECC1_N303Tfs*63_ | 51 | 0.36% |
| Missense | FBXW7_R465H_ | 51 | 0.36% |
| Missense | TP53_H179R_ | 50 | 0.35% |
| Frameshift | ARID1A_D1850Tfs*33_ | 48 | 0.34% |
| Missense | KRAS_G12S_ | 47 | 0.33% |
| Intron | MYH11 | 45 | 0.32% |
| Frameshift | CTCF_T204Nfs*26_ | 45 | 0.32% |
| Missense | PIK3CA_H1047L_ | 44 | 0.31% |
| Stop Gained | TP53_R342*_ | 43 | 0.30% |
| Stop Gained | TP53_R306*_ | 43 | 0.30% |
| Missense | BCOR_N1459S_ | 42 | 0.29% |
| Missense | PIK3CA_N345K_ | 41 | 0.29% |
| Missense | ERBB2_S310F_ | 41 | 0.29% |
| Stop Gained | ARID1A_R1989*_ | 40 | 0.28% |
| Frameshift | CSMD3_F3640Lfs*61_ | 40 | 0.28% |
| Missense | BRAF_V640M_ | 39 | 0.27% |
| Frameshift | PTEN_K267Rfs*9_ | 39 | 0.27% |
| Stop Gained | PTEN_R130*_ | 38 | 0.27% |
| Missense | TP53_H193R_ | 38 | 0.27% |
| Missense | TP53_V157F_ | 38 | 0.27% |
| Frameshift | APC_T1556Nfs*3_ | 38 | 0.27% |
| Missense | CTNNB1_S37F_ | 38 | 0.27% |
| Missense | GNA11_Q209L_ | 37 | 0.26% |
| Missense | POLE_P286R_ | 37 | 0.26% |
| Missense | FGFR3_S249C_ | 37 | 0.26% |
| Missense | KRAS_A146T_ | 37 | 0.26% |
| Frameshift | PTEN_T319*_ | 36 | 0.25% |
| Missense | NRAS_G13R_ | 36 | 0.25% |
| Missense | NRAS_G13D_ | 36 | 0.25% |
| Missense | FGFR2_S252W_ | 36 | 0.25% |
| Missense | FBXW7_R479Q_ | 36 | 0.25% |
| Frameshift | GLI1_G274Afs*6_ | 35 | 0.25% |
| Frameshift | B2M_L15Ffs*41_ | 35 | 0.25% |
| Missense | NRAS_Q61L_ | 35 | 0.25% |
| Splice Region | TP53_T125=_ | 35 | 0.25% |
| Stop Gained | APC_R876*_ | 34 | 0.24% |
| Frameshift | BRD3_P24Rfs*24_ | 34 | 0.24% |
| Missense | TP53_I195T_ | 33 | 0.23% |
| Missense | PPP2R1A_P179R_ | 32 | 0.22% |
| Missense | TP53_Y163C_ | 32 | 0.22% |
| Missense | FBXW7_R505G_ | 32 | 0.22% |
| Frameshift | SALL4_V995Ffs*14_ | 32 | 0.22% |
| Frameshift | ARID1A_F2141Sfs*59_ | 31 | 0.22% |
| Missense | FBXW7_R505C_ | 31 | 0.22% |
| Stop Gained | CDKN2A_R58*_ | 31 | 0.22% |
| Frameshift | NPM1_W317Cfs*12_ | 31 | 0.22% |
| Frameshift | BLM_N515Mfs*16_ | 31 | 0.22% |
| Missense | TP53_R273L_ | 31 | 0.22% |
| Missense | PIK3CA_C420R_ | 30 | 0.21% |
| Stop Gained | APC_R1114*_ | 30 | 0.21% |
| Missense | GNAQ_Q209P_ | 30 | 0.21% |
| Missense | PIK3CA_E726K_ | 30 | 0.21% |
| Missense | HRAS_Q61R_ | 30 | 0.21% |
| Missense | ERBB3_V104M_ | 30 | 0.21% |
| Frameshift | JAK1_P430Rfs*2_ | 30 | 0.21% |
| Missense | PPP2R1A_R183W_ | 29 | 0.20% |
| Stop Gained | TP53_Q192*_ | 29 | 0.20% |
| Frameshift | ZMYM2_K1044Rfs*33_ | 29 | 0.20% |
| **TOTAL** |  | **8074** | **56.65%** |

Cases indicate the number of cancer patients with the specific mutation.

**SUPPL. TABLE 2** List of the 100 most frequent mutations reported in TCGA.

| **PROTEIN** | **TYPE OF MUTATION** | **MUTATION** | **CASES** | **TOT FREQUENCY** | **TOP FREQUENCY** | **TUMOR** |
| --- | --- | --- | --- | --- | --- | --- |
| **ACVR2A** | Frameshift | K437Rfs*5 | 182 | 1,28% | 14,13% | Stomach |
|  |  |  |  |  |  |  |
| **AKT1** | Missense | E17K | 68 | 0,48% |  |  |
|  |  |  |  |  |  |  |
| **APC** | Stop Gained | R1450* | 58 | 0,41% |  |  |
|  | Frameshift | T1556Nfs*3 | 38 | 0,27% |  |  |
|  | Stop Gained | R876* | 34 | 0,24% |  |  |
|  | Stop Gained | R1114* | 30 | 0,21% |  |  |
|  | **TOTAL** |  | **160** | **1,13%** |  |  |
|  |  |  |  |  |  |  |
| **ARID1A** | Frameshift | D1850Tfs*33 | 48 | 0,34% |  |  |
|  | Stop Gained | R1989* | 40 | 0,28% |  |  |
|  | Frameshift | F2141Sfs*59 | 31 | 0,22% |  |  |
|  | **TOTAL** |  | **119** | **0,84%** |  |  |
|  |  |  |  |  |  |  |
| **B2M** | Frameshift | L15Ffs*41 | 35 | 0,25% |  |  |
|  |  |  |  |  |  |  |
| **BCOR** | Missense | N1459S | 42 | 0,29% |  |  |
|  |  |  |  |  |  |  |
| **BCORL1** | Frameshift | P1681Qfs*20 | 63 | 0,44% |  |  |
|  |  |  |  |  |  |  |
| **BLM** | Frameshift | N515Mfs*16 | 31 | 0,22% |  |  |
|  |  |  |  |  |  |  |
| **BRAF** | Missense | V600E | 619 | 4,34% | 11,74%  41,35%  61,52% | Colon  Skin  Thyroid gland |
|  | Missense | V600M | 39 | 0,27% |  |  |
|  | **TOTAL** |  | **658** | **4,61%** |  |  |
|  |  |  |  |  |  |  |
| **BRD3** | Frameshift | P24Rfs*24 | 34 | 0,24% |  |  |
|  |  |  |  |  |  |  |
| **CDKN2A** | Stop Gained | R80* | 57 | 0,40% |  |  |
|  | Stop Gained | R58* | 31 | 0,22% |  |  |
|  | **TOTAL** |  | **88** | **0,62%** |  |  |
|  |  |  |  |  |  |  |
| **CSMD3** | Frameshift | F3640Lfs*61 | 40 | 0,28% |  |  |
|  |  |  |  |  |  |  |
| **CTCF** | Frameshift | T204Nfs*26 | 45 | 0,32% |  |  |
|  |  |  |  |  |  |  |
| **CTNNB1** | Missense | S37F | 38 | 0,27% |  |  |
|  |  |  |  |  |  |  |
| **EGFR** | Missense | L858R | 56 | 0,39% |  |  |
|  |  |  |  |  |  |  |
| **ERBB2** | Missense | S310F | 41 | 0,29% |  |  |
|  |  |  |  |  |  |  |
| **ERBB3** | Missense | V104M | 30 | 0,21% |  |  |
|  |  |  |  |  |  |  |
| **FBXW7** | Missense | R465C | 62 | 0,43% |  |  |
|  | Missense | R465H | 51 | 0,36% |  |  |
|  | Missense | R479Q | 36 | 0,25% |  |  |
|  | Missense | R505G | 32 | 0,22% |  |  |
|  | Missense | R505C | 31 | 0,22% |  |  |
|  | **TOTAL** |  | **212** | **1,48%** |  |  |
|  |  |  |  |  |  |  |
| **FGFR2** | Missense | S252W | 36 | 0,25% |  |  |
|  |  |  |  |  |  |  |
| **FGFR3** | Missense | S249C | 37 | 0,26% | 7,14% | Bladder |
|  |  |  |  |  |  |  |
| **GLI1** | Frameshift | G274Afs*6 | 35 | 0,25% |  |  |
|  |  |  |  |  |  |  |
| **GNA11** | Missense | Q209L | 37 | 0,26% | 42,50% | Uveal melanoma |
|  |  |  |  |  |  |  |
| **GNAQ** | Missense | Q209P | 30 | 0,21% |  |  |
|  |  |  |  |  |  |  |
| **MY** | Intron | H11 | 45 | 0,32% |  |  |
|  |  |  |  |  |  |  |
| **HRAS** | Missense | Q61R | 30 | 0,21% | 4,41% | Adrenal gland |
|  |  |  |  |  |  |  |
| **IDH1** | Missense | R132H | 391 | 2,74% | 37,65% | Brain |
|  | Missense | R132C | 74 | 0,52% |  |  |
|  | **TOTAL** |  | **465** | **3,26%** |  |  |
|  |  |  |  |  |  |  |
| **JAK1** | Frameshift | K860Nfs*16 | 80 | 0,56% |  |  |
|  | Frameshift | P430Rfs*2 | 30 | 0,21% |  |  |
|  | **TOTAL** |  | **110** | **0,77%** |  |  |
|  |  |  |  |  |  |  |
| **KRAS** | Missense | G12D | 381 | 2,67% | 32,87%  13,00% | Pancreas  Rectum |
|  | Missense | G12V | 287 | 2,01% |  |  |
|  | Missense | G12C | 156 | 1,09% | 7,5% | Bronchus & lung |
|  | Missense | G13D | 145 | 1,02% |  |  |
|  | Missense | Q61H | 82 | 0,58% |  |  |
|  | Missense | G12R | 81 | 0,57% |  |  |
|  | Missense | G12A | 57 | 0,40% |  |  |
|  | Missense | G12S | 47 | 0,33% |  |  |
|  | Missense | A146T | 37 | 0,26% |  |  |
|  | **TOTAL** |  | **1273** | **8,93%** |  |  |
|  |  |  |  |  |  |  |
| **LARP4B** | Frameshift | T163Hfs*47 | 62 | 0,43% |  |  |
|  |  |  |  |  |  |  |
| **NPM1** | Frameshift | W317Cfs*12 | 31 | 0,22% |  |  |
|  |  |  |  |  |  |  |
| **NRAS** | Missense | Q61R | 184 | 1,29% | 3,4% | Hematopoietic |
|  | Missense | Q61K | 132 | 0,93% |  |  |
|  | Missense | G12D | 55 | 0,39% |  |  |
|  | Missense | G13R | 36 | 0,25% |  |  |
|  | Missense | G13D | 36 | 0,25% |  |  |
|  | Missense | Q61L | 35 | 0,25% |  |  |
|  | **TOTAL** |  | **478** | **3,36%** |  |  |
|  |  |  |  |  |  |  |
| **PIK3CA** | Missense | E545K | 313 | 2,20% | 12,97%  6,31% | Cervix Uteri  Larynx |
|  | Missense | H1047R | 287 | 2,01% | 11,63% | Breast |
|  | Missense | E542K | 196 | 1,38% |  |  |
|  | Missense | R88Q | 81 | 0,57% |  |  |
|  | Missense | H1047L | 44 | 0,31% |  |  |
|  | Missense | N345K | 41 | 0,29% |  |  |
|  | Missense | C420R | 30 | 0,21% |  |  |
|  | Missense | E726K | 30 | 0,21% |  |  |
|  | **TOTAL** |  | **1022** | **7,18%** |  |  |
|  |  |  |  |  |  |  |
| **POLE** | Missense | P286R | 37 | 0,26% |  |  |
|  |  |  |  |  |  |  |
| **PPP2R1A** | Missense | P179R | 32 | 0,22% |  |  |
|  | Missense | R183W | 29 | 0,20% |  |  |
|  | **TOTAL** |  | **61** | **0,42%** |  |  |
|  |  |  |  |  |  |  |
| **PTEN** | Missense | R130G | 69 | 0,48% | 9,09% | Uterus |
|  | Missense | R130Q | 65 | 0,46% |  |  |
|  | Stop Gained | R233* | 52 | 0,36% |  |  |
|  | Frameshift | K267Rfs*9 | 39 | 0,27% |  |  |
|  | Stop Gained | R130* | 38 | 0,27% |  |  |
|  | Frameshift | T319* | 36 | 0,25% |  |  |
|  | **TOTAL** |  | **299** | **2,09%** |  |  |
|  |  |  |  |  |  |  |
| **RNF43** | Frameshift | G659Vfs*41 | 176 | 1,23% |  |  |
|  |  |  |  |  |  |  |
| **RPL22** | Frameshift | K15Rfs*5 | 199 | 1,40% | 15,18% | Corpus Uteri |
|  |  |  |  |  |  |  |
| **SALL4** | Frameshift | V995Ffs*14 | 32 | 0,22% |  |  |
|  |  |  |  |  |  |  |
| **SPECC1** | Frameshift | N303Tfs*63 | 51 | 0,36% |  |  |
|  |  |  |  |  |  |  |
| **TP53** | Missense | R175H | 195 | 1,37% | 3,48%  5,74%  3,10% | Ovary  Esophagus  Retroperitoneum |
|  | Missense | R248Q | 149 | 1,05% |  |  |
|  | Missense | R273C | 138 | 0,97% |  |  |
|  | Missense | R273H | 119 | 0,83% |  |  |
|  | Missense | R248W | 112 | 0,79% | 3,66% | Bones |
|  | Missense | R282W | 109 | 0,76% |  |  |
|  | Stop Gained | R213* | 89 | 0,62% |  |  |
|  | Missense | Y220C | 82 | 0,58% |  |  |
|  | Stop Gained | R196* | 66 | 0,46% |  |  |
|  | Missense | G245S | 56 | 0,39% |  |  |
|  | Missense | H179R | 50 | 0,35% |  |  |
|  | Stop Gained | R342* | 43 | 0,30% |  |  |
|  | Stop Gained | R306* | 43 | 0,30% |  |  |
|  | Missense | H193R | 38 | 0,27% |  |  |
|  | Missense | V157F | 38 | 0,27% |  |  |
|  | Splice Region | T125= | 35 | 0,25% |  |  |
|  | Missense | I195T | 33 | 0,23% |  |  |
|  | Missense | Y163C | 32 | 0,22% |  |  |
|  | Missense | R273L | 31 | 0,22% |  |  |
|  | Stop Gained | Q192* | 29 | 0,20% |  |  |
|  | **TOTAL** |  | **1487** | **10,43%** |  |  |
|  |  |  |  |  |  |  |
| **UBR5** | Frameshift | E2121Kfs*28 | 70 | 0,49% |  |  |
|  |  |  |  |  |  |  |
| **ZMYM2** | Frameshift | K1044Rfs*33 | 29 | 0,20% |  |  |

For each of them, the total frequency is reported together with the tumor type in which they represent most frequent mutation, if applies.

**SUPPL. TABLE. 3** List of all peptides from the wt and mutated sequences derived from proteins with missense mutations.

| **PROTEIN** | **PEPTIDE** | **A*01:01** | **A*02:01** | **A*03:01** | **A*24:02** | **A*26:02** | **B*07:02** | **B*08:02** | **B*27:05** | **B*39:01** | **B*40:01** | **B*58:01** | **B*15:01** |
| --- | --- | --- | --- | --- | --- | --- | --- | --- | --- | --- | --- | --- | --- |
| **BRAF_600wt_** | VKSRWSGSH | 40843,48 | 43920 | 33365,98 | 45333,19 | 39210,55 | 36058,26 | 34905,49 | 20073,41 | 29664,71 | 37796,76 | 39505,67 | 18785,64 |
| **BRAF_600wt_** | TVKSRWSGS | 37462,91 | 37242,26 | 24078 | 41154,9 | 18061,54 | 19518,5 | 6118,78 | 33026,91 | 42282,38 | 44674,37 | 37770,2 | 19984,99 |
| **BRAF_600wt_** | ATVKSRWSG | 32615,69 | 28803,14 | 26854,32 | 32985,85 | 30947,27 | 20634,29 | 19700,96 | 26147,56 | 33990,15 | 37492,91 | 2132,26 | 13818,01 |
| **BRAF_600wt_** | LATVKSRWS | 40112,99 | 38686,32 | 37453,18 | 45416,64 | 43917,14 | 33389,07 | 18131,63 | 36455,24 | 42282,38 | 44333,46 | 22054,04 | 35417,16 |
| **BRAF_600wt_** | GLATVKSRW | 31469,62 | 24170,66 | 22507,49 | 18076 | 29944,61 | 29791,44 | 27279,54 | 22993,63 | 39976,94 | 35884,28 | 738,27 | 6535,97 |
| **BRAF_600wt_** | FGLATVKSR | 41486,16 | 33829,46 | 21572,35 | 39428,37 | 37657,15 | 35014,8 | 26370,3 | 21171,85 | 37349,17 | 42398,3 | 30889,41 | 29824,98 |
| **BRAF_600wt_** | DFGLATVKS | 41697,69 | 40740,2 | 40963,85 | 35218,07 | 41604,4 | 42853,5 | 35725,46 | 43451,12 | 41315,95 | 46155,74 | 44342,1 | 43728,89 |
| **BRAF_600wt_** | GDFGLATVK | 34480,57 | 36910,09 | 15984,83 | 43389,57 | 38351,66 | 41068,6 | 41184,75 | 24130,15 | 41518,49 | 27959,66 | 34956,88 | 29950,44 |
| **BRAF_600wt_** | IGDFGLATV | 14741,32 | 2217,6 | 33885,14 | 24992,96 | 40584,91 | 23202,05 | 26527,12 | 34464,17 | 13503,79 | 28370,44 | 16824,19 | 28536,07 |
|  |  |  |  |  |  |  |  |  |  |  |  |  |  |
| **BRAF_V600E_** | EKSRWSGSH | 40581,4 | 46504,64 | 41017,09 | 46517,71 | 31614,34 | 38010,01 | 33454,16 | 28853,06 | 25425,27 | 38796,97 | 42898,48 | 25804,68 |
| **BRAF_V600E_** | TEKSRWSGS | 41085,93 | 44981,39 | 40374,23 | 44327,69 | 37085,82 | 35251,25 | 19045,97 | 32037,53 | 39224,13 | 30763,33 | 43020,75 | 32501,19 |
| **BRAF_V600E_** | ATEKSRWSG | 24420,92 | 36693,07 | 33396,68 | 38960,62 | 41496,95 | 28926,21 | 25419,76 | 33801,63 | 37436,97 | 40761,36 | 10569,91 | 27317,35 |
| **BRAF_V600E_** | LATEKSRWS | 40840,38 | 37177,82 | 39989,47 | 45852,62 | 42501,64 | 33875,59 | 22817,16 | 37923,75 | 41198,98 | 43646,65 | 24234,03 | 35640,89 |
| **BRAF_V600E_** | GLATEKSRW | 33573,84 | 30559,3 | 30579,47 | 27362,31 | 35243,23 | 35985,36 | 33783 | 32755,02 | 43182,55 | 39308,25 | 2204,96 | 11343,47 |
| **BRAF_V600E_** | FGLATEKSR | 42364,35 | 38380,31 | 29765,67 | 43098,53 | 42673,49 | 38630,27 | 33209,29 | 29776,94 | 40868,22 | 43609,84 | 34532,85 | 35605,44 |
| **BRAF_V600E_** | DFGLATEKS | 42503,01 | 43283,59 | 43805,16 | 37093,45 | 43837,37 | 44456,43 | 39071,64 | 44429,49 | 43247,54 | 47048,2 | 44627,03 | 44753,24 |
| **BRAF_V600E_** | GDFGLATEK | 34265,24 | 37379,51 | 14784,92 | 43522,64 | 38030,59 | 41826,02 | 42596,01 | 25799,66 | 43122,33 | 30919,16 | 36162,96 | 34275,25 |
| **BRAF_V600E_** | IGDFGLATE | 30386,85 | 31930,95 | 38381,96 | 41161,11 | 45762,9 | 38242,69 | 39504,37 | 41426,97 | 37802,9 | 41630,51 | 28082,14 | 37712,61 |
|  |  |  |  |  |  |  |  |  |  |  |  |  |  |
| **BRAF_V600M_** | MKSRWSGSH | 36374,48 | 41433,25 | 28843,07 | 43222,28 | 32436,55 | 29571,44 | 24878,3 | 11284,47 | 16800,72 | 32812,12 | 32713,94 | 9283,6 |
| **BRAF_V600M_** | TMKSRWSGS | 36091,04 | 26712,32 | 18212,63 | 36134,8 | 29968,6 | 21138,67 | 1190,02 | 24493,69 | 37772,25 | 41755,46 | 37080,61 | 10508,21 |
| **BRAF_V600M_** | ATMKSRWSG | 26462,64 | 19531,59 | 17675,46 | 22159,05 | 29738,3 | 9060,12 | 6722,66 | 15895,83 | 26415,13 | 32597,33 | 854,18 | 7932,28 |
| **BRAF_V600M_** | LATMKSRWS | 38374,91 | 37060,55 | 36316,28 | 44709,19 | 42779,36 | 30726,72 | 14223,49 | 34944,03 | 41546,8 | 43483,08 | 21143,01 | 32897,09 |
| **BRAF_V600M_** | GLATMKSRW | 29446,6 | 25878,78 | 22604,87 | 17102,61 | 31320,51 | 29354,04 | 23189,5 | 22121,19 | 40136,84 | 35844,7 | 1033,63 | 6013,38 |
| **BRAF_V600M_** | FGLATMKSR | 40675,91 | 32575,47 | 19841,07 | 39357,62 | 34675,12 | 34726,18 | 29053,86 | 20643,89 | 37869,23 | 42548,1 | 30244,17 | 30234,69 |
| **BRAF_V600M_** | DFGLATMKS | 39705,36 | 40259,93 | 39287,85 | 32829,88 | 40255,57 | 42092,04 | 33586,55 | 42336,87 | 41370,07 | 46363,47 | 43366,57 | 43035,18 |
| **BRAF_V600M_** | GDFGLATMK | 33875,59 | 37335,85 | 13923,82 | 43329,98 | 37874,14 | 42794,17 | 42126,67 | 23499,16 | 42947,73 | 32481,16 | 36576,95 | 33584,36 |
| **BRAF_V600M_** | IGDFGLATM | 11881,38 | 12840,78 | 32740,49 | 25540,24 | 34733,69 | 12560,87 | 20279,06 | 32500,85 | 7648,84 | 23939,28 | 9575,36 | 13543,74 |
|  |  |  |  |  |  |  |  |  |  |  |  |  |  |
|  |  |  |  |  |  |  |  |  |  |  |  |  |  |
| **PROTEIN** | **PEPTIDE** | **A*01:01** | **A*02:01** | **A*03:01** | **A*24:02** | **A*26:02** | **B*07:02** | **B*08:02** | **B*27:05** | **B*39:01** | **B*40:01** | **B*58:01** | **B*15:01** |
| **IDH1_132wt_** | RHAYGDQYR | 37079,41 | 36718,09 | 20660,88 | 32354,19 | 39680,01 | 36123,08 | 39122,39 | 12786,29 | 24339,14 | 38378,65 | 32588,52 | 35600,43 |
| **IDH1_132wt_** | GRHAYGDQY | 29766,30 | 43418,21 | 31018,34 | 39372,09 | 30165,09 | 39278,48 | 36049,68 | 285,72 | 23713,45 | 30052,37 | 34903,22 | 12919,65 |
| **IDH1_132wt_** | IGRHAYGDQ | 44342,10 | 44675,35 | 35348,25 | 46150,74 | 46021,10 | 39447,57 | 39437,32 | 41010,43 | 46866,32 | 46645,24 | 38802,45 | 33103,82 |
| **IDH1_132wt_** | IIGRHAYGD | 41064,60 | 33643,29 | 39672,72 | 39390,43 | 46156,25 | 42811,32 | 32874,31 | 40764,91 | 45214,62 | 46157,74 | 25757,26 | 36294,27 |
| **IDH1_132wt_** | IIIGRHAYG | 37752,20 | 18236,10 | 25445,91 | 34645,50 | 26970,21 | 23558,22 | 8676,47 | 34073,73 | 39644,41 | 41594,04 | 20528,96 | 8623,22 |
| **IDH1_132wt_** | PIIIGRHAY | 18881,21 | 38371,18 | 18804,55 | 38409,38 | 9774,99 | 30266,44 | 31603,39 | 37164,16 | 38164,98 | 41310,14 | 29919,03 | 1846,51 |
| **IDH1_132wt_** | KPIIIGRHA | 42378,56 | 35278,72 | 34842,86 | 43964,21 | 41524,36 | 584,81 | 13874,80 | 28831,52 | 27233,53 | 36157,88 | 32782,68 | 31739,44 |
| **IDH1_132wt_** | VKPIIIGRH | 43953,27 | 45166,70 | 39152,05 | 45831,78 | 39625,52 | 44981,39 | 41802,02 | 31662,59 | 43149,87 | 42280,55 | 44065,63 | 30822,29 |
| **IDH1_132wt_** | WVKPIIIGR | 37154,50 | 25620,78 | 4597,56 | 37456,41 | 11353,90 | 33034,41 | 25278,79 | 17530,90 | 38599,77 | 39450,55 | 34504,46 | 25237,52 |
|  |  |  |  |  |  |  |  |  |  |  |  |  |  |
| **IDH1_R132H_** | HHAYGDQYR | 35246,68 | 36298,6 | 25326,71 | 35638,2 | 32238,51 | 38740,34 | 37836,05 | 19131,06 | 17733,89 | 38828,49 | 34147,55 | 36059,43 |
| **IDH1_R132H_** | GHHAYGDQY | 24167 | 43119,52 | 30433,25 | 34230,79 | 29148,64 | 37831,54 | 39981,68 | 17421,79 | 15393,48 | 29633,26 | 34156,05 | 11638,23 |
| **IDH1_R132H_** | IGHHAYGDQ | 43615,98 | 44077,09 | 38838,98 | 45762,4 | 46101,84 | 43502,85 | 42118,46 | 41912,99 | 46243,73 | 46713,42 | 38528,01 | 35632,04 |
| **IDH1_R132H_** | IIGHHAYGD | 41269,48 | 31624,94 | 39672,29 | 40367,23 | 45804,54 | 42869,72 | 36390,22 | 42261,8 | 45552,46 | 45834,26 | 27785,96 | 35731,24 |
| **IDH1_R132H_** | IIIGHHAYG | 36605,05 | 15622,68 | 24342,3 | 34359,91 | 23315,3 | 26018,02 | 16627,65 | 34778,45 | 38441,39 | 39565,13 | 17409,74 | 6172,24 |
| **IDH1_R132H_** | PIIIGHHAY | 14772,92 | 36769,39 | 20548,52 | 34893,41 | 8316,7 | 35232,57 | 32734,11 | 37617,66 | 37171,8 | 40013,72 | 25191,97 | 1528,5 |
| **IDH1_R132H_** | KPIIIGHHA | 40256,02 | 31209,89 | 34381,1 | 41349,93 | 40696,59 | 757,61 | 11921,04 | 28610,27 | 18996,98 | 34223,74 | 27598,99 | 31833,68 |
| **IDH1_R132H_** | VKPIIIGHH | 44304,2 | 45030,09 | 39551,86 | 46155,74 | 40003,76 | 44746,95 | 41622,86 | 33805,66 | 42555,92 | 42248,99 | 44042,28 | 26641,61 |
| **IDH1_R132H_** | WVKPIIIGH | 35802,08 | 30966,04 | 10643,47 | 43882,94 | 7166,97 | 27895,3 | 22344,42 | 27203,8 | 37386,38 | 35138,92 | 29895,41 | 5598,51 |
|  |  |  |  |  |  |  |  |  |  |  |  |  |  |
| **IDH1_R132C_** | CHAYGDQYR | 31490,07 | 34979,59 | 25085,07 | 30991,17 | 34533,58 | 39809,02 | 38509,66 | 19550,19 | 24376,83 | 41009,11 | 34252,27 | 36757,85 |
| **IDH1_R132C_** | GCHAYGDQY | 16416 | 40951,02 | 29835,29 | 44767,27 | 31531,67 | 39987,74 | 41399,62 | 34913,42 | 41980,62 | 36633,18 | 21342,06 | 6566,66 |
| **IDH1_R132C_** | IGCHAYGDQ | 42501,18 | 43290,15 | 38570,55 | 45908,22 | 46941,91 | 45674,36 | 44555,62 | 42851,17 | 46882,55 | 47062,95 | 36962,85 | 38508,84 |
| **IDH1_R132C_** | IIGCHAYGD | 39305,7 | 30566,58 | 39204,18 | 38431,41 | 45488,43 | 42463,48 | 35720,43 | 40642,89 | 44635,23 | 45502,72 | 25943,53 | 34913,42 |
| **IDH1_R132C_** | IIIGCHAYG | 35231,8 | 9616,58 | 20698,69 | 34240,79 | 29798,2 | 28528,96 | 25622,17 | 33763,63 | 37753,03 | 39698,06 | 13264,71 | 7577,42 |
| **IDH1_R132C_** | PIIIGCHAY | 16555,31 | 33418,35 | 22064,07 | 37706,1 | 11584,84 | 38282,41 | 35472,39 | 39186,38 | 40417,06 | 41590,9 | 29875,03 | 2088,87 |
| **IDH1_R132C_** | KPIIIGCHA | 39563,82 | 30144,53 | 32871,83 | 43123,72 | 41092,15 | 1108,01 | 12840,23 | 28814,68 | 21033,95 | 33830,92 | 27676,74 | 27207,32 |
| **IDH1_R132C_** | VKPIIIGCH | 43560,8 | 45233,22 | 39302,3 | 45874,45 | 39716,52 | 44235,22 | 39569,82 | 34077,79 | 40830,66 | 41332,05 | 44355,05 | 28499,05 |
| **IDH1_R132C_** | WVKPIIIGC | 40772,85 | 16780,01 | 31812,66 | 42095,22 | 26663,24 | 26434,87 | 14028,47 | 35043,98 | 34675,88 | 36612,58 | 30474,77 | 21498,26 |
|  |  |  |  |  |  |  |  |  |  |  |  |  |  |
|  |  |  |  |  |  |  |  |  |  |  |  |  |  |
| **PROTEIN** | **PEPTIDE** | **A*01:01** | **A*02:01** | **A*03:01** | **A*24:02** | **A*26:02** | **B*07:02** | **B*08:02** | **B*27:05** | **B*39:01** | **B*40:01** | **B*58:01** | **B*15:01** |
| **KRAS_12wt_** | GGVGKSALT | 42555,02 | 32717,12 | 40529,61 | 44292,22 | 44048,95 | 38240,62 | 35041,34 | 38614,4 | 41036,17 | 42233 | 33025,84 | 35819,88 |
| **KRAS_12wt_** | AGGVGKSAL | 41395,59 | 37727,72 | 40048,37 | 40498,05 | 45135,46 | 8070,71 | 12928,19 | 38101,85 | 26438,29 | 37266,84 | 34331,66 | 26978,68 |
| **KRAS_12wt_** | GAGGVGKSA | 41691,82 | 36104,71 | 41513,57 | 46064,96 | 42863,22 | 19759,45 | 31377,84 | 36937,66 | 36857,02 | 38797,4 | 30676,91 | 27374,15 |
| **KRAS_12wt_** | VGAGGVGKS | 40112,99 | 38122,05 | 38782,29 | 43760,15 | 42342,36 | 37437,38 | 41561,65 | 40982,03 | 44009,91 | 44638,6 | 32018,47 | 34476,11 |
| **KRAS_12wt_** | VVGAGGVGK | 33041,2 | 33838,23 | 247,59 | 42170,89 | 37006,05 | 33608,35 | 41400,53 | 31974,51 | 44932,25 | 43724,63 | 30976,42 | 27511,63 |
| **KRAS_12wt_** | VVVGAGGVG | 39796,54 | 35040,19 | 36348,9 | 43699,57 | 29721,89 | 21409,82 | 36999,65 | 38520,51 | 39166,45 | 39605,39 | 23915,21 | 8184,41 |
| **KRAS_12wt_** | LVVVGAGGV | 30656,66 | 3818,17 | 30038,73 | 40547,6 | 8683,6 | 21639,46 | 26665,54 | 30907,12 | 30697,83 | 34869,63 | 24690,85 | 10748,43 |
| **KRAS_12wt_** | KLVVVGAGG | 40247,73 | 7808,12 | 24998,36 | 40992,68 | 40701 | 33425,96 | 32631,57 | 27047,95 | 39820,21 | 36296,64 | 19771 | 5358,07 |
| **KRAS_12wt_** | YKLVVVGAG | 43169,94 | 38426 | 41986,07 | 42259,05 | 42367,57 | 35876,52 | 20889,02 | 17249,05 | 17408,22 | 31170,75 | 34030,62 | 23499,68 |
|  |  |  |  |  |  |  |  |  |  |  |  |  |  |
| **KRAS_G12D_** | DGVGKSALT | 41713,02 | 40144,25 | 43706,67 | 43791,88 | 36965,24 | 38631,52 | 25051,44 | 43426,66 | 36247,98 | 44808,46 | 39858,6 | 42436,38 |
| **KRAS_G12D_** | ADGVGKSAL | 40581,4 | 41470,03 | 42758,09 | 43030,51 | 42415,25 | 23436,96 | 24586,36 | 41424,72 | 33353,69 | 18514,85 | 43235,86 | 36373,31 |
| **KRAS_G12D_** | GADGVGKSA | 29969,57 | 32620,97 | 40211,18 | 45982,29 | 42342,36 | 21777,57 | 34488,4 | 38356,23 | 27472,36 | 31865,72 | 30514,7 | 32902,77 |
| **KRAS_G12D_** | VGADGVGKS | 39982,97 | 38789,42 | 40812,57 | 44543,56 | 42193,25 | 39426,23 | 42247,63 | 42378,56 | 44320,97 | 44522,36 | 33534,26 | 35535,4 |
| **KRAS_G12D_** | VVGADGVGK | 33577,11 | 35485,07 | 1104,62 | 44031,32 | 38243,5 | 37932,78 | 43687,76 | 35680,26 | 45951,46 | 44619,78 | 31725,71 | 31581,51 |
| **KRAS_G12D_** | VVVGADGVG | 39979,96 | 35733,96 | 39973,9 | 44914,75 | 35498,11 | 31815,41 | 39659,83 | 41137,52 | 40877,53 | 40745,48 | 20626,26 | 13231,74 |
| **KRAS_G12D_** | LVVVGADGV | 27214,97 | 1973,93 | 34742,71 | 39045,87 | 17919,44 | 29821,1 | 31481,89 | 33016,54 | 26708,28 | 32563,86 | 18829,19 | 15519,4 |
| **KRAS_G12D_** | KLVVVGADG | 41160,23 | 16029,34 | 31676,99 | 42381,79 | 43338,42 | 37152,08 | 36157,48 | 33830,92 | 42568,36 | 39119,88 | 24615,37 | 9848,77 |
| **KRAS_G12D_** | YKLVVVGAD | 44791,99 | 40800,2 | 44021,32 | 43692,5 | 44509,36 | 40374,68 | 30692,17 | 26314,73 | 25449,77 | 33478,79 | 35716,57 | 34014,05 |
|  |  |  |  |  |  |  |  |  |  |  |  |  |  |
| **KRAS_G12V_** | VGVGKSALT | 40862,93 | 29738,61 | 38393,6 | 40445,07 | 43182,11 | 35602,75 | 30025,4 | 38610,64 | 39064,89 | 42573,43 | 28909,31 | 32852,63 |
| **KRAS_G12V_** | AVGVGKSAL | 38116,7 | 23090,86 | 31331,36 | 38846,55 | 34367,35 | 855,43 | 8593,04 | 34918,71 | 24280,49 | 30522,29 | 31700,31 | 10315,36 |
| **KRAS_G12V_** | GAVGVGKSA | 40835,08 | 29895,08 | 39329,1 | 45857,09 | 32529,33 | 14990,62 | 29176,72 | 32327,24 | 29396,62 | 32781,96 | 25809,71 | 17410,68 |
| **KRAS_G12V_** | VGAVGVGKS | 40461,68 | 39026,86 | 38929,43 | 43283,12 | 44389,13 | 39565,54 | 40498,91 | 40802,84 | 44229 | 44874,94 | 30555,66 | 34783,72 |
| **KRAS_G12V_** | VVGAVGVGK | 32152,14 | 30145,19 | 134,21 | 39971,29 | 34139,05 | 33829,81 | 40613,45 | 27856,1 | 44399,21 | 42673,96 | 26416,29 | 25475,12 |
| **KRAS_G12V_** | VVVGAVGVG | 39701,49 | 28413,77 | 34173,41 | 41651,24 | 23983,09 | 26355,19 | 36708,98 | 38318,9 | 39205,47 | 38743,29 | 17634,96 | 7097,45 |
| **KRAS_G12V_** | LVVVGAVGV | 31963,09 | 552,52 | 28085,18 | 39714,37 | 16672,87 | 27014,89 | 25742,77 | 32966,22 | 26478,09 | 32184,85 | 18717,27 | 10383,77 |
| **KRAS_G12V_** | KLVVVGAVG | 40182,04 | 8434,42 | 25087,79 | 39439,46 | 40355,02 | 28620,18 | 27922,48 | 27973,27 | 35831,9 | 32514,92 | 15826,85 | 1981,12 |
| **KRAS_G12V_** | YKLVVVGAV | 36357,17 | 13418,88 | 38305,21 | 36342,61 | 32523,35 | 25692,41 | 7515,04 | 8282,31 | 1104,78 | 15432 | 35115,72 | 15032,53 |
|  |  |  |  |  |  |  |  |  |  |  |  |  |  |
| **KRAS_G12C_** | CGVGKSALT | 36773,76 | 27323,55 | 37736,29 | 39141,05 | 38924,39 | 34874,53 | 25460,5 | 34666,86 | 36610,6 | 41984,71 | 26890,37 | 32060,08 |
| **KRAS_G12C_** | ACGVGKSAL | 41973,8 | 35482,76 | 42051,54 | 43558,91 | 45581,05 | 11127,93 | 17552,16 | 40786,95 | 31105,04 | 35232,57 | 36159,84 | 32212,37 |
| **KRAS_G12C_** | GACGVGKSA | 40557,7 | 32732,34 | 38578,48 | 46032,55 | 41800,21 | 24634,81 | 32524,76 | 34247,46 | 34480,95 | 36789,27 | 26313,31 | 21632,42 |
| **KRAS_G12C_** | VGACGVGKS | 38653,7 | 37624,16 | 38800,35 | 43005,85 | 43611,25 | 39572,83 | 40696,59 | 39358,48 | 43493,45 | 44333,92 | 31256,52 | 34151,98 |
| **KRAS_G12C_** | VVGACGVGK | 30607,94 | 30282,48 | 214,77 | 39917,27 | 37710,57 | 33118,52 | 39905,61 | 25758,09 | 45074,43 | 43899,56 | 28004,77 | 26503,61 |
| **KRAS_G12C_** | VVVGACGVG | 38495,5 | 27593,32 | 32551,18 | 41030,85 | 30157,58 | 27699,81 | 34407,89 | 37148,08 | 38811,69 | 38971,58 | 18945,46 | 7411,52 |
| **KRAS_G12C_** | LVVVGACGV | 27350,17 | 596,43 | 26974,59 | 38316,4 | 14390,04 | 23005,32 | 20886,31 | 30553,02 | 25343,14 | 33928,06 | 17070,26 | 7631,39 |
| **KRAS_G12C_** | KLVVVGACG | 39125,38 | 10844,33 | 26786,42 | 39896,12 | 41089,48 | 33554,6 | 28853,68 | 30472,79 | 40033,62 | 37682,03 | 19310,74 | 5025,14 |
| **KRAS_G12C_** | YKLVVVGAC | 45008,18 | 34629,37 | 43625,4 | 43477,92 | 44702,9 | 35646,68 | 18058,02 | 23469,95 | 11946,99 | 31571,96 | 40926,64 | 31056,29 |
|  |  |  |  |  |  |  |  |  |  |  |  |  |  |
| **KRAS_G12R_** | RGVGKSALT | 41151,32 | 28548,42 | 33343,58 | 37908,18 | 42489,23 | 27264,2 | 26554,7 | 29134,77 | 37063,37 | 39287,42 | 22981,44 | 28279,11 |
| **KRAS_G12R_** | ARGVGKSAL | 42373,97 | 39653,84 | 39360,59 | 38414,78 | 42175,46 | 11923,5 | 8736,47 | 2242,06 | 2617,24 | 26406,86 | 40761,8 | 28549,36 |
| **KRAS_G12R_** | GARGVGKSA | 42481,39 | 39251,31 | 36847,45 | 45400,92 | 38884,82 | 2549,84 | 13625,46 | 31365,62 | 37180,65 | 36940,45 | 32397,27 | 15688,92 |
| **KRAS_G12R_** | VGARGVGKS | 40213,36 | 40401,32 | 38892,82 | 43101,81 | 43591,91 | 38892,82 | 38954,72 | 39503,53 | 44217,54 | 45124,69 | 30946,27 | 35218,07 |
| **KRAS_G12R_** | VVGARGVGK | 33695,75 | 34574,34 | 135,1 | 41371,41 | 36763,42 | 31390,39 | 33432,1 | 27792,27 | 45213,16 | 44859,4 | 34495,5 | 27822,67 |
| **KRAS_G12R_** | VVVGARGVG | 41201,21 | 37592,44 | 35435,56 | 43800,41 | 33971,76 | 15644,5 | 32149,34 | 37749,77 | 39632,8 | 41437,28 | 25203,15 | 12346,63 |
| **KRAS_G12R_** | LVVVGARGV | 35471,61 | 4909,12 | 30017,93 | 40556,36 | 16923,32 | 20770,49 | 21959,28 | 30342,5 | 31731,54 | 35831,9 | 22586,04 | 11820,98 |
| **KRAS_G12R_** | KLVVVGARG | 40142,49 | 12741,83 | 27034,78 | 39671,42 | 41384,4 | 34741,21 | 33398,12 | 27134,41 | 41535,58 | 38348,34 | 18385,89 | 6894,3 |
| **KRAS_G12R_** | YKLVVVGAR | 43067,3 | 34018,11 | 26482,97 | 41713,02 | 36589,62 | 36740,36 | 26386 | 2824,38 | 24560,82 | 35381,55 | 40235,12 | 31557,61 |
|  |  |  |  |  |  |  |  |  |  |  |  |  |  |
| **KRAS_G12A_** | AGVGKSALT | 41261,44 | 29981,25 | 38575,13 | 42419,38 | 41653,5 | 34457,83 | 32018,82 | 38760,06 | 38934,5 | 41513,11 | 30658,32 | 32543,77 |
| **KRAS_G12A_** | AAGVGKSAL | 38115,05 | 34162,71 | 38243,5 | 41058,83 | 40207,26 | 888,71 | 7119,44 | 35969,8 | 16432,35 | 28935,28 | 23978,68 | 16048,08 |
| **KRAS_G12A_** | GAAGVGKSA | 38513,01 | 29005,19 | 37600,97 | 45019,84 | 34318,67 | 9726,46 | 25424,17 | 32709,34 | 27715,09 | 32239,57 | 24442,33 | 16577,53 |
| **KRAS_G12A_** | VGAAGVGKS | 39823,68 | 38188,52 | 38219,51 | 42972,36 | 43359,05 | 38170,74 | 40878,85 | 40411,82 | 44121 | 44425,65 | 29350,23 | 32386,05 |
| **KRAS_G12A_** | VVGAAGVGK | 32696,6 | 32498,38 | 225,09 | 40885,04 | 35924,68 | 32948,75 | 41630,51 | 30548,72 | 44754,68 | 43247,54 | 28785,08 | 25204,5 |
| **KRAS_G12A_** | VVVGAAGVG | 39577,96 | 32458,67 | 34956,13 | 42685,05 | 29753,09 | 25550,74 | 37270,46 | 38697,2 | 39955,31 | 39451,41 | 19041,44 | 9133,05 |
| **KRAS_G12A_** | LVVVGAAGV | 29980,92 | 558,69 | 28336,71 | 38291,53 | 8809,37 | 20987,8 | 23066,14 | 29910,62 | 25882,7 | 31841,58 | 18876,51 | 8016,4 |
| **KRAS_G12A_** | KLVVVGAAG | 39711,81 | 6501,06 | 23666,55 | 39967,4 | 39382,75 | 22935,99 | 24131,99 | 26506,46 | 33391,61 | 32396,22 | 19906,44 | 1733,47 |
| **KRAS_G12A_** | YKLVVVGAA | 40125,12 | 24557,1 | 39395,1 | 41047,26 | 37413,08 | 26770,77 | 9232,61 | 12240,08 | 3226,98 | 21331,89 | 36726,05 | 18935,42 |
|  |  |  |  |  |  |  |  |  |  |  |  |  |  |
| **KRAS_G12S_** | SGVGKSALT | 39465,92 | 29523,17 | 38192,23 | 40312,68 | 39652,11 | 31038,81 | 25676,56 | 37782,87 | 34927,79 | 41186,52 | 29607,95 | 32428,47 |
| **KRAS_G12S_** | ASGVGKSAL | 33274,76 | 33331,33 | 35178,85 | 39367,85 | 39887,91 | 3602,61 | 13783,68 | 34827,78 | 24006,72 | 31823,33 | 16971,37 | 14511,85 |
| **KRAS_G12S_** | GASGVGKSA | 39026,86 | 33094,52 | 38260,47 | 45548,02 | 38844,44 | 14124,72 | 28668,84 | 34529,86 | 30429,31 | 33503,44 | 22693,34 | 20327,6 |
| **KRAS_G12S_** | VGASGVGKS | 39722,54 | 39024,75 | 38340,45 | 43221,84 | 43399,9 | 37790,64 | 40718,61 | 40594,12 | 44113,83 | 44595,64 | 30770,31 | 33361,63 |
| **KRAS_G12S_** | VVGASGVGK | 32043,09 | 33425,96 | 154,06 | 41709,87 | 36655,39 | 33375,36 | 41239,12 | 30673,59 | 44958,51 | 43595,2 | 29356,89 | 26407,71 |
| **KRAS_G12S_** | VVVGASGVG | 39384,45 | 33027,97 | 34784,08 | 42533,37 | 29211,78 | 22721,59 | 36154,76 | 38825,11 | 38384,86 | 38849,5 | 18721,31 | 7233,9 |
| **KRAS_G12S_** | LVVVGASGV | 29972,16 | 1532,38 | 28337 | 39010,38 | 8958,65 | 18751,32 | 22728,96 | 29683,64 | 27546,18 | 33696,83 | 20252,09 | 7663,92 |
| **KRAS_G12S_** | KLVVVGASG | 39937,15 | 7827,5 | 23944,72 | 38852,43 | 39491,56 | 24467,46 | 25642,14 | 25842,12 | 35823,77 | 34063,77 | 16415,12 | 2008,53 |
| **KRAS_G12S_** | YKLVVVGAS | 42865,08 | 34296,77 | 40883,73 | 43329,06 | 41432,79 | 34547,04 | 22316,4 | 17916,92 | 14221,33 | 31127,28 | 38196,77 | 27031,28 |
|  |  |  |  |  |  |  |  |  |  |  |  |  |  |
|  |  |  |  |  |  |  |  |  |  |  |  |  |  |
| **PROTEIN** | **PEPTIDE** | **A*01:01** | **A*02:01** | **A*03:01** | **A*24:02** | **A*26:02** | **B*07:02** | **B*08:02** | **B*27:05** | **B*39:01** | **B*40:01** | **B*58:01** | **B*15:01** |
| **KRAS_13wt_** | GVGKSALTI | 33068,75 | 11060,83 | 24204,95 | 22383,87 | 36559,92 | 21243,45 | 26530,58 | 28730,33 | 32708,62 | 32115,97 | 9639,92 | 12178,26 |
| **KRAS_13wt_** | GGVGKSALT | 42555,02 | 32717,12 | 40529,61 | 44292,22 | 44048,95 | 38240,62 | 35041,34 | 38614,4 | 41036,17 | 42233 | 33025,84 | 35819,88 |
| **KRAS_13wt_** | AGGVGKSAL | 41395,59 | 37727,72 | 40048,37 | 40498,05 | 45135,46 | 8070,71 | 12928,19 | 38101,85 | 26438,29 | 37266,84 | 34331,66 | 26978,68 |
| **KRAS_13wt_** | GAGGVGKSA | 41691,82 | 36104,71 | 41513,57 | 46064,96 | 42863,22 | 19759,45 | 31377,84 | 36937,66 | 36857,02 | 38797,4 | 30676,91 | 27374,15 |
| **KRAS_13wt_** | VGAGGVGKS | 40112,99 | 38122,05 | 38782,29 | 43760,15 | 42342,36 | 37437,38 | 41561,65 | 40982,03 | 44009,91 | 44638,6 | 32018,47 | 34476,11 |
| **KRAS_13wt_** | VVGAGGVGK | 33041,2 | 33838,23 | 247,59 | 42170,89 | 37006,05 | 33608,35 | 41400,53 | 31974,51 | 44932,25 | 43724,63 | 30976,42 | 27511,63 |
| **KRAS_13wt_** | VVVGAGGVG | 39796,54 | 35040,19 | 36348,9 | 43699,57 | 29721,89 | 21409,82 | 36999,65 | 38520,51 | 39166,45 | 39605,39 | 23915,21 | 8184,41 |
| **KRAS_13wt_** | LVVVGAGGV | 30656,66 | 3818,17 | 30038,73 | 40547,6 | 8683,6 | 21639,46 | 26665,54 | 30907,12 | 30697,83 | 34869,63 | 24690,85 | 10748,43 |
| **KRAS_13wt_** | KLVVVGAGG | 40247,73 | 7808,12 | 24998,36 | 40992,68 | 40701 | 33425,96 | 32631,57 | 27047,95 | 39820,21 | 36296,64 | 19771 | 5358,07 |
|  |  |  |  |  |  |  |  |  |  |  |  |  |  |
| **KRAS_G13D_** | DVGKSALTI | 29414,13 | 26006,77 | 35749,43 | 24279,96 | 16254,29 | 26546,08 | 16119,6 | 37832,78 | 25037,89 | 38611,46 | 25352,2 | 27875,68 |
| **KRAS_G13D_** | GDVGKSALT | 41313,26 | 40754,76 | 42432,24 | 45893,33 | 40580,06 | 42342,82 | 39479,18 | 40261,26 | 42102,52 | 33712,16 | 42754,37 | 41763,59 |
| **KRAS_G13D_** | AGDVGKSAL | 31572,97 | 30485 | 38617,74 | 40687,35 | 44395,86 | 6716,99 | 18214,8 | 36970,43 | 11205,98 | 26527,41 | 31455 | 33350,81 |
| **KRAS_G13D_** | GAGDVGKSA | 41167,35 | 34336,49 | 42439,14 | 46512,2 | 42550,87 | 22918,36 | 32149,34 | 38675,02 | 36991,66 | 37878,65 | 31197,39 | 27852,47 |
| **KRAS_G13D_** | VGAGDVGKS | 41579,64 | 40294,79 | 41842,29 | 45306,71 | 43789,99 | 40818,73 | 43224,62 | 42667,96 | 45203,86 | 45689,18 | 31946,83 | 37500,21 |
| **KRAS_G13D_** | VVGAGDVGK | 33557,13 | 34585,56 | 1911,19 | 43407,88 | 39592,95 | 39072,07 | 43218,55 | 36841,46 | 45655,1 | 44723,2 | 29510,08 | 32660,18 |
| **KRAS_G13D_** | VVVGAGDVG | 39172,38 | 34941,01 | 40708,48 | 42523,71 | 36479,32 | 31767,27 | 40196,4 | 40316,6 | 37943,86 | 39379,76 | 19880,39 | 13837,77 |
| **KRAS_G13D_** | LVVVGAGDV | 31620,15 | 11985,45 | 35274,52 | 41712,13 | 19543,85 | 25775,38 | 29994,88 | 35453,2 | 34878,69 | 37795,54 | 29138,23 | 16097,3 |
| **KRAS_G13D_** | KLVVVGAGD | 42124,85 | 11792,76 | 33210,74 | 42937,04 | 43342,17 | 40481,84 | 39453,55 | 33880,72 | 43056,13 | 39362,72 | 23898,39 | 18194,72 |
|  |  |  |  |  |  |  |  |  |  |  |  |  |  |
|  |  |  |  |  |  |  |  |  |  |  |  |  |  |
| **PROTEIN** | **PEPTIDE** | **A*01:01** | **A*02:01** | **A*03:01** | **A*24:02** | **A*26:02** | **B*07:02** | **B*08:02** | **B*27:05** | **B*39:01** | **B*40:01** | **B*58:01** | **B*15:01** |
| **KRAS_61wt_** | QEEYSAMRD | 42268,21 | 45863,54 | 45973,84 | 44706,27 | 46828,28 | 45525,85 | 43199,85 | 41958,35 | 41755,92 | 26205,9 | 40112,55 | 41942,46 |
| **KRAS_61wt_** | GQEEYSAMR | 31945,45 | 23530,71 | 17336,99 | 40609,05 | 37436,18 | 40808,15 | 38219,09 | 12844,54 | 33103,82 | 23805,48 | 40446,81 | 22307,48 |
| **KRAS_61wt_** | AGQEEYSAM | 33307,18 | 30092,73 | 36777,34 | 30021,82 | 27091,59 | 11957,08 | 13548,43 | 34369,21 | 17872,19 | 28859,61 | 28108,9 | 5107,53 |
| **KRAS_61wt_** | TAGQEEYSA | 34355,08 | 33281,6 | 43295,77 | 43100,39 | 43745,93 | 31811,64 | 25047,37 | 41887,13 | 26491,56 | 40488,86 | 20610,86 | 34880,94 |
| **KRAS_61wt_** | DTAGQEEYS | 28811,26 | 41179,38 | 42076,55 | 46052 | 24936,52 | 43302,8 | 42214,73 | 45154,5 | 42635,66 | 45928,09 | 36305,67 | 42861,37 |
| **KRAS_61wt_** | LDTAGQEEY | 9306,63 | 44050,86 | 40166,83 | 43545,23 | 30732,71 | 40227,29 | 42953,3 | 39806,43 | 41172,71 | 35378,11 | 26403,71 | 20855,83 |
| **KRAS_61wt_** | ILDTAGQEE | 20706,3 | 18298,36 | 33622,92 | 43376,9 | 45039,82 | 35958,13 | 37271,26 | 40307,01 | 36807,19 | 39477,88 | 38935,76 | 34364,01 |
| **KRAS_61wt_** | DILDTAGQE | 43543,36 | 40670,61 | 40810,78 | 47314,17 | 28796,61 | 42741,44 | 41050,83 | 44945,38 | 45121,78 | 46417,17 | 44771,62 | 41269,93 |
| **KRAS_61wt_** | LDILDTAGQ | 40934,17 | 42059,27 | 42682,27 | 47125,64 | 39741,45 | 43615,5 | 43308,89 | 40482,28 | 44013,71 | 41776,71 | 39993,37 | 37257,15 |
|  |  |  |  |  |  |  |  |  |  |  |  |  |  |
| **KRAS_Q61H_** | HEEYSAMRD | 40279,1 | 45487,46 | 45448,58 | 44368,01 | 45754,49 | 43931,39 | 40924,88 | 39889,64 | 37204,78 | 22001,13 | 38019,48 | 40363,74 |
| **KRAS_Q61H_** | GHEEYSAMR | 36046,17 | 39778,89 | 31766,23 | 39598,52 | 41012,65 | 40958,54 | 40914,24 | 22077,91 | 26445,46 | 38459,7 | 42587,25 | 40723,89 |
| **KRAS_Q61H_** | AGHEEYSAM | 35121,43 | 28897,1 | 33349,37 | 31280,88 | 19988,46 | 8433,42 | 8548,99 | 31743,21 | 13520,75 | 26574,81 | 25997,76 | 2322,38 |
| **KRAS_Q61H_** | TAGHEEYSA | 32425,32 | 30253,34 | 41568,84 | 42180,93 | 43786,66 | 31742,53 | 26239,95 | 41792,53 | 28390,1 | 40858,5 | 20120,38 | 35072,45 |
| **KRAS_Q61H_** | DTAGHEEYS | 26203,06 | 38773,48 | 39986,45 | 45235,68 | 17848,81 | 41184,29 | 39185,95 | 43958,96 | 40983,8 | 44768,25 | 35279,11 | 41111,71 |
| **KRAS_Q61H_** | LDTAGHEEY | 9356,3 | 43593,31 | 38972,01 | 41069,47 | 28493,18 | 37426,45 | 40641,14 | 37763,65 | 38232,75 | 30758,33 | 25595,02 | 17283,24 |
| **KRAS_Q61H_** | ILDTAGHEE | 18087,54 | 11965,49 | 30021,82 | 41400,53 | 44529,6 | 32852,63 | 33811,51 | 38524,66 | 31948,23 | 37150,88 | 36614,95 | 33445,13 |
| **KRAS_Q61H_** | DILDTAGHE | 43400,38 | 40507,69 | 40906,27 | 47194,52 | 29556,42 | 42875,75 | 40912,47 | 45113,49 | 45341,02 | 46489,55 | 44791,5 | 39884,46 |
| **KRAS_Q61H_** | LDILDTAGH | 36278,57 | 42006,97 | 39157,56 | 47004,44 | 31376,47 | 41457,01 | 42253,57 | 39316,34 | 42706,75 | 37874,14 | 40003,32 | 27040,92 |
|  |  |  |  |  |  |  |  |  |  |  |  |  |  |
|  |  |  |  |  |  |  |  |  |  |  |  |  |  |
| **PROTEIN** | **PEPTIDE** | **A*01:01** | **A*02:01** | **A*03:01** | **A*24:02** | **A*26:02** | **B*07:02** | **B*08:02** | **B*27:05** | **B*39:01** | **B*40:01** | **B*58:01** | **B*15:01** |
| **KRAS_146wt_** | AKTRQRVED | 47304,95 | 47853,71 | 46600,35 | 47370,01 | 48018,14 | 43856,83 | 39230,51 | 35655,95 | 43084,55 | 43576,81 | 42418,02 | 45502,72 |
| **KRAS_146wt_** | SAKTRQRVE | 44670,51 | 45335,15 | 37384,35 | 46648,76 | 42940,75 | 24250,82 | 3519,77 | 38407,3 | 42811,79 | 44012,75 | 35845,49 | 29688,14 |
| **KRAS_146wt_** | TSAKTRQRV | 22246,96 | 29306,45 | 33012,26 | 32423,23 | 27220,87 | 13045,38 | 15938,72 | 30551,7 | 28739,96 | 38106,37 | 8839,17 | 22811,74 |
| **KRAS_146wt_** | ETSAKTRQR | 31726,38 | 42213,8 | 17310,75 | 43537,23 | 11351,08 | 40083,89 | 35456,27 | 37805,37 | 44203,65 | 45033,99 | 38018,25 | 40741,09 |
| **KRAS_146wt_** | IETSAKTRQ | 44116,7 | 46421,7 | 43064,96 | 47540,47 | 45686,73 | 43694,84 | 43597,11 | 39928,5 | 43865,37 | 28331,19 | 41015,74 | 39772,86 |
| **KRAS_146wt_** | FIETSAKTR | 29923,88 | 32688,82 | 18401,6 | 44034,67 | 31568,19 | 33234,09 | 33845,19 | 35074,71 | 40125,56 | 42907,78 | 41647,19 | 33954,85 |
| **KRAS_146wt_** | PFIETSAKT | 44036,09 | 36782,51 | 42988,16 | 33158,67 | 41807,46 | 42587,71 | 41463,29 | 43170,41 | 43468,05 | 45527,82 | 43437,95 | 42320,82 |
| **KRAS_146wt_** | IPFIETSAK | 36994,05 | 38406,88 | 9490,79 | 42933,77 | 35216,55 | 6079,65 | 24337,29 | 30548,72 | 32393,07 | 38393,18 | 32109,37 | 30376,33 |
| **KRAS_146wt_** | GIPFIETSA | 37711,4 | 10358,86 | 34539,95 | 40653,02 | 36069,96 | 30256,62 | 32821,01 | 40016,3 | 37029,68 | 38708,5 | 36740,76 | 21288,55 |
|  |  |  |  |  |  |  |  |  |  |  |  |  |  |
| **KRAS_A146T_** | TKTRQRVED | 46921,6 | 47962,59 | 46774,11 | 47330,04 | 48025,91 | 43479,8 | 37099,48 | 37812,73 | 40037,96 | 45277,29 | 43221,84 | 45684,73 |
| **KRAS_A146T_** | STKTRQRVE | 41690,02 | 44707,25 | 30147,47 | 45931,58 | 39369,55 | 27770,32 | 8078,22 | 38690,51 | 45027,16 | 44730,47 | 35514,26 | 28341,61 |
| **KRAS_A146T_** | TSTKTRQRV | 25280,16 | 34068,58 | 35944,91 | 35549,61 | 33738,06 | 19591,28 | 20304,74 | 31904,36 | 32995,12 | 40201,61 | 10321,95 | 30198,08 |
| **KRAS_A146T_** | ETSTKTRQR | 31589,02 | 43037,95 | 18951,21 | 43948,5 | 13638,29 | 40983,8 | 35097,5 | 38858,32 | 44520,44 | 45484 | 38866,32 | 41315,5 |
| **KRAS_A146T_** | IETSTKTRQ | 44050,86 | 46555,5 | 42974,69 | 47632,11 | 45905,73 | 43598,51 | 43390,05 | 39856,85 | 44193,61 | 30106,72 | 41188,75 | 39641,82 |
| **KRAS_A146T_** | FIETSTKTR | 28374,74 | 32579,72 | 16392,57 | 43043,56 | 28872,1 | 30962,68 | 32502,59 | 34038,71 | 39123,68 | 42791,86 | 41550,85 | 32542,36 |
| **KRAS_A146T_** | PFIETSTKT | 44106,2 | 39426,66 | 43292,97 | 34736,33 | 43219,94 | 42855,35 | 41914,8 | 43858,27 | 43835,48 | 45865,01 | 44029,9 | 42858,14 |
| **KRAS_A146T_** | IPFIETSTK | 37054,93 | 38719,81 | 10397,38 | 41783,48 | 36464,72 | 9331,43 | 27891,69 | 32412,69 | 35576,56 | 39737,58 | 29116,79 | 32362,94 |
| **KRAS_A146T_** | GIPFIETST | 41394,71 | 20479,94 | 38819,25 | 43229,3 | 40158,57 | 36463,54 | 39100,4 | 42532,46 | 41709,87 | 42051,54 | 39834,88 | 29829,48 |
|  |  |  |  |  |  |  |  |  |  |  |  |  |  |
|  |  |  |  |  |  |  |  |  |  |  |  |  |  |
| **PROTEIN** | **PEPTIDE** | **A*01:01** | **A*02:01** | **A*03:01** | **A*24:02** | **A*26:02** | **B*07:02** | **B*08:02** | **B*27:05** | **B*39:01** | **B*40:01** | **B*58:01** | **B*15:01** |
| **PIK3CA_545wt_** | EQEKDFLWS | 35105,46 | 35336,79 | 43219,94 | 42172,71 | 39352,52 | 42068,38 | 35215,42 | 36213,09 | 29700,68 | 33199,61 | 41439,53 | 34789,73 |
| **PIK3CA_545wt_** | TEQEKDFLW | 29149,9 | 38760,91 | 42745,13 | 11587,6 | 36575,74 | 37931,55 | 32108,33 | 28890,54 | 28073,03 | 9762,1 | 4199,95 | 27005,54 |
| **PIK3CA_545wt_** | ITEQEKDFL | 9242,7 | 26417,71 | 40474,84 | 35489,28 | 40661,82 | 32365,04 | 34427,27 | 39941,92 | 30058,88 | 34198,57 | 16555,31 | 36872,57 |
| **PIK3CA_545wt_** | EITEQEKDF | 38611,46 | 44521,88 | 44148,69 | 42960,28 | 13721,62 | 38948,39 | 33912,27 | 43790,92 | 43073,82 | 45337,6 | 40328,38 | 30477,74 |
| **PIK3CA_545wt_** | SEITEQEKD | 45994,22 | 46495,58 | 47038,52 | 46361,47 | 46475,97 | 46397,09 | 44110,5 | 43254,57 | 44147,72 | 25528,63 | 41462,84 | 41943,39 |
| **PIK3CA_545wt_** | LSEITEQEK | 9749,42 | 40259,93 | 15562,62 | 44863,28 | 43495,33 | 42029,25 | 42825,68 | 38548,84 | 43926,66 | 43521,68 | 20559,64 | 37190,7 |
| **PIK3CA_545wt_** | PLSEITEQE | 39979,52 | 27966,02 | 40291,31 | 43355,78 | 43888,63 | 44970,2 | 43486,86 | 44206,53 | 45279,25 | 45233,72 | 38928,18 | 38367,02 |
| **PIK3CA_545wt_** | DPLSEITEQ | 46304,31 | 47093,52 | 44017,52 | 48227,96 | 40825,81 | 34985,27 | 37766,52 | 44904,06 | 41936,57 | 46514,71 | 45094,44 | 43284,99 |
| **PIK3CA_545wt_** | RDPLSEITE | 44086,16 | 44344,01 | 45000,37 | 44491,54 | 47044,65 | 45036,92 | 44129,59 | 44297,96 | 45559,36 | 41421,14 | 43531,11 | 43306,54 |
|  |  |  |  |  |  |  |  |  |  |  |  |  |  |
| **PIK3CA_E545K_** | KQEKDFLWS | 38087,41 | 26227,75 | 36176,66 | 38052 | 44214,65 | 41420,23 | 36747,92 | 23106,1 | 34621,89 | 27676,44 | 33345,39 | 27171,13 |
| **PIK3CA_E545K_** | TKQEKDFLW | 38337,96 | 41133,96 | 43616,43 | 17695,17 | 40943,49 | 40530,06 | 27858,5 | 20111,25 | 24821,84 | 33468,64 | 3178,57 | 30716,76 |
| **PIK3CA_E545K_** | ITKQEKDFL | 28134,46 | 29857,91 | 37414,31 | 28725,97 | 35741,68 | 24878,03 | 16694,9 | 37296,29 | 35793,16 | 35157,55 | 10664,56 | 19360,32 |
| **PIK3CA_E545K_** | EITKQEKDF | 38986,34 | 45590,93 | 43888,18 | 42690,12 | 20035,44 | 38517,16 | 31151,2 | 43302,8 | 44219,45 | 46004,18 | 39502,23 | 30277,25 |
| **PIK3CA_E545K_** | SEITKQEKD | 45401,42 | 46798,43 | 46314,34 | 46242,71 | 46185,22 | 45638,29 | 40794,89 | 41408,14 | 43246,15 | 26080,6 | 42361,15 | 42384,98 |
| **PIK3CA_E545K_** | LSEITKQEK | 14885,89 | 41534,67 | 11423,77 | 44591,3 | 43543,83 | 39086,86 | 40182,04 | 36669,68 | 43855,89 | 43675,48 | 23408,82 | 36441,45 |
| **PIK3CA_E545K_** | PLSEITKQE | 43179,29 | 33801,28 | 39600,66 | 45431,88 | 44787,62 | 44807,96 | 42170,46 | 44643,93 | 46691,71 | 46348,91 | 41897,13 | 39442,87 |
| **PIK3CA_E545K_** | DPLSEITKQ | 46740,21 | 47676,98 | 44305,65 | 48430,34 | 41897,56 | 38218,28 | 40122,09 | 45368,02 | 44664,69 | 47359,75 | 45650,16 | 44829,79 |
| **PIK3CA_E545K_** | RDPLSEITK | 38210 | 43190,49 | 27976,92 | 41001,12 | 44509,36 | 43571,64 | 43991,8 | 37299,51 | 45625,46 | 41229,31 | 38647,84 | 39624,24 |
|  |  |  |  |  |  |  |  |  |  |  |  |  |  |
|  |  |  |  |  |  |  |  |  |  |  |  |  |  |
| **PROTEIN** | **PEPTIDE** | **A*01:01** | **A*02:01** | **A*03:01** | **A*24:02** | **A*26:02** | **B*07:02** | **B*08:02** | **B*27:05** | **B*39:01** | **B*40:01** | **B*58:01** | **B*15:01** |
| **PIK3CA_1047wt_** | HHGGWTTKM | 30579,13 | 32913,11 | 34627,88 | 19901,06 | 22007,56 | 21081,35 | 24397,94 | 15663,97 | 149,18 | 30776,64 | 30095 | 22950,62 |
| **PIK3CA_1047wt_** | AHHGGWTTK | 39036,15 | 34641,36 | 9840,68 | 29029,98 | 37188,28 | 30055,62 | 36595,54 | 10216,72 | 16893,14 | 33532,45 | 33219,72 | 27708,8 |
| **PIK3CA_1047wt_** | DAHHGGWTT | 36721,29 | 35690,68 | 41393,37 | 41333,38 | 24291,52 | 30156,28 | 19812,75 | 39340,16 | 19686,25 | 38872,18 | 33844,1 | 36990,85 |
| **PIK3CA_1047wt_** | NDAHHGGWT | 38591,43 | 44249,11 | 43940,43 | 45716,38 | 40421 | 39065,29 | 36085,57 | 41773,54 | 40924,43 | 41070,82 | 44883,68 | 43013,28 |
| **PIK3CA_1047wt_** | MNDAHHGGW | 11488,48 | 37665,31 | 34417,21 | 21048,29 | 25759,21 | 26008,75 | 21289,7 | 25679,34 | 24009,83 | 29803,04 | 932,03 | 23911,32 |
| **PIK3CA_1047wt_** | QMNDAHHGG | 34867,36 | 22488,74 | 33917,04 | 34948,2 | 38955,98 | 34800,28 | 25375,52 | 33473,35 | 30397,04 | 35528,85 | 22557,96 | 9093,22 |
| **PIK3CA_1047wt_** | KQMNDAHHG | 41190,08 | 22333,79 | 34867,36 | 28761,42 | 41807,01 | 36854,22 | 28490,41 | 12899,41 | 27766,11 | 19154,25 | 15326,84 | 4296,89 |
| **PIK3CA_1047wt_** | MKQMNDAHH | 41307,02 | 44262,04 | 39698,06 | 45492,86 | 41957 | 38708,1 | 29308,98 | 25789,33 | 29746,02 | 36233,07 | 36490,36 | 15001,33 |
| **PIK3CA_1047wt_** | FMKQMNDAH | 25480,9 | 23347,11 | 18678,43 | 39240,68 | 22569,18 | 19605,06 | 6249,72 | 19414,23 | 20286,29 | 30118,12 | 31352,38 | 331,41 |
|  |  |  |  |  |  |  |  |  |  |  |  |  |  |
| **PIK3CA_H1047R_** | RHGGWTTKM | 32518,42 | 28747,12 | 30363,19 | 11223,83 | 30720,09 | 17034,83 | 26129,74 | 9794,79 | 951,43 | 27030,98 | 23839,77 | 17940,39 |
| **PIK3CA_H1047R_** | ARHGGWTTK | 42006,05 | 35100,91 | 12220,23 | 37189,5 | 38953,46 | 31653,36 | 31900,22 | 152,47 | 27018,99 | 33535,34 | 35531,55 | 30924,17 |
| **PIK3CA_H1047R_** | DARHGGWTT | 38214,56 | 38920,6 | 40480,97 | 42653,18 | 25698,81 | 21184,69 | 11176,8 | 39530,46 | 30437,2 | 40760,04 | 36116,05 | 34928,15 |
| **PIK3CA_H1047R_** | NDARHGGWT | 38483,02 | 44321,45 | 43971,81 | 45558,86 | 41344,57 | 38721,91 | 32663,01 | 40282,61 | 40205,96 | 40507,69 | 44705,32 | 43180,68 |
| **PIK3CA_H1047R_** | MNDARHGGW | 15539,4 | 39456,12 | 36121,12 | 21810,57 | 29160,94 | 27519,37 | 17456,89 | 26274,9 | 28575,31 | 35368,91 | 1697,46 | 27736,69 |
| **PIK3CA_H1047R_** | QMNDARHGG | 37989,45 | 27321,49 | 33386,55 | 38533,43 | 40365,93 | 29824,33 | 22857,95 | 33095,95 | 33153,29 | 37764,48 | 26388,01 | 11496,06 |
| **PIK3CA_H1047R_** | KQMNDARHG | 43721,33 | 31476,1 | 35173,52 | 37136,01 | 42653,65 | 36227,58 | 29389,32 | 13644,64 | 35323,03 | 24351 | 20370,09 | 4815,42 |
| **PIK3CA_H1047R_** | MKQMNDARH | 40669,3 | 44570,08 | 39199,1 | 45742,13 | 42105,24 | 39549,72 | 30538,48 | 24168,82 | 30367,14 | 36757,85 | 37262,39 | 20484,81 |
| **PIK3CA_H1047R_** | FMKQMNDAR | 29756,64 | 16651,06 | 12433,9 | 34134,25 | 23226,17 | 24628,95 | 7027,3 | 12167,99 | 24715,17 | 33245,24 | 35808,27 | 10240,08 |
|  |  |  |  |  |  |  |  |  |  |  |  |  |  |
| **PIK3CA_H1047L_** | LHGGWTTKM | 30200,69 | 31195,38 | 35670,99 | 18069,55 | 27849,47 | 24170,66 | 25671,84 | 20647,02 | 704,18 | 32992,61 | 29635,51 | 21372,79 |
| **PIK3CA_H1047L_** | ALHGGWTTK | 32357,68 | 15050,91 | 35,26 | 32319,89 | 32272,73 | 25572,04 | 30715,77 | 12768,32 | 37186,67 | 34940,63 | 29501,46 | 12316,61 |
| **PIK3CA_H1047L_** | DALHGGWTT | 35967,85 | 26376,3 | 40219,45 | 39319,31 | 29754,07 | 32107,28 | 21675,53 | 37616,04 | 21048,29 | 38257,17 | 28487,32 | 37247,49 |
| **PIK3CA_H1047L_** | NDALHGGWT | 37400,94 | 43489,22 | 43848,77 | 44769,69 | 41348,6 | 38320,13 | 33359,11 | 40850,99 | 38869,68 | 38257,98 | 44186,43 | 42460,27 |
| **PIK3CA_H1047L_** | MNDALHGGW | 12052,16 | 34482,06 | 34545,92 | 17485,82 | 26443,17 | 28261,08 | 26011,28 | 22872,78 | 23804,45 | 28157,6 | 399,61 | 22538,68 |
| **PIK3CA_H1047L_** | QMNDALHGG | 34459,68 | 11464,26 | 32962,65 | 36491,96 | 34101,4 | 36860,61 | 29615,32 | 32461,47 | 32963,71 | 36250,32 | 21445,76 | 7709,15 |
| **PIK3CA_H1047L_** | KQMNDALHG | 38807,91 | 17682,54 | 27935,16 | 28458,99 | 41320,88 | 34977,69 | 28836,82 | 8632,27 | 26364,89 | 15045,54 | 10507,76 | 2213,38 |
| **PIK3CA_H1047L_** | MKQMNDALH | 36215,83 | 41792,53 | 34506,69 | 43322,02 | 39415,58 | 37010,46 | 26835,16 | 18522,06 | 23554,41 | 30909,12 | 32510,33 | 13608,52 |
| **PIK3CA_H1047L_** | FMKQMNDAL | 22962,79 | 89,17 | 31815,76 | 13261,7 | 22130,54 | 2713,52 | 40,19 | 12360,79 | 285,55 | 10406,38 | 20578,56 | 102,31 |
|  |  |  |  |  |  |  |  |  |  |  |  |  |  |
|  |  |  |  |  |  |  |  |  |  |  |  |  |  |
| **PROTEIN** | **PEPTIDE** | **A*01:01** | **A*02:01** | **A*03:01** | **A*24:02** | **A*26:02** | **B*07:02** | **B*08:02** | **B*27:05** | **B*39:01** | **B*40:01** | **B*58:01** | **B*15:01** |
| **PIK3CA_542wt_** | EITEQEKDF | 38611,46 | 44521,88 | 44148,69 | 42960,28 | 13721,62 | 38948,39 | 33912,27 | 43790,92 | 43073,82 | 45337,6 | 40328,38 | 30477,74 |
| **PIK3CA_542wt_** | SEITEQEKD | 45994,22 | 46495,58 | 47038,52 | 46361,47 | 46475,97 | 46397,09 | 44110,5 | 43254,57 | 44147,72 | 25528,63 | 41462,84 | 41943,39 |
| **PIK3CA_542wt_** | LSEITEQEK | 9749,42 | 40259,93 | 15562,62 | 44863,28 | 43495,33 | 42029,25 | 42825,68 | 38548,84 | 43926,66 | 43521,68 | 20559,64 | 37190,7 |
| **PIK3CA_542wt_** | PLSEITEQE | 39979,52 | 27966,02 | 40291,31 | 43355,78 | 43888,63 | 44970,2 | 43486,86 | 44206,53 | 45279,25 | 45233,72 | 38928,18 | 38367,02 |
| **PIK3CA_542wt_** | DPLSEITEQ | 46304,31 | 47093,52 | 44017,52 | 48227,96 | 40825,81 | 34985,27 | 37766,52 | 44904,06 | 41936,57 | 46514,71 | 45094,44 | 43284,99 |
| **PIK3CA_542wt_** | RDPLSEITE | 44086,16 | 44344,01 | 45000,37 | 44491,54 | 47044,65 | 45036,92 | 44129,59 | 44297,96 | 45559,36 | 41421,14 | 43531,11 | 43306,54 |
| **PIK3CA_542wt_** | TRDPLSEIT | 37644,94 | 42073,37 | 44683,07 | 42958,39 | 45869,98 | 39445,43 | 38020,31 | 24625,22 | 4985,56 | 31730,85 | 41121,95 | 43666,5 |
| **PIK3CA_542wt_** | STRDPLSEI | 24498,99 | 14191,05 | 16362,63 | 24844,94 | 3599,73 | 1051,9 | 12510,41 | 28496,58 | 29125,62 | 28986,36 | 8976,11 | 5995,9 |
| **PIK3CA_542wt_** | ISTRDPLSE | 32422,87 | 40018,91 | 35642,06 | 45000,37 | 45533,25 | 40794,47 | 38984,23 | 40457,33 | 41699,95 | 45038,87 | 8749,24 | 36132,85 |
|  |  |  |  |  |  |  |  |  |  |  |  |  |  |
| **PIK3CA_E542K_** | KITEQEKDF | 40290,45 | 35764,9 | 36560,72 | 37171,4 | 38176,95 | 33772,39 | 37448,32 | 38454,29 | 44761,46 | 41244,5 | 17982,95 | 13850,05 |
| **PIK3CA_E542K_** | SKITEQEKD | 47536,86 | 47655,31 | 47688,82 | 47487 | 47728,08 | 46536,86 | 44441,98 | 42376,26 | 43242,88 | 43211,08 | 40907,62 | 44948,3 |
| **PIK3CA_E542K_** | LSKITEQEK | 29853,39 | 40934,62 | 6448,58 | 43762,52 | 41684,59 | 38500,49 | 35662,11 | 34560,88 | 44963,38 | 44180,22 | 19362,63 | 26710,88 |
| **PIK3CA_E542K_** | PLSKITEQE | 40366,37 | 33617,45 | 38868 | 42772,9 | 44735,81 | 44987,71 | 42563,74 | 43800,89 | 45912,69 | 45939,52 | 38522,6 | 38420,6 |
| **PIK3CA_E542K_** | DPLSKITEQ | 45877,44 | 46826,77 | 42320,82 | 47774,06 | 39843,48 | 29920,96 | 20244,64 | 43400,82 | 40177,27 | 46172,24 | 45584,02 | 43758,71 |
| **PIK3CA_E542K_** | RDPLSKITE | 44161,11 | 45013,02 | 44076,15 | 44396,81 | 46783,73 | 43272,82 | 42165,89 | 43548,07 | 45383,24 | 41353,08 | 43933,29 | 43484,51 |
| **PIK3CA_E542K_** | TRDPLSKIT | 40672,81 | 43869,16 | 43954,68 | 44586,96 | 46049,51 | 38239,78 | 35890,11 | 21475,95 | 9445,3 | 35637,81 | 43301,85 | 44474,21 |
| **PIK3CA_E542K_** | STRDPLSKI | 27964,51 | 21213,36 | 16676,67 | 24761,21 | 5175,85 | 3057,27 | 14993,7 | 28116,81 | 35091,41 | 32717,12 | 9642,52 | 11134,68 |
| **PIK3CA_E542K_** | ISTRDPLSK | 18822,46 | 39578,4 | 1443,75 | 42416,64 | 42031,07 | 39246,2 | 40490,61 | 29024,01 | 43236,78 | 44230,91 | 9857,3 | 31971,73 |
|  |  |  |  |  |  |  |  |  |  |  |  |  |  |
|  |  |  |  |  |  |  |  |  |  |  |  |  |  |
| **PROTEIN** | **PEPTIDE** | **A*01:01** | **A*02:01** | **A*03:01** | **A*24:02** | **A*26:02** | **B*07:02** | **B*08:02** | **B*27:05** | **B*39:01** | **B*40:01** | **B*58:01** | **B*15:01** |
| **PIK3CA_88wt_** | RLCDLRLFQ | 28143,89 | 12533,17 | 1767,94 | 35670,99 | 39564,26 | 33055,14 | 27909,79 | 12500,13 | 38882,29 | 37706,48 | 21360,54 | 13525,43 |
| **PIK3CA_88wt_** | RRLCDLRLF | 34839,46 | 28033,88 | 29701,64 | 5600,69 | 29964,7 | 24519,41 | 17337,37 | 19,07 | 15073,41 | 21176,21 | 11340,28 | 12012,84 |
| **PIK3CA_88wt_** | TRRLCDLRL | 35311,18 | 36370,54 | 35117,62 | 31732,56 | 39325,27 | 15173,88 | 5458,07 | 261,02 | 6500,78 | 29752,46 | 35200,18 | 26552,97 |
| **PIK3CA_88wt_** | ETRRLCDLR | 25216,78 | 40620,91 | 19165,66 | 40222,07 | 16299,72 | 38258,82 | 30526,58 | 28290,75 | 42310,75 | 43308,41 | 35181,14 | 37264,82 |
| **PIK3CA_88wt_** | DETRRLCDL | 36313,14 | 42236,65 | 43619,27 | 39440,75 | 34714,16 | 32964,07 | 6494,94 | 34915,31 | 15985,69 | 12756,03 | 42372,6 | 33070,54 |
| **PIK3CA_88wt_** | FDETRRLCD | 39460,8 | 46077,91 | 46057,47 | 45832,28 | 47160,32 | 44717,89 | 31291,37 | 42344,19 | 43219,49 | 45637,32 | 44372,79 | 45961,9 |
| **PIK3CA_88wt_** | FFDETRRLC | 24392,92 | 14317,83 | 37147,68 | 24202,59 | 39933,26 | 24098,07 | 9918,17 | 30674,91 | 18809,24 | 35780,77 | 36361,89 | 37194,73 |
| **PIK3CA_88wt_** | EFFDETRRL | 37658,78 | 24550,2 | 36922,07 | 11202,72 | 12146,81 | 33075,89 | 16944,03 | 30859,66 | 17704,94 | 29285,21 | 34360,29 | 25282,63 |
| **PIK3CA_88wt_** | EEFFDETRR | 41141,53 | 43799,45 | 36522,36 | 46267,74 | 34845,48 | 43357,66 | 43510,85 | 35963,18 | 40063,09 | 31778,6 | 41199,43 | 41522,54 |
|  |  |  |  |  |  |  |  |  |  |  |  |  |  |
| **PIK3CA_R88Q_** | QLCDLRLFQ | 28520,95 | 17157,66 | 8428,86 | 40320,97 | 34780,71 | 38998,99 | 31437,98 | 28139,32 | 39540,71 | 40638,52 | 29238,35 | 20304,53 |
| **PIK3CA_R88Q_** | RQLCDLRLF | 29147,7 | 6147,05 | 17440,66 | 495,74 | 19641,57 | 23491,8 | 17878,76 | 398,81 | 13721,18 | 3180,6 | 1660,39 | 47,05 |
| **PIK3CA_R88Q_** | TRQLCDLRL | 33337,09 | 31419,62 | 37877,41 | 24571,19 | 39867,21 | 26889,5 | 13203,99 | 354,43 | 1053,53 | 24244,25 | 32897,09 | 31953,41 |
| **PIK3CA_R88Q_** | ETRQLCDLR | 26775,41 | 40559,45 | 22184,71 | 41677,39 | 13994,35 | 38974,12 | 32849,07 | 31849,16 | 42512,66 | 43131,2 | 36697,05 | 37286,19 |
| **PIK3CA_R88Q_** | DETRQLCDL | 36225,24 | 42215,66 | 44252,45 | 40693,07 | 36295,46 | 35895,94 | 20517,41 | 36778,55 | 17746,18 | 8318,68 | 42236,65 | 32978,34 |
| **PIK3CA_R88Q_** | FDETRQLCD | 37713,44 | 45690,18 | 46366,46 | 45673,39 | 47311,61 | 46045,03 | 37531,47 | 43665,54 | 43279,38 | 45780,23 | 43289,2 | 45871,96 |
| **PIK3CA_R88Q_** | FFDETRQLC | 17663,42 | 8899,43 | 37165,77 | 21650 | 39239,84 | 24967,28 | 10589,82 | 31493,48 | 15177,49 | 33415,83 | 34247,81 | 37107,5 |
| **PIK3CA_R88Q_** | EFFDETRQL | 38326,36 | 21046,24 | 36936,05 | 12070,04 | 11696,68 | 30362,21 | 14677,33 | 31298,5 | 12420,18 | 26089,35 | 33081,27 | 22396,47 |
| **PIK3CA_R88Q_** | EEFFDETRQ | 44545,02 | 46208,22 | 45152,05 | 47996,84 | 42567,45 | 45147,66 | 44742,59 | 42449,7 | 42097,07 | 31681,79 | 43146,58 | 39963,1 |
|  |  |  |  |  |  |  |  |  |  |  |  |  |  |
|  |  |  |  |  |  |  |  |  |  |  |  |  |  |
| **PROTEIN** | **PEPTIDE** | **A*01:01** | **A*02:01** | **A*03:01** | **A*24:02** | **A*26:02** | **B*07:02** | **B*08:02** | **B*27:05** | **B*39:01** | **B*40:01** | **B*58:01** | **B*15:01** |
| **PIK3CA_345wt_** | NVNIRDIDK | 31330,35 | 40013,72 | 8982,72 | 44746,46 | 37285,78 | 39355,92 | 32580,05 | 38044,59 | 44636,21 | 45267,02 | 37108,31 | 36918,88 |
| **PIK3CA_345wt_** | VNVNIRDID | 43311,71 | 44187,86 | 43939,46 | 43845,91 | 46537,88 | 44082,8 | 39590,39 | 39458,66 | 42464,41 | 43568,8 | 31460,78 | 41613,39 |
| **PIK3CA_345wt_** | YVNVNIRDI | 24725,6 | 7930,13 | 31437,98 | 21807,98 | 15092 | 18577,04 | 8317,6 | 28634,42 | 21481,99 | 31372,74 | 13759,83 | 9935,68 |
| **PIK3CA_345wt_** | TYVNVNIRD | 43406 | 42404,26 | 40604,23 | 17619,52 | 43745 | 43906,69 | 41306,57 | 37642,1 | 41494,7 | 44676,79 | 33583,65 | 41739,2 |
| **PIK3CA_345wt_** | ATYVNVNIR | 26940,76 | 29667,6 | 1113,85 | 38431,41 | 22480,96 | 36951,65 | 34701,02 | 24661,48 | 40168,12 | 37514,02 | 22251,07 | 25593,63 |
| **PIK3CA_345wt_** | CATYVNVNI | 23449,39 | 7706,32 | 29428,77 | 22271,54 | 26207,9 | 24050,65 | 21271,97 | 26554,7 | 17423,5 | 27223,22 | 861,3 | 17452,55 |
| **PIK3CA_345wt_** | LCATYVNVN | 39678,73 | 39849,52 | 42348,78 | 44772,12 | 45840,23 | 41449,82 | 35672,14 | 41759,53 | 42918,91 | 43893,87 | 19235,88 | 32410,93 |
| **PIK3CA_345wt_** | ILCATYVNV | 25354,95 | 36,09 | 12666,91 | 17502,66 | 33496,18 | 35158,3 | 19488,95 | 29229,17 | 28996,4 | 32642,52 | 17391,47 | 7209,36 |
| **PIK3CA_345wt_** | KILCATYVN | 36722,88 | 20845,23 | 16792,73 | 29390,9 | 41961,54 | 32509,64 | 31764,51 | 29288,68 | 38567,61 | 39202,08 | 5395,25 | 18471,02 |
|  |  |  |  |  |  |  |  |  |  |  |  |  |  |
| **PIK3CA_N345K_** | KVNIRDIDK | 36074,27 | 38656,61 | 478,35 | 43911,9 | 43929,97 | 37141,64 | 36888,93 | 29402,04 | 46609,42 | 44622,68 | 30110,32 | 32812,48 |
| **PIK3CA_N345K_** | VKVNIRDID | 46938,88 | 46330,38 | 46146,75 | 46622,02 | 47330,04 | 44266,84 | 41589,08 | 35490,45 | 39301,87 | 40854,1 | 38977,06 | 41503,69 |
| **PIK3CA_N345K_** | YVKVNIRDI | 34870,75 | 18417,74 | 34993,21 | 24344,94 | 15162,06 | 14964,69 | 1355,92 | 29556,42 | 31262,95 | 35488,13 | 25635,21 | 8843,18 |
| **PIK3CA_N345K_** | TYVKVNIRD | 44046,57 | 43311,23 | 40960,76 | 17329,3 | 44254,85 | 44018,94 | 41110,39 | 38653,7 | 41986,53 | 45328,28 | 34116,89 | 41789,81 |
| **PIK3CA_N345K_** | ATYVKVNIR | 29348,02 | 31008,94 | 681,93 | 38376,57 | 23764,05 | 36898,12 | 26501,88 | 22385,08 | 40592,81 | 39314,62 | 27567,95 | 26736,04 |
| **PIK3CA_N345K_** | CATYVKVNI | 29248,48 | 16029,34 | 30848,97 | 25258,02 | 31629,39 | 22568,94 | 17482,03 | 28164,3 | 22282,86 | 32094,09 | 2003,82 | 23225,17 |
| **PIK3CA_N345K_** | LCATYVKVN | 43099,47 | 43175,09 | 43776,24 | 46635,64 | 46979,53 | 42991,43 | 36325,32 | 42272,79 | 45376,36 | 45489,43 | 25682,95 | 37039,71 |
| **PIK3CA_N345K_** | ILCATYVKV | 25665,45 | 80,57 | 15146,48 | 17958,45 | 34968,62 | 36385,11 | 20524,52 | 29801,43 | 30593,71 | 33246,31 | 19173,12 | 9870,21 |
| **PIK3CA_N345K_** | KILCATYVK | 32807,87 | 18294,8 | 39,61 | 24155,23 | 38363,28 | 28598,5 | 28996,4 | 11823,16 | 37563,96 | 37830,32 | 14049,58 | 20325,62 |
|  |  |  |  |  |  |  |  |  |  |  |  |  |  |
|  |  |  |  |  |  |  |  |  |  |  |  |  |  |
| **PROTEIN** | **PEPTIDE** | **A*01:01** | **A*02:01** | **A*03:01** | **A*24:02** | **A*26:02** | **B*07:02** | **B*08:02** | **B*27:05** | **B*39:01** | **B*40:01** | **B*58:01** | **B*15:01** |
| **PIK3CA_420wt_** | CPLAWGNIN | 37257,15 | 40179,01 | 37462,09 | 40801,52 | 41776,25 | 14147,67 | 27646,5 | 30345,45 | 33381,86 | 41932,49 | 28944,68 | 36702,61 |
| **PIK3CA_420wt_** | HCPLAWGNI | 34712,28 | 27749,61 | 38847,4 | 21141,65 | 35201,7 | 29929,4 | 28325,96 | 38471,36 | 33879,26 | 37510,78 | 28769,21 | 32265,38 |
| **PIK3CA_420wt_** | EHCPLAWGN | 41783,05 | 44629,91 | 42827,52 | 38970,32 | 43395,2 | 44944,91 | 40866,46 | 32600,86 | 23432,64 | 43690,58 | 36271,9 | 42124,39 |
| **PIK3CA_420wt_** | EEHCPLAWG | 42395,54 | 42672,11 | 43137,26 | 43751,62 | 36880,94 | 39427,1 | 34069,68 | 34672,12 | 34450,37 | 23151,89 | 39868,08 | 30683,87 |
| **PIK3CA_420wt_** | KEEHCPLAW | 31330,35 | 40960,76 | 37918,42 | 26552,69 | 41452,97 | 33863,51 | 38373,65 | 27523,84 | 29607,62 | 6287,84 | 7181,57 | 26142,18 |
| **PIK3CA_420wt_** | AKEEHCPLA | 38970,32 | 38388,6 | 42220,66 | 43442,18 | 43721,81 | 38956,41 | 28587,37 | 27302,87 | 21745,78 | 22662,42 | 42272,32 | 37064,57 |
| **PIK3CA_420wt_** | GAKEEHCPL | 35238,66 | 24771,93 | 37291,04 | 36943,64 | 30538,48 | 3815,12 | 1533,71 | 29999,41 | 10547,29 | 18852,02 | 20720,65 | 3119,35 |
| **PIK3CA_420wt_** | KGAKEEHCP | 45571,21 | 44420,84 | 45622,52 | 44941,49 | 48414,1 | 45261,62 | 42676,74 | 44805,57 | 45596,34 | 45401,89 | 29771,14 | 43496,73 |
| **PIK3CA_420wt_** | RKGAKEEHC | 47584,21 | 45902,74 | 46449,34 | 46079,9 | 48314,66 | 44931,79 | 33665,51 | 32757,15 | 40875,76 | 42734,49 | 41255,64 | 44064,21 |
|  |  |  |  |  |  |  |  |  |  |  |  |  |  |
| **PIK3CA_C420R_** | RPLAWGNIN | 39245,79 | 38868,42 | 31895,39 | 39196,97 | 43493,45 | 2837,4 | 26985,4 | 24528,15 | 32225,62 | 38292,79 | 24424,35 | 33925,85 |
| **PIK3CA_C420R_** | HRPLAWGNI | 36795,65 | 35818,34 | 37412,69 | 17039,07 | 32098,96 | 28392,24 | 23740,15 | 3740,69 | 10083,08 | 31776,21 | 37649,43 | 30541,79 |
| **PIK3CA_C420R_** | EHRPLAWGN | 43802,3 | 45771,82 | 42527,39 | 39979,96 | 43281,72 | 41817,88 | 35482 | 32270,63 | 27258,89 | 44454,02 | 38963,57 | 40103 |
| **PIK3CA_C420R_** | EEHRPLAWG | 43234,46 | 44587,93 | 43795,18 | 44427,57 | 37314,45 | 39577,96 | 32504,71 | 33728,2 | 36835,49 | 23201,05 | 40596,32 | 33316,18 |
| **PIK3CA_C420R_** | KEEHRPLAW | 32496,62 | 43094,33 | 38359,97 | 27320,61 | 41636,82 | 33324,84 | 30488,3 | 28527,12 | 30965,7 | 10654,87 | 11495,69 | 27772,43 |
| **PIK3CA_C420R_** | AKEEHRPLA | 40114,72 | 42376,26 | 41030,85 | 44642,49 | 44387,21 | 32400,78 | 23864,29 | 26241,1 | 22049,51 | 23729,62 | 42892,45 | 37295,06 |
| **PIK3CA_C420R_** | GAKEEHRPL | 39876,7 | 32352,44 | 38304,79 | 39346,12 | 31698,59 | 2816,6 | 2074,75 | 30193,17 | 18512,43 | 21704,17 | 26643,92 | 6233,99 |
| **PIK3CA_C420R_** | KGAKEEHRP | 45565,77 | 44024,19 | 45302,3 | 44363,69 | 48343,43 | 45735,7 | 43897,66 | 44754,68 | 45863,03 | 45650,66 | 28519,11 | 44283,11 |
| **PIK3CA_C420R_** | RKGAKEEHR | 46630,62 | 46305,32 | 39697,19 | 46310,82 | 47256,35 | 45815,91 | 40485,78 | 20634,73 | 45715,88 | 45789,15 | 44368,96 | 44319,05 |
|  |  |  |  |  |  |  |  |  |  |  |  |  |  |
|  |  |  |  |  |  |  |  |  |  |  |  |  |  |
| **PROTEIN** | **PEPTIDE** | **A*01:01** | **A*02:01** | **A*03:01** | **A*24:02** | **A*26:02** | **B*07:02** | **B*08:02** | **B*27:05** | **B*39:01** | **B*40:01** | **B*58:01** | **B*15:01** |
| **PIK3CA_726wt_** | ETQKVQMKF | 9158,29 | 38289,06 | 30151,71 | 8364,17 | 374,18 | 27899,23 | 20483,48 | 28553,69 | 32705,1 | 34535,07 | 1757,64 | 10158,52 |
| **PIK3CA_726wt_** | DETQKVQMK | 42293,38 | 46689,66 | 38723,58 | 46496,6 | 39102,09 | 44061,34 | 38640,73 | 39086,03 | 42326,77 | 39536,87 | 45462,36 | 42327,7 |
| **PIK3CA_726wt_** | KDETQKVQM | 36588,01 | 43882,94 | 41408,14 | 43791,4 | 44516,11 | 36783,72 | 34451,48 | 41310,59 | 40091,29 | 24672,43 | 38636,55 | 39813,33 |
| **PIK3CA_726wt_** | KKDETQKVQ | 45938,52 | 46374 | 42988,16 | 48393,15 | 47755,95 | 44381,43 | 43611,73 | 39524,91 | 40455,14 | 39303,13 | 43183,5 | 43090,61 |
| **PIK3CA_726wt_** | EKKDETQKV | 44883,68 | 45063,24 | 46237,23 | 45669,91 | 35540,4 | 41342,34 | 23130,61 | 39978,23 | 26171,06 | 41086,83 | 45234,21 | 37571,28 |
| **PIK3CA_726wt_** | QEKKDETQK | 44659,88 | 47106,77 | 38442,24 | 47625,94 | 43628,24 | 45072,98 | 43658,47 | 40015,01 | 46468,93 | 36231,5 | 44656,97 | 38494,66 |
| **PIK3CA_726wt_** | KQEKKDETQ | 43988,49 | 44604,33 | 42888,28 | 46258,73 | 46874,93 | 44476,16 | 39894,83 | 37100,68 | 42054,25 | 37584,71 | 39938,88 | 32045,16 |
| **PIK3CA_726wt_** | LKQEKKDET | 46082,4 | 46429,72 | 47220,07 | 47433,59 | 48026,44 | 43322,02 | 27344,85 | 40017,18 | 35113,82 | 43271,42 | 46163,25 | 45175,01 |
| **PIK3CA_726wt_** | ILKQEKKDE | 46918,06 | 43387,22 | 42094,79 | 48048,28 | 47460,28 | 43772,93 | 31003,24 | 45465,79 | 47854,23 | 47426,41 | 46287,77 | 36973,65 |
|  |  |  |  |  |  |  |  |  |  |  |  |  |  |
| **PIK3CA_E726K_** | KTQKVQMKF | 12879,61 | 25829,26 | 9939,87 | 953,74 | 18473,02 | 15638,41 | 25134,8 | 13142,42 | 36210,34 | 28957,83 | 15,89 | 1378,42 |
| **PIK3CA_E726K_** | DKTQKVQMK | 45565,27 | 47144,51 | 38352,89 | 46974,95 | 42979,79 | 45734,7 | 35652,47 | 36093,77 | 42408,85 | 46085,4 | 46063,95 | 44283,6 |
| **PIK3CA_E726K_** | KDKTQKVQM | 40242,07 | 44286,48 | 39787,07 | 42530,62 | 41471,82 | 30788,97 | 19941,58 | 38130,29 | 42388,21 | 29449,49 | 37134,4 | 31119,53 |
| **PIK3CA_E726K_** | KKDKTQKVQ | 46190,73 | 47146,55 | 42031,07 | 48380,05 | 47943,38 | 44325,29 | 42954,68 | 37658,78 | 42169,07 | 41365,59 | 43255,05 | 43309,36 |
| **PIK3CA_E726K_** | EKKDKTQKV | 44814,29 | 45347,89 | 46127,3 | 45296,89 | 34961,82 | 39322,71 | 6264,08 | 38511,74 | 28027,21 | 41862,23 | 46171,22 | 39451,85 |
| **PIK3CA_E726K_** | QEKKDKTQK | 45176 | 47448,49 | 37674,28 | 47612,03 | 43755,89 | 44302,28 | 41591,79 | 38834,77 | 46385,54 | 37684,07 | 45450,55 | 39043,76 |
| **PIK3CA_E726K_** | KQEKKDKTQ | 45918,17 | 45612,63 | 41792,99 | 47568,26 | 47305,96 | 44042,76 | 38666,66 | 36750,29 | 44618,83 | 39822,8 | 42796,49 | 32181,72 |
| **PIK3CA_E726K_** | LKQEKKDKT | 46676,53 | 47268,63 | 47431,02 | 47691,4 | 48208,66 | 45178,45 | 31288,33 | 39750,06 | 40113,84 | 45062,25 | 46486,02 | 46347,42 |
| **PIK3CA_E726K_** | ILKQEKKDK | 44482,87 | 42410,22 | 7194,87 | 46728,1 | 46017,62 | 42405,62 | 35209,7 | 39947,95 | 47916,39 | 46960,71 | 44668,58 | 33834,56 |
|  |  |  |  |  |  |  |  |  |  |  |  |  |  |
|  |  |  |  |  |  |  |  |  |  |  |  |  |  |
| **PROTEIN** | **PEPTIDE** | **A*01:01** | **A*02:01** | **A*03:01** | **A*24:02** | **A*26:02** | **B*07:02** | **B*08:02** | **B*27:05** | **B*39:01** | **B*40:01** | **B*58:01** | **B*15:01** |
| **TP53_157wt_** | VRAMAIYKQ | 43626,84 | 41286,46 | 37095,05 | 40599,83 | 43222,28 | 36759,03 | 37816 | 2626,03 | 29000,79 | 41110,39 | 34697,64 | 38299,82 |
| **TP53_157wt_** | RVRAMAIYK | 28640 | 23964,17 | 7,61 | 25419,76 | 33270,45 | 39725,13 | 21645,3 | 3539,52 | 41726,12 | 36241,29 | 16983,49 | 12469,19 |
| **TP53_157wt_** | TRVRAMAIY | 24818,61 | 40917,36 | 23367,58 | 36074,64 | 7799,09 | 11084,08 | 33116,72 | 509,54 | 13330,46 | 32200,86 | 24354,69 | 5678,3 |
| **TP53_157wt_** | GTRVRAMAI | 24763,36 | 25613,3 | 18828,58 | 30833,3 | 27171,43 | 22630,07 | 293,87 | 21206,02 | 31367,99 | 30527,57 | 17358,95 | 7303,73 |
| **TP53_157wt_** | PGTRVRAMA | 41452,52 | 42268,67 | 42034,23 | 42893,84 | 46656,85 | 46397,09 | 32546,58 | 39116,89 | 42466,24 | 45980,31 | 36532,65 | 42788,16 |
| **TP53_157wt_** | PPGTRVRAM | 43343,59 | 44139,12 | 42543,96 | 43484,51 | 41472,25 | 40526,11 | 2582,58 | 38888,18 | 33478,79 | 44017,52 | 43727,48 | 37362,11 |
| **TP53_157wt_** | PPPGTRVRA | 45309,63 | 44417 | 43588,62 | 45928,59 | 45191,15 | 44952,19 | 35565,39 | 43723,7 | 39538,17 | 45921,13 | 45984,28 | 45222,96 |
| **TP53_157wt_** | TPPPGTRVR | 42434,07 | 44699,04 | 32483,61 | 44807,5 | 31549,06 | 28314,33 | 31540,88 | 40773,71 | 38461,78 | 44038,96 | 44224,69 | 41400,53 |
| **TP53_157wt_** | STPPPGTRV | 20378,7 | 6637,16 | 27080,74 | 19426,42 | 9095,38 | 2715,6 | 32365,04 | 32937,34 | 29145,8 | 32773,8 | 23477,3 | 20741,73 |
|  |  |  |  |  |  |  |  |  |  |  |  |  |  |
| **TP53_V157F_** | FRAMAIYKQ | 40431,5 | 37110,7 | 38721,91 | 38761,73 | 42329,07 | 40145,98 | 28880,22 | 1005,21 | 14125,03 | 38566,79 | 33162,27 | 34137,58 |
| **TP53_V157F_** | RFRAMAIYK | 34475,73 | 31180,2 | 101,99 | 13616,62 | 12084,8 | 25997,21 | 22064,54 | 6464,16 | 42563,74 | 39352,08 | 28631,33 | 24135,38 |
| **TP53_V157F_** | TRFRAMAIY | 23264,65 | 38141,43 | 15745,03 | 31789,61 | 35927,02 | 35329,13 | 30394,42 | 164,64 | 12260,76 | 28924,64 | 22177,27 | 4604,43 |
| **TP53_V157F_** | GTRFRAMAI | 21718,27 | 22501,16 | 14781,88 | 28154,86 | 1143,56 | 866,79 | 322,25 | 19558,02 | 28803,46 | 28457,45 | 16653,23 | 6210,63 |
| **TP53_V157F_** | PGTRFRAMA | 39934,99 | 41130,41 | 40959,86 | 41927,95 | 37947,14 | 38807,48 | 31184,9 | 37877,02 | 41827,37 | 45703,53 | 36259,34 | 42396 |
| **TP53_V157F_** | PPGTRFRAM | 42377,19 | 43247,09 | 40644,23 | 39974,34 | 1093,74 | 667,67 | 743,83 | 35939,07 | 29858,24 | 42863,7 | 43356,26 | 34830,79 |
| **TP53_V157F_** | PPPGTRFRA | 44046,11 | 42774,28 | 42699,37 | 43226,49 | 18641,88 | 19283,18 | 32548,34 | 41560,31 | 37394,48 | 45431,38 | 44473,75 | 44807,01 |
| **TP53_V157F_** | TPPPGTRFR | 39976,94 | 43569,73 | 26692,97 | 42016,97 | 17937,48 | 23090,1 | 33431 | 39096,16 | 41208,82 | 44092,36 | 43224,62 | 42098,42 |
| **TP53_V157F_** | STPPPGTRF | 13768,47 | 26734 | 26883,1 | 3803,37 | 17357,26 | 12525,72 | 32514,92 | 29593,86 | 29632,95 | 29242,14 | 5736,41 | 2198,39 |
|  |  |  |  |  |  |  |  |  |  |  |  |  |  |
|  |  |  |  |  |  |  |  |  |  |  |  |  |  |
| **PROTEIN** | **PEPTIDE** | **A*01:01** | **A*02:01** | **A*03:01** | **A*24:02** | **A*26:02** | **B*07:02** | **B*08:02** | **B*27:05** | **B*39:01** | **B*40:01** | **B*58:01** | **B*15:01** |
| **TP53_282wt_** | RRTEEENLR | 43776,71 | 40801,52 | 36626,04 | 42644,88 | 45149,11 | 43598,04 | 42629,2 | 1017,89 | 36913,68 | 41680,53 | 39560,84 | 43285,94 |
| **TP53_282wt_** | DRRTEEENL | 44316,65 | 45382,24 | 45694,13 | 42586,34 | 43127,92 | 37293,45 | 17080,61 | 24751,03 | 11122,04 | 41389,34 | 44428,54 | 40712,44 |
| **TP53_282wt_** | RDRRTEEEN | 44614 | 47492,64 | 45052,03 | 46734,15 | 46968,35 | 43974,19 | 41436,83 | 39787,92 | 47575,44 | 44409,78 | 39952,71 | 43705,26 |
| **TP53_282wt_** | GRDRRTEEE | 44050,4 | 46330,86 | 45397 | 46137,77 | 47375,11 | 43629,65 | 35965,13 | 23058,15 | 34804,41 | 42770,11 | 44749,34 | 46163,25 |
| **TP53_282wt_** | PGRDRRTEE | 47402,8 | 48230,58 | 44741,61 | 48011,88 | 48129,97 | 41946,55 | 32752,88 | 44891,93 | 47549,73 | 48443,43 | 46864,78 | 45615,61 |
| **TP53_282wt_** | CPGRDRRTE | 45332,69 | 46451,34 | 42698,43 | 46637,18 | 46210,22 | 8860,23 | 19275,26 | 38231,94 | 40514,27 | 46185,22 | 42381,32 | 43431,84 |
| **TP53_282wt_** | ACPGRDRRT | 47104,73 | 45460,39 | 46697,76 | 47435,12 | 47247,66 | 45004,75 | 42357,46 | 46099,85 | 47253,79 | 47866,67 | 45961,9 | 46623,55 |
| **TP53_282wt_** | CACPGRDRR | 30307,06 | 37631,49 | 23101,85 | 40396,53 | 35183,8 | 36472,62 | 37312,02 | 29877,31 | 40592,37 | 44250,54 | 27722,6 | 39101,26 |
| **TP53_282wt_** | VCACPGRDR | 41986,96 | 41932,49 | 30102,16 | 46290,78 | 44563,33 | 38969,04 | 42891,98 | 39694,6 | 45678,81 | 45493,86 | 40389,96 | 41041,94 |
|  |  |  |  |  |  |  |  |  |  |  |  |  |  |
| **TP53_R282W_** | WRTEEENLR | 40967,84 | 40499,8 | 41051,26 | 43074,77 | 42729,39 | 43972,29 | 40708,03 | 3545,04 | 28552,75 | 41568,84 | 41050,38 | 42933,77 |
| **TP53_R282W_** | DWRTEEENL | 43852,08 | 42500,24 | 44288,85 | 19974,4 | 41885,32 | 37426,86 | 16839,12 | 40868,67 | 38579,73 | 43781,91 | 44374,25 | 37624,16 |
| **TP53_R282W_** | RDWRTEEEN | 43031,9 | 45445,65 | 44990,15 | 44606,25 | 47373,58 | 45245,95 | 43171,36 | 35408,74 | 45859,57 | 38684,64 | 35606,2 | 43777,2 |
| **TP53_R282W_** | GRDWRTEEE | 42913,81 | 44929,35 | 44198,4 | 45322,37 | 46974,95 | 42606,61 | 37143,65 | 22575,77 | 29166 | 40707,61 | 43630,61 | 45576,11 |
| **TP53_R282W_** | PGRDWRTEE | 46397,09 | 47017,66 | 43208,26 | 47241,54 | 47540,47 | 42911,49 | 41009,98 | 44247,2 | 47370,5 | 47931,97 | 45443,19 | 44270,66 |
| **TP53_R282W_** | CPGRDWRTE | 42868,34 | 44184,04 | 41697,23 | 44607,72 | 45147,16 | 16035,76 | 26110,52 | 36813,17 | 39679,58 | 45305,71 | 37968,09 | 42154,04 |
| **TP53_R282W_** | ACPGRDWRT | 43074,3 | 37164,16 | 45302,3 | 43091,08 | 46111,83 | 44909,42 | 39524,04 | 44504,08 | 44956,1 | 45944 | 42405,62 | 45495,82 |
| **TP53_R282W_** | CACPGRDWR | 29351,18 | 34480,2 | 20795,66 | 38224,49 | 33671,33 | 34967,86 | 35173,16 | 26479,52 | 39157,99 | 43053,33 | 25541,63 | 37983,72 |
| **TP53_R282W_** | VCACPGRDW | 36776,15 | 40623,11 | 40032,33 | 32380,81 | 42106,62 | 33334,94 | 38554,29 | 38363,7 | 43192,38 | 41833,71 | 1234,74 | 29337,85 |
|  |  |  |  |  |  |  |  |  |  |  |  |  |  |
|  |  |  |  |  |  |  |  |  |  |  |  |  |  |
| **PROTEIN** | **PEPTIDE** | **A*01:01** | **A*02:01** | **A*03:01** | **A*24:02** | **A*26:02** | **B*07:02** | **B*08:02** | **B*27:05** | **B*39:01** | **B*40:01** | **B*58:01** | **B*15:01** |
| **TP53_273wt_** | RVCACPGRD | 39329,1 | 37038,11 | 32700,48 | 42812,7 | 44291,25 | 41244,04 | 41400,98 | 34533,58 | 46040,54 | 44760,02 | 20854,03 | 33113,86 |
| **TP53_273wt_** | VRVCACPGR | 44125,75 | 34321,64 | 25981,45 | 38943,34 | 40142,07 | 41283,77 | 36827,11 | 542,95 | 34962,56 | 41109,49 | 40005,05 | 38351,66 |
| **TP53_273wt_** | EVRVCACPG | 34144,95 | 37715,48 | 31646,85 | 41034,84 | 23520,78 | 12612,34 | 7790,4 | 32411,64 | 36778,93 | 41816,04 | 30162,15 | 15879,33 |
| **TP53_273wt_** | FEVRVCACP | 38050,75 | 29980,59 | 41587,28 | 39790,51 | 39567,27 | 35675,61 | 23476,29 | 31151,54 | 23677,3 | 4039,81 | 36214,26 | 23330,94 |
| **TP53_273wt_** | SFEVRVCAC | 36020,82 | 29885,71 | 37222,5 | 30126,59 | 43759,19 | 26822,96 | 3522,59 | 37872,91 | 24861,08 | 38775,59 | 42022,42 | 36164,13 |
| **TP53_273wt_** | NSFEVRVCA | 20696,01 | 8933,97 | 27677,63 | 36840,67 | 22066,94 | 19999,92 | 8928,65 | 26643,92 | 9107,11 | 28033,27 | 12734,1 | 19008,09 |
| **TP53_273wt_** | RNSFEVRVC | 40612,58 | 37686,5 | 37058,16 | 38759,64 | 45327,28 | 34703,64 | 30630,81 | 33185,95 | 31565,81 | 34683,75 | 18932,55 | 32137,17 |
| **TP53_273wt_** | GRNSFEVRV | 34883,21 | 24865,12 | 34723,18 | 35272,61 | 40646,86 | 39257,66 | 30663,64 | 355,46 | 11077,6 | 25180,52 | 33176,62 | 36406,77 |
| **TP53_273wt_** | LGRNSFEVR | 39476,61 | 37651,06 | 20165,07 | 38670,43 | 40537,06 | 31054,93 | 23193,27 | 24428,84 | 39765,11 | 41431 | 32254,57 | 23473,25 |
|  |  |  |  |  |  |  |  |  |  |  |  |  |  |
|  |  |  |  |  |  |  |  |  |  |  |  |  |  |
| **TP53_R273C_** | CVCACPGRD | 37042,1 | 38906,69 | 38427,66 | 44216,56 | 43174,14 | 43188,18 | 41011,3 | 39468,92 | 45875,46 | 46024,1 | 27593,62 | 37194,73 |
| **TP53_R273C_** | VCVCACPGR | 41658,45 | 34699,14 | 25475,12 | 43028,18 | 41299,41 | 42250,38 | 41882,62 | 36493,54 | 44389,13 | 44635,23 | 34948,2 | 39838,76 |
| **TP53_R273C_** | EVCVCACPG | 31239,29 | 32662,29 | 33650,21 | 41572,88 | 29512 | 32840,89 | 25575,37 | 34654,12 | 35097,88 | 43115,79 | 25095,93 | 23482,13 |
| **TP53_R273C_** | FEVCVCACP | 36538,96 | 27822,05 | 40149,89 | 39322,71 | 39072,9 | 35642,82 | 24497,39 | 31433,56 | 23255,08 | 4524,18 | 35697,64 | 24141,39 |
| **TP53_R273C_** | SFEVCVCAC | 34521,27 | 29378,5 | 36644,69 | 31824,38 | 44931,29 | 33328,79 | 19633,51 | 39632,8 | 31764,85 | 39915,99 | 41055,25 | 37572,11 |
| **TP53_R273C_** | NSFEVCVCA | 17260,44 | 2810,3 | 29008,63 | 35708,43 | 25823,68 | 30498,52 | 14545,8 | 29367,06 | 12697,78 | 25547,99 | 10697,73 | 16454,05 |
| **TP53_R273C_** | RNSFEVCVC | 36724,06 | 31792,71 | 36957,25 | 36884,94 | 44668,58 | 35018,97 | 29833,04 | 32437,62 | 26125,5 | 32843,04 | 15918,9 | 28647,44 |
| **TP53_R273C_** | GRNSFEVCV | 33111 | 20958,53 | 34263,39 | 33499,8 | 40672,37 | 37523,75 | 23747,6 | 652,34 | 4921,83 | 21403,8 | 33226,19 | 32696,24 |
| **TP53_R273C_** | LGRNSFEVC | 41508,18 | 33886,97 | 38281,18 | 39332,5 | 45429,42 | 21897,82 | 10887,01 | 34771,68 | 34800,28 | 38553,02 | 23700,88 | 19225,47 |
|  |  |  |  |  |  |  |  |  |  |  |  |  |  |
| **TP53_R273L_** | LVCACPGRD | 38037,18 | 36959,64 | 37265,22 | 44545,97 | 43494,86 | 42898,48 | 41273,5 | 39592,11 | 45936,06 | 45663,49 | 24104,32 | 34288,61 |
| **TP53_R273L_** | VLVCACPGR | 36696,64 | 14087,94 | 3566,54 | 36544,5 | 30716,42 | 38308,52 | 36843,86 | 24425,93 | 42245,8 | 42123,49 | 34559 | 26032,39 |
| **TP53_R273L_** | EVLVCACPG | 32725,63 | 29830,14 | 32957,29 | 41560,31 | 28519,71 | 28912,74 | 18422,72 | 34034,68 | 33459,97 | 41685,05 | 23979,72 | 23426,3 |
| **TP53_R273L_** | FEVLVCACP | 38398,58 | 29276,96 | 41690,02 | 40869,57 | 41095,25 | 38574,73 | 26095,56 | 35095,98 | 26278,88 | 5226,15 | 38434,33 | 26261,26 |
| **TP53_R273L_** | SFEVLVCAC | 36096,12 | 27711,5 | 38797,4 | 33383,67 | 44850,17 | 35945,68 | 18852,63 | 38875,57 | 28569,43 | 37554,64 | 41250,75 | 36488,79 |
| **TP53_R273L_** | NSFEVLVCA | 15868,68 | 1745,27 | 27291,94 | 36693,07 | 17957,88 | 30270,69 | 15202,14 | 28450,05 | 11618,86 | 26820,35 | 8501,49 | 16674,49 |
| **TP53_R273L_** | RNSFEVLVC | 34405,3 | 30518 | 34622,27 | 33438,25 | 44813,31 | 35402,98 | 30619,19 | 30312,65 | 24589,27 | 29566,33 | 11686,18 | 28232,35 |
| **TP53_R273L_** | GRNSFEVLV | 30358,92 | 17640,31 | 31390,39 | 29896,37 | 39060,23 | 37534,32 | 28504,91 | 175,18 | 5659,17 | 18428,9 | 28071,82 | 32118,75 |
| **TP53_R273L_** | LGRNSFEVL | 37173 | 26018,02 | 33673,15 | 23785,15 | 38670,43 | 2984,45 | 1009,63 | 24645,2 | 17527,68 | 25789,61 | 14537,15 | 3141,06 |
|  |  |  |  |  |  |  |  |  |  |  |  |  |  |
|  |  |  |  |  |  |  |  |  |  |  |  |  |  |
| **PROTEIN** | **PEPTIDE** | **A*01:01** | **A*02:01** | **A*03:01** | **A*24:02** | **A*26:02** | **B*07:02** | **B*08:02** | **B*27:05** | **B*39:01** | **B*40:01** | **B*58:01** | **B*15:01** |
| **TP53_248wt_** | RRPILTIIT | 43011,43 | 38687,16 | 38766,76 | 33510,33 | 44123,84 | 33750,48 | 30089,8 | 3281,02 | 21058,78 | 35305,06 | 40109,5 | 38324,29 |
| **TP53_248wt_** | NRRPILTII | 39440,32 | 36247,98 | 36214,26 | 28670,08 | 34098,08 | 15040,33 | 4556,66 | 1022,97 | 4891,35 | 32254,57 | 35194,46 | 24394,51 |
| **TP53_248wt_** | MNRRPILTI | 28035,71 | 25191,97 | 23078,88 | 11784,08 | 25990,74 | 3718,41 | 607,53 | 13528,07 | 10707,92 | 22112,34 | 5529,94 | 6292,61 |
| **TP53_248wt_** | GMNRRPILT | 31213,94 | 11368,04 | 16999,11 | 38148,44 | 43395,67 | 33484,6 | 8510,41 | 21935,05 | 34621,51 | 38200,49 | 30870,35 | 21145,3 |
| **TP53_248wt_** | GGMNRRPIL | 38800,35 | 27294,9 | 31095,64 | 30871,68 | 42039,25 | 3160,94 | 476,35 | 15572,06 | 21183,78 | 29625,25 | 26611,94 | 18645,91 |
| **TP53_248wt_** | MGGMNRRPI | 31564,77 | 27134,41 | 33039,05 | 22823,35 | 41001,54 | 5458,48 | 721,77 | 21499,89 | 16720,93 | 31660,9 | 15658,89 | 15672,29 |
| **TP53_248wt_** | CMGGMNRRP | 39437,75 | 24625,22 | 36949,65 | 39896,99 | 44987,22 | 39759,52 | 31056,29 | 34247,08 | 42453,84 | 42879,45 | 32905,98 | 28638,76 |
| **TP53_248wt_** | SCMGGMNRR | 38037,18 | 33271,88 | 15434 | 41156,68 | 28033,27 | 36101,98 | 38588,09 | 32029,21 | 39927,65 | 41509,98 | 32815,32 | 37208,81 |
| **TP53_248wt_** | SSCMGGMNR | 21319,21 | 34028,79 | 3897,27 | 37889,3 | 31477,46 | 36365,03 | 37245,07 | 25265,66 | 40209 | 41168,7 | 23587,8 | 29458,4 |
|  |  |  |  |  |  |  |  |  |  |  |  |  |  |
| **TP53_R248Q_** | QRPILTIIT | 43427,62 | 41050,83 | 42914,73 | 39696,75 | 43589,54 | 40365,07 | 33466,12 | 15493,4 | 16998,75 | 39099,14 | 43455,8 | 41102,83 |
| **TP53_R248Q_** | NQRPILTII | 36075,03 | 13361,08 | 30533,85 | 14809,74 | 24908,2 | 10377,6 | 3363 | 11315,52 | 4893,15 | 8597,04 | 24832,31 | 649,32 |
| **TP53_R248Q_** | MNQRPILTI | 23713,19 | 18237,48 | 26426,58 | 5414,9 | 30776,98 | 15963,4 | 4628,01 | 14192,28 | 4611,86 | 18239,26 | 3345,33 | 13064,3 |
| **TP53_R248Q_** | GMNQRPILT | 32195,29 | 7683,67 | 19771,22 | 39086,86 | 42906,84 | 34292,32 | 12025,59 | 26831,66 | 33761,43 | 37127,58 | 31676,32 | 19493,16 |
| **TP53_R248Q_** | GGMNQRPIL | 39275,52 | 26258,7 | 33221,88 | 32699,78 | 42511,76 | 5258,94 | 5876,51 | 18878,76 | 19701,81 | 26797,71 | 23059,15 | 18188,8 |
| **TP53_R248Q_** | MGGMNQRPI | 30526,92 | 22434,31 | 34585,94 | 22397,44 | 41402,77 | 15002,63 | 3227,57 | 25804,96 | 17502,66 | 31534,74 | 9166,52 | 16783,46 |
| **TP53_R248Q_** | CMGGMNQRP | 36461,17 | 20644,12 | 37119,54 | 35714,24 | 44785,69 | 39950,55 | 32426,74 | 35075,84 | 41276,64 | 41650,79 | 30428,31 | 27789,56 |
| **TP53_R248Q_** | SCMGGMNQR | 38549,69 | 32475,19 | 14880,25 | 41324 | 25906,23 | 34850,39 | 37927,86 | 31385,31 | 38491,75 | 40306,57 | 31920,25 | 36096,51 |
| **TP53_R248Q_** | SSCMGGMNQ | 26398,56 | 37946,33 | 21246,67 | 44820,59 | 40175,09 | 38807,91 | 39865,05 | 36662,91 | 41850,01 | 42906,37 | 16908,32 | 23629,96 |
|  |  |  |  |  |  |  |  |  |  |  |  |  |  |
| **TP53_R248W_** | WRPILTIIT | 39685,16 | 37548,54 | 42132,13 | 36718,89 | 41870,39 | 36355,98 | 24268,14 | 8509,77 | 9682,67 | 35704,96 | 42460,71 | 37022,47 |
| **TP53_R248W_** | NWRPILTII | 39252,57 | 25926,42 | 32622,73 | 944,75 | 31371,04 | 17674,7 | 4897,55 | 20649,03 | 28138,72 | 35970,98 | 31422,68 | 15939,41 |
| **TP53_R248W_** | MNWRPILTI | 23349,62 | 4791,83 | 19863,63 | 5037,66 | 28502,45 | 15374 | 4467,66 | 4672,79 | 3053,44 | 11320,54 | 869,49 | 10038,77 |
| **TP53_R248W_** | GMNWRPILT | 28454,68 | 3415,29 | 13631,95 | 34828,91 | 42270,5 | 32416,91 | 12813,16 | 23711,91 | 30040,34 | 34127,23 | 28693,98 | 19478,84 |
| **TP53_R248W_** | GGMNWRPIL | 36140,28 | 12923,85 | 26702,51 | 20156,12 | 39150,77 | 5010,43 | 6775,31 | 10591,19 | 12431,48 | 23156,91 | 14014,36 | 14211,03 |
| **TP53_R248W_** | MGGMNWRPI | 25316,56 | 13629,3 | 28997,33 | 9830,88 | 37326,56 | 10604,04 | 1678,56 | 18592,93 | 13706,64 | 26937,84 | 8533,28 | 12504,32 |
| **TP53_R248W_** | CMGGMNWRP | 31339,82 | 5910,62 | 32170,22 | 24888,8 | 43850,18 | 41085,48 | 26715,22 | 28922,14 | 36674,43 | 39732,87 | 22971,99 | 29227,27 |
| **TP53_R248W_** | SCMGGMNWR | 37512,79 | 29803,04 | 14043,35 | 40078,7 | 25577,58 | 36635,94 | 36048,89 | 28356,32 | 39941,04 | 40604,23 | 30745,7 | 36661,33 |
| **TP53_R248W_** | SSCMGGMNW | 13361,21 | 35375,8 | 23490,53 | 11386,5 | 24675,63 | 30402,97 | 31584,93 | 27410,62 | 34487,67 | 33845,19 | 14,01 | 6103,58 |
|  |  |  |  |  |  |  |  |  |  |  |  |  |  |
|  |  |  |  |  |  |  |  |  |  |  |  |  |  |
| **PROTEIN** | **PEPTIDE** | **A*01:01** | **A*02:01** | **A*03:01** | **A*24:02** | **A*26:02** | **B*07:02** | **B*08:02** | **B*27:05** | **B*39:01** | **B*40:01** | **B*58:01** | **B*15:01** |
| **TP53_245wt_** | GMNRRPILT | 31213,94 | 11368,04 | 16999,11 | 38148,44 | 43395,67 | 33484,6 | 8510,41 | 21935,05 | 34621,51 | 38200,49 | 30870,35 | 21145,3 |
| **TP53_245wt_** | GGMNRRPIL | 38800,35 | 27294,9 | 31095,64 | 30871,68 | 42039,25 | 3160,94 | 476,35 | 15572,06 | 21183,78 | 29625,25 | 26611,94 | 18645,91 |
| **TP53_245wt_** | MGGMNRRPI | 31564,77 | 27134,41 | 33039,05 | 22823,35 | 41001,54 | 5458,48 | 721,77 | 21499,89 | 16720,93 | 31660,9 | 15658,89 | 15672,29 |
| **TP53_245wt_** | CMGGMNRRP | 39437,75 | 24625,22 | 36949,65 | 39896,99 | 44987,22 | 39759,52 | 31056,29 | 34247,08 | 42453,84 | 42879,45 | 32905,98 | 28638,76 |
| **TP53_245wt_** | SCMGGMNRR | 38037,18 | 33271,88 | 15434 | 41156,68 | 28033,27 | 36101,98 | 38588,09 | 32029,21 | 39927,65 | 41509,98 | 32815,32 | 37208,81 |
| **TP53_245wt_** | SSCMGGMNR | 21319,21 | 34028,79 | 3897,27 | 37889,3 | 31477,46 | 36365,03 | 37245,07 | 25265,66 | 40209 | 41168,7 | 23587,8 | 29458,4 |
| **TP53_245wt_** | NSSCMGGMN | 23150,14 | 42006,51 | 37158,53 | 43399,9 | 37720,37 | 38016,19 | 37076,59 | 35373,52 | 42537,51 | 43365,15 | 13663,55 | 32361,89 |
| **TP53_245wt_** | CNSSCMGGM | 18057,44 | 30628,14 | 26863,33 | 34253,38 | 13145,12 | 25735,81 | 22591,17 | 22885,66 | 25128,27 | 33735,14 | 17494,33 | 15305,29 |
| **TP53_245wt_** | MCNSSCMGG | 32869,33 | 29674,66 | 33146,83 | 37967,29 | 42311,21 | 34981,87 | 23723,72 | 34183,02 | 35818,34 | 38174,46 | 9397,59 | 19698,4 |
|  |  |  |  |  |  |  |  |  |  |  |  |  |  |
| **TP53_G245S_** | SMNRRPILT | 25089,42 | 9113,41 | 11195,81 | 33094,52 | 40119,92 | 26017,47 | 1648,03 | 21587,54 | 26565,04 | 37646,15 | 25538,58 | 16792,18 |
| **TP53_G245S_** | GSMNRRPIL | 30073,19 | 19260,87 | 21368,16 | 25049,28 | 32576,54 | 989,06 | 556,13 | 10347,77 | 17766,73 | 21160,42 | 7710,41 | 7526,76 |
| **TP53_G245S_** | MGSMNRRPI | 27692,31 | 23155,16 | 28290,13 | 21003,25 | 36455,64 | 2231,94 | 540,56 | 17438,77 | 9102,57 | 25557,1 | 7377,99 | 9489,35 |
| **TP53_G245S_** | CMGSMNRRP | 37395,68 | 24050,14 | 34766,02 | 38072,99 | 44696,13 | 38473,44 | 27536,06 | 32638,98 | 42123,49 | 42511,29 | 30653,67 | 26294,8 |
| **TP53_G245S_** | SCMGSMNRR | 37548,95 | 33119,22 | 13601,16 | 40567,35 | 29230,43 | 35841,61 | 37967,68 | 30620,2 | 40173,36 | 41778,06 | 31040,5 | 36387,48 |
| **TP53_G245S_** | SSCMGSMNR | 20910,06 | 33061,24 | 2610,14 | 36691,89 | 31971,4 | 37016,88 | 36809,59 | 25637,15 | 39772,86 | 41215,48 | 21003,48 | 29279,51 |
| **TP53_G245S_** | NSSCMGSMN | 20866,44 | 40442,88 | 35468,55 | 42305,73 | 36891,31 | 35987,71 | 34393,76 | 34591,94 | 40841,27 | 42708,62 | 9941,06 | 29595,77 |
| **TP53_G245S_** | CNSSCMGSM | 16585,25 | 29902,85 | 24652,67 | 30636,44 | 10813,52 | 16802,54 | 15219,09 | 21648,59 | 17568,12 | 29663,41 | 14063,27 | 7564,8 |
| **TP53_G245S_** | MCNSSCMGS | 30385,87 | 24631,35 | 30633,12 | 40652,15 | 42340,53 | 34990,19 | 28213,4 | 35632,41 | 35004,21 | 38446,8 | 15870,57 | 23589,08 |
|  |  |  |  |  |  |  |  |  |  |  |  |  |  |
|  |  |  |  |  |  |  |  |  |  |  |  |  |  |
| **PROTEIN** | **PEPTIDE** | **A*01:01** | **A*02:01** | **A*03:01** | **A*24:02** | **A*26:02** | **B*07:02** | **B*08:02** | **B*27:05** | **B*39:01** | **B*40:01** | **B*58:01** | **B*15:01** |
| **TP53_220wt_** | YEPPEVGSD | 44588,88 | 44202,71 | 46858,7 | 42928,68 | 42194,64 | 42276,44 | 40618,29 | 43585,78 | 39963,95 | 29027,16 | 44785,69 | 37072,19 |
| **TP53_220wt_** | PYEPPEVGS | 44731,45 | 45655,6 | 45073,47 | 41485,28 | 48291,68 | 46218,2 | 46928,71 | 46770,57 | 46390,06 | 47534,8 | 46393,08 | 47508,08 |
| **TP53_220wt_** | VPYEPPEVG | 43124,66 | 40913,82 | 40342,79 | 44183,55 | 43900,52 | 6952,8 | 30671,27 | 40584,91 | 24890,68 | 37739,96 | 27604,07 | 32975,13 |
| **TP53_220wt_** | VVPYEPPEV | 30533,18 | 803,37 | 34707,4 | 20328,04 | 25781,24 | 30884,05 | 33740,98 | 39454,82 | 33930,62 | 37698,34 | 32475,54 | 25992,43 |
| **TP53_220wt_** | VVVPYEPPE | 42140,34 | 25842,4 | 31171,09 | 44314,74 | 38914,7 | 33436,07 | 37420,36 | 41073,93 | 40325,34 | 41154 | 27628,57 | 26370,59 |
| **TP53_220wt_** | SVVVPYEPP | 38113,38 | 19354,46 | 33685,9 | 38830,16 | 31308,31 | 31186,27 | 33569,83 | 39249,61 | 33310,79 | 32754,32 | 22698,24 | 20965,79 |
| **TP53_220wt_** | HSVVVPYEP | 31581,85 | 32033,38 | 36600,31 | 39209,29 | 39012,5 | 37250,3 | 33704,5 | 37697,93 | 26315,87 | 33880 | 3122,94 | 29331,18 |
| **TP53_220wt_** | RHSVVVPYE | 42783,08 | 34751,75 | 31077,47 | 32870,41 | 44818,66 | 37296,67 | 38034,29 | 23105,1 | 19575,6 | 34975,8 | 26041,41 | 37340,69 |
| **TP53_220wt_** | FRHSVVVPY | 19947,63 | 29670,49 | 18547,52 | 25541,91 | 10263,81 | 25677,12 | 15073,89 | 53,29 | 2245,24 | 23997,37 | 21994,23 | 1456,24 |
|  |  |  |  |  |  |  |  |  |  |  |  |  |  |
| **TP53_Y220C_** | CEPPEVGSD | 44506,95 | 45108,11 | 46593,8 | 43058,91 | 43750,19 | 43992,29 | 42832,16 | 43212,47 | 42799,75 | 34312,72 | 43529,23 | 39128,75 |
| **TP53_Y220C_** | PCEPPEVGS | 45713,44 | 46039,53 | 46960,2 | 48728,88 | 49044,12 | 48067,53 | 48129,44 | 48379,52 | 48244,14 | 47868,74 | 45739,65 | 48085,2 |
| **TP53_Y220C_** | VPCEPPEVG | 44491,54 | 43877,71 | 44533,94 | 45701,05 | 47044,13 | 21873,91 | 38355,81 | 42879,45 | 34451,48 | 42773,34 | 33237,33 | 39401,5 |
| **TP53_Y220C_** | VVPCEPPEV | 29476,89 | 564,05 | 35083,45 | 20861,25 | 26233,7 | 32180,66 | 33576,37 | 37469,39 | 32843,04 | 36179,02 | 31701 | 25978,92 |
| **TP53_Y220C_** | VVVPCEPPE | 41964,71 | 27649,2 | 29765,34 | 44756,12 | 40802,41 | 33809,7 | 39699,34 | 40721,26 | 41649,88 | 41815,61 | 26783,51 | 26881,35 |
| **TP53_Y220C_** | SVVVPCEPP | 37371,41 | 14956,28 | 33792,13 | 40847,46 | 34351,73 | 30126,59 | 32915,59 | 37835,64 | 32268,17 | 32270,98 | 23373,14 | 19204,48 |
| **TP53_Y220C_** | HSVVVPCEP | 30796,95 | 34855,29 | 36043,43 | 43624,92 | 38109,66 | 34819,86 | 34808,94 | 37842,18 | 29418,26 | 36714,14 | 5360,74 | 25425,27 |
| **TP53_Y220C_** | RHSVVVPCE | 43346,86 | 39088,55 | 35416,02 | 35339,45 | 45669,41 | 38430,59 | 36120,34 | 24192,9 | 18974,6 | 36898,5 | 30430,62 | 38278,29 |
| **TP53_Y220C_** | FRHSVVVPC | 40897,44 | 22781,16 | 39118,59 | 36693,88 | 39127,06 | 25848,55 | 9277,17 | 1031,74 | 800,01 | 26734,88 | 35867,99 | 26615,1 |
|  |  |  |  |  |  |  |  |  |  |  |  |  |  |
|  |  |  |  |  |  |  |  |  |  |  |  |  |  |
| **PROTEIN** | **PEPTIDE** | **A*01:01** | **A*02:01** | **A*03:01** | **A*24:02** | **A*26:02** | **B*07:02** | **B*08:02** | **B*27:05** | **B*39:01** | **B*40:01** | **B*58:01** | **B*15:01** |
| **TP53_195wt_** | IRVEGNLRV | 36096,51 | 23411,61 | 37850,38 | 33494,73 | 36145,76 | 34299 | 31254,51 | 781,19 | 3437,13 | 29838,53 | 31095,29 | 33467,2 |
| **TP53_195wt_** | LIRVEGNLR | 35636,27 | 35376,58 | 7690,08 | 41797,06 | 31263,64 | 30418,44 | 28697,7 | 29319,13 | 44089,03 | 43490,14 | 39910,81 | 25706,87 |
| **TP53_195wt_** | HLIRVEGNL | 35034,52 | 2686,41 | 25826,47 | 31604,76 | 18050,79 | 17030,59 | 9688,33 | 20809,17 | 10853,72 | 23549,82 | 20536,52 | 4150,75 |
| **TP53_195wt_** | QHLIRVEGN | 45222,96 | 46069,95 | 44036,09 | 41765,4 | 46729,09 | 44380,49 | 37574,55 | 33815,54 | 32684,92 | 45928,59 | 39043,76 | 43397,55 |
| **TP53_195wt_** | PQHLIRVEG | 44351,19 | 43025,85 | 43977,99 | 40362,44 | 46426,72 | 41073,93 | 31948,56 | 32232,23 | 36186,83 | 37514,82 | 40441,99 | 27645,92 |
| **TP53_195wt_** | PPQHLIRVE | 46679,06 | 46300,81 | 43795,18 | 46949,54 | 47438,21 | 29462,86 | 34365,49 | 42546,73 | 44240,01 | 46290,78 | 44788,58 | 44376,17 |
| **TP53_195wt_** | APPQHLIRV | 36144,96 | 19095,9 | 36289,57 | 31867,78 | 23068,64 | 1370,82 | 9630,75 | 34259,67 | 18519,85 | 32660,54 | 37963,59 | 35251,64 |
| **TP53_195wt_** | LAPPQHLIR | 28417,14 | 35364,33 | 20069,72 | 33504,52 | 30320,18 | 33110,27 | 30330,68 | 31496,2 | 38605,19 | 41794,34 | 29302 | 31489,72 |
| **TP53_195wt_** | GLAPPQHLI | 24552,59 | 128,74 | 12699,85 | 10582,03 | 30138,66 | 21723,2 | 24203,11 | 20498,33 | 20786,89 | 19511,1 | 8113 | 8770,29 |
|  |  |  |  |  |  |  |  |  |  |  |  |  |  |
| **TP53_I195T_** | TRVEGNLRV | 35734,72 | 27512,24 | 38132,77 | 35692,62 | 31287,66 | 33595,63 | 29459,37 | 1050,53 | 1129,83 | 29138,87 | 34872,26 | 35067,5 |
| **TP53_I195T_** | LTRVEGNLR | 25140,23 | 37993,57 | 9018,37 | 40973,6 | 25748,34 | 31247,07 | 31362,89 | 28724,42 | 44109,53 | 42411,59 | 30847,32 | 26021,97 |
| **TP53_I195T_** | HLTRVEGNL | 33452,01 | 5963,49 | 29394,73 | 34917,58 | 31077,47 | 19343,99 | 15341,93 | 26049,02 | 14816,95 | 28391,64 | 23200,05 | 12395,08 |
| **TP53_I195T_** | QHLTRVEGN | 45051,53 | 46147,26 | 43866,81 | 41872,2 | 46292,78 | 43840,71 | 38572,21 | 33602,17 | 31096,64 | 45768,86 | 39064,03 | 43170,41 |
| **TP53_I195T_** | PQHLTRVEG | 44909,91 | 44328,17 | 44393,95 | 41961,08 | 46712,91 | 40602,03 | 34371,81 | 34362,52 | 38144,75 | 39708,79 | 41190,96 | 27007,59 |
| **TP53_I195T_** | PPQHLTRVE | 46077,91 | 46629,09 | 43613,14 | 47028,85 | 47728,57 | 26315,02 | 34612,89 | 42764,1 | 43862,99 | 46491,56 | 45157,44 | 44294,14 |
| **TP53_I195T_** | APPQHLTRV | 36355,6 | 22084,13 | 37934,83 | 34756,63 | 21730,48 | 867,12 | 7733,3 | 34832,3 | 20806,7 | 34581,07 | 39113,95 | 33992,36 |
| **TP53_I195T_** | LAPPQHLTR | 27836,5 | 32767,08 | 16812,18 | 31540,54 | 27617,21 | 30190,89 | 27674,05 | 30093,7 | 35983,44 | 41303 | 25930,63 | 29051,65 |
| **TP53_I195T_** | GLAPPQHLT | 31670,82 | 2140,39 | 19586,4 | 38655,37 | 39040,79 | 32889,26 | 34766,02 | 32642,52 | 35175,81 | 33217,55 | 28540,39 | 22715,2 |
|  |  |  |  |  |  |  |  |  |  |  |  |  |  |
|  |  |  |  |  |  |  |  |  |  |  |  |  |  |
| **PROTEIN** | **PEPTIDE** | **A*01:01** | **A*02:01** | **A*03:01** | **A*24:02** | **A*26:02** | **B*07:02** | **B*08:02** | **B*27:05** | **B*39:01** | **B*40:01** | **B*58:01** | **B*15:01** |
| **TP53_193wt_** | HLIRVEGNL | 35034,52 | 2686,41 | 25826,47 | 31604,76 | 18050,79 | 17030,59 | 9688,33 | 20809,17 | 10853,72 | 23549,82 | 20536,52 | 4150,75 |
| **TP53_193wt_** | QHLIRVEGN | 45222,96 | 46069,95 | 44036,09 | 41765,4 | 46729,09 | 44380,49 | 37574,55 | 33815,54 | 32684,92 | 45928,59 | 39043,76 | 43397,55 |
| **TP53_193wt_** | PQHLIRVEG | 44351,19 | 43025,85 | 43977,99 | 40362,44 | 46426,72 | 41073,93 | 31948,56 | 32232,23 | 36186,83 | 37514,82 | 40441,99 | 27645,92 |
| **TP53_193wt_** | PPQHLIRVE | 46679,06 | 46300,81 | 43795,18 | 46949,54 | 47438,21 | 29462,86 | 34365,49 | 42546,73 | 44240,01 | 46290,78 | 44788,58 | 44376,17 |
| **TP53_193wt_** | APPQHLIRV | 36144,96 | 19095,9 | 36289,57 | 31867,78 | 23068,64 | 1370,82 | 9630,75 | 34259,67 | 18519,85 | 32660,54 | 37963,59 | 35251,64 |
| **TP53_193wt_** | LAPPQHLIR | 28417,14 | 35364,33 | 20069,72 | 33504,52 | 30320,18 | 33110,27 | 30330,68 | 31496,2 | 38605,19 | 41794,34 | 29302 | 31489,72 |
| **TP53_193wt_** | GLAPPQHLI | 24552,59 | 128,74 | 12699,85 | 10582,03 | 30138,66 | 21723,2 | 24203,11 | 20498,33 | 20786,89 | 19511,1 | 8113 | 8770,29 |
| **TP53_193wt_** | DGLAPPQHL | 42343,26 | 39270,83 | 43214,34 | 36000,17 | 41061,93 | 29359,13 | 19982,19 | 41752,77 | 25125,54 | 38779,79 | 32230,49 | 37871,28 |
| **TP53_193wt_** | SDGLAPPQH | 38253,44 | 47028,85 | 41493,36 | 47718,79 | 44045,15 | 44818,66 | 44573,47 | 43807,98 | 46369,48 | 42169,54 | 43158,75 | 40083,89 |
|  |  |  |  |  |  |  |  |  |  |  |  |  |  |
| **TP53_H193R_** | RLIRVEGNL | 36445,79 | 935,8 | 15897,38 | 20155,46 | 29663,41 | 10784,19 | 11991,67 | 9442,65 | 19378,97 | 16990,65 | 10543,63 | 1686,8 |
| **TP53_H193R_** | QRLIRVEGN | 46632,12 | 45811,45 | 44875,89 | 44908,44 | 46629,6 | 44942,97 | 31889,51 | 6393,35 | 38862,53 | 45256,71 | 39949,7 | 43118,6 |
| **TP53_H193R_** | PQRLIRVEG | 44712,1 | 44544,05 | 42399,66 | 41317,73 | 46235,73 | 36501,02 | 24519,68 | 31011,28 | 40803,28 | 40634,56 | 41162,92 | 25847,44 |
| **TP53_H193R_** | PPQRLIRVE | 46374 | 46337,38 | 43272,37 | 46455,36 | 47635,71 | 26702,78 | 29589,05 | 41057,93 | 43211,52 | 46291,29 | 44180,22 | 43816,99 |
| **TP53_H193R_** | APPQRLIRV | 38332,99 | 24296,25 | 37967,68 | 34060,83 | 28405,78 | 1460,72 | 4271,91 | 34985,66 | 23267,92 | 37605,85 | 40020,2 | 38328,44 |
| **TP53_H193R_** | LAPPQRLIR | 33684,45 | 38466,37 | 21303,06 | 37997,68 | 32627,68 | 32747,58 | 30635,45 | 31425,07 | 40761,8 | 43617,86 | 34039,84 | 33981,32 |
| **TP53_H193R_** | GLAPPQRLI | 33093,79 | 1815,28 | 13953,23 | 22121,44 | 33565,11 | 19690,73 | 26352,91 | 21396,86 | 30662,96 | 26438,29 | 12997,75 | 10315,46 |
| **TP53_H193R_** | DGLAPPQRL | 41408,59 | 39967,4 | 43053,8 | 35954,23 | 40502,44 | 31055,96 | 20381,12 | 40823,6 | 26146,14 | 39192,32 | 33132,12 | 40712,44 |
| **TP53_H193R_** | SDGLAPPQR | 38412,7 | 45185,29 | 35971,35 | 45900,76 | 43285,46 | 44349,27 | 44252,94 | 41281,11 | 45842,69 | 44747,9 | 43622,11 | 44703,39 |
|  |  |  |  |  |  |  |  |  |  |  |  |  |  |
|  |  |  |  |  |  |  |  |  |  |  |  |  |  |
| **PROTEIN** | **PEPTIDE** | **A*01:01** | **A*02:01** | **A*03:01** | **A*24:02** | **A*26:02** | **B*07:02** | **B*08:02** | **B*27:05** | **B*39:01** | **B*40:01** | **B*58:01** | **B*15:01** |
| **TP53_163wt_** | YKQSQHMTE | 41772,19 | 41224,87 | 41120,62 | 41179,83 | 43900,52 | 34897,19 | 15124,22 | 22055 | 13886,21 | 34894,53 | 40663,58 | 32416,2 |
| **TP53_163wt_** | IYKQSQHMT | 42791,41 | 42037,87 | 38593,09 | 16502,56 | 43606,05 | 37842,59 | 25873,74 | 37257,97 | 39304 | 44657,46 | 40725,23 | 36801,23 |
| **TP53_163wt_** | AIYKQSQHM | 31531,67 | 9054,05 | 6644,13 | 25435,18 | 7525,62 | 5821,01 | 7557,52 | 22049,51 | 24193,68 | 26581,43 | 12765,83 | 192,07 |
| **TP53_163wt_** | MAIYKQSQH | 31334,06 | 37982,47 | 14680,51 | 44314,28 | 11593,74 | 23446,85 | 13018,3 | 28653,65 | 30681,88 | 35315,37 | 10893,84 | 5490,29 |
| **TP53_163wt_** | AMAIYKQSQ | 39210,98 | 25267,03 | 19203,44 | 43364,23 | 41473,16 | 29973,15 | 19835,71 | 31536,45 | 38074,24 | 39356,35 | 30625,49 | 10908,94 |
| **TP53_163wt_** | RAMAIYKQS | 39499,68 | 24570,66 | 22018,52 | 33441,87 | 39514,2 | 13683,22 | 15801,35 | 14719,8 | 32700,48 | 34086,27 | 4719,48 | 14755,83 |
| **TP53_163wt_** | VRAMAIYKQ | 43626,84 | 41286,46 | 37095,05 | 40599,83 | 43222,28 | 42329,07 | 37816 | 2626,03 | 29000,79 | 41110,39 | 34697,64 | 38299,82 |
| **TP53_163wt_** | RVRAMAIYK | 28640 | 23964,17 | 7,61 | 25419,76 | 33270,45 | 12084,8 | 21645,3 | 3539,52 | 41726,12 | 36241,29 | 16983,49 | 12469,19 |
| **TP53_163wt_** | TRVRAMAIY | 24818,61 | 40917,36 | 23367,58 | 36074,64 | 7799,09 | 35927,02 | 33116,72 | 509,54 | 13330,46 | 32200,86 | 24354,69 | 5678,3 |
|  |  |  |  |  |  |  |  |  |  |  |  |  |  |
| **TP53_Y163C_** | CKQSQHMTE | 42545,33 | 43818,41 | 40192,93 | 43208,26 | 45657,57 | 38000,16 | 22819,39 | 24165,43 | 29789,5 | 39981,25 | 40991,36 | 36685,55 |
| **TP53_Y163C_** | ICKQSQHMT | 43528,75 | 40996,68 | 42990,02 | 45997,72 | 47062,95 | 38557,62 | 28934,96 | 44201,74 | 44995,98 | 45252,3 | 35538,09 | 39394,26 |
| **TP53_Y163C_** | AICKQSQHM | 32171,97 | 23324,62 | 24519,68 | 32873,25 | 23816,81 | 18917,2 | 20296,83 | 33217,92 | 35668,66 | 35659,03 | 19969,65 | 1555,45 |
| **TP53_Y163C_** | MAICKQSQH | 30141,6 | 36240,92 | 15396,31 | 43637,67 | 12683,23 | 22364,03 | 10352,48 | 25418,12 | 29557,05 | 34626,38 | 11178,5 | 4492,43 |
| **TP53_Y163C_** | AMAICKQSQ | 39557,42 | 26279,73 | 16862,82 | 43775,76 | 42665,18 | 29479,13 | 26381,15 | 31157,6 | 40935,52 | 40536,17 | 29868,25 | 12722,68 |
| **TP53_Y163C_** | RAMAICKQS | 38443,05 | 22990,14 | 23167,93 | 36266,01 | 41268,15 | 16021,36 | 17805,41 | 16293,91 | 33310,05 | 34116,52 | 6015,59 | 15082,7 |
| **TP53_Y163C_** | VRAMAICKQ | 44076,6 | 42764,1 | 37222,11 | 43862,99 | 42606,61 | 41296,74 | 37670,2 | 4314,92 | 32695,52 | 42899,87 | 37492,52 | 36524,73 |
| **TP53_Y163C_** | RVRAMAICK | 31135,69 | 29683,96 | 21,08 | 29777,57 | 36972,84 | 13643,31 | 21100,97 | 6913,79 | 41925,67 | 37229,36 | 20495,67 | 14990,95 |
| **TP53_Y163C_** | TRVRAMAIC | 43516,04 | 38425,18 | 40454,25 | 40263,86 | 38074,24 | 33560,03 | 28293,5 | 4909,75 | 9945,58 | 34650,75 | 35548,08 | 35118,39 |
|  |  |  |  |  |  |  |  |  |  |  |  |  |  |
|  |  |  |  |  |  |  |  |  |  |  |  |  |  |
| **PROTEIN** | **PEPTIDE** | **A*01:01** | **A*02:01** | **A*03:01** | **A*24:02** | **A*26:02** | **B*07:02** | **B*08:02** | **B*27:05** | **B*39:01** | **B*40:01** | **B*58:01** | **B*15:01** |
| **TP53_175wt_** | RCPHHERCS | 45799,57 | 44766,31 | 44445,36 | 46018,64 | 47818,54 | 42622,75 | 38975,8 | 43024,93 | 47009,02 | 47071,63 | 42947,73 | 44224,69 |
| **TP53_175wt_** | RRCPHHERC | 43778,61 | 39276,78 | 38844,44 | 36130,12 | 44997,46 | 36040,31 | 28482,71 | 2126,71 | 24280,23 | 35375,8 | 34065,62 | 39534,75 |
| **TP53_175wt_** | VRRCPHHER | 43047,75 | 40182,04 | 22853,5 | 38654,09 | 42800,66 | 31189,63 | 23177,46 | 1041,56 | 32166,74 | 41232,9 | 42357,46 | 36903,71 |
| **TP53_175wt_** | VVRRCPHHE | 42452 | 38697,62 | 22234,95 | 43592,38 | 43251,31 | 23945,75 | 22779,44 | 35158,3 | 45859,57 | 44902,12 | 32201,21 | 25792,69 |
| **TP53_175wt_** | EVVRRCPHH | 38501,34 | 43157,32 | 28311,26 | 45985,77 | 5704,47 | 35026,18 | 21786,28 | 37102,28 | 42237,11 | 43595,2 | 40179,42 | 21332,36 |
| **TP53_175wt_** | TEVVRRCPH | 36648,25 | 45067,63 | 35500,04 | 44906,99 | 33002,98 | 29128,78 | 16693,64 | 25734,42 | 29349,59 | 22555,76 | 40512,94 | 18928,46 |
| **TP53_175wt_** | MTEVVRRCP | 26190,88 | 38159,59 | 35935,96 | 42429,5 | 41083,7 | 30365,83 | 22327,02 | 37826,63 | 36752,28 | 39678,73 | 18806,79 | 33338,89 |
| **TP53_175wt_** | HMTEVVRRC | 39556,12 | 5812,57 | 31358,83 | 37688,13 | 34600,17 | 33159,38 | 18890,4 | 26118,44 | 23749,91 | 34294,54 | 24233,25 | 18943,21 |
| **TP53_175wt_** | QHMTEVVRR | 39410,45 | 38059 | 23203,8 | 33951,55 | 38407,72 | 40230,32 | 37259,17 | 14489,26 | 23738,87 | 41315,07 | 36441,05 | 38267,11 |
|  |  |  |  |  |  |  |  |  |  |  |  |  |  |
| **TP53_R175H_** | HCPHHERCS | 45432,38 | 46022,11 | 46191,71 | 47214,95 | 46955,63 | 43356,26 | 37270,87 | 45836,24 | 45685,23 | 47837,17 | 44884,14 | 45445,15 |
| **TP53_R175H_** | RHCPHHERC | 41866,31 | 39960,93 | 40275,62 | 30777,97 | 45839,23 | 37407,02 | 33783,37 | 24723,21 | 14089,92 | 35396,09 | 35439,39 | 39728,56 |
| **TP53_R175H_** | VRHCPHHER | 41853,62 | 38094,01 | 24090,77 | 37525,39 | 42238,94 | 35696,85 | 28170,1 | 482,34 | 23247,03 | 39560,84 | 40991,79 | 37701,6 |
| **TP53_R175H_** | VVRHCPHHE | 42487,36 | 36871,38 | 21792,64 | 44131,02 | 42572,5 | 26207,04 | 28118,64 | 37511,58 | 46044,02 | 44683,56 | 34494,39 | 25379,09 |
| **TP53_R175H_** | EVVRHCPHH | 36714,52 | 42515,43 | 26862,76 | 45728,27 | 3022,08 | 35741,31 | 27800,08 | 37000,46 | 41127,28 | 42279,19 | 38264,2 | 17290,16 |
| **TP53_R175H_** | TEVVRHCPH | 34579,58 | 44286,48 | 34085,53 | 44265,38 | 32271,67 | 30815,29 | 17082,82 | 25841 | 26303,63 | 17961,18 | 39924,2 | 15681,96 |
| **TP53_R175H_** | MTEVVRHCP | 15749,46 | 32958,71 | 34492,89 | 38036,77 | 38650,75 | 30209,18 | 19805,69 | 37369 | 29739,26 | 36362,68 | 14212,26 | 31828,5 |
| **TP53_R175H_** | HMTEVVRHC | 40131,2 | 4543,17 | 31088,22 | 36924,88 | 34124,66 | 32341,24 | 16802,54 | 27495,87 | 22374,43 | 33536,45 | 22385,57 | 12648,56 |
| **TP53_R175H_** | QHMTEVVRH | 40045,76 | 43561,25 | 29924,86 | 40586,66 | 37580,22 | 41483,03 | 39097,45 | 25218,97 | 17490,17 | 38342,53 | 38436,81 | 28241,82 |
|  |  |  |  |  |  |  |  |  |  |  |  |  |  |
|  |  |  |  |  |  |  |  |  |  |  |  |  |  |
| **PROTEIN** | **PEPTIDE** | **A*01:01** | **A*02:01** | **A*03:01** | **A*24:02** | **A*26:02** | **B*07:02** | **B*08:02** | **B*27:05** | **B*39:01** | **B*40:01** | **B*58:01** | **B*15:01** |
| **TP53_179wt_** | HERCSDSDG | 44646,84 | 47125,64 | 45273,38 | 45967,35 | 45543,09 | 36676,41 | 33183,08 | 36724,06 | 41280,21 | 27621,09 | 39869,37 | 30703,47 |
| **TP53_179wt_** | HHERCSDSD | 43762,04 | 46948,54 | 46305,32 | 42831,7 | 47533,79 | 43709,52 | 40812,99 | 38422,68 | 20203,06 | 43790,44 | 40099,95 | 43800,89 |
| **TP53_179wt_** | PHHERCSDS | 46060,47 | 47788,02 | 46854,14 | 45806,01 | 47655,31 | 44901,17 | 40239,04 | 43174,14 | 42078,39 | 47777,14 | 46986,65 | 46189,21 |
| **TP53_179wt_** | CPHHERCSD | 43429,02 | 45425,96 | 42622,28 | 45527,82 | 45782,71 | 11904,03 | 19669,22 | 37481,95 | 36075,43 | 45148,65 | 35942,18 | 39114,78 |
| **TP53_179wt_** | RCPHHERCS | 45799,57 | 44766,31 | 44445,36 | 46018,64 | 47818,54 | 42622,75 | 38975,8 | 43024,93 | 47009,02 | 47071,63 | 42947,73 | 44224,69 |
| **TP53_179wt_** | RRCPHHERC | 43778,61 | 39276,78 | 38844,44 | 36130,12 | 44997,46 | 36040,31 | 28482,71 | 2126,71 | 24280,23 | 35375,8 | 34065,62 | 39534,75 |
| **TP53_179wt_** | VRRCPHHER | 43047,75 | 40182,04 | 22853,5 | 38654,09 | 42800,66 | 31189,63 | 23177,46 | 1041,56 | 32166,74 | 41232,9 | 42357,46 | 36903,71 |
| **TP53_179wt_** | VVRRCPHHE | 42452 | 38697,62 | 22234,95 | 43592,38 | 43251,31 | 23945,75 | 22779,44 | 35158,3 | 45859,57 | 44902,12 | 32201,21 | 25792,69 |
| **TP53_179wt_** | EVVRRCPHH | 38501,34 | 43157,32 | 28311,26 | 45985,77 | 5704,47 | 35026,18 | 21786,28 | 37102,28 | 42237,11 | 43595,2 | 40179,42 | 21332,36 |
|  |  |  |  |  |  |  |  |  |  |  |  |  |  |
| **TP53_H179R_** | RERCSDSDG | 44223,27 | 46250,23 | 42614,91 | 44003,7 | 45599,31 | 33242 | 32933,05 | 29746,99 | 43127,47 | 21891,19 | 37839,73 | 29967,95 |
| **TP53_H179R_** | HRERCSDSD | 44454,02 | 47506,52 | 46822,21 | 45417,11 | 47160,84 | 42596,01 | 35958,92 | 26381,44 | 26638,44 | 42159,96 | 39619,51 | 44282,63 |
| **TP53_H179R_** | PHRERCSDS | 46393,56 | 48060,24 | 46336,39 | 45988,76 | 47597,6 | 42561,9 | 35956,18 | 42149,01 | 44117,16 | 48187,8 | 47142,99 | 45562,8 |
| **TP53_H179R_** | CPHRERCSD | 43074,3 | 45727,77 | 42144,9 | 45098,33 | 46189,21 | 10118,81 | 14687,98 | 36168,05 | 36145,36 | 45076,88 | 36294,27 | 38849,9 |
| **TP53_H179R_** | RCPHRERCS | 46556,49 | 45676,84 | 44997,93 | 46230,73 | 48269,72 | 42984,45 | 35879,62 | 43184,89 | 47355,13 | 47708,97 | 44265,87 | 45212,66 |
| **TP53_H179R_** | RRCPHRERC | 44630,4 | 40896,54 | 39131,71 | 38963,99 | 45225,88 | 33444,39 | 27011,97 | 1708,2 | 28667,6 | 38857,47 | 37251,12 | 40655,21 |
| **TP53_H179R_** | VRRCPHRER | 44444,88 | 42808,53 | 25940,17 | 41776,25 | 44006,56 | 30488,95 | 25067,72 | 1841,49 | 37295,47 | 42946,79 | 44131,5 | 37736,7 |
| **TP53_H179R_** | VVRRCPHRE | 42393,7 | 39838,32 | 22405,92 | 44156,81 | 43023,52 | 26258,13 | 24582,63 | 35219,23 | 46144,25 | 45269,44 | 34301,21 | 29913,54 |
| **TP53_H179R_** | EVVRRCPHR | 38579,73 | 38527,59 | 15931,82 | 41540,08 | 11545,3 | 38625,26 | 26194,28 | 31974,51 | 42913,81 | 44969,21 | 40875,76 | 37288,21 |
|  |  |  |  |  |  |  |  |  |  |  |  |  |  |
|  |  |  |  |  |  |  |  |  |  |  |  |  |  |
| **PROTEIN** | **PEPTIDE** | **A*01:01** | **A*02:01** | **A*03:01** | **A*24:02** | **A*26:02** | **B*07:02** | **B*08:02** | **B*27:05** | **B*39:01** | **B*40:01** | **B*58:01** | **B*15:01** |
| **FRGFR3_249wt_** | SPHRPILQA | 35958,92 | 34054,94 | 29391,54 | 42141,72 | 33774,59 | 149,94 | 5130,52 | 30552,03 | 13830,43 | 35561,93 | 34345,79 | 32608,26 |
| **FRGFR3_249wt_** | RSPHRPILQ | 30746,35 | 38420,6 | 19046,18 | 37635,57 | 41294,05 | 36349,3 | 31868,47 | 30577,5 | 43710,93 | 43735,07 | 23813,22 | 30915,14 |
| **FRGFR3_249wt_** | ERSPHRPIL | 38295,26 | 39312,92 | 38139,79 | 35325,33 | 30335,28 | 13440,67 | 3949,91 | 3004,38 | 383,23 | 23431,88 | 37692,62 | 30435,55 |
| **FRGFR3_249wt_** | LERSPHRPI | 36488,41 | 36770,58 | 33881,84 | 31648,57 | 35509,27 | 2934,85 | 4077,27 | 18432,09 | 15964,25 | 1257,24 | 32933,41 | 6180,66 |
| **FRGFR3_249wt_** | VLERSPHRP | 34524,63 | 31268,7 | 38696,8 | 41811,54 | 46880,01 | 42362,52 | 37176,62 | 42230,71 | 42798,81 | 42752,99 | 37385,56 | 39411,31 |
| **FRGFR3_249wt_** | DVLERSPHR | 38284,49 | 37963,98 | 18042,2 | 42259,05 | 15546,3 | 40420,14 | 27556,61 | 36741,54 | 42779,82 | 44928,86 | 43537,71 | 41056,15 |
| **FRGFR3_249wt_** | LDVLERSPH | 37005,67 | 44588,42 | 37719,96 | 46924,14 | 35747,1 | 34893,41 | 34684,13 | 37400,94 | 40329,71 | 37187,49 | 41929,75 | 26378,87 |
| **FRGFR3_249wt_** | TLDVLERSP | 33105,25 | 22450,08 | 38324,29 | 44862,32 | 46183,23 | 39541,15 | 35795,11 | 41839,13 | 37730,15 | 38591 | 37093,04 | 37533,09 |
| **FRGFR3_249wt_** | YTLDVLERS | 17953,79 | 5221,97 | 33786,29 | 38233,17 | 16988,09 | 37290,64 | 35249,73 | 30653,02 | 34473,86 | 36817,16 | 10970,25 | 26470,64 |
|  |  |  |  |  |  |  |  |  |  |  |  |  |  |
| **FRGFR3_S249C_** | CPHRPILQA | 33867,53 | 31415,2 | 30678,89 | 39968,71 | 32701,55 | 561,27 | 4395,83 | 24927,61 | 15737,54 | 35560,78 | 31392,44 | 31337,12 |
| **FRGFR3_S249C_** | RCPHRPILQ | 41553,1 | 41889,86 | 35121,43 | 42420,78 | 46664,43 | 41912,54 | 37337,05 | 39549,72 | 45767,36 | 45368,99 | 38364,52 | 41677,39 |
| **FRGFR3_S249C_** | ERCPHRPIL | 40624,87 | 39358,05 | 40229,46 | 34875,66 | 33954,85 | 23004,32 | 6904 | 2721,04 | 1554,29 | 27716 | 39568,55 | 32720,67 |
| **FRGFR3_S249C_** | LERCPHRPI | 34556,77 | 33980,22 | 32994,04 | 29196,62 | 34006,7 | 3872,64 | 3363,84 | 16950,63 | 13657,19 | 1209,64 | 30907,78 | 6339,69 |
| **FRGFR3_S249C_** | VLERCPHRP | 34667,25 | 27869,66 | 37089,84 | 41023,73 | 46654,83 | 42104,8 | 36832,69 | 39897,84 | 43783,35 | 43094,33 | 36868,98 | 40018,47 |
| **FRGFR3_S249C_** | DVLERCPHR | 39010,38 | 38223,23 | 22351,2 | 42246,7 | 21430,91 | 41763,59 | 28125,93 | 37843 | 43289,68 | 45697,61 | 44216,56 | 42059,27 |
| **FRGFR3_S249C_** | LDVLERCPH | 34685,63 | 44475,18 | 37721,59 | 46425,2 | 37538,37 | 36954,04 | 34151,6 | 37505,89 | 40250,36 | 37883,56 | 40274,76 | 27177,02 |
| **FRGFR3_S249C_** | TLDVLERCP | 31613,99 | 24895,52 | 38904,18 | 44791,5 | 46557,51 | 42250,38 | 37170,18 | 42962,13 | 40778,58 | 41290,03 | 37859,8 | 39920,31 |
| **FRGFR3_S249C_** | YTLDVLERC | 23680,38 | 1748,1 | 36587,63 | 31923 | 22362,82 | 37321,31 | 33305,01 | 29979,29 | 31179,17 | 34835,69 | 6578,75 | 29876 |
|  |  |  |  |  |  |  |  |  |  |  |  |  |  |
|  |  |  |  |  |  |  |  |  |  |  |  |  |  |
| **PROTEIN** | **PEPTIDE** | **A*01:01** | **A*02:01** | **A*03:01** | **A*24:02** | **A*26:02** | **B*07:02** | **B*08:02** | **B*27:05** | **B*39:01** | **B*40:01** | **B*58:01** | **B*15:01** |
| **GNA11_209wt_** | QRSERRKWI | 41940,65 | 42393,24 | 41044,61 | 39245,36 | 40848,78 | 25817,25 | 7188,87 | 1729,85 | 20411,79 | 38386,52 | 38894,07 | 37304,35 |
| **GNA11_209wt_** | GQRSERRKW | 41243,14 | 41967,44 | 36977,25 | 33026,18 | 38260,89 | 31870,2 | 26699,03 | 14374,64 | 41405,46 | 30515,35 | 13663,11 | 5329,05 |
| **GNA11_209wt_** | GGQRSERRK | 43329,06 | 45651,62 | 17386,39 | 45790,15 | 47579,06 | 44883,68 | 43060,77 | 32184,5 | 47788,51 | 46862,24 | 36819,56 | 41538,29 |
| **GNA11_209wt_** | VGGQRSERR | 40710,23 | 44029,9 | 34626,38 | 43650,42 | 46298,81 | 43829,32 | 36088,69 | 38304,79 | 45836,24 | 47334,64 | 40633,67 | 42185,07 |
| **GNA11_209wt_** | DVGGQRSER | 39586,95 | 44303,25 | 31043,52 | 45445,15 | 18996,37 | 38399 | 38538,43 | 42295,66 | 44592,77 | 46667,44 | 45790,15 | 43500,5 |
| **GNA11_209wt_** | VDVGGQRSE | 45009,62 | 45106,65 | 44395,86 | 47678 | 44377,6 | 41969,71 | 43223,23 | 43415,41 | 45322,87 | 42693,83 | 43073,35 | 42399,66 |
| **GNA11_209wt_** | MVDVGGQRS | 8956,23 | 28135,67 | 33928,41 | 43680,66 | 37571,28 | 28948,12 | 34155,68 | 37272,89 | 32786,93 | 38804,53 | 30722,41 | 34052,35 |
| **GNA11_209wt_** | RMVDVGGQR | 34722,8 | 15991,23 | 768,88 | 34947,82 | 24729,62 | 24839,57 | 33535 | 5055,19 | 35068,65 | 30727,4 | 27317,05 | 10098,8 |
| **GNA11_209wt_** | FRMVDVGGQ | 43235,86 | 39347,39 | 41085,93 | 44289,34 | 37012,06 | 38910,05 | 30614,24 | 2659,46 | 21669,44 | 40332,75 | 37297,49 | 33616,37 |
|  |  |  |  |  |  |  |  |  |  |  |  |  |  |
| **GNA11_Q209L_** | LRSERRKWI | 40263,86 | 41077,05 | 40815,2 | 38716,04 | 40667,54 | 21839,62 | 4523,16 | 1512,81 | 20295,3 | 38541,33 | 37890,94 | 35330,66 |
| **GNA11_Q209L_** | GLRSERRKW | 39977,35 | 39419,83 | 28654,26 | 34407,54 | 37290,64 | 27328,29 | 23355,18 | 27594,21 | 45270,43 | 39492,42 | 11628,8 | 11653,11 |
| **GNA11_Q209L_** | GGLRSERRK | 44310,42 | 44388,65 | 12184,98 | 45795,61 | 47417,69 | 44521,42 | 42659,65 | 28483,32 | 47210,35 | 46517,71 | 34268,58 | 41124,18 |
| **GNA11_Q209L_** | VGGLRSERR | 39318,88 | 42947,26 | 31928,87 | 42396,46 | 46239,22 | 43338,9 | 33431,37 | 36415,84 | 45107,61 | 47005,95 | 39986,87 | 42102,52 |
| **GNA11_Q209L_** | DVGGLRSER | 38000,16 | 41774,45 | 27521,75 | 43538,16 | 15355,38 | 36386,68 | 36586,83 | 38791,09 | 42737,71 | 45590,93 | 44220,87 | 41347,7 |
| **GNA11_Q209L_** | VDVGGLRSE | 44249,6 | 41850,9 | 43176 | 46313,32 | 39958,33 | 41347,7 | 41527,95 | 41996,06 | 43954,23 | 41048,61 | 41867,66 | 39741,45 |
| **GNA11_Q209L_** | MVDVGGLRS | 3773,25 | 21862,08 | 26883,68 | 41883,05 | 36471,42 | 27118,56 | 33004,04 | 35169,71 | 30124,96 | 37247,49 | 24866,45 | 31642,06 |
| **GNA11_Q209L_** | RMVDVGGLR | 29194,09 | 12128,68 | 474,58 | 29813,04 | 19589,37 | 25488,07 | 32010,17 | 3441,41 | 33594,91 | 28436,2 | 23076,87 | 8914,46 |
| **GNA11_Q209L_** | FRMVDVGGL | 33485,68 | 14356,45 | 37259,17 | 27555,14 | 22164,08 | 18486,82 | 5197,23 | 99,13 | 92,24 | 16113,33 | 28513,24 | 16842,76 |
|  |  |  |  |  |  |  |  |  |  |  |  |  |  |
| **GNA11_Q209P_** | PRSERRKWI | 44266,84 | 46724,03 | 45656,07 | 43463,82 | 45857,09 | 38206,7 | 24894,72 | 15658,72 | 37893,8 | 45809,47 | 44553,7 | 44095,23 |
| **GNA11_Q209P_** | GPRSERRKW | 41387,53 | 46419,18 | 39653,41 | 41926,59 | 37496,17 | 3661,28 | 22055,48 | 34217,83 | 43281,25 | 43137,26 | 20265,45 | 35499,65 |
| **GNA11_Q209P_** | GGPRSERRK | 44428,54 | 46151,25 | 27143,51 | 44979,45 | 46926,17 | 44997,46 | 44880,76 | 38873,89 | 47837,17 | 47283,45 | 40841,27 | 42160,86 |
| **GNA11_Q209P_** | VGGPRSERR | 39795,23 | 42937,48 | 30462,24 | 41485,73 | 45744,58 | 42951,44 | 36976,44 | 38682,55 | 45759,95 | 46985,62 | 39022,64 | 41527,49 |
| **GNA11_Q209P_** | DVGGPRSER | 38198,84 | 42313,97 | 23534,01 | 44648,77 | 14020,27 | 35981,86 | 38228,19 | 40496,75 | 43313,11 | 45467,29 | 45176,49 | 41514,02 |
| **GNA11_Q209P_** | VDVGGPRSE | 45253,8 | 45387,16 | 44602,89 | 47784,38 | 44860,85 | 42331,36 | 43134,93 | 43858,72 | 45371,45 | 42703,06 | 44288,39 | 42721,07 |
| **GNA11_Q209P_** | MVDVGGPRS | 9815,69 | 22808,78 | 31316,1 | 43128,39 | 36967,64 | 27847,35 | 34217,83 | 36385,88 | 32805,38 | 36489,59 | 28176,2 | 33637,47 |
| **GNA11_Q209P_** | RMVDVGGPR | 33288,09 | 10597,96 | 453,31 | 33284,11 | 20315,95 | 17805,21 | 27623,78 | 3854,41 | 28158,53 | 25913,8 | 25309,72 | 5224,51 |
| **GNA11_Q209P_** | FRMVDVGGP | 42785,84 | 36451,32 | 43551,84 | 41676,02 | 39416,42 | 37834,8 | 27348,99 | 6772,31 | 12733,28 | 32057,99 | 37452,38 | 34297,52 |
|  |  |  |  |  |  |  |  |  |  |  |  |  |  |
|  |  |  |  |  |  |  |  |  |  |  |  |  |  |
| **PROTEIN** | **PEPTIDE** | **A*01:01** | **A*02:01** | **A*03:01** | **A*24:02** | **A*26:02** | **B*07:02** | **B*08:02** | **B*27:05** | **B*39:01** | **B*40:01** | **B*58:01** | **B*15:01** |
| **HRAS_61wt_** | QEEYSAMRD | 42268,21 | 45863,54 | 45973,84 | 44706,27 | 46828,28 | 45525,85 | 43199,85 | 41958,35 | 41755,92 | 26205,9 | 40112,55 | 41942,46 |
| **HRAS_61wt_** | GQEEYSAMR | 31945,45 | 23530,71 | 17336,99 | 40609,05 | 37436,18 | 40808,15 | 38219,09 | 12844,54 | 33103,82 | 23805,48 | 40446,81 | 22307,48 |
| **HRAS_61wt_** | AGQEEYSAM | 33307,18 | 30092,73 | 36777,34 | 30021,82 | 27091,59 | 11957,08 | 13548,43 | 34369,21 | 17872,19 | 28859,61 | 28108,9 | 5107,53 |
| **HRAS_61wt_** | TAGQEEYSA | 34355,08 | 33281,6 | 43295,77 | 43100,39 | 43745,93 | 31811,64 | 25047,37 | 41887,13 | 26491,56 | 40488,86 | 20610,86 | 34880,94 |
| **HRAS_61wt_** | DTAGQEEYS | 28811,26 | 41179,38 | 42076,55 | 46052 | 24936,52 | 43302,8 | 42214,73 | 45154,5 | 42635,66 | 45928,09 | 36305,67 | 42861,37 |
| **HRAS_61wt_** | LDTAGQEEY | 9306,63 | 44050,86 | 40166,83 | 43545,23 | 30732,71 | 40227,29 | 42953,3 | 39806,43 | 41172,71 | 35378,11 | 26403,71 | 20855,83 |
| **HRAS_61wt_** | ILDTAGQEE | 20706,3 | 18298,36 | 33622,92 | 43376,9 | 45039,82 | 35958,13 | 37271,26 | 40307,01 | 36807,19 | 39477,88 | 38935,76 | 34364,01 |
| **HRAS_61wt_** | DILDTAGQE | 43543,36 | 40670,61 | 40810,78 | 47314,17 | 28796,61 | 42741,44 | 41050,83 | 44945,38 | 45121,78 | 46417,17 | 44771,62 | 41269,93 |
| **HRAS_61wt_** | LDILDTAGQ | 40934,17 | 42059,27 | 42682,27 | 47125,64 | 39741,45 | 43615,5 | 43308,89 | 40482,28 | 44013,71 | 41776,71 | 39993,37 | 37257,15 |
|  |  |  |  |  |  |  |  |  |  |  |  |  |  |
| **HRAS_Q61R_** | REEYSAMRD | 40787,84 | 44428,05 | 44043,25 | 42242,15 | 46460,9 | 43892,45 | 41308,36 | 34918,33 | 41401,86 | 15981,89 | 35877,68 | 40477,03 |
| **HRAS_Q61R_** | GREEYSAMR | 41668,36 | 38777,69 | 33260 | 42966,32 | 42930,06 | 42470,38 | 38897,86 | 1290,14 | 32951,23 | 37943,05 | 43798,04 | 41138,42 |
| **HRAS_Q61R_** | AGREEYSAM | 35396,87 | 31763,83 | 29377,88 | 33329,52 | 21194,54 | 1380,61 | 3402,28 | 30209,18 | 24625,76 | 30082,64 | 26587,19 | 1427,01 |
| **HRAS_Q61R_** | TAGREEYSA | 33193,14 | 33341,78 | 41971,99 | 41653,04 | 44235,7 | 30448,72 | 19697,34 | 40152,07 | 26946,58 | 41002,44 | 17746,94 | 35395,72 |
| **HRAS_Q61R_** | DTAGREEYS | 29316,9 | 40279,1 | 41080,15 | 45405,84 | 22697,99 | 41434,58 | 36957,63 | 44520,44 | 42318,99 | 46184,24 | 36886,13 | 42322,65 |
| **HRAS_Q61R_** | LDTAGREEY | 12404,47 | 44099,52 | 38222,82 | 42728,95 | 28881,79 | 35668,3 | 40005,49 | 37162,55 | 39550,99 | 34141,27 | 29464,78 | 17909,95 |
| **HRAS_Q61R_** | ILDTAGREE | 28030,84 | 20765,76 | 31130,63 | 44187,4 | 45246,44 | 33824,33 | 36545,68 | 39838,76 | 38327,18 | 40777,69 | 39622,53 | 32728,81 |
| **HRAS_Q61R_** | DILDTAGRE | 43218,07 | 41711,67 | 41048,16 | 47165,92 | 28824,66 | 43356,26 | 41426,51 | 44966,8 | 45851,14 | 47027,84 | 45010,61 | 41929,75 |
| **HRAS_Q61R_** | LDILDTAGR | 36687,53 | 38912,59 | 32183,11 | 44857,46 | 30899,43 | 42178,21 | 41570,2 | 36148,48 | 42146,26 | 41352,18 | 39392,96 | 39306,55 |
|  |  |  |  |  |  |  |  |  |  |  |  |  |  |
|  |  |  |  |  |  |  |  |  |  |  |  |  |  |
| **PROTEIN** | **PEPTIDE** | **A*01:01** | **A*02:01** | **A*03:01** | **A*24:02** | **A*26:02** | **B*07:02** | **B*08:02** | **B*27:05** | **B*39:01** | **B*40:01** | **B*58:01** | **B*15:01** |
| **PTEN_130wt_** | RTGVMICAY | 738,23 | 28052,38 | 1293,08 | 27221,45 | 10171,94 | 19104,17 | 23012,79 | 13065,57 | 35584,64 | 32306,26 | 236,42 | 97,64 |
| **PTEN_130wt_** | GRTGVMICA | 40212,91 | 31279,53 | 35774,57 | 41159,79 | 41001,12 | 37085,82 | 30953,64 | 1347,26 | 11619,99 | 30356,96 | 35858,67 | 36749,91 |
| **PTEN_130wt_** | KGRTGVMIC | 43506,61 | 34202,28 | 29937,16 | 38175,71 | 46566,07 | 20283,89 | 21473,38 | 31966,56 | 40360,25 | 40467,82 | 19245,46 | 24872,11 |
| **PTEN_130wt_** | GKGRTGVMI | 43077,11 | 39740,61 | 42503,01 | 39707,07 | 44495,87 | 36385,51 | 30740,7 | 14705,15 | 24025,43 | 26137,09 | 38024,82 | 31687,27 |
| **PTEN_130wt_** | AGKGRTGVM | 38260,47 | 39904,33 | 32533,92 | 38751,66 | 26132,86 | 2730,72 | 983,82 | 31482,92 | 31737,38 | 36031,74 | 34371,05 | 4416,23 |
| **PTEN_130wt_** | KAGKGRTGV | 38522,18 | 23799,05 | 31158,95 | 39410,04 | 41185,62 | 5618,84 | 18456,04 | 28164,3 | 35371,61 | 38706,43 | 16830,74 | 24047,27 |
| **PTEN_130wt_** | CKAGKGRTG | 45703,03 | 46642,71 | 44545,02 | 45536,69 | 44668,58 | 36717,71 | 23332,7 | 26723,02 | 35629,33 | 42910,11 | 42270,03 | 34772,06 |
| **PTEN_130wt_** | HCKAGKGRT | 45684,73 | 47104,21 | 44796,35 | 48241 | 46491,56 | 36994,45 | 32302,76 | 44342,1 | 46370,5 | 47410,5 | 43955,17 | 42910,11 |
| **PTEN_130wt_** | IHCKAGKGR | 43318,72 | 43127 | 32245,5 | 41692,71 | 43363,28 | 42184,14 | 42110,72 | 20163,31 | 37759,97 | 45064,2 | 41147,31 | 40092,14 |
|  |  |  |  |  |  |  |  |  |  |  |  |  |  |
| **PTEN_R130G_** | GTGVMICAY | 1068,69 | 30826,62 | 5259,73 | 37460,06 | 6790,72 | 32958,38 | 31883,99 | 25062,83 | 39509,08 | 34317,57 | 2946,05 | 366,99 |
| **PTEN_R130G_** | GGTGVMICA | 37961,12 | 20310,46 | 36258,17 | 42672,58 | 43919,04 | 37362,11 | 37028,89 | 36068,01 | 36908,9 | 38331,33 | 22886,65 | 32827,75 |
| **PTEN_R130G_** | KGGTGVMIC | 43894,8 | 35498,89 | 38314,33 | 38982,54 | 48048,28 | 40047,05 | 36789,27 | 39431,34 | 41725,2 | 42718,79 | 22038,31 | 37379,51 |
| **PTEN_R130G_** | GKGGTGVMI | 43815,1 | 37579,84 | 42956,54 | 41303,88 | 43597,11 | 37289,82 | 36776,93 | 19296,75 | 20682,12 | 24955,93 | 39324 | 30210,16 |
| **PTEN_R130G_** | AGKGGTGVM | 37850,38 | 37437,79 | 32913,11 | 38923,96 | 26261,82 | 2762,04 | 12286,13 | 33953,39 | 26676,79 | 31429,81 | 29091,29 | 2136,65 |
| **PTEN_R130G_** | KAGKGGTGV | 36612,58 | 17327,8 | 32202,61 | 39304,43 | 39945,37 | 11598,13 | 29347,7 | 29047,27 | 33722,75 | 35581,96 | 12830,78 | 18851,61 |
| **PTEN_R130G_** | CKAGKGGTG | 44685,98 | 46157,26 | 44496,84 | 45205,81 | 43270,95 | 36824,33 | 26804,39 | 27498,24 | 34764,52 | 41178,05 | 41141,98 | 32838,05 |
| **PTEN_R130G_** | HCKAGKGGT | 46116,32 | 46604,88 | 44837,56 | 48240,5 | 45875,46 | 35776,11 | 32600,86 | 44438,63 | 45806,51 | 47185,33 | 44017,52 | 42179,11 |
| **PTEN_R130G_** | IHCKAGKGG | 45569,24 | 45222,46 | 44238,1 | 43243,8 | 46808,54 | 42691,05 | 41087,7 | 34262,28 | 33787,38 | 44770,18 | 40650,39 | 39213,94 |
|  |  |  |  |  |  |  |  |  |  |  |  |  |  |
| **PTEN_R130Q_** | QTGVMICAY | 529,95 | 33143,6 | 8256,28 | 36156,32 | 2674,66 | 31881,24 | 27324,45 | 27480,69 | 36217,39 | 35350,94 | 2499,04 | 398,33 |
| **PTEN_R130Q_** | GQTGVMICA | 34259,3 | 2266,11 | 29525,73 | 37808,63 | 36734,39 | 36269,54 | 31883,32 | 15621,66 | 13080,14 | 11638,87 | 28892,11 | 6793,52 |
| **PTEN_R130Q_** | KGQTGVMIC | 42543,02 | 32583,94 | 36017,31 | 35912,24 | 47483,91 | 35929,36 | 31725,36 | 35857,12 | 38547,6 | 39974,34 | 19095,9 | 33360,19 |
| **PTEN_R130Q_** | GKGQTGVMI | 43771,04 | 38657,46 | 43290,62 | 40745,48 | 44268,75 | 37293,04 | 34111,72 | 20089,05 | 21515,94 | 23676,03 | 38870,93 | 29359,45 |
| **PTEN_R130Q_** | AGKGQTGVM | 37930,33 | 39955,73 | 34176,37 | 39771,99 | 28269,63 | 4613,96 | 9262,23 | 33675,35 | 31300,19 | 34692,38 | 31223,74 | 3673,3 |
| **PTEN_R130Q_** | KAGKGQTGV | 36955,66 | 17445,38 | 32216,9 | 40516,44 | 41507,73 | 21054,67 | 30770,65 | 32250,38 | 36894,91 | 38588,91 | 9527,93 | 26232,3 |
| **PTEN_R130Q_** | CKAGKGQTG | 45277,29 | 46268,75 | 44974,57 | 44866,69 | 44581,67 | 38212,07 | 24876,14 | 28712,6 | 31659,18 | 41446,23 | 40102,56 | 32324,79 |
| **PTEN_R130Q_** | HCKAGKGQT | 45905,73 | 46697,25 | 44314,74 | 48214,93 | 46150,74 | 33231,22 | 29381,69 | 44322,43 | 45491,39 | 46820,7 | 43211,08 | 41621,49 |
| **PTEN_R130Q_** | IHCKAGKGQ | 45473,18 | 45909,7 | 41733,79 | 45396,5 | 45919,65 | 43115,32 | 43235,86 | 34072,25 | 37354,45 | 45225,41 | 42921,7 | 41186,52 |
|  |  |  |  |  |  |  |  |  |  |  |  |  |  |
|  |  |  |  |  |  |  |  |  |  |  |  |  |  |
| **PROTEIN** | **PEPTIDE** | **A*01:01** | **A*02:01** | **A*03:01** | **A*24:02** | **A*26:02** | **B*07:02** | **B*08:02** | **B*27:05** | **B*39:01** | **B*40:01** | **B*58:01** | **B*15:01** |
| **AKT1_17wt_** | EYIKTWRPR | 40032,77 | 38103,91 | 21744,36 | 19097,14 | 17630,39 | 37029,3 | 28304,21 | 18132,41 | 32858,31 | 40787,84 | 38313,91 | 33975,44 |
| **AKT1_17wt_** | GEYIKTWRP | 42261,34 | 36231,5 | 40047,49 | 40669,3 | 44286 | 43233,52 | 31104,04 | 30091,41 | 32859,39 | 3752,44 | 38716,89 | 35885,46 |
| **AKT1_17wt_** | RGEYIKTWR | 37613,59 | 40008,95 | 15709,47 | 38376,57 | 44408,84 | 37218,87 | 35899,81 | 20533,84 | 43676,41 | 43432,77 | 31641,36 | 37592,85 |
| **AKT1_17wt_** | KRGEYIKTW | 43598,99 | 40176,83 | 39739,3 | 15949,58 | 40878,85 | 37587,16 | 30072,22 | 1442,93 | 31602,7 | 38639,04 | 5845,05 | 31988,68 |
| **AKT1_17wt_** | HKRGEYIKT | 46205,21 | 45533,75 | 42578,05 | 46378,52 | 43954,23 | 36306,84 | 28537,01 | 30285,1 | 33086,64 | 41810,62 | 44255,33 | 38295,68 |
| **AKT1_17wt_** | LHKRGEYIK | 40542,76 | 41662,04 | 22243,84 | 34126,13 | 43890,07 | 39500,12 | 32059,03 | 15131,9 | 28875,23 | 41559,4 | 35470,86 | 36013,04 |
| **AKT1_17wt_** | WLHKRGEYI | 23091,35 | 1023,7 | 26483,53 | 14709,45 | 22904,74 | 10068,68 | 75,55 | 13611,61 | 11392,17 | 24529,23 | 22719,12 | 4002,82 |
| **AKT1_17wt_** | GWLHKRGEY | 34301,96 | 43847,8 | 27046,48 | 27689,02 | 34638,37 | 38974,12 | 32499,43 | 26443,17 | 44759,04 | 41129,96 | 40242,07 | 16293,02 |
| **AKT1_17wt_** | EGWLHKRGE | 45940,03 | 44663,25 | 43192,83 | 45250,35 | 44459,79 | 41118,85 | 22244,81 | 38404,4 | 43511,32 | 46160,74 | 40353,7 | 43641,92 |
|  |  |  |  |  |  |  |  |  |  |  |  |  |  |
| **AKT1_E17K_** | KYIKTWRPR | 40227,29 | 31838,48 | 10608,06 | 11581,59 | 32196,69 | 32282,49 | 29106,09 | 6925,02 | 37733,02 | 41009,11 | 31937,52 | 30270,69 |
| **AKT1_E17K_** | GKYIKTWRP | 45806,51 | 41163,34 | 42157,21 | 43507,57 | 46727,58 | 45009,13 | 28260,46 | 18486,02 | 33158,31 | 34284,16 | 39722,54 | 41189,18 |
| **AKT1_E17K_** | RGKYIKTWR | 41365,16 | 39392,96 | 8579,48 | 36419,38 | 42458,88 | 33680,8 | 27614,52 | 13950,67 | 43348,26 | 43742,62 | 31277,84 | 31745,28 |
| **AKT1_E17K_** | KRGKYIKTW | 44073,28 | 41898,94 | 39916,84 | 17194,64 | 42484,62 | 37266,02 | 27370,31 | 1479,62 | 35963,58 | 41192,77 | 7499,6 | 33800,54 |
| **AKT1_E17K_** | HKRGKYIKT | 46186,23 | 46090,87 | 41718,43 | 46332,86 | 43847,36 | 32757,15 | 9831,1 | 25206,42 | 33709,61 | 43279,85 | 45377,33 | 39056 |
| **AKT1_E17K_** | LHKRGKYIK | 41343,24 | 42594,63 | 20570,32 | 33909,34 | 43637,67 | 36804,41 | 25528,63 | 12417,22 | 30150,07 | 42372,14 | 38103,91 | 36029,39 |
| **AKT1_E17K_** | WLHKRGKYI | 30311,99 | 8799,75 | 27066,69 | 24635,88 | 26877 | 7869,28 | 39,58 | 14807,65 | 20770,7 | 30641,08 | 31130,31 | 6700,3 |
| **AKT1_E17K_** | GWLHKRGKY | 36634,76 | 45068,1 | 29736,04 | 30227,81 | 36757,07 | 42030,61 | 35467,02 | 26871,47 | 45824,36 | 42960,72 | 40921,79 | 22200,56 |
| **AKT1_E17K_** | EGWLHKRGK | 42224,78 | 42983,5 | 19166,91 | 42746,51 | 38753,36 | 38588,91 | 25961,22 | 25042,22 | 42809,46 | 44764,38 | 36285,64 | 39564,26 |
|  |  |  |  |  |  |  |  |  |  |  |  |  |  |
|  |  |  |  |  |  |  |  |  |  |  |  |  |  |
| **PROTEIN** | **PEPTIDE** | **A*01:01** | **A*02:01** | **A*03:01** | **A*24:02** | **A*26:02** | **B*07:02** | **B*08:02** | **B*27:05** | **B*39:01** | **B*40:01** | **B*58:01** | **B*15:01** |
| **BCOR_1459wt_** | NKNAGETLL | 38181,89 | 36108,22 | 41133,96 | 39899,57 | 40058,75 | 28747,12 | 20014,65 | 20776,54 | 356,25 | 15971 | 34946,69 | 26030,69 |
| **BCOR_1459wt_** | VNKNAGETL | 39703,2 | 38357,05 | 40715,54 | 27870,26 | 37471,83 | 20696,01 | 12951,43 | 34001,55 | 16797,81 | 21282,79 | 29584,26 | 13373,65 |
| **BCOR_1459wt_** | IVNKNAGET | 34087,38 | 30710,44 | 34114,69 | 44376,17 | 35114,98 | 26264,38 | 30868,68 | 40106,47 | 40436,3 | 42350,14 | 31002,56 | 23647,6 |
| **BCOR_1459wt_** | LIVNKNAGE | 42254,93 | 27103,02 | 32815,32 | 46355,43 | 34324,98 | 34971,65 | 28468,22 | 39629,81 | 42578,05 | 44185,95 | 37507,51 | 24206,51 |
| **BCOR_1459wt_** | RLIVNKNAG | 41650,79 | 22237,83 | 29756,64 | 36697,85 | 42898,01 | 20151,97 | 10501,4 | 24676,69 | 32824,21 | 35857,51 | 22017,57 | 7649,25 |
| **BCOR_1459wt_** | RRLIVNKNA | 45069,09 | 37730,15 | 36175,89 | 39346,55 | 45484,5 | 30804,62 | 26072,7 | 245,18 | 24995,39 | 36023,56 | 36308,41 | 38676,29 |
| **BCOR_1459wt_** | ARRLIVNKN | 46008,66 | 46070,93 | 40314 | 44722,74 | 45597,84 | 40567,35 | 34058,26 | 4718,82 | 42273,25 | 44286,48 | 40587,97 | 38748,31 |
| **BCOR_1459wt_** | EARRLIVNK | 37184,27 | 41527,04 | 5117,99 | 42055,17 | 20594,14 | 31557,95 | 21851,44 | 27368,82 | 41898,48 | 42645,81 | 28148,78 | 30381,6 |
| **BCOR_1459wt_** | PEARRLIVN | 43772,45 | 47135,33 | 45386,66 | 44606,75 | 45549,99 | 45133,02 | 37682,83 | 39917,27 | 43943,29 | 38923,13 | 44209,86 | 42664,27 |
|  |  |  |  |  |  |  |  |  |  |  |  |  |  |
| **BCOR_N1459S_** | SKNAGETLL | 37803,71 | 32158,73 | 36866,98 | 38908,8 | 39373,38 | 25183,52 | 22294,45 | 15566,49 | 944,82 | 10563,96 | 30744,68 | 16544,03 |
| **BCOR_N1459S_** | VSKNAGETL | 32282,85 | 30507,1 | 35981,86 | 21893,32 | 33015,11 | 7034,37 | 13915,54 | 30856,99 | 19006,65 | 23210,34 | 4320,06 | 2629,53 |
| **BCOR_N1459S_** | IVSKNAGET | 34674 | 29656,05 | 35563,46 | 44488,67 | 36033,3 | 27308,77 | 33350,08 | 40517,77 | 40308,75 | 42323,11 | 29018,68 | 26491,56 |
| **BCOR_N1459S_** | LIVSKNAGE | 41708,96 | 26253,3 | 31221,71 | 45936,53 | 33667,68 | 33887,7 | 27617,51 | 39837,45 | 41824,19 | 43894,8 | 36477,75 | 23245,52 |
| **BCOR_N1459S_** | RLIVSKNAG | 41413,08 | 19528,2 | 25157,1 | 37445,47 | 42569,75 | 17572,49 | 13722,96 | 22513,09 | 32401,82 | 34799,16 | 20073,41 | 4462,2 |
| **BCOR_N1459S_** | RRLIVSKNA | 44848,23 | 37037,71 | 35005,34 | 38801,6 | 45198 | 29669,84 | 23408,06 | 253,71 | 23983,62 | 35813,3 | 35859,82 | 36953,65 |
| **BCOR_N1459S_** | ARRLIVSKN | 45830,8 | 46137,77 | 38970,73 | 45068,1 | 44899,69 | 38509,66 | 32435,5 | 3650,8 | 42553,18 | 44443,9 | 39966,99 | 37148,08 |
| **BCOR_N1459S_** | EARRLIVSK | 35946,46 | 40469,57 | 5210,23 | 41545,46 | 16746,28 | 22533,31 | 11924,91 | 22921,1 | 38112,98 | 40610,82 | 26555,26 | 24615,9 |
| **BCOR_N1459S_** | PEARRLIVS | 41902,56 | 45397,96 | 43709,97 | 43909,55 | 44171,62 | 42191,43 | 32194,94 | 39007,01 | 39080,94 | 29985,79 | 44741,61 | 40744,62 |
|  |  |  |  |  |  |  |  |  |  |  |  |  |  |
|  |  |  |  |  |  |  |  |  |  |  |  |  |  |
| **PROTEIN** | **PEPTIDE** | **A*01:01** | **A*02:01** | **A*03:01** | **A*24:02** | **A*26:02** | **B*07:02** | **B*08:02** | **B*27:05** | **B*39:01** | **B*40:01** | **B*58:01** | **B*15:01** |
| **CTNNB1_37wt_** | SGATTTAPS | 32525,47 | 25687,41 | 30291 | 37960,7 | 37901,6 | 17002,97 | 25780,96 | 33963,68 | 25584,49 | 36491,96 | 20864,41 | 17629,24 |
| **CTNNB1_37wt_** | HSGATTTAP | 32741,56 | 38749,99 | 36445,79 | 42955,62 | 40677,22 | 25240,81 | 30944,94 | 38475,1 | 31263,29 | 38187,29 | 12631,33 | 21829,23 |
| **CTNNB1_37wt_** | IHSGATTTA | 35129,79 | 32544,12 | 34723,92 | 32105,54 | 40646,86 | 24825,33 | 32325,14 | 27453,36 | 553,66 | 30239,92 | 30583,45 | 28230,2 |
| **CTNNB1_37wt_** | GIHSGATTT | 41480,33 | 24372,87 | 32775,94 | 45092,49 | 39338,46 | 23578,62 | 33042,99 | 38354,57 | 37323,33 | 38104,73 | 36456,04 | 17303,82 |
| **CTNNB1_37wt_** | SGIHSGATT | 38974,95 | 27889,57 | 38310,6 | 40545,85 | 38485,92 | 22571,87 | 29897,03 | 36958,85 | 27866,34 | 35863,71 | 27002,33 | 21305,84 |
| **CTNNB1_37wt_** | DSGIHSGAT | 33791,41 | 42708,62 | 42402,86 | 45004,75 | 33625,09 | 32447,43 | 20415,33 | 42039,25 | 36819,94 | 44716,91 | 38919,34 | 37522,12 |
| **CTNNB1_37wt_** | LDSGIHSGA | 32189,73 | 34425,03 | 37368,18 | 42206,97 | 35878,47 | 29931,65 | 29293,44 | 35226,84 | 34007,8 | 28855,87 | 33795,05 | 32525,11 |
| **CTNNB1_37wt_** | YLDSGIHSG | 10025,63 | 589,31 | 33045,12 | 28524,35 | 35634,34 | 24652,67 | 14998,9 | 28834,01 | 10263,03 | 29433,86 | 25273,32 | 15183,73 |
| **CTNNB1_37wt_** | SYLDSGIHS | 42222,49 | 31730,85 | 34347,66 | 17184,22 | 41225,75 | 38486,34 | 39521,47 | 34683,75 | 34874,91 | 40663,58 | 38734,07 | 34477,59 |
|  |  |  |  |  |  |  |  |  |  |  |  |  |  |
| **CTNNB1_S37F_** | FGATTTAPS | 28030,84 | 14789,88 | 31442,75 | 37164,16 | 32677,14 | 15460,41 | 17298,58 | 29029,67 | 15550 | 34150,13 | 18321,34 | 13749,71 |
| **CTNNB1_S37F_** | HFGATTTAP | 40912,47 | 36344,58 | 36609,39 | 31019,34 | 42520,94 | 27826,58 | 26125,5 | 37950,84 | 30568,57 | 39979,52 | 37146,07 | 29763,07 |
| **CTNNB1_S37F_** | IHFGATTTA | 35633,95 | 24205,99 | 29355,62 | 29282,04 | 38007,56 | 24478,85 | 29503,38 | 21727,9 | 383,76 | 25588,93 | 29490,62 | 22559,91 |
| **CTNNB1_S37F_** | GIHFGATTT | 39776,73 | 21696,19 | 31098,32 | 44067,57 | 39077,99 | 23946,28 | 32540,95 | 37695,08 | 36220,92 | 37478,71 | 34866,62 | 16829,64 |
| **CTNNB1_S37F_** | SGIHFGATT | 36816,35 | 19888,58 | 35249,34 | 36668,87 | 35473,54 | 24241,89 | 25684,06 | 32807,15 | 23222,65 | 33877,07 | 23191,25 | 18273,23 |
| **CTNNB1_S37F_** | DSGIHFGAT | 29075,57 | 38098,14 | 39161,37 | 41226,2 | 27000,87 | 31523,47 | 10938,37 | 39457,39 | 32331,44 | 43242,41 | 37483,98 | 34795,39 |
| **CTNNB1_S37F_** | LDSGIHFGA | 27570,64 | 24807,07 | 34361,04 | 36600,31 | 37677,95 | 32832,36 | 28758,62 | 30506,12 | 29392,49 | 22219,55 | 27895,61 | 33756,32 |
| **CTNNB1_S37F_** | YLDSGIHFG | 4337,2 | 223,97 | 28179,54 | 26147,28 | 34950,84 | 32292,63 | 22057,62 | 27506,58 | 19163,38 | 30705,13 | 23317,57 | 21135,47 |
| **CTNNB1_S37F_** | SYLDSGIHF | 34152,35 | 23546,25 | 30205,58 | 44,06 | 24053,26 | 26536,89 | 27547,07 | 19749,2 | 19610,36 | 27054,38 | 16511,66 | 6835,77 |
|  |  |  |  |  |  |  |  |  |  |  |  |  |  |
|  |  |  |  |  |  |  |  |  |  |  |  |  |  |
| **PROTEIN** | **PEPTIDE** | **A*01:01** | **A*02:01** | **A*03:01** | **A*24:02** | **A*26:02** | **B*07:02** | **B*08:02** | **B*27:05** | **B*39:01** | **B*40:01** | **B*58:01** | **B*15:01** |
| **EGFR_858wt_** | LAKLLGAEE | 40973,18 | 37788,18 | 36233,07 | 45213,16 | 39817,21 | 25980,62 | 14815,67 | 36050,84 | 39632,39 | 40877,98 | 26836,32 | 19496,96 |
| **EGFR_858wt_** | GLAKLLGAE | 39301,44 | 12523,82 | 25165,81 | 43392,84 | 34170,09 | 30920,84 | 28382,72 | 30720,74 | 39184,69 | 37494,94 | 37445,09 | 12317,93 |
| **EGFR_858wt_** | FGLAKLLGA | 30679,56 | 2240,82 | 28571,61 | 34376,27 | 37508,75 | 25124,46 | 2320,27 | 19513,84 | 19781,92 | 35927,79 | 18931,53 | 20913,68 |
| **EGFR_858wt_** | DFGLAKLLG | 39720,39 | 41818,77 | 40929,31 | 26906,09 | 43484,03 | 43518,4 | 28673,8 | 39887,91 | 41957 | 46503,14 | 40358,07 | 41822,84 |
| **EGFR_858wt_** | TDFGLAKLL | 32604,04 | 28508 | 36668,47 | 28922,76 | 29085,62 | 26885,43 | 25970,21 | 29841,76 | 21491,76 | 4644,76 | 29215,88 | 27906,48 |
| **EGFR_858wt_** | ITDFGLAKL | 1919,38 | 2077,56 | 27689,31 | 21168,43 | 24679,64 | 15364,53 | 25842,12 | 31102,7 | 13895,53 | 22468,07 | 6689,29 | 22985,92 |
| **EGFR_858wt_** | KITDFGLAK | 27224,7 | 19690,52 | 31,68 | 37442,23 | 34662,73 | 25403,55 | 37308,39 | 14502,27 | 39272,55 | 37522,54 | 24009,32 | 18459,04 |
| **EGFR_858wt_** | VKITDFGLA | 39895,68 | 33973,6 | 39513,36 | 41526,15 | 37324,12 | 36967,24 | 29279,18 | 21316,67 | 21437,63 | 28286,46 | 36139,49 | 26825,57 |
| **EGFR_858wt_** | HVKITDFGL | 33792,87 | 10019,45 | 28408,53 | 30739,03 | 14326,66 | 8927,21 | 5417,36 | 22193,84 | 14434,02 | 26484,12 | 15135,5 | 5665,36 |
|  |  |  |  |  |  |  |  |  |  |  |  |  |  |
| **EGFR_L858R_** | RAKLLGAEE | 41908,45 | 37143,24 | 28619,87 | 43145,66 | 42222,49 | 13743,32 | 15030,41 | 26807,59 | 39295,07 | 38185,61 | 21275,19 | 16722,56 |
| **EGFR_L858R_** | GRAKLLGAE | 44863,28 | 43322,94 | 39880,14 | 44412,18 | 41186,52 | 36208,77 | 30233,7 | 3030,46 | 31603,74 | 37388,81 | 41383,52 | 36214,66 |
| **EGFR_L858R_** | FGRAKLLGA | 34417,21 | 16762,77 | 28212,18 | 38065,57 | 34378,88 | 12390,79 | 364,53 | 21331,21 | 29141,38 | 39419,83 | 29493,16 | 14143,99 |
| **EGFR_L858R_** | DFGRAKLLG | 39886,19 | 43838,82 | 40624,45 | 26885,13 | 43968,97 | 42847 | 28277,29 | 38767,61 | 41985,61 | 46816,66 | 39841,77 | 42024,23 |
| **EGFR_L858R_** | TDFGRAKLL | 31695,5 | 31912,65 | 35511,95 | 30829,3 | 28449,76 | 25858,06 | 12850,09 | 31227,79 | 22385,33 | 11027,37 | 32307,3 | 30953,3 |
| **EGFR_L858R_** | ITDFGRAKL | 3945,21 | 7343,91 | 27220,57 | 24950,27 | 27623,5 | 4188,88 | 17991,51 | 29435,13 | 10639,09 | 22539,9 | 9686,55 | 23046,42 |
| **EGFR_L858R_** | KITDFGRAK | 37166,17 | 28344,68 | 72,17 | 41075,7 | 36202,89 | 23573,78 | 36624,86 | 19028,25 | 41784,39 | 39711,38 | 30819,29 | 19714,81 |
| **EGFR_L858R_** | VKITDFGRA | 43035,18 | 39191,89 | 42154,04 | 43898,62 | 39345,26 | 39079,25 | 31675,62 | 25024,07 | 29118,69 | 35273,77 | 40245,58 | 31800,62 |
| **EGFR_L858R_** | HVKITDFGR | 37367,77 | 34242,27 | 10232,76 | 39531,74 | 19883,63 | 34948,56 | 31594,16 | 23446,09 | 40285,2 | 41989,23 | 33976,91 | 31164,68 |
|  |  |  |  |  |  |  |  |  |  |  |  |  |  |
|  |  |  |  |  |  |  |  |  |  |  |  |  |  |
| **PROTEIN** | **PEPTIDE** | **A*01:01** | **A*02:01** | **A*03:01** | **A*24:02** | **A*26:02** | **B*07:02** | **B*08:02** | **B*27:05** | **B*39:01** | **B*40:01** | **B*58:01** | **B*15:01** |
| **ERBB2_310wt_** | SCTLVCPLH | 33705,59 | 36913,68 | 27103,89 | 43693,91 | 41579,64 | 40085,65 | 39124,52 | 37084,22 | 42634,73 | 40059,19 | 24338,88 | 31426,75 |
| **ERBB2_310wt_** | GSCTLVCPL | 18980,34 | 3911,5 | 19873,51 | 24767,38 | 28678,75 | 16868,29 | 13928,95 | 15184,56 | 11315,15 | 17698,81 | 1563,56 | 2537,6 |
| **ERBB2_310wt_** | VGSCTLVCP | 39187,64 | 32552,92 | 40071,33 | 39837,04 | 46553,49 | 40900,07 | 37699,97 | 40558,11 | 40098,66 | 41252,98 | 20547,84 | 34988,68 |
| **ERBB2_310wt_** | DVGSCTLVC | 35059,15 | 33881,09 | 40074,36 | 42324,51 | 36281,73 | 34724,3 | 27942,12 | 42555,92 | 35172,77 | 43886,73 | 35664,82 | 35175,81 |
| **ERBB2_310wt_** | TDVGSCTLV | 26217,82 | 21956,42 | 37129,59 | 33971,04 | 27233,23 | 29807,56 | 31939,93 | 35921,18 | 25482 | 18906,56 | 33269,35 | 31392,44 |
| **ERBB2_310wt_** | STDVGSCTL | 1044,42 | 5162,7 | 27079,87 | 27445,04 | 25001,34 | 7346,93 | 16461,36 | 29632,95 | 2232,74 | 12675,42 | 7057,47 | 16153,65 |
| **ERBB2_310wt_** | LSTDVGSCT | 23872,54 | 31873,98 | 38082,89 | 44472,77 | 36811,58 | 32615,33 | 34177,49 | 37585,92 | 35748,27 | 40530,06 | 14332,09 | 25640,21 |
| **ERBB2_310wt_** | YLSTDVGSC | 32286,35 | 1512,51 | 35657,88 | 38995,19 | 33978,01 | 25454,72 | 18499,82 | 33922,92 | 22235,91 | 34304,55 | 31079,82 | 11548,92 |
| **ERBB2_310wt_** | NYLSTDVGS | 41775,8 | 34385,95 | 39789,64 | 24748,89 | 42848,85 | 40033,62 | 38201,32 | 36570,2 | 34191,91 | 43481,2 | 38765,93 | 40150,75 |
|  |  |  |  |  |  |  |  |  |  |  |  |  |  |
| **ERBB2_S310F_** | FCTLVCPLH | 28711,68 | 31922,65 | 31981,07 | 43512,28 | 38582,24 | 38950,07 | 33516,49 | 36599,11 | 39782,32 | 39006,58 | 25247,9 | 27638,73 |
| **ERBB2_S310F_** | GFCTLVCPL | 31954,46 | 6856,28 | 25782,36 | 9148,78 | 34373,67 | 23636,09 | 9963,99 | 18072,09 | 14824,33 | 21109,88 | 26009,3 | 9274,76 |
| **ERBB2_S310F_** | VGFCTLVCP | 40104,73 | 24307,57 | 36175,89 | 37762,84 | 45698,08 | 40339,72 | 33815,91 | 38168,28 | 37433,33 | 38954,72 | 22556,01 | 29220,31 |
| **ERBB2_S310F_** | DVGFCTLVC | 34547,04 | 31368,68 | 39568,55 | 41626 | 36888,12 | 36343,01 | 27058,78 | 43040,28 | 35932,84 | 44056,6 | 36450,12 | 35622,78 |
| **ERBB2_S310F_** | TDVGFCTLV | 21801,85 | 11766,11 | 33990,52 | 28680,94 | 22238,07 | 30757,67 | 26521,96 | 32218,64 | 20991,44 | 13551,8 | 29333,09 | 28753,33 |
| **ERBB2_S310F_** | STDVGFCTL | 897,38 | 3039,46 | 24782,38 | 17056,23 | 20708,32 | 7754,66 | 11262,26 | 26322,13 | 2185,42 | 12399,37 | 6577,54 | 13219,15 |
| **ERBB2_S310F_** | LSTDVGFCT | 16790,37 | 19182,25 | 35248,95 | 40280,87 | 38522,18 | 35973,69 | 34186,36 | 33249,21 | 30148,44 | 37802,49 | 5792,29 | 27575,12 |
| **ERBB2_S310F_** | YLSTDVGFC | 26040,28 | 711,54 | 31883,99 | 37566 | 34751,36 | 34716,78 | 27169,08 | 34118,01 | 31391,75 | 35031,11 | 28467,91 | 17709,16 |
| **ERBB2_S310F_** | NYLSTDVGF | 34813,07 | 24227,22 | 35086,48 | 111,34 | 24542,49 | 30351,37 | 26710,01 | 21777,79 | 16580,41 | 30961,01 | 18017,04 | 15065,09 |
|  |  |  |  |  |  |  |  |  |  |  |  |  |  |
|  |  |  |  |  |  |  |  |  |  |  |  |  |  |
| **PROTEIN** | **PEPTIDE** | **A*01:01** | **A*02:01** | **A*03:01** | **A*24:02** | **A*26:02** | **B*07:02** | **B*08:02** | **B*27:05** | **B*39:01** | **B*40:01** | **B*58:01** | **B*15:01** |
| **ERBB3_104wt_** | VVRGTQVYD | 41894,86 | 35418,31 | 31089,24 | 43337,5 | 41784,39 | 30292,31 | 36299,4 | 40114,72 | 45159,39 | 43705,71 | 18459,63 | 24769,52 |
| **ERBB3_104wt_** | RVVRGTQVY | 10326,97 | 30672,6 | 1124,64 | 27620,5 | 3764,07 | 10454,12 | 27289,88 | 13340,85 | 28536,38 | 26377,45 | 460,74 | 24 |
| **ERBB3_104wt_** | LRVVRGTQV | 39310,79 | 36119,17 | 37759,56 | 39007,01 | 36039,52 | 23457,49 | 3560,87 | 701,19 | 8341,67 | 34773,18 | 38611,04 | 27888,36 |
| **ERBB3_104wt_** | NLRVVRGTQ | 42048,34 | 37826,63 | 24478,58 | 44519,95 | 35361,64 | 17461,24 | 10100,65 | 32521,95 | 41699,03 | 43232,12 | 41539,17 | 18323,92 |
| **ERBB3_104wt_** | PNLRVVRGT | 45345,94 | 45237,63 | 43824,57 | 45732,22 | 46560,51 | 46008,16 | 39885,78 | 39865,93 | 45133,02 | 47326,46 | 42832,63 | 45539,15 |
| **ERBB3_104wt_** | LPNLRVVRG | 40905,4 | 41635,02 | 38852,01 | 42092,5 | 43471,33 | 6243,24 | 3036,93 | 33327,72 | 33771,67 | 43484,03 | 31093,27 | 36443,42 |
| **ERBB3_104wt_** | PLPNLRVVR | 39034,89 | 35278,72 | 20248,79 | 37520,09 | 36501,82 | 40665,34 | 37847,11 | 36173,14 | 44346,41 | 45104,7 | 44423,24 | 37528,64 |
| **ERBB3_104wt_** | LPLPNLRVV | 37883,98 | 19090,12 | 34645,86 | 33007,25 | 32136,49 | 204,58 | 3069,8 | 29730,25 | 5191,04 | 32084,36 | 23219,62 | 28965,04 |
| **ERBB3_104wt_** | TLPLPNLRV | 23851,12 | 562,36 | 25035,73 | 13903,5 | 24094,42 | 27908,58 | 25590,58 | 32059,03 | 29010,2 | 35132,44 | 35543,47 | 23519,51 |
|  |  |  |  |  |  |  |  |  |  |  |  |  |  |
| **ERBB3_V104M_** | MVRGTQVYD | 37843,84 | 27701,01 | 25896,71 | 40290,87 | 32132,3 | 22024,71 | 24707,43 | 34828,14 | 40182,92 | 39615,25 | 10624,48 | 15517,39 |
| **ERBB3_V104M_** | RMVRGTQVY | 8985,44 | 17179,77 | 305,54 | 13934,83 | 11096,56 | 14600,2 | 19161,52 | 4636,22 | 17751,17 | 16665,84 | 707,33 | 6,05 |
| **ERBB3_V104M_** | LRMVRGTQV | 34473,86 | 26898,82 | 31419,96 | 29569,84 | 34342,8 | 11777,2 | 380,16 | 107,28 | 3004,54 | 29880,85 | 34116,89 | 20989,17 |
| **ERBB3_V104M_** | NLRMVRGTQ | 41344,57 | 36545,68 | 22839,9 | 43811,32 | 32889,62 | 15120,45 | 7093,91 | 31010,28 | 40463,43 | 42360,69 | 41453,43 | 15378,32 |
| **ERBB3_V104M_** | PNLRMVRGT | 45165,74 | 45571,68 | 44263,47 | 45369,48 | 46851,09 | 45797,09 | 37714,66 | 38453,05 | 45291,49 | 47303,91 | 43554,67 | 45722,81 |
| **ERBB3_V104M_** | LPNLRMVRG | 39954 | 41391,55 | 38141,02 | 42668,43 | 42053,81 | 5670,51 | 3800,04 | 33109,55 | 34422,79 | 43869,64 | 30806,29 | 36244,45 |
| **ERBB3_V104M_** | PLPNLRMVR | 36204,85 | 33943,47 | 15452,55 | 35493,51 | 35425,98 | 39617,8 | 36068,8 | 33594,91 | 44300,37 | 45176,49 | 43685,4 | 36200,93 |
| **ERBB3_V104M_** | LPLPNLRMV | 37774,27 | 17450,09 | 35164,76 | 32414,45 | 31371,04 | 705,58 | 6071,23 | 29973,15 | 11805,52 | 35203,97 | 25385,69 | 35196,37 |
| **ERBB3_V104M_** | TLPLPNLRM | 22083,41 | 5058,8 | 22979,46 | 11730,66 | 10011,65 | 18424,32 | 21062,42 | 30437,2 | 21773,32 | 29272,54 | 30590,73 | 6668,62 |
|  |  |  |  |  |  |  |  |  |  |  |  |  |  |
|  |  |  |  |  |  |  |  |  |  |  |  |  |  |
| **PROTEIN** | **PEPTIDE** | **A*01:01** | **A*02:01** | **A*03:01** | **A*24:02** | **A*26:02** | **B*07:02** | **B*08:02** | **B*27:05** | **B*39:01** | **B*40:01** | **B*58:01** | **B*15:01** |
| **FBXW7_465wt_** | RCMHLHEKR | 39475,31 | 36951,65 | 17102,06 | 35761,81 | 41748,25 | 35102,43 | 33218,64 | 15078,13 | 40725,65 | 41388,43 | 24638,02 | 37333,01 |
| **FBXW7_465wt_** | VRCMHLHEK | 39805,58 | 36206,42 | 15458,74 | 36801,23 | 41523,9 | 39474,04 | 28369,22 | 282,41 | 32958,38 | 41216,84 | 37771,83 | 35227,98 |
| **FBXW7_465wt_** | TVRCMHLHE | 36562,3 | 34939,5 | 22007,32 | 40758,28 | 34621,13 | 22116,42 | 12513,25 | 32771,33 | 42150,83 | 42518,2 | 29225,7 | 18686,71 |
| **FBXW7_465wt_** | STVRCMHLH | 11758,1 | 27526,52 | 4146,18 | 37843 | 3485,74 | 28925,57 | 33076,62 | 19675,18 | 34865,85 | 35205,88 | 5576,14 | 8720,52 |
| **FBXW7_465wt_** | TSTVRCMHL | 16922,23 | 21208,08 | 28285,24 | 22096,8 | 29773,38 | 16447,83 | 1150,32 | 25362,63 | 20938,57 | 33397,39 | 5237,08 | 13520,31 |
| **FBXW7_465wt_** | HTSTVRCMH | 13001,42 | 38518 | 6579,67 | 42911,02 | 8957,68 | 21959,99 | 27093,36 | 26605,32 | 33610,17 | 38650,33 | 10282,82 | 9214,75 |
| **FBXW7_465wt_** | GHTSTVRCM | 36908,9 | 35578,1 | 37395,27 | 32788,71 | 35826,1 | 26809,61 | 27296,68 | 20292,45 | 1440,06 | 27208,8 | 32549,05 | 22310,12 |
| **FBXW7_465wt_** | YGHTSTVRC | 38483,84 | 31681,79 | 38699,29 | 36946,05 | 44464,6 | 31204,82 | 20358,86 | 33726,03 | 25929,78 | 39052,62 | 21959,28 | 28322,6 |
| **FBXW7_465wt_** | LYGHTSTVR | 39080,51 | 39663,69 | 24828,28 | 20844,1 | 39895,27 | 38325,1 | 33236,26 | 29445,65 | 41869,47 | 44062,31 | 39526,18 | 36930,07 |
|  |  |  |  |  |  |  |  |  |  |  |  |  |  |
| **FBXW7_R465C_** | CCMHLHEKR | 35583,1 | 36207,21 | 25653,52 | 37533,09 | 38801,6 | 38910,05 | 32005,32 | 25575,09 | 40835,08 | 43350,61 | 30585,43 | 40622,25 |
| **FBXW7_R465C_** | VCCMHLHEK | 35407,98 | 36283,67 | 10517,88 | 40887,71 | 44208,92 | 42319,46 | 35375,8 | 35136,63 | 43855,41 | 44711,12 | 30260,54 | 38495,08 |
| **FBXW7_R465C_** | TVCCMHLHE | 30108,02 | 28732,5 | 24660,69 | 38460,93 | 37905,29 | 38073,82 | 29104,52 | 36181,75 | 40529,61 | 42643,5 | 23037,95 | 26020,29 |
| **FBXW7_R465C_** | STVCCMHLH | 9340,02 | 22250,82 | 4643,56 | 37862,66 | 3032,83 | 30813,28 | 34574,34 | 22532,59 | 33830,18 | 32534,97 | 6496,69 | 8577,07 |
| **FBXW7_R465C_** | TSTVCCMHL | 11893,21 | 10589,48 | 24202,59 | 18230,58 | 29834,66 | 20603,51 | 13452,75 | 23689,09 | 18406,39 | 28376,59 | 1304,59 | 10868,65 |
| **FBXW7_R465C_** | HTSTVCCMH | 8436,97 | 32849,43 | 7189,5 | 41046,39 | 8641,06 | 30289,38 | 31384,62 | 27403,79 | 34075,57 | 36757,85 | 8025,95 | 7062,28 |
| **FBXW7_R465C_** | GHTSTVCCM | 31850,89 | 29913,21 | 34448,88 | 28576,86 | 33465,75 | 28045,4 | 26694,71 | 20289,81 | 644,91 | 23689,35 | 29139,19 | 18476,41 |
| **FBXW7_R465C_** | YGHTSTVCC | 38159,2 | 31814,73 | 40010,24 | 37886,84 | 45544,09 | 29874,07 | 15344,42 | 36135,2 | 22027,1 | 38738,67 | 24517,56 | 26929,1 |
| **FBXW7_R465C_** | LYGHTSTVC | 40927,54 | 36490,76 | 40361,14 | 13622,21 | 45358,69 | 32135,78 | 26562,16 | 38602,28 | 31227,45 | 41661,16 | 36822,74 | 33297,45 |
|  |  |  |  |  |  |  |  |  |  |  |  |  |  |
| **FBXW7_R465H_** | HCMHLHEKR | 37547,32 | 37495,76 | 25426,65 | 39012,92 | 34318,67 | 35998,22 | 31028,07 | 23405,27 | 36141,45 | 41173,14 | 29997,47 | 38733,65 |
| **FBXW7_R465H_** | VHCMHLHEK | 34408,27 | 35923,13 | 14435,42 | 27871,77 | 40239,04 | 38197,61 | 32575,47 | 14010,11 | 26272,92 | 39879,73 | 33108,84 | 34647 |
| **FBXW7_R465H_** | TVHCMHLHE | 34037,62 | 31984,89 | 26673,04 | 39226,25 | 34168,23 | 32622,04 | 20383,33 | 33816,28 | 38555,95 | 41020,64 | 25827,31 | 22242,88 |
| **FBXW7_R465H_** | STVHCMHLH | 11871,62 | 25951,39 | 4541,59 | 39545,87 | 2929,24 | 31372,06 | 36517,62 | 24414,84 | 36221,32 | 35346,73 | 8632,65 | 10433,67 |
| **FBXW7_R465H_** | TSTVHCMHL | 13307,41 | 17908,01 | 26948,04 | 19503,08 | 25350,82 | 16911,97 | 4298,7 | 24972,14 | 15394,31 | 28005,08 | 2824,69 | 9234,71 |
| **FBXW7_R465H_** | HTSTVHCMH | 7964,87 | 35316,54 | 5422,93 | 40696,14 | 6470,04 | 26420,01 | 27573,91 | 26363,75 | 31343,56 | 35343,29 | 5647,05 | 7062,43 |
| **FBXW7_R465H_** | GHTSTVHCM | 31928,17 | 29532,12 | 35924,68 | 25137,51 | 34029,14 | 26683,72 | 25381,84 | 20021,36 | 245,96 | 21251,5 | 29316,9 | 21286,94 |
| **FBXW7_R465H_** | YGHTSTVHC | 39503,1 | 30079,38 | 38758,81 | 36868,57 | 44624,61 | 29612,43 | 18487,41 | 34520,13 | 24917,9 | 38461,78 | 21664,04 | 22578,22 |
| **FBXW7_R465H_** | LYGHTSTVH | 36884,54 | 44653,11 | 29732,82 | 28134,76 | 39412,15 | 37558,68 | 34680,37 | 34607,27 | 38894,07 | 42627,36 | 37560,72 | 19952,38 |
|  |  |  |  |  |  |  |  |  |  |  |  |  |  |
|  |  |  |  |  |  |  |  |  |  |  |  |  |  |
| **PROTEIN** | **PEPTIDE** | **A*01:01** | **A*02:01** | **A*03:01** | **A*24:02** | **A*26:02** | **B*07:02** | **B*08:02** | **B*27:05** | **B*39:01** | **B*40:01** | **B*58:01** | **B*15:01** |
| **FBXW7_479wt_** | RDATLRVWD | 40206,41 | 42836,34 | 40645,99 | 41896,67 | 45234,68 | 42149,47 | 35950,34 | 32109,72 | 43105,53 | 34645,12 | 34724,68 | 42189,14 |
| **FBXW7_479wt_** | SRDATLRVW | 34381,85 | 39992,07 | 38848,65 | 20091,01 | 36195,47 | 30612,24 | 30185,01 | 4338,51 | 11593,37 | 29062,97 | 6819,66 | 28521,57 |
| **FBXW7_479wt_** | GSRDATLRV | 23922,97 | 22636,93 | 23355,69 | 35577,71 | 29787,56 | 13320,23 | 24778,37 | 26629,5 | 37871,28 | 34451,48 | 9892,13 | 13744,5 |
| **FBXW7_479wt_** | SGSRDATLR | 33273,68 | 40005,05 | 19628,62 | 41687,77 | 40868,22 | 39822,37 | 39512,93 | 31951,68 | 43187,23 | 43824,12 | 27636,34 | 36112,53 |
| **FBXW7_479wt_** | VSGSRDATL | 28690,87 | 25439,29 | 37424,01 | 25437,93 | 39272,55 | 12793,49 | 6719,25 | 35065,23 | 24387,91 | 33786,29 | 6218,36 | 13314,32 |
| **FBXW7_479wt_** | VVSGSRDAT | 37575,76 | 33155,45 | 35569,25 | 44577,32 | 38357,47 | 14686,07 | 32463,94 | 39505,23 | 32804,68 | 40602,47 | 31137,38 | 26470,64 |
| **FBXW7_479wt_** | RVVSGSRDA | 38347,11 | 23009,31 | 22928,04 | 41797,52 | 32062,15 | 8132,77 | 28394,71 | 28013,88 | 35100,91 | 34199,67 | 22626,39 | 11484,13 |
| **FBXW7_479wt_** | KRVVSGSRD | 46875,45 | 46382,04 | 44030,86 | 46006,17 | 46836,41 | 42359,32 | 40847,46 | 9867,65 | 42091,12 | 42995,62 | 37928,67 | 41801,13 |
| **FBXW7_479wt_** | EKRVVSGSR | 45054,46 | 45610,16 | 34484,31 | 46187,21 | 37439 | 35575,79 | 27285,75 | 21972,12 | 38643,65 | 42902,19 | 44909,42 | 36556,36 |
|  |  |  |  |  |  |  |  |  |  |  |  |  |  |
| **FBXW7_R479Q_** | QDATLRVWD | 41726,55 | 44599,04 | 43649,49 | 44712,1 | 45694,13 | 44055,15 | 39889,64 | 40230,32 | 44090,45 | 40168,57 | 38468,43 | 43516,04 |
| **FBXW7_R479Q_** | SQDATLRVW | 22360,39 | 22517,23 | 34470,12 | 11370,01 | 31198,42 | 27242,09 | 30772,32 | 17440,28 | 12545,11 | 9696,93 | 613,41 | 3203,39 |
| **FBXW7_R479Q_** | GSQDATLRV | 11618,11 | 10043,77 | 26624,9 | 32029,91 | 32386,4 | 31173,44 | 35240,18 | 26736,33 | 31984,54 | 31177,48 | 7885,13 | 21963,08 |
| **FBXW7_R479Q_** | SGSQDATLR | 34329,8 | 40706,27 | 23898,65 | 43168,54 | 40418,81 | 40301,79 | 41644,02 | 35978,36 | 43396,59 | 43840,71 | 29439,29 | 36297,8 |
| **FBXW7_R479Q_** | VSGSQDATL | 27059,95 | 25006,21 | 39702,77 | 27193,78 | 39665,43 | 21918,69 | 25352,2 | 37070,97 | 24207,3 | 32218,29 | 4320,15 | 15853,76 |
| **FBXW7_R479Q_** | VVSGSQDAT | 35037,54 | 30761,66 | 37318,88 | 44999,41 | 38463,86 | 29598,65 | 39195,28 | 41044,61 | 36444,99 | 40560,31 | 27325,03 | 27966,92 |
| **FBXW7_R479Q_** | RVVSGSQDA | 35653,62 | 20238,94 | 25869,25 | 40219,89 | 31747,32 | 11843,91 | 30976,08 | 30620,85 | 32744,04 | 33295,64 | 20347,63 | 10009,91 |
| **FBXW7_R479Q_** | KRVVSGSQD | 47050,23 | 46085,4 | 43958,03 | 45878,92 | 46880,5 | 41570,2 | 40620,49 | 10838,47 | 40745,48 | 42387,74 | 37447,91 | 41280,66 |
| **FBXW7_R479Q_** | EKRVVSGSQ | 47020,72 | 47225,18 | 43531,11 | 47988,02 | 42485,09 | 35828,8 | 28465,45 | 33693,55 | 40368,12 | 43527,35 | 44895,81 | 35274,52 |
|  |  |  |  |  |  |  |  |  |  |  |  |  |  |
|  |  |  |  |  |  |  |  |  |  |  |  |  |  |
| **PROTEIN** | **PEPTIDE** | **A*01:01** | **A*02:01** | **A*03:01** | **A*24:02** | **A*26:02** | **B*07:02** | **B*08:02** | **B*27:05** | **B*39:01** | **B*40:01** | **B*58:01** | **B*15:01** |
| **FBXW7_505wt_** | RCVQYDGRR | 41554,47 | 38606,04 | 25183,52 | 44826,89 | 42480,95 | 39789,64 | 40855,42 | 27944,84 | 44405,45 | 43637,67 | 33747,2 | 36685,14 |
| **FBXW7_505wt_** | VRCVQYDGR | 43768,19 | 41828,26 | 35549,61 | 42476,82 | 45305,71 | 45096,39 | 41227,98 | 3561,95 | 41659,79 | 45540,65 | 42472,21 | 44344,5 |
| **FBXW7_505wt_** | AVRCVQYDG | 38752,51 | 34871,52 | 31593,49 | 41005,98 | 40885,04 | 24935,97 | 26721,87 | 34991,32 | 43581,54 | 41566,15 | 19259,61 | 19347,55 |
| **FBXW7_505wt_** | AAVRCVQYD | 38457,21 | 35261,16 | 37973,02 | 41774,91 | 41578,3 | 39767,27 | 37758,34 | 37387,99 | 42958,39 | 41279,75 | 6379,81 | 30757 |
| **FBXW7_505wt_** | VAAVRCVQY | 4897,66 | 32276,91 | 7100,21 | 31542,24 | 12431,07 | 25243,54 | 13070,81 | 28435,29 | 32453,41 | 34040,21 | 2728,77 | 498,42 |
| **FBXW7_505wt_** | HVAAVRCVQ | 34356,56 | 33842,99 | 15739,92 | 43429,49 | 17927,2 | 11968,73 | 20064,73 | 30455,99 | 28008,42 | 38450,96 | 19734,88 | 10331,89 |
| **FBXW7_505wt_** | GHVAAVRCV | 37579,42 | 28249,45 | 37941,41 | 31896,77 | 36363,86 | 32336,69 | 30278,56 | 19551,04 | 1958,97 | 25956,16 | 34364,74 | 25638,25 |
| **FBXW7_505wt_** | MGHVAAVRC | 39462,07 | 27887,16 | 35372,75 | 35049,3 | 45306,21 | 34023,99 | 20164,4 | 32825,63 | 28765,78 | 37488,87 | 12005,31 | 27000,58 |
| **FBXW7_505wt_** | LMGHVAAVR | 29106,73 | 12731,21 | 2820,84 | 30622,18 | 31913,68 | 31260,25 | 25020,02 | 19803,76 | 35651,69 | 36040,31 | 26830,5 | 13378,29 |
|  |  |  |  |  |  |  |  |  |  |  |  |  |  |
| **FBXW7_R505G_** | GCVQYDGRR | 42068,81 | 40775,03 | 33163,34 | 47236,94 | 42668,43 | 44030,86 | 44145,82 | 37968,09 | 46193,23 | 45015 | 40474,39 | 41055,7 |
| **FBXW7_R505G_** | VGCVQYDGR | 39738,02 | 40842,59 | 30074,49 | 41728,81 | 45273,38 | 44943,43 | 41793,88 | 37905,29 | 46021,1 | 46775,64 | 37820,09 | 42767,33 |
| **FBXW7_R505G_** | AVGCVQYDG | 37364,13 | 32190,42 | 37436,97 | 39468,49 | 43845,91 | 40768,88 | 37870,03 | 39569,39 | 44058,02 | 43128,39 | 16662,78 | 32269,23 |
| **FBXW7_R505G_** | AAVGCVQYD | 39278,48 | 32599,09 | 38662,89 | 43145,66 | 38957,26 | 39729,41 | 41176,27 | 39021,38 | 41780,32 | 40482,72 | 8541,78 | 30115,52 |
| **FBXW7_R505G_** | VAAVGCVQY | 4115,83 | 31075,79 | 9986,12 | 35432,11 | 14566,91 | 28924,01 | 32197,04 | 32525,83 | 32355,23 | 31094,61 | 1346,75 | 358,26 |
| **FBXW7_R505G_** | HVAAVGCVQ | 32312,2 | 32640,39 | 18961,46 | 44847,25 | 15246,12 | 18429,1 | 29147,06 | 32868,27 | 28668,21 | 37979,19 | 20525,84 | 7337 |
| **FBXW7_R505G_** | GHVAAVGCV | 35582,71 | 27310,85 | 38042,95 | 33327,72 | 33749 | 32865,07 | 33113,13 | 21779,21 | 1657,41 | 24294,94 | 35920,42 | 24795 |
| **FBXW7_R505G_** | MGHVAAVGC | 40548,91 | 25426,37 | 34924,39 | 36589,62 | 44720,81 | 32775,94 | 21330,97 | 33082,7 | 26021,97 | 35857,88 | 12538,87 | 23971,42 |
| **FBXW7_R505G_** | LMGHVAAVG | 31665,7 | 5883,44 | 26286,27 | 29478,81 | 39137,64 | 29057,01 | 15789,38 | 30775,31 | 31214,96 | 32430,59 | 12542,8 | 1563,65 |
|  |  |  |  |  |  |  |  |  |  |  |  |  |  |
| **FBXW7_R505C_** | CCVQYDGRR | 37820,48 | 39923,76 | 33338,55 | 45772,82 | 39725,13 | 42246,27 | 40344,98 | 36860,21 | 44999,41 | 45384,2 | 37662,86 | 41203,45 |
| **FBXW7_R505C_** | VCCVQYDGR | 40769,75 | 41910,72 | 34641,36 | 44658,9 | 46188,73 | 45933,57 | 44211,77 | 42396,9 | 46795,38 | 47403,32 | 40410,08 | 45595,37 |
| **FBXW7_R505C_** | AVCCVQYDG | 35368,16 | 27621,7 | 32923,79 | 39125,38 | 42141,72 | 40517,33 | 37272,89 | 37080,2 | 41387,98 | 41493,36 | 13588,94 | 28193,58 |
| **FBXW7_R505C_** | AAVCCVQYD | 36439,47 | 31707,18 | 36544,5 | 41549,51 | 39944,93 | 39436,05 | 38294,86 | 37247,88 | 41216,39 | 39505,67 | 8766,96 | 30182,07 |
| **FBXW7_R505C_** | VAAVCCVQY | 4422,74 | 28231,42 | 8892,11 | 34778,83 | 19035,05 | 32111,11 | 31918,87 | 30413,83 | 35636,27 | 33753,4 | 1044,22 | 704,78 |
| **FBXW7_R505C_** | HVAAVCCVQ | 31006,6 | 26051,55 | 16848,05 | 42253,57 | 17753,09 | 23600,83 | 27564,66 | 31322,2 | 30128,22 | 38193,89 | 17289,59 | 6764,69 |
| **FBXW7_R505C_** | GHVAAVCCV | 33557,84 | 20654,4 | 36367,8 | 28032,06 | 35077 | 32547,29 | 29618,83 | 18998,01 | 754,13 | 22231,82 | 31656,1 | 21988,76 |
| **FBXW7_R505C_** | MGHVAAVCC | 39629,38 | 30146,82 | 36702,21 | 37744,85 | 46341,41 | 32989,4 | 15849,81 | 36110,18 | 26642,76 | 37463,71 | 17486,76 | 26685,17 |
| **FBXW7_R505C_** | LMGHVAAVC | 36016,92 | 3446,25 | 30503,8 | 34426,16 | 44237,61 | 31061,32 | 14994,35 | 35814,45 | 27351,65 | 33420,89 | 22601,19 | 6957,77 |
|  |  |  |  |  |  |  |  |  |  |  |  |  |  |
|  |  |  |  |  |  |  |  |  |  |  |  |  |  |
| **PROTEIN** | **PEPTIDE** | **A*01:01** | **A*02:01** | **A*03:01** | **A*24:02** | **A*26:02** | **B*07:02** | **B*08:02** | **B*27:05** | **B*39:01** | **B*40:01** | **B*58:01** | **B*15:01** |
| **FGFR2_252wt_** | SPHRPILQA | 35958,92 | 34054,94 | 29391,54 | 42141,72 | 33774,59 | 149,94 | 5130,52 | 30552,03 | 13830,43 | 35561,93 | 34345,79 | 32608,26 |
| **FGFR2_252wt_** | RSPHRPILQ | 30746,35 | 38420,6 | 19046,18 | 37635,57 | 41294,05 | 36349,3 | 31868,47 | 30577,5 | 43710,93 | 43735,07 | 23813,22 | 30915,14 |
| **FGFR2_252wt_** | ERSPHRPIL | 38295,26 | 39312,92 | 38139,79 | 35325,33 | 30335,28 | 13440,67 | 3949,91 | 3004,38 | 383,23 | 23431,88 | 37692,62 | 30435,55 |
| **FGFR2_252wt_** | VERSPHRPI | 37959,06 | 37721,59 | 33280,88 | 30511,39 | 36935,66 | 3904,74 | 7439,8 | 21816,94 | 19643,7 | 1509,72 | 34495,12 | 9223,12 |
| **FGFR2_252wt_** | VVERSPHRP | 35657,88 | 40386,89 | 40605,09 | 44139,12 | 46178,22 | 41411,29 | 40060,92 | 43929,97 | 44703,39 | 44014,2 | 33148,99 | 41919,35 |
| **FGFR2_252wt_** | DVVERSPHR | 38903,76 | 38428,5 | 21611,61 | 43641,47 | 6339,69 | 40213,36 | 32417,25 | 38875,99 | 42329,07 | 44629,91 | 43676,41 | 39454,39 |
| **FGFR2_252wt_** | LDVVERSPH | 38442,24 | 44981,39 | 37800,86 | 47279,88 | 36966,45 | 36430,02 | 35935,57 | 38552,62 | 41359,35 | 38926,92 | 41732,88 | 28127,76 |
| **FGFR2_252wt_** | HLDVVERSP | 34092,91 | 22700,2 | 37043,73 | 44962,89 | 45549,52 | 37242,64 | 33095,95 | 40225,11 | 32926,29 | 36934,07 | 34236,7 | 34960,28 |
| **FGFR2_252wt_** | YHLDVVERS | 38465,95 | 32279,35 | 41702,63 | 33737,34 | 41605,28 | 41123,28 | 38988,45 | 28182,91 | 5089,66 | 36355,98 | 37627,44 | 39723,82 |
|  |  |  |  |  |  |  |  |  |  |  |  |  |  |
| **FGFR2_S252W_** | WPHRPILQA | 34287,12 | 31713,01 | 34478,71 | 39976,5 | 30328,06 | 210,99 | 1830,3 | 22041,16 | 6468,43 | 32103,82 | 31271,74 | 28600,68 |
| **FGFR2_S252W_** | RWPHRPILQ | 42231,64 | 42564,21 | 30914,82 | 16057,11 | 44460,76 | 41089,48 | 32397,98 | 29943,64 | 45603,25 | 45285,12 | 42990,49 | 39483 |
| **FGFR2_S252W_** | ERWPHRPIL | 37410,25 | 28062,71 | 34710,78 | 26954,45 | 31813,01 | 12383,41 | 1973,65 | 179,41 | 283,12 | 18350,71 | 34244,48 | 29292,18 |
| **FGFR2_S252W_** | VERWPHRPI | 37187,08 | 34817,97 | 31681,79 | 25600,01 | 36181,75 | 3591,79 | 6381,74 | 19170,85 | 15024,88 | 1413,32 | 32592,05 | 9731,51 |
| **FGFR2_S252W_** | VVERWPHRP | 31988,68 | 36764,23 | 37601,8 | 40884,59 | 44945,87 | 41522,99 | 38320,13 | 41686,39 | 44078,05 | 43331,38 | 32379,05 | 41435,04 |
| **FGFR2_S252W_** | DVVERWPHR | 36421,73 | 36330,83 | 20944,25 | 39657,71 | 5156,17 | 39853,41 | 30716,42 | 34923,62 | 41907,99 | 44649,25 | 42055,17 | 40233,82 |
| **FGFR2_S252W_** | LDVVERWPH | 33572,39 | 42290,62 | 37215,25 | 44114,31 | 38474,68 | 38925,21 | 33980,59 | 35147,64 | 37806,59 | 34471,61 | 35852,47 | 29575,29 |
| **FGFR2_S252W_** | HLDVVERWP | 33137,14 | 20414,89 | 36655,77 | 44545,48 | 45708,48 | 41265,04 | 38210,84 | 40137,73 | 38675,02 | 39997,7 | 34429,49 | 40096,05 |
| **FGFR2_S252W_** | YHLDVVERW | 33740,98 | 27163,49 | 39550,99 | 1219,22 | 31713,35 | 35999,38 | 33947,14 | 14428,4 | 4206,23 | 28247,01 | 857,32 | 23952,75 |
|  |  |  |  |  |  |  |  |  |  |  |  |  |  |
|  |  |  |  |  |  |  |  |  |  |  |  |  |  |
| **PROTEIN** | **PEPTIDE** | **A*01:01** | **A*02:01** | **A*03:01** | **A*24:02** | **A*26:02** | **B*07:02** | **B*08:02** | **B*27:05** | **B*39:01** | **B*40:01** | **B*58:01** | **B*15:01** |
| **NRAS_61wt_** | QEEYSAMRD | 42268,21 | 45863,54 | 45973,84 | 44706,27 | 46828,28 | 45525,85 | 43199,85 | 41958,35 | 41755,92 | 26205,9 | 40112,55 | 41942,46 |
| **NRAS_61wt_** | GQEEYSAMR | 31945,45 | 23530,71 | 17336,99 | 40609,05 | 37436,18 | 40808,15 | 38219,09 | 12844,54 | 33103,82 | 23805,48 | 40446,81 | 22307,48 |
| **NRAS_61wt_** | AGQEEYSAM | 33307,18 | 30092,73 | 36777,34 | 30021,82 | 27091,59 | 11957,08 | 13548,43 | 34369,21 | 17872,19 | 28859,61 | 28108,9 | 5107,53 |
| **NRAS_61wt_** | TAGQEEYSA | 34355,08 | 33281,6 | 43295,77 | 43100,39 | 43745,93 | 31811,64 | 25047,37 | 41887,13 | 26491,56 | 40488,86 | 20610,86 | 34880,94 |
| **NRAS_61wt_** | DTAGQEEYS | 28811,26 | 41179,38 | 42076,55 | 46052 | 24936,52 | 43302,8 | 42214,73 | 45154,5 | 42635,66 | 45928,09 | 36305,67 | 42861,37 |
| **NRAS_61wt_** | LDTAGQEEY | 9306,63 | 44050,86 | 40166,83 | 43545,23 | 30732,71 | 40227,29 | 42953,3 | 39806,43 | 41172,71 | 35378,11 | 26403,71 | 20855,83 |
| **NRAS_61wt_** | ILDTAGQEE | 20706,3 | 18298,36 | 33622,92 | 43376,9 | 45039,82 | 35958,13 | 37271,26 | 40307,01 | 36807,19 | 39477,88 | 38935,76 | 34364,01 |
| **NRAS_61wt_** | DILDTAGQE | 43543,36 | 40670,61 | 40810,78 | 47314,17 | 28796,61 | 42741,44 | 41050,83 | 44945,38 | 45121,78 | 46417,17 | 44771,62 | 41269,93 |
| **NRAS_61wt_** | LDILDTAGQ | 40934,17 | 42059,27 | 42682,27 | 47125,64 | 39741,45 | 43615,5 | 43308,89 | 40482,28 | 44013,71 | 41776,71 | 39993,37 | 37257,15 |
|  |  |  |  |  |  |  |  |  |  |  |  |  |  |
| **NRAS_Q61R_** | REEYSAMRD | 40787,84 | 44428,05 | 44043,25 | 42242,15 | 46460,9 | 43892,45 | 41308,36 | 34918,33 | 41401,86 | 15981,89 | 35877,68 | 40477,03 |
| **NRAS_Q61R_** | GREEYSAMR | 41668,36 | 38777,69 | 33260 | 42966,32 | 42930,06 | 42470,38 | 38897,86 | 1290,14 | 32951,23 | 37943,05 | 43798,04 | 41138,42 |
| **NRAS_Q61R_** | AGREEYSAM | 35396,87 | 31763,83 | 29377,88 | 33329,52 | 21194,54 | 1380,61 | 3402,28 | 30209,18 | 24625,76 | 30082,64 | 26587,19 | 1427,01 |
| **NRAS_Q61R_** | TAGREEYSA | 33193,14 | 33341,78 | 41971,99 | 41653,04 | 44235,7 | 30448,72 | 19697,34 | 40152,07 | 26946,58 | 41002,44 | 17746,94 | 35395,72 |
| **NRAS_Q61R_** | DTAGREEYS | 29316,9 | 40279,1 | 41080,15 | 45405,84 | 22697,99 | 41434,58 | 36957,63 | 44520,44 | 42318,99 | 46184,24 | 36886,13 | 42322,65 |
| **NRAS_Q61R_** | LDTAGREEY | 12404,47 | 44099,52 | 38222,82 | 42728,95 | 28881,79 | 35668,3 | 40005,49 | 37162,55 | 39550,99 | 34141,27 | 29464,78 | 17909,95 |
| **NRAS_Q61R_** | ILDTAGREE | 28030,84 | 20765,76 | 31130,63 | 44187,4 | 45246,44 | 33824,33 | 36545,68 | 39838,76 | 38327,18 | 40777,69 | 39622,53 | 32728,81 |
| **NRAS_Q61R_** | DILDTAGRE | 43218,07 | 41711,67 | 41048,16 | 47165,92 | 28824,66 | 43356,26 | 41426,51 | 44966,8 | 45851,14 | 47027,84 | 45010,61 | 41929,75 |
| **NRAS_Q61R_** | LDILDTAGR | 36687,53 | 38912,59 | 32183,11 | 44857,46 | 30899,43 | 42178,21 | 41570,2 | 36148,48 | 42146,26 | 41352,18 | 39392,96 | 39306,55 |
|  |  |  |  |  |  |  |  |  |  |  |  |  |  |
| **NRAS_Q61K_** | KEEYSAMRD | 42544,42 | 45238,12 | 44773,1 | 43917,62 | 46979,01 | 45572,68 | 43366,57 | 39440,32 | 43717,07 | 22831,99 | 37377,48 | 42561 |
| **NRAS_Q61K_** | GKEEYSAMR | 42573,9 | 41042,39 | 36024,33 | 45891,34 | 43830,28 | 43898,62 | 41473,61 | 20535,63 | 38288,64 | 37624,99 | 45120,79 | 40213,36 |
| **NRAS_Q61K_** | AGKEEYSAM | 37377,07 | 33736,6 | 34555,64 | 32770,97 | 20821,79 | 7720,42 | 3563,84 | 33944,59 | 24523,38 | 31219,01 | 29613,39 | 1837,07 |
| **NRAS_Q61K_** | TAGKEEYSA | 34610,64 | 32179,62 | 42572,5 | 42164,53 | 44025,61 | 30832,62 | 21614,18 | 41545,01 | 27153,51 | 41219,5 | 19503,08 | 34713,4 |
| **NRAS_Q61K_** | DTAGKEEYS | 29895,73 | 40278,24 | 41609,79 | 45769,84 | 25589,76 | 42178,67 | 38652 | 44982,35 | 42392,34 | 46176,73 | 37355,65 | 43037,95 |
| **NRAS_Q61K_** | LDTAGKEEY | 15846,72 | 45185,29 | 40909,84 | 44242,41 | 34065,99 | 39898,28 | 42737,71 | 40480,53 | 41972,88 | 36918,88 | 31355,43 | 24468,26 |
| **NRAS_Q61K_** | ILDTAGKEE | 29431,64 | 23374,65 | 34401,95 | 45030,09 | 45832,28 | 36810,77 | 38424,76 | 41492,02 | 40245,58 | 41705,35 | 41090,83 | 35802,45 |
| **NRAS_Q61K_** | DILDTAGKE | 43627,29 | 42049,26 | 41082,82 | 47347,96 | 29040,97 | 43341,72 | 41659,36 | 45202,4 | 45867 | 47018,17 | 45018,89 | 42285,57 |
| **NRAS_Q61K_** | LDILDTAGK | 34297,88 | 38140,2 | 24209,91 | 44167,32 | 33814,45 | 41970,18 | 42075,19 | 33977,66 | 43005,85 | 39986,02 | 35759,48 | 34910,79 |
|  |  |  |  |  |  |  |  |  |  |  |  |  |  |
| **NRAS_Q61L_** | LEEYSAMRD | 39710,5 | 44227,57 | 45267,48 | 44380,98 | 46461,38 | 44942,48 | 41162,92 | 40435 | 40027,55 | 25128,54 | 37560,3 | 39927,65 |
| **NRAS_Q61L_** | GLEEYSAMR | 23750,17 | 12969,8 | 8056,66 | 41035,72 | 35296,27 | 39468,49 | 37171,8 | 26408 | 41400,53 | 37686,5 | 41358,44 | 30716,09 |
| **NRAS_Q61L_** | AGLEEYSAM | 36120,73 | 21329,13 | 34549,29 | 29428,45 | 24934,89 | 14131,44 | 12406,08 | 31385,63 | 15174,86 | 27316,75 | 19708,63 | 4012,28 |
| **NRAS_Q61L_** | TAGLEEYSA | 32136,49 | 27952,7 | 41712,13 | 42058,34 | 44228,52 | 31353,41 | 21652,56 | 41430,55 | 25777,62 | 39533,88 | 19253,16 | 34925,13 |
| **NRAS_Q61L_** | DTAGLEEYS | 25825,08 | 37162,94 | 39859,88 | 44852,11 | 21248,51 | 42296,57 | 41006,43 | 43333,28 | 40056,58 | 44262,04 | 31530,98 | 40297,86 |
| **NRAS_Q61L_** | LDTAGLEEY | 9162,25 | 42039,71 | 39319,31 | 41178,05 | 23037,95 | 39524,91 | 42350,14 | 37655,14 | 40576,56 | 33893,57 | 25561,54 | 15582,33 |
| **NRAS_Q61L_** | ILDTAGLEE | 12952,27 | 15297,68 | 27633,65 | 42142,16 | 44589,37 | 35413,71 | 37010,06 | 38993,1 | 35529,63 | 38609,38 | 36788,09 | 32932,71 |
| **NRAS_Q61L_** | DILDTAGLE | 40803,73 | 37988,64 | 38505,09 | 46130,3 | 24345,2 | 42727,11 | 40011,55 | 44008,95 | 44230,91 | 45763,91 | 43301,85 | 40051,83 |
| **NRAS_Q61L_** | LDILDTAGL | 32552,22 | 22602,91 | 39957,04 | 34585,94 | 26439,44 | 25682,41 | 26032,96 | 34308,65 | 20172,48 | 5938,18 | 29394,4 | 19872,87 |
|  |  |  |  |  |  |  |  |  |  |  |  |  |  |
|  |  |  |  |  |  |  |  |  |  |  |  |  |  |
| **PROTEIN** | **PEPTIDE** | **A*01:01** | **A*02:01** | **A*03:01** | **A*24:02** | **A*26:02** | **B*07:02** | **B*08:02** | **B*27:05** | **B*39:01** | **B*40:01** | **B*58:01** | **B*15:01** |
| **NRAS_12wt_** | GGVGKSALT | 42555,02 | 32717,12 | 40529,61 | 44292,22 | 44048,95 | 38240,62 | 35041,34 | 38614,4 | 41036,17 | 42233 | 33025,84 | 35819,88 |
| **NRAS_12wt_** | AGGVGKSAL | 41395,59 | 37727,72 | 40048,37 | 40498,05 | 45135,46 | 8070,71 | 12928,19 | 38101,85 | 26438,29 | 37266,84 | 34331,66 | 26978,68 |
| **NRAS_12wt_** | GAGGVGKSA | 41691,82 | 36104,71 | 41513,57 | 46064,96 | 42863,22 | 19759,45 | 31377,84 | 36937,66 | 36857,02 | 38797,4 | 30676,91 | 27374,15 |
| **NRAS_12wt_** | VGAGGVGKS | 40112,99 | 38122,05 | 38782,29 | 43760,15 | 42342,36 | 37437,38 | 41561,65 | 40982,03 | 44009,91 | 44638,6 | 32018,47 | 34476,11 |
| **NRAS_12wt_** | VVGAGGVGK | 33041,2 | 33838,23 | 247,59 | 42170,89 | 37006,05 | 33608,35 | 41400,53 | 31974,51 | 44932,25 | 43724,63 | 30976,42 | 27511,63 |
| **NRAS_12wt_** | VVVGAGGVG | 39796,54 | 35040,19 | 36348,9 | 43699,57 | 29721,89 | 21409,82 | 36999,65 | 38520,51 | 39166,45 | 39605,39 | 23915,21 | 8184,41 |
| **NRAS_12wt_** | LVVVGAGGV | 30656,66 | 3818,17 | 30038,73 | 40547,6 | 8683,6 | 21639,46 | 26665,54 | 30907,12 | 30697,83 | 34869,63 | 24690,85 | 10748,43 |
| **NRAS_12wt_** | KLVVVGAGG | 40247,73 | 7808,12 | 24998,36 | 40992,68 | 40701 | 33425,96 | 32631,57 | 27047,95 | 39820,21 | 36296,64 | 19771 | 5358,07 |
| **NRAS_12wt_** | YKLVVVGAG | 43169,94 | 38426 | 41986,07 | 42259,05 | 42367,57 | 35876,52 | 20889,02 | 17249,05 | 17408,22 | 31170,75 | 34030,62 | 23499,68 |
|  |  |  |  |  |  |  |  |  |  |  |  |  |  |
| **NRAS_G12D_** | DGVGKSALT | 41713,02 | 40144,25 | 43706,67 | 43791,88 | 36965,24 | 38631,52 | 25051,44 | 43426,66 | 36247,98 | 44808,46 | 39858,6 | 42436,38 |
| **NRAS_G12D_** | ADGVGKSAL | 40581,4 | 41470,03 | 42758,09 | 43030,51 | 42415,25 | 23436,96 | 24586,36 | 41424,72 | 33353,69 | 18514,85 | 43235,86 | 36373,31 |
| **NRAS_G12D_** | GADGVGKSA | 29969,57 | 32620,97 | 40211,18 | 45982,29 | 42342,36 | 21777,57 | 34488,4 | 38356,23 | 27472,36 | 31865,72 | 30514,7 | 32902,77 |
| **NRAS_G12D_** | VGADGVGKS | 39982,97 | 38789,42 | 40812,57 | 44543,56 | 42193,25 | 39426,23 | 42247,63 | 42378,56 | 44320,97 | 44522,36 | 33534,26 | 35535,4 |
| **NRAS_G12D_** | VVGADGVGK | 33577,11 | 35485,07 | 1104,62 | 44031,32 | 38243,5 | 37932,78 | 43687,76 | 35680,26 | 45951,46 | 44619,78 | 31725,71 | 31581,51 |
| **NRAS_G12D_** | VVVGADGVG | 39979,96 | 35733,96 | 39973,9 | 44914,75 | 35498,11 | 31815,41 | 39659,83 | 41137,52 | 40877,53 | 40745,48 | 20626,26 | 13231,74 |
| **NRAS_G12D_** | LVVVGADGV | 27214,97 | 1973,93 | 34742,71 | 39045,87 | 17919,44 | 29821,1 | 31481,89 | 33016,54 | 26708,28 | 32563,86 | 18829,19 | 15519,4 |
| **NRAS_G12D_** | KLVVVGADG | 41160,23 | 16029,34 | 31676,99 | 42381,79 | 43338,42 | 37152,08 | 36157,48 | 33830,92 | 42568,36 | 39119,88 | 24615,37 | 9848,77 |
| **NRAS_G12D_** | YKLVVVGAD | 44791,99 | 40800,2 | 44021,32 | 43692,5 | 44509,36 | 40374,68 | 30692,17 | 26314,73 | 25449,77 | 33478,79 | 35716,57 | 34014,05 |
|  |  |  |  |  |  |  |  |  |  |  |  |  |  |
|  |  |  |  |  |  |  |  |  |  |  |  |  |  |
| **PROTEIN** | **PEPTIDE** | **A*01:01** | **A*02:01** | **A*03:01** | **A*24:02** | **A*26:02** | **B*07:02** | **B*08:02** | **B*27:05** | **B*39:01** | **B*40:01** | **B*58:01** | **B*15:01** |
| **NRAS_13wt_** | GVGKSALTI | 33068,75 | 11060,83 | 24204,95 | 22383,87 | 36559,92 | 21243,45 | 26530,58 | 28730,33 | 32708,62 | 32115,97 | 9639,92 | 12178,26 |
| **NRAS_13wt_** | GGVGKSALT | 42555,02 | 32717,12 | 40529,61 | 44292,22 | 44048,95 | 38240,62 | 35041,34 | 38614,4 | 41036,17 | 42233 | 33025,84 | 35819,88 |
| **NRAS_13wt_** | AGGVGKSAL | 41395,59 | 37727,72 | 40048,37 | 40498,05 | 45135,46 | 8070,71 | 12928,19 | 38101,85 | 26438,29 | 37266,84 | 34331,66 | 26978,68 |
| **NRAS_13wt_** | GAGGVGKSA | 41691,82 | 36104,71 | 41513,57 | 46064,96 | 42863,22 | 19759,45 | 31377,84 | 36937,66 | 36857,02 | 38797,4 | 30676,91 | 27374,15 |
| **NRAS_13wt_** | VGAGGVGKS | 40112,99 | 38122,05 | 38782,29 | 43760,15 | 42342,36 | 37437,38 | 41561,65 | 40982,03 | 44009,91 | 44638,6 | 32018,47 | 34476,11 |
| **NRAS_13wt_** | VVGAGGVGK | 33041,2 | 33838,23 | 247,59 | 42170,89 | 37006,05 | 33608,35 | 41400,53 | 31974,51 | 44932,25 | 43724,63 | 30976,42 | 27511,63 |
| **NRAS_13wt_** | VVVGAGGVG | 39796,54 | 35040,19 | 36348,9 | 43699,57 | 29721,89 | 21409,82 | 36999,65 | 38520,51 | 39166,45 | 39605,39 | 23915,21 | 8184,41 |
| **NRAS_13wt_** | LVVVGAGGV | 30656,66 | 3818,17 | 30038,73 | 40547,6 | 8683,6 | 21639,46 | 26665,54 | 30907,12 | 30697,83 | 34869,63 | 24690,85 | 10748,43 |
| **NRAS_13wt_** | KLVVVGAGG | 40247,73 | 7808,12 | 24998,36 | 40992,68 | 40701 | 33425,96 | 32631,57 | 27047,95 | 39820,21 | 36296,64 | 19771 | 5358,07 |
|  |  |  |  |  |  |  |  |  |  |  |  |  |  |
| **NRAS_G13R_** | RVGKSALTI | 28764,53 | 7459,39 | 12818,71 | 4566,87 | 35746,71 | 5211,59 | 15504,47 | 14148,89 | 25611,65 | 26389,71 | 1176,86 | 4553,01 |
| **NRAS_G13R_** | GRVGKSALT | 44839,01 | 41164,7 | 41682,34 | 44383,38 | 41401,43 | 38587,25 | 30890,4 | 2497,61 | 27389,27 | 37796,35 | 41538,29 | 39814,61 |
| **NRAS_G13R_** | AGRVGKSAL | 40670,16 | 37404,18 | 32369,95 | 39370,39 | 41625,55 | 283,04 | 2228,32 | 30669,61 | 26555,84 | 32808,57 | 32730,23 | 11381,83 |
| **NRAS_G13R_** | GAGRVGKSA | 40948,35 | 37917,2 | 40782,97 | 45570,21 | 44233,79 | 18104,38 | 22985,42 | 33957,07 | 36853,43 | 39705,36 | 27450,08 | 26820,64 |
| **NRAS_G13R_** | VGAGRVGKS | 41301,19 | 39960,05 | 38137,72 | 42732,64 | 41621,95 | 35541,93 | 31274,45 | 39022,21 | 44227,57 | 45852,62 | 34661,99 | 35175,04 |
| **NRAS_G13R_** | VVGAGRVGK | 35963,18 | 35046,27 | 171,29 | 41049,93 | 37591,2 | 30357,94 | 37444,68 | 29697,14 | 44372,79 | 44471,34 | 31987,3 | 29575,29 |
| **NRAS_G13R_** | VVVGAGRVG | 42594,63 | 36907,3 | 37823,36 | 43565,49 | 35086,86 | 22933 | 35750,97 | 38263,39 | 39778,89 | 40294,79 | 22939,95 | 10533,83 |
| **NRAS_G13R_** | LVVVGAGRV | 30106,06 | 8999,26 | 31031,77 | 39108,02 | 11932,66 | 24107,46 | 26086,24 | 31433,56 | 34266,72 | 36984,05 | 24008,8 | 13406,25 |
| **NRAS_G13R_** | KLVVVGAGR | 38899,12 | 17497,55 | 1102,49 | 39771,58 | 32945,88 | 35247,05 | 36504,98 | 14936,06 | 41981,98 | 39196,54 | 32306,96 | 21494,78 |
|  |  |  |  |  |  |  |  |  |  |  |  |  |  |
| **NRAS_G13D_** | DVGKSALTI | 29414,13 | 26006,77 | 35749,43 | 24279,96 | 16254,29 | 26546,08 | 16119,6 | 37832,78 | 25037,89 | 38611,46 | 25352,2 | 27875,68 |
| **NRAS_G13D_** | GDVGKSALT | 41313,26 | 40754,76 | 42432,24 | 45893,33 | 40580,06 | 42342,82 | 39479,18 | 40261,26 | 42102,52 | 33712,16 | 42754,37 | 41763,59 |
| **NRAS_G13D_** | AGDVGKSAL | 31572,97 | 30485 | 38617,74 | 40687,35 | 44395,86 | 6716,99 | 18214,8 | 36970,43 | 11205,98 | 26527,41 | 31455 | 33350,81 |
| **NRAS_G13D_** | GAGDVGKSA | 41167,35 | 34336,49 | 42439,14 | 46512,2 | 42550,87 | 22918,36 | 32149,34 | 38675,02 | 36991,66 | 37878,65 | 31197,39 | 27852,47 |
| **NRAS_G13D_** | VGAGDVGKS | 41579,64 | 40294,79 | 41842,29 | 45306,71 | 43789,99 | 40818,73 | 43224,62 | 42667,96 | 45203,86 | 45689,18 | 31946,83 | 37500,21 |
| **NRAS_G13D_** | VVGAGDVGK | 33557,13 | 34585,56 | 1911,19 | 43407,88 | 39592,95 | 39072,07 | 43218,55 | 36841,46 | 45655,1 | 44723,2 | 29510,08 | 32660,18 |
| **NRAS_G13D_** | VVVGAGDVG | 39172,38 | 34941,01 | 40708,48 | 42523,71 | 36479,32 | 31767,27 | 40196,4 | 40316,6 | 37943,86 | 39379,76 | 19880,39 | 13837,77 |
| **NRAS_G13D_** | LVVVGAGDV | 31620,15 | 11985,45 | 35274,52 | 41712,13 | 19543,85 | 25775,38 | 29994,88 | 35453,2 | 34878,69 | 37795,54 | 29138,23 | 16097,3 |
| **NRAS_G13D_** | KLVVVGAGD | 42124,85 | 11792,76 | 33210,74 | 42937,04 | 43342,17 | 40481,84 | 39453,55 | 33880,72 | 43056,13 | 39362,72 | 23898,39 | 18194,72 |
|  |  |  |  |  |  |  |  |  |  |  |  |  |  |
|  |  |  |  |  |  |  |  |  |  |  |  |  |  |
| **PROTEIN** | **PEPTIDE** | **A*01:01** | **A*02:01** | **A*03:01** | **A*24:02** | **A*26:02** | **B*07:02** | **B*08:02** | **B*27:05** | **B*39:01** | **B*40:01** | **B*58:01** | **B*15:01** |
| **POLE_286wt_** | PDAETDQIM | 39550,99 | 44778,9 | 46285,29 | 44519,46 | 43342,64 | 41948,82 | 42840,98 | 46059,96 | 40659,62 | 37240,62 | 41492,9 | 39321,01 |
| **POLE_286wt_** | FPDAETDQI | 14984,46 | 13884,41 | 40195,08 | 33898,34 | 35765,29 | 5946,09 | 15788,7 | 34244,48 | 1743,36 | 26800,91 | 19967,92 | 34734,07 |
| **POLE_286wt_** | KFPDAETDQ | 44996,48 | 44786,18 | 40414,43 | 42316,26 | 46148,27 | 45585,99 | 45779,25 | 45500,26 | 47559,49 | 46808,54 | 44440,06 | 44651,64 |
| **POLE_286wt_** | LKFPDAETD | 47050,23 | 44615,92 | 46747,81 | 45940,53 | 47252,27 | 45495,33 | 43175,56 | 39894,83 | 33132,12 | 37498,59 | 33729,66 | 38383,2 |
| **POLE_286wt_** | PLKFPDAET | 42622,75 | 29784,35 | 40416,18 | 45338,07 | 45063,71 | 40180,3 | 37353,62 | 44658,41 | 46073,42 | 45110,05 | 43650,9 | 35625,47 |
| **POLE_286wt_** | LPLKFPDAE | 42589,55 | 41801,59 | 40151,63 | 45146,2 | 44696,62 | 13093,88 | 20364,37 | 37201,98 | 26316,16 | 41441,32 | 35198,27 | 38117,51 |
| **POLE_286wt_** | KLPLKFPDA | 35813,69 | 943,45 | 23482,13 | 21871,3 | 38853,29 | 29403,64 | 8878,08 | 31687,96 | 36235,03 | 38159,59 | 39145,69 | 21441,35 |
| **POLE_286wt_** | TKLPLKFPD | 45703,53 | 43425,74 | 44799,24 | 42725,26 | 47187,37 | 40915,59 | 33230,86 | 27933,36 | 27072,83 | 37537,57 | 35421 | 40972,73 |
| **POLE_286wt_** | TTKLPLKFP | 38937,02 | 37666,53 | 33499,8 | 42676,27 | 35073,58 | 38229,45 | 28971,61 | 41973,8 | 44571,52 | 43861,58 | 26723,02 | 33987,56 |
|  |  |  |  |  |  |  |  |  |  |  |  |  |  |
| **POLE_P286R_** | RDAETDQIM | 32259,46 | 36255,41 | 41416,2 | 36936,05 | 34594,55 | 24529,48 | 32948,75 | 36103,54 | 28606,25 | 7141,89 | 25168,26 | 16020,85 |
| **POLE_P286R_** | FRDAETDQI | 21800,67 | 24005,16 | 43229,78 | 30250,39 | 40119,04 | 32786,93 | 27851,26 | 12792,66 | 430,63 | 20919,79 | 31219,01 | 36405,57 |
| **POLE_P286R_** | KFRDAETDQ | 46132,8 | 45580,08 | 31828,85 | 44556,11 | 46625,57 | 40889,92 | 42423,51 | 42754,37 | 47460,28 | 45903,75 | 41696,77 | 38328,84 |
| **POLE_P286R_** | LKFRDAETD | 46743,26 | 45172,09 | 46649,27 | 45546,55 | 47366,92 | 45128,12 | 40196,4 | 35699,18 | 34716,78 | 37806,17 | 33885,14 | 38779,79 |
| **POLE_P286R_** | PLKFRDAET | 43005,38 | 36685,93 | 42008,79 | 45505,66 | 45488,93 | 40223,81 | 16864,28 | 44307,08 | 45886,88 | 46782,7 | 45572,68 | 36876,95 |
| **POLE_P286R_** | LPLKFRDAE | 43096,2 | 42442,8 | 38654,52 | 45046,16 | 43963,25 | 4153,68 | 9985,36 | 34523,88 | 26640,17 | 41706,7 | 37686,11 | 38393,18 |
| **POLE_P286R_** | KLPLKFRDA | 39387,43 | 7788,21 | 27587,64 | 30343,16 | 39980,39 | 29125,3 | 6036,19 | 33579,64 | 37876,19 | 41244,04 | 40376,42 | 21935,05 |
| **POLE_P286R_** | TKLPLKFRD | 46462,4 | 45908,22 | 45911,68 | 44475,67 | 47827,85 | 45095,92 | 38830,99 | 30354,01 | 39116,06 | 42970,95 | 38148,86 | 45218,06 |
| **POLE_P286R_** | TTKLPLKFR | 32281,1 | 38145,57 | 3659,26 | 39699,77 | 19039,58 | 37523,75 | 33742,8 | 32149,69 | 43919,04 | 44726,61 | 35374,27 | 32975,49 |
|  |  |  |  |  |  |  |  |  |  |  |  |  |  |
|  |  |  |  |  |  |  |  |  |  |  |  |  |  |
| **PROTEIN** | **PEPTIDE** | **A*01:01** | **A*02:01** | **A*03:01** | **A*24:02** | **A*26:02** | **B*07:02** | **B*08:02** | **B*27:05** | **B*39:01** | **B*40:01** | **B*58:01** | **B*15:01** |
| **PPP2R1A_179wt_** | PMVRRAAAS | 40921,79 | 30790,3 | 36234,63 | 42061,08 | 39946,66 | 33710,7 | 15098,38 | 37358,47 | 39358,48 | 43553,24 | 41399,19 | 25170,99 |
| **PPP2R1A_179wt_** | TPMVRRAAA | 33891,74 | 31684,18 | 28009,02 | 38971,58 | 30553,02 | 10,52 | 19,12 | 21391,99 | 4409,6 | 34481,69 | 38549,26 | 27721,69 |
| **PPP2R1A_179wt_** | DTPMVRRAA | 34300,11 | 35403,76 | 40175,09 | 41015,32 | 11021,29 | 20991,89 | 11258,25 | 39501,82 | 29280,77 | 42596,01 | 41498,74 | 35223,41 |
| **PPP2R1A_179wt_** | DDTPMVRRA | 41159,33 | 44568,16 | 44615,92 | 44959,5 | 40778,58 | 40654,77 | 33290,6 | 41831,44 | 39590,8 | 42918,46 | 44117,16 | 43631,54 |
| **PPP2R1A_179wt_** | SDDTPMVRR | 25332,46 | 44095,68 | 28500,28 | 46199,22 | 39580,95 | 43614,54 | 44689,84 | 40602,92 | 45109,1 | 41783,48 | 43328,11 | 44984,32 |
| **PPP2R1A_179wt_** | CSDDTPMVR | 1525,12 | 32219,68 | 12127,63 | 40675,02 | 33833,1 | 36481,3 | 37516,85 | 29325,46 | 33336,75 | 38687,59 | 21768,14 | 35803,23 |
| **PPP2R1A_179wt_** | LCSDDTPMV | 27700,71 | 4552,86 | 40368,56 | 40450,31 | 40296,12 | 36739,17 | 36233,07 | 41799,32 | 31897,79 | 35682,95 | 17721,04 | 34883,21 |
| **PPP2R1A_179wt_** | NLCSDDTPM | 21817,42 | 1839,42 | 32340,88 | 35842,37 | 20193,01 | 21319,67 | 15616,25 | 30738,7 | 7999,94 | 26074,95 | 25628,55 | 1951,59 |
| **PPP2R1A_179wt_** | RNLCSDDTP | 42474,05 | 41347,27 | 43155,93 | 40384,29 | 47043,62 | 42125,31 | 39592,11 | 36734,39 | 37114,72 | 34617,76 | 26992,39 | 38790,27 |
|  |  |  |  |  |  |  |  |  |  |  |  |  |  |
| **PPP2R1A_P179R_** | RMVRRAAAS | 36282,1 | 9159,38 | 9233,81 | 32784,1 | 33609,82 | 6618,73 | 1246,16 | 10263,92 | 21472,22 | 29154 | 23639,17 | 2124,59 |
| **PPP2R1A_P179R_** | TRMVRRAAA | 37500,21 | 35012,54 | 31721,93 | 36070,36 | 35845,09 | 6000,38 | 94,11 | 662,45 | 2045,57 | 33464,31 | 39796,11 | 26219,51 |
| **PPP2R1A_P179R_** | DTRMVRRAA | 36204,46 | 41136,64 | 34764,52 | 42477,25 | 15784,77 | 6055,95 | 1571,33 | 33908,59 | 30931,87 | 41210,59 | 40779 | 27664,77 |
| **PPP2R1A_P179R_** | DDTRMVRRA | 40885,04 | 45662,49 | 44763,4 | 44746,46 | 41578,75 | 40819,18 | 28185,94 | 39856,85 | 39723,39 | 44035,15 | 44288,39 | 44189,31 |
| **PPP2R1A_P179R_** | SDDTRMVRR | 24051,45 | 43713,3 | 28498,73 | 45208,76 | 39060,66 | 43198,9 | 39916,84 | 39210,98 | 44289,82 | 43363,28 | 43063,11 | 45372,91 |
| **PPP2R1A_P179R_** | CSDDTRMVR | 2922,62 | 32302,76 | 9824,4 | 39085,17 | 30573,52 | 29957,9 | 32379,74 | 24607,1 | 31095,29 | 37290,23 | 23531,98 | 32110,4 |
| **PPP2R1A_P179R_** | LCSDDTRMV | 34752,48 | 19537,51 | 42291,98 | 43123,72 | 42418,02 | 35909,53 | 34167,88 | 42318,09 | 36265,23 | 39952,27 | 23453,44 | 35576,17 |
| **PPP2R1A_P179R_** | NLCSDDTRM | 24550,47 | 6532,01 | 35793,54 | 38137,32 | 25998,05 | 32579,36 | 26316,16 | 34320,91 | 24590,08 | 34865,11 | 27585,56 | 9585,53 |
| **PPP2R1A_P179R_** | RNLCSDDTR | 38783,14 | 40723,89 | 29466,05 | 40675,46 | 43280,8 | 41118,85 | 39209,71 | 22162,65 | 41013,1 | 41767,66 | 31910,57 | 39993,8 |
|  |  |  |  |  |  |  |  |  |  |  |  |  |  |
|  |  |  |  |  |  |  |  |  |  |  |  |  |  |
| **PROTEIN** | **PEPTIDE** | **A*01:01** | **A*02:01** | **A*03:01** | **A*24:02** | **A*26:02** | **B*07:02** | **B*08:02** | **B*27:05** | **B*39:01** | **B*40:01** | **B*58:01** | **B*15:01** |
| **PPP2R1A_183wt_** | RAAASKLGE | 39950,98 | 35346,73 | 23119,86 | 43300,46 | 44143,43 | 20972,14 | 32007,38 | 28938,09 | 39342,29 | 39247,48 | 8467,52 | 24967,83 |
| **PPP2R1A_183wt_** | RRAAASKLG | 43793,29 | 43158,27 | 36397,71 | 37844,23 | 43779,09 | 29478,48 | 30269,71 | 698,75 | 29695,85 | 35785,41 | 27067,56 | 30406,59 |
| **PPP2R1A_183wt_** | VRRAAASKL | 41520,74 | 37984,95 | 32782,68 | 32390,6 | 37364,54 | 7529,45 | 9415 | 828,74 | 12094,35 | 29614,99 | 35511,55 | 21088,42 |
| **PPP2R1A_183wt_** | MVRRAAASK | 31275,82 | 28904,61 | 44,02 | 34680,37 | 17042,02 | 4317,58 | 10245,17 | 11747,55 | 37220,09 | 35742,07 | 20709,44 | 6832,73 |
| **PPP2R1A_183wt_** | PMVRRAAAS | 40921,79 | 30790,3 | 36234,63 | 42061,08 | 39946,66 | 33710,7 | 15098,38 | 37358,47 | 39358,48 | 43553,24 | 41399,19 | 25170,99 |
| **PPP2R1A_183wt_** | TPMVRRAAA | 33891,74 | 31684,18 | 28009,02 | 38971,58 | 30553,02 | 10,52 | 19,12 | 21391,99 | 4409,6 | 34481,69 | 38549,26 | 27721,69 |
| **PPP2R1A_183wt_** | DTPMVRRAA | 34300,11 | 35403,76 | 40175,09 | 41015,32 | 11021,29 | 20991,89 | 11258,25 | 39501,82 | 29280,77 | 42596,01 | 41498,74 | 35223,41 |
| **PPP2R1A_183wt_** | DDTPMVRRA | 41159,33 | 44568,16 | 44615,92 | 44959,5 | 40778,58 | 40654,77 | 33290,6 | 41831,44 | 39590,8 | 42918,46 | 44117,16 | 43631,54 |
| **PPP2R1A_183wt_** | SDDTPMVRR | 25332,46 | 44095,68 | 28500,28 | 46199,22 | 39580,95 | 43614,54 | 44689,84 | 40602,92 | 45109,1 | 41783,48 | 43328,11 | 44984,32 |
|  |  |  |  |  |  |  |  |  |  |  |  |  |  |
| **PPP2R1A_R183W_** | WAAASKLGE | 33193,48 | 34100,65 | 35419,86 | 43682,55 | 37780,82 | 27516,11 | 27172,6 | 33190,61 | 31454,31 | 38119,99 | 12262,48 | 25916,04 |
| **PPP2R1A_R183W_** | RWAAASKLG | 41682,34 | 38695,12 | 31162,31 | 7161,78 | 43213,42 | 31339,82 | 29941,37 | 17609,22 | 41129,51 | 40705,4 | 22460,29 | 22129,34 |
| **PPP2R1A_R183W_** | VRWAAASKL | 38017,02 | 20614,65 | 30783,97 | 19373,31 | 36928,85 | 18878,55 | 19231,92 | 39,57 | 1124,49 | 19290,9 | 24100,68 | 24925,18 |
| **PPP2R1A_R183W_** | MVRWAAASK | 28957,21 | 20984,4 | 30,24 | 30629,15 | 11215,45 | 4154,21 | 12673,08 | 11889,49 | 33405,7 | 32240,96 | 17508,16 | 4930,68 |
| **PPP2R1A_R183W_** | PMVRWAAAS | 37784,92 | 15736,51 | 34026,21 | 38286,14 | 36747,51 | 36913,68 | 32219,68 | 35894,76 | 37154,91 | 41319,97 | 37887,67 | 22402,77 |
| **PPP2R1A_R183W_** | TPMVRWAAA | 27902,55 | 19244,83 | 23737,32 | 32026,09 | 20292,22 | 16,76 | 24,34 | 16596,56 | 2449,86 | 27977,22 | 32201,57 | 22924,81 |
| **PPP2R1A_R183W_** | DTPMVRWAA | 19906,23 | 16863,19 | 36394,57 | 30729,72 | 5459,25 | 24476,99 | 8222,13 | 35274,13 | 16659,17 | 37275,3 | 34193,39 | 33292,04 |
| **PPP2R1A_R183W_** | DDTPMVRWA | 40908,94 | 42709,53 | 43375,02 | 44991,11 | 40149,45 | 39629,81 | 33137,14 | 41648,99 | 39996,38 | 42164,96 | 43102,73 | 42718,79 |
| **PPP2R1A_R183W_** | SDDTPMVRW | 22600,21 | 42670,27 | 41666,56 | 31092,27 | 38611,89 | 37542,43 | 42707,21 | 40217,71 | 41272,17 | 32855,83 | 9003,25 | 39440,32 |

Overlapping peptides have been designed to have the wt and mutated aminoacid residue in all nine positions. Values of binding affinity (nM) to each haplotypes are reported. Green, neoepitopes with affinity <100nM; Red, neoepitopes with affinity between 100-200 nM; Gold, neoepitopes with affinity between 200-400 nM.

**SUPPL. TABLE. 4** List of all peptides from the wt and mutated sequences derived from proteins with frameshift mutations.

| **PROTEIN** | **PEPTIDE** | **A*01:01** | **A*02:01** | **A*03:01** | **A*24:02** | **A*26:02** | **B*07:02** | **B*08:02** | **B*27:05** | **B*39:01** | **B*40:01** | **B*58:01** | **B*15:01** |
| --- | --- | --- | --- | --- | --- | --- | --- | --- | --- | --- | --- | --- | --- |
| **RPL22_15wt_** | LVVKGGKKK | 40773,71 | 39896,55 | 1832,44 | 45993,74 | 31533,72 | 33773,85 | 40250,36 | 30746,69 | 45398,46 | 44446,31 | 35878,47 | 26973,43 |
| **RPL22_15wt_** | VVKGGKKKK | 45342,02 | 44742,59 | 4673,75 | 46192,22 | 41487,98 | 38580,15 | 39133,83 | 39034,46 | 47684,18 | 46729,60 | 42559,60 | 36164,93 |
| **RPL22_15wt_** | VKGGKKKKQ | 48728,34 | 49072,27 | 47075,70 | 49230,73 | 48692,00 | 47797,32 | 43984,66 | 42790,47 | 48391,03 | 48346,05 | 48094,57 | 47740,46 |
| **RPL22_15wt_** | KGGKKKKQV | 46206,73 | 44430,92 | 42853,50 | 43883,90 | 47938,18 | 38330,49 | 17791,35 | 40620,49 | 45461,36 | 46617,48 | 40102,12 | 44181,64 |
| **RPL22_15wt_** | GGKKKKQVL | 44789,56 | 44661,32 | 43552,79 | 42411,59 | 46590,76 | 25243,26 | 889,79 | 38138,14 | 39153,75 | 39739,73 | 41206,58 | 31711,64 |
| **RPL22_15wt_** | GKKKKQVLK | 44774,54 | 45606,22 | 19050,09 | 45976,81 | 45662,99 | 43572,09 | 29890,55 | 8902,41 | 45699,58 | 42710,46 | 43984,20 | 35857,88 |
| **RPL22_15wt_** | KKKKQVLKF | 42496,11 | 43871,09 | 35836,93 | 26387,14 | 36200,93 | 35277,95 | 13204,28 | 6542,48 | 37165,77 | 33796,53 | 23252,07 | 8411,18 |
| **RPL22_15wt_** | KKKQVLKFT | 46759,93 | 44790,51 | 40396,53 | 45455,47 | 45312,09 | 38979,17 | 30217,03 | 17896,58 | 43326,71 | 42974,69 | 42552,24 | 37405,00 |
| **RPL22_15wt_** | KKQVLKFTL | 42095,22 | 25937,64 | 36741,95 | 21041,24 | 44061,34 | 21095,94 | 8239,06 | 2231,73 | 2853,66 | 12524,91 | 19690,09 | 25079,65 |
|  |  |  |  |  |  |  |  |  |  |  |  |  |  |
| **RPL22_K15Rfs*5_** | LVVKGGKKR | 41953,83 | 38915,95 | 14613,79 | 45449,58 | 26693,26 | 34698,00 | 39741,02 | 33436,44 | 44289,82 | 44465,55 | 40197,25 | 32351,04 |
| **RPL22_K15Rfs*5_** | VVKGGKKRS | 46640,18 | 45495,82 | 39659,83 | 47697,62 | 44601,91 | 36809,99 | 35498,11 | 44017,04 | 47824,21 | 47335,68 | 43682,55 | 37694,67 |
| **RPL22_K15Rfs*5_** | VKGGKKRSK | 47420,26 | 47161,33 | 33811,88 | 47572,37 | 47286,53 | 43562,68 | 36090,25 | 25561,54 | 46219,73 | 46891,17 | 46205,21 | 43160,61 |
| **RPL22_K15Rfs*5_** | KGGKKRSKF | 40856,29 | 45759,45 | 36465,12 | 22642,80 | 42777,98 | 25614,41 | 10032,79 | 30399,36 | 44615,43 | 44284,54 | 20388,83 | 21806,80 |
|  |  |  |  |  |  |  |  |  |  |  |  |  |  |
| **PROTEIN** | **PEPTIDE** | **A*01:01** | **A*02:01** | **A*03:01** | **A*24:02** | **A*26:02** | **B*07:02** | **B*08:02** | **B*27:05** | **B*39:01** | **B*40:01** | **B*58:01** | **B*15:01** |
| **ACVR2A_437wt_** | QEVVVHKKK | 41558,06 | 43810,36 | 29911,26 | 45596,84 | 40254,71 | 42943,55 | 41356,66 | 27274,82 | 42607,52 | 27517,88 | 41151,77 | 35673,70 |
| **ACVR2A_437wt_** | EVVVHKKKR | 43296,24 | 44415,57 | 29367,06 | 46617,48 | 19280,06 | 42544,89 | 37037,71 | 41368,74 | 45386,16 | 46625,05 | 44505,02 | 43900,04 |
| **ACVR2A_437wt_** | VVVHKKKRP | 46219,73 | 43563,16 | 42371,70 | 47136,85 | 45838,22 | 42102,52 | 33228,35 | 44752,75 | 47537,38 | 47087,93 | 42117,54 | 42041,53 |
| **ACVR2A_437wt_** | VVHKKKRPV | 39626,39 | 26477,52 | 28747,73 | 37845,86 | 34391,14 | 4621,85 | 265,38 | 29708,71 | 32348,58 | 38907,97 | 36620,09 | 18747,67 |
| **ACVR2A_437wt_** | VHKKKRPVL | 41423,82 | 40639,83 | 37227,75 | 27933,96 | 42790,47 | 12542,80 | 1363,00 | 20654,84 | 5344,41 | 30017,27 | 39190,20 | 23602,11 |
| **ACVR2A_437wt_** | HKKKRPVLR | 43926,66 | 45257,71 | 28742,13 | 44737,25 | 41461,50 | 39392,56 | 17151,35 | 9720,46 | 38802,03 | 43614,54 | 44718,39 | 38405,21 |
| **ACVR2A_437wt_** | KKKRPVLRD | 47574,95 | 47198,60 | 43496,26 | 46268,75 | 47449,50 | 44858,42 | 37611,96 | 23564,84 | 46291,80 | 44996,97 | 40024,09 | 41910,72 |
| **ACVR2A_437wt_** | KKRPVLRDY | 40278,24 | 44543,10 | 28375,97 | 43762,52 | 34324,98 | 31579,12 | 35962,02 | 14707,54 | 42199,21 | 40332,31 | 30963,02 | 6281,25 |
| **ACVR2A_437wt_** | KRPVLRDYW | 39891,39 | 40648,61 | 39613,95 | 9126,34 | 39969,57 | 35011,39 | 31643,42 | 3382,13 | 33220,79 | 36966,83 | 9390,69 | 31728,78 |
|  |  |  |  |  |  |  |  |  |  |  |  |  |  |
| **ACVR2A_K437Rfs*5_** | QEVVVHKKR | 42141,72 | 44596,62 | 35819,51 | 45948,98 | 38606,88 | 43456,28 | 40271,26 | 29711,60 | 41580,98 | 30181,41 | 42647,66 | 40686,90 |
| **ACVR2A_K437Rfs*5_** | EVVVHKKRG | 45592,90 | 47225,18 | 44881,74 | 47788,02 | 35780,38 | 42165,43 | 28519,11 | 43657,04 | 46554,48 | 47820,60 | 42853,02 | 43351,56 |
| **ACVR2A_K437Rfs*5_** | VVVHKKRGL | 41580,09 | 29682,36 | 31806,12 | 37705,68 | 28900,23 | 8615,66 | 5681,99 | 32593,82 | 37417,13 | 39116,06 | 34258,57 | 21939,57 |
| **ACVR2A_K437Rfs*5_** | VVHKKRGLF | 27819,96 | 36990,44 | 17476,93 | 10958,27 | 7825,21 | 3725,66 | 1041,08 | 22524,06 | 39670,57 | 34535,07 | 16626,22 | 1037,44 |
|  |  |  |  |  |  |  |  |  |  |  |  |  |  |
|  |  |  |  |  |  |  |  |  |  |  |  |  |  |
| **PROTEIN** | **PEPTIDE** | **A*01:01** | **A*02:01** | **A*03:01** | **A*24:02** | **A*26:02** | **B*07:02** | **B*08:02** | **B*27:05** | **B*39:01** | **B*40:01** | **B*58:01** | **B*15:01** |
| **RNF43_659wt_** | HPQRKRRGG | 44963,38 | 47423,84 | 43492,50 | 46303,32 | 45410,26 | 2570,48 | 3106,32 | 33262,16 | 38811,26 | 46667,95 | 44150,12 | 43589,07 |
| **RNF43_659wt_** | PQRKRRGGP | 47142,99 | 47910,71 | 45651,16 | 46201,73 | 47252,79 | 39652,54 | 26971,67 | 38168,28 | 46737,68 | 46586,73 | 47010,05 | 40121,65 |
| **RNF43_659wt_** | QRKRRGGPS | 45761,93 | 46907,41 | 42960,28 | 45574,14 | 43112,07 | 29422,40 | 6767,11 | 11134,32 | 36125,41 | 44807,01 | 45827,82 | 38181,08 |
| **RNF43_659wt_** | RKRRGGPSE | 46705,35 | 45442,19 | 34061,95 | 45910,20 | 45200,45 | 19173,33 | 21136,85 | 12908,20 | 38138,97 | 38040,05 | 41165,12 | 27804,30 |
| **RNF43_659wt_** | KRRGGPSEP | 46936,34 | 46488,53 | 42294,74 | 46160,24 | 46914,01 | 31810,26 | 34965,96 | 15673,47 | 39341,43 | 41490,20 | 43047,28 | 39286,56 |
| **RNF43_659wt_** | RRGGPSEPT | 45164,26 | 41297,20 | 41749,13 | 40946,13 | 45347,89 | 29978,98 | 34460,05 | 5527,31 | 19559,50 | 36213,47 | 39603,24 | 39596,37 |
| **RNF43_659wt_** | RGGPSEPTP | 45410,73 | 41170,03 | 43108,79 | 41536,92 | 48417,22 | 40535,31 | 42324,04 | 42959,34 | 43947,09 | 43058,91 | 25860,86 | 41022,41 |
| **RNF43_659wt_** | GGPSEPTPG | 45451,04 | 43906,21 | 45885,37 | 44373,28 | 47462,85 | 41995,14 | 41910,72 | 44389,13 | 43995,12 | 45125,68 | 40017,18 | 38511,34 |
| **RNF43_659wt_** | GPSEPTPGS | 43027,24 | 41481,24 | 41074,38 | 47136,85 | 42298,86 | 8346,36 | 38918,06 | 41612,50 | 35229,14 | 40540,12 | 38908,38 | 41718,88 |
|  |  |  |  |  |  |  |  |  |  |  |  |  |  |
| **RFN43_G659Vfs*41_** | HPQRKRRGV | 39833,57 | 42190,54 | 39904,33 | 42817,33 | 38548,44 | 73,06 | 160,65 | 27511,63 | 19359,49 | 41886,24 | 42010,14 | 41463,75 |
| **RFN43_G659Vfs*41_** | PQRKRRGVP | 46518,22 | 47508,57 | 44978,46 | 45316,50 | 46991,22 | 35033,00 | 19748,34 | 36771,39 | 45104,70 | 44548,39 | 46540,88 | 35441,32 |
| **RFN43_G659Vfs*41_** | QRKRRGVPP | 45567,24 | 46433,25 | 42765,01 | 44360,32 | 45218,06 | 31334,06 | 5419,64 | 8433,96 | 28887,10 | 40384,73 | 45026,18 | 37457,64 |
| **RFN43_G659Vfs*41_** | RKRRGVPPS | 45136,91 | 41133,52 | 30589,08 | 41762,25 | 42894,30 | 14353,81 | 14670,98 | 8278,28 | 32222,82 | 33102,03 | 38316,82 | 23467,40 |
| **RFN43_G659Vfs*41_** | KRRGVPPSP | 46938,37 | 44656,97 | 39920,31 | 44172,10 | 46617,99 | 29942,35 | 32710,39 | 6824,24 | 36567,04 | 39486,84 | 39546,30 | 37458,85 |
| **RFN43_G659Vfs*41_** | RRGVPPSPP | 45106,16 | 43927,11 | 40662,24 | 42021,96 | 46542,38 | 32682,10 | 31948,23 | 7189,73 | 28352,96 | 36004,84 | 39967,84 | 39116,06 |
| **RFN43_G659Vfs*41_** | RGVPPSPPL | 37223,31 | 11735,09 | 21750,95 | 17852,29 | 36972,84 | 768,56 | 15819,65 | 19496,96 | 7242,90 | 14383,66 | 3360,78 | 3442,86 |
| **RFN43_G659Vfs*41_** | GVPPSPPLA | 32357,68 | 6907,66 | 28031,46 | 34624,12 | 32314,65 | 32613,92 | 38849,07 | 38160,43 | 37973,43 | 36457,62 | 33971,76 | 27279,83 |
| **RFN43_G659Vfs*41_** | VPPSPPLAL | 35640,52 | 27552,15 | 34270,81 | 27048,82 | 31352,72 | 84,22 | 9163,54 | 34679,25 | 2734,68 | 24715,97 | 31419,27 | 27713,89 |
| **RFN43_G659Vfs*41_** | PPSPPLALG | 42931,45 | 42442,34 | 40623,11 | 43290,15 | 44688,38 | 25641,31 | 38817,14 | 41745,07 | 40802,84 | 45384,20 | 31176,82 | 41871,74 |
| **RFN43_G659Vfs*41_** | PSPPLALGP | 37816,80 | 40421,00 | 43081,30 | 40712,89 | 45904,75 | 45449,58 | 44918,17 | 45131,07 | 45456,94 | 46150,27 | 34793,12 | 42950,50 |
| **RFN43_G659Vfs*41_** | SPPLALGPR | 37553,40 | 38574,73 | 23309,24 | 40355,44 | 17679,87 | 10321,39 | 26738,93 | 31315,09 | 33439,32 | 40650,39 | 40021,93 | 35451,28 |
| **RFN43_G659Vfs*41_** | PPLALGPRM | 42265,02 | 39519,35 | 40648,19 | 40679,87 | 41827,37 | 5809,74 | 26078,62 | 37075,80 | 29952,72 | 40912,03 | 34793,50 | 38554,29 |
| **RFN43_G659Vfs*41_** | PLALGPRMQ | 42636,57 | 39811,17 | 35916,13 | 46941,43 | 45082,25 | 44860,36 | 43494,38 | 44588,42 | 47543,54 | 47078,25 | 44076,15 | 38453,87 |
| **RFN43_G659Vfs*41_** | LALGPRMQL | 35059,92 | 11491,71 | 26776,57 | 25010,55 | 28856,19 | 491,66 | 5325,13 | 18324,32 | 6240,94 | 20776,54 | 2239,81 | 9183,10 |
| **RFN43_G659Vfs*41_** | ALGPRMQLC | 34635,76 | 3867,74 | 21650,22 | 30132,79 | 39874,54 | 33042,99 | 14815,34 | 36387,48 | 39818,49 | 41093,48 | 30358,28 | 25131,25 |
| **RFN43_G659Vfs*41_** | LGPRMQLCT | 35995,11 | 36245,22 | 40534,42 | 37120,76 | 45893,33 | 39421,96 | 28145,11 | 38319,71 | 38255,52 | 44619,78 | 32868,63 | 38044,18 |
| **RFN43_G659Vfs*41_** | GPRMQLCTQ | 43531,11 | 44471,34 | 35383,08 | 46585,71 | 41102,83 | 3534,43 | 14927,01 | 33972,51 | 42110,26 | 43233,52 | 40103,00 | 34688,25 |
| **RFN43_G659Vfs*41_** | PRMQLCTQL | 39863,33 | 33639,29 | 41271,71 | 24740,59 | 40368,12 | 29720,93 | 13136,16 | 773,13 | 4573,60 | 28842,44 | 37728,94 | 30801,62 |
| **RFN43_G659Vfs*41_** | RMQLCTQLA | 15828,04 | 440,28 | 5531,43 | 13074,91 | 37310,41 | 9653,90 | 5907,62 | 5479,73 | 19398,49 | 21172,78 | 13201,71 | 2334,98 |
| **RFN43_G659Vfs*41_** | MQLCTQLAR | 27454,85 | 10941,45 | 2069,72 | 25917,72 | 30614,57 | 26488,40 | 17631,53 | 525,50 | 10551,05 | 19525,04 | 17411,62 | 7444,23 |
| **RFN43_G659Vfs*41_** | QLCTQLARF | 22193,84 | 14464,67 | 19769,93 | 7902,73 | 12170,36 | 33167,63 | 17965,65 | 23962,34 | 35596,57 | 34569,48 | 9816,43 | 629,07 |
| **RFN43_G659Vfs*41_** | LCTQLARFF | 29256,06 | 35005,34 | 37572,90 | 23617,44 | 36683,96 | 34489,89 | 25078,82 | 31956,52 | 40216,83 | 36144,96 | 2533,86 | 13811,44 |
| **RFN43_G659Vfs*41_** | CTQLARFFP | 16760,96 | 20583,67 | 23171,19 | 26637,87 | 37295,06 | 33041,93 | 23392,37 | 30230,76 | 35229,50 | 36275,43 | 9570,19 | 30629,48 |
| **RFN43_G659Vfs*41_** | TQLARFFPI | 20921,15 | 35,39 | 12274,17 | 429,33 | 19908,60 | 9730,88 | 48,47 | 561,27 | 87,73 | 1563,53 | 8403,09 | 449,87 |
| **RFN43_G659Vfs*41_** | QLARFFPIT | 30882,04 | 1305,38 | 21867,29 | 31868,82 | 31677,34 | 31503,70 | 13376,55 | 27872,68 | 23936,95 | 30957,65 | 27371,19 | 12790,03 |
| **RFN43_G659Vfs*41_** | LARFFPITP | 37218,08 | 28163,99 | 30077,09 | 37874,96 | 40779,89 | 20631,16 | 14182,61 | 36974,04 | 33343,58 | 34364,01 | 10095,30 | 16289,85 |
| **RFN43_G659Vfs*41_** | ARFFPITPP | 43202,19 | 31059,32 | 33622,55 | 37348,38 | 41296,74 | 33703,39 | 30410,54 | 2075,13 | 9785,47 | 21036,00 | 35546,93 | 29420,82 |
| **RFN43_G659Vfs*41_** | RFFPITPPV | 31895,04 | 260,64 | 8262,89 | 1040,58 | 28615,22 | 8961,27 | 11360,79 | 8155,41 | 8346,45 | 13260,12 | 10614,83 | 7145,99 |
| **RFN43_G659Vfs*41_** | FFPITPPVW | 29258,29 | 26394,85 | 36566,64 | 175,64 | 25717,44 | 28287,68 | 19882,98 | 29025,27 | 26866,24 | 31971,73 | 3996,29 | 16200,56 |
| **RFN43_G659Vfs*41_** | FPITPPVWH | 36888,12 | 40763,12 | 30226,51 | 46034,55 | 23350,89 | 8800,90 | 24987,29 | 33582,91 | 24870,76 | 35733,18 | 34323,87 | 26505,61 |
| **RFN43_G659Vfs*41_** | PITPPVWHI | 35722,35 | 9267,74 | 34410,51 | 12028,45 | 36125,41 | 38331,75 | 37825,41 | 40291,75 | 39916,43 | 41864,04 | 16950,81 | 35779,62 |
| **RFN43_G659Vfs*41_** | ITPPVWHIL | 16037,84 | 4037,71 | 27306,71 | 404,60 | 10967,88 | 13822,65 | 18959,62 | 26847,07 | 14268,04 | 19342,74 | 2863,03 | 10389,17 |
| **RFN43_G659Vfs*41_** | TPPVWHILG | 38094,01 | 35948,41 | 37818,85 | 31782,04 | 39569,39 | 14261,71 | 22078,64 | 36210,34 | 29021,50 | 40372,93 | 33733,32 | 39074,18 |
| **RFN43_G659Vfs*41_** | PPVWHILGP | 44490,59 | 41014,42 | 42849,32 | 45141,31 | 44446,80 | 25394,76 | 34834,18 | 40820,94 | 39061,91 | 43976,09 | 39423,26 | 42996,06 |
| **RFN43_G659Vfs*41_** | PVWHILGPQ | 40209,00 | 31495,86 | 27178,49 | 44452,10 | 35878,84 | 39300,60 | 40436,30 | 37948,80 | 45802,53 | 45346,43 | 37885,62 | 33471,91 |
| **RFN43_G659Vfs*41_** | VWHILGPQR | 43862,06 | 40111,67 | 21432,07 | 27081,34 | 42189,14 | 41207,91 | 36021,61 | 23910,03 | 45065,66 | 45193,10 | 41869,93 | 38525,08 |
| **RFN43_G659Vfs*41_** | WHILGPQRH | 38514,25 | 43965,63 | 35834,23 | 39944,93 | 38350,00 | 39684,75 | 36917,28 | 21491,29 | 11737,89 | 35951,91 | 39218,62 | 26487,55 |
| **RFN43_G659Vfs*41_** | HILGPQRHT | 43479,80 | 30057,58 | 30995,53 | 45830,30 | 37387,58 | 28360,93 | 33319,08 | 37878,24 | 40332,75 | 43551,36 | 34653,36 | 30856,34 |
| **RFN43_G659Vfs*41_** | ILGPQRHTP | 39686,46 | 18375,94 | 34684,13 | 35324,94 | 45548,52 | 31711,96 | 19249,00 | 40767,98 | 38809,16 | 41618,81 | 34038,71 | 26805,27 |
|  |  |  |  |  |  |  |  |  |  |  |  |  |  |
| **PROTEIN** | **PEPTIDE** | **A*01:01** | **A*02:01** | **A*03:01** | **A*24:02** | **A*26:02** | **B*07:02** | **B*08:02** | **B*27:05** | **B*39:01** | **B*40:01** | **B*58:01** | **B*15:01** |
| **JAK1_860wt_** | NPDIVSEKK | 31575,36 | 45031,55 | 31563,75 | 45439,73 | 41400,07 | 28983,22 | 36257,00 | 40477,89 | 37797,59 | 43144,72 | 38804,53 | 43251,31 |
| **JAK1_860wt_** | PDIVSEKKP | 48136,73 | 48702,52 | 48185,19 | 48539,43 | 49107,32 | 48159,65 | 47809,22 | 47814,38 | 48600,42 | 47586,27 | 47874,94 | 48296,91 |
| **JAK1_860wt_** | DIVSEKKPA | 43102,73 | 37839,32 | 41830,98 | 46816,66 | 24853,27 | 30582,45 | 21236,56 | 43563,16 | 35007,98 | 44054,21 | 43903,83 | 35721,20 |
| **JAK1_860wt_** | IVSEKKPAT | 41013,10 | 26538,33 | 34883,96 | 45170,14 | 41550,42 | 20747,57 | 13053,85 | 41704,44 | 34868,12 | 40456,88 | 35841,61 | 30444,78 |
| **JAK1_860wt_** | VSEKKPATE | 30958,33 | 41604,86 | 37777,95 | 45410,26 | 45834,76 | 38330,09 | 30385,87 | 43473,68 | 42046,52 | 44508,87 | 28341,29 | 38663,72 |
| **JAK1_860wt_** | SEKKPATEV | 37631,91 | 33886,23 | 38009,62 | 39182,14 | 31844,68 | 20408,27 | 16469,55 | 33457,79 | 19740,86 | 746,08 | 39775,02 | 14385,84 |
| **JAK1_860wt_** | EKKPATEVD | 47812,83 | 48114,86 | 47756,99 | 47370,50 | 46840,97 | 44076,60 | 37767,74 | 42812,70 | 37219,28 | 44677,28 | 44001,79 | 40271,70 |
| **JAK1_860wt_** | KKPATEVDP | 47671,82 | 46740,21 | 47153,18 | 47328,51 | 48318,31 | 46115,31 | 46187,21 | 43732,22 | 44439,12 | 42475,42 | 44824,47 | 44971,64 |
| **JAK1_860wt_** | KPATEVDPT | 40689,97 | 35080,03 | 39342,29 | 44636,67 | 42845,15 | 3750,09 | 30774,97 | 38851,58 | 23133,62 | 39144,43 | 32436,90 | 37018,87 |
|  |  |  |  |  |  |  |  |  |  |  |  |  |  |
| **JAK1_K860Nfs*16_** | NPDIVSEKN | 39765,96 | 46105,85 | 44545,48 | 46967,83 | 46582,20 | 34886,23 | 39741,89 | 44184,04 | 39645,69 | 45601,78 | 37566,82 | 44488,18 |
| **JAK1_K860Nfs*16_** | PDIVSEKNQ | 47934,57 | 48560,47 | 47287,05 | 49183,36 | 48610,92 | 48743,11 | 48296,38 | 47309,56 | 48923,82 | 48655,11 | 47797,32 | 47718,79 |
| **JAK1_K860Nfs*16_** | DIVSEKNQQ | 44938,10 | 45910,20 | 42040,61 | 48470,71 | 30568,57 | 44293,17 | 43021,19 | 46505,66 | 46301,29 | 47582,16 | 45586,99 | 42927,27 |
| **JAK1_K860Nfs*16_** | IVSEKNQQL | 29988,71 | 3228,48 | 29622,69 | 23872,04 | 24317,55 | 7713,66 | 4131,31 | 32663,36 | 15025,21 | 24546,48 | 12577,59 | 9673,25 |
| **JAK1_K860Nfs*16_** | VSEKNQQLK | 6913,49 | 40906,72 | 4806,31 | 41063,25 | 41902,10 | 41042,39 | 40844,80 | 34664,25 | 44180,71 | 43307,97 | 17650,81 | 35973,69 |
| **JAK1_K860Nfs*16_** | SEKNQQLKW | 34033,58 | 44310,91 | 40820,52 | 29247,84 | 35486,99 | 37122,77 | 32703,33 | 27361,73 | 38773,91 | 19680,30 | 11687,96 | 20359,07 |
| **JAK1_K860Nfs*16_** | EKNQQLKWT | 46442,80 | 47280,40 | 46388,57 | 47753,39 | 43997,05 | 44277,86 | 33426,30 | 37464,94 | 39760,37 | 45958,90 | 45795,61 | 44390,57 |
| **JAK1_K860Nfs*16_** | KNQQLKWTP | 42097,50 | 40214,65 | 40517,33 | 33775,68 | 46735,15 | 41594,04 | 31266,68 | 29904,14 | 34078,89 | 36148,11 | 24317,55 | 39990,76 |
| **JAK1_K860Nfs*16_** | NQQLKWTPH | 34005,61 | 38651,18 | 24430,43 | 36676,41 | 33712,89 | 29896,70 | 9750,06 | 14979,11 | 18891,22 | 27981,45 | 40196,84 | 4818,71 |
| **JAK1_K860Nfs*16_** | QQLKWTPHI | 30115,85 | 182,70 | 23529,69 | 2039,58 | 26623,17 | 27129,42 | 14656,23 | 1843,22 | 3619,53 | 5760,36 | 7383,67 | 1412,25 |
| **JAK1_K860Nfs*16_** | QLKWTPHIL | 35355,14 | 6570,57 | 25383,77 | 16443,38 | 30898,43 | 9790,87 | 935,95 | 26251,32 | 10597,16 | 21880,06 | 24841,17 | 3135,23 |
| **JAK1_K860Nfs*16_** | LKWTPHILK | 39565,54 | 35268,42 | 4820,06 | 37558,30 | 43644,29 | 37461,70 | 33838,60 | 1127,03 | 32793,68 | 33502,36 | 32114,92 | 30124,65 |
| **JAK1_K860Nfs*16_** | KWTPHILKS | 42837,27 | 35143,86 | 26418,87 | 15083,68 | 43657,52 | 39606,66 | 35266,89 | 25763,39 | 43778,61 | 43896,25 | 34008,17 | 35750,97 |
| **JAK1_K860Nfs*16_** | WTPHILKSA | 25490,55 | 11028,81 | 35321,11 | 35187,61 | 7493,93 | 23180,72 | 16803,99 | 31045,54 | 28237,85 | 35855,57 | 28524,66 | 19768,00 |
| **JAK1_K860Nfs*16_** | TPHILKSAS | 42441,90 | 44536,35 | 39390,43 | 45920,62 | 42247,63 | 774,77 | 6902,80 | 35128,66 | 26342,65 | 42734,49 | 41608,91 | 37980,83 |
|  |  |  |  |  |  |  |  |  |  |  |  |  |  |
| **PROTEIN** | **PEPTIDE** | **A*01:01** | **A*02:01** | **A*03:01** | **A*24:02** | **A*26:02** | **B*07:02** | **B*08:02** | **B*27:05** | **B*39:01** | **B*40:01** | **B*58:01** | **B*15:01** |
| **UBR5_2121wt_** | NMQNRQKKE | 44302,28 | 41867,66 | 39902,6 | 45279,25 | 45252,8 | 40487,08 | 12880,85 | 38460,12 | 43794,25 | 46205,21 | 43969,43 | 38887,75 |
| **UBR5_2121wt_** | MQNRQKKEG | 43593,79 | 44110,5 | 41932,49 | 43273,77 | 45613,13 | 36739,55 | 16771,67 | 25954,49 | 34877,16 | 37239,83 | 33531,36 | 17179,39 |
| **UBR5_2121wt_** | QNRQKKEGE | 47189,93 | 48170,59 | 45079,81 | 48215,43 | 46860,21 | 43816,99 | 26711,75 | 43719,92 | 46725,56 | 47915,38 | 45291,98 | 44940,54 |
| **UBR5_2121wt_** | NRQKKEGEE | 47172,07 | 47895,17 | 46693,72 | 47066,02 | 47613,56 | 42855,35 | 28308,2 | 29277,28 | 35846,25 | 47075,18 | 46629,09 | 46062,96 |
| **UBR5_2121wt_** | RQKKEGEEQ | 44532,47 | 42906,37 | 35841,21 | 44948,8 | 43491,55 | 38579,73 | 33826,88 | 23639,67 | 40452,06 | 31593,81 | 38224,07 | 6611,79 |
| **UBR5_2121wt_** | QKKEGEEQP | 48014,48 | 47945,98 | 48146,64 | 48276,51 | 47914,85 | 46019,11 | 41969,25 | 43635,79 | 41016,64 | 41016,19 | 45006,23 | 42357,93 |
| **UBR5_2121wt_** | KKEGEEQPV | 43604,16 | 38152,18 | 44992,1 | 44959 | 45846,18 | 38743,29 | 39404,47 | 34629,37 | 19214,46 | 29609,23 | 41695,43 | 38999,41 |
| **UBR5_2121wt_** | KEGEEQPVL | 40535,31 | 28303,61 | 42425,81 | 36601,49 | 41500,98 | 35068,65 | 31873,65 | 34690,88 | 15806,83 | 48,41 | 32749,7 | 22439,89 |
| **UBR5_2121wt_** | EGEEQPVLP | 41430,55 | 45757,47 | 46929,74 | 46877,99 | 47202,68 | 45323,37 | 42453,37 | 46972,92 | 43022,61 | 45235,68 | 41254,31 | 46953,11 |
|  |  |  |  |  |  |  |  |  |  |  |  |  |  |
| **UBR5_E2121Kfs*28_** | NMQNRQKKK | 37847,91 | 39352,08 | 3131,8 | 41994,24 | 40935,07 | 39191,03 | 21016,66 | 24590,34 | 43668,86 | 45391,58 | 42066,54 | 35211,99 |
| **UBR5_E2121Kfs*28_** | MQNRQKKKG | 44423,24 | 45417,11 | 42077,01 | 43714,23 | 46039,53 | 40410,5 | 20754,53 | 25713,83 | 39439,02 | 40078,26 | 34270,06 | 23440 |
| **UBR5_E2121Kfs*28_** | QNRQKKKGK | 46009,67 | 48254,07 | 31260,92 | 47751,82 | 45673,86 | 43302,32 | 31178,17 | 37714,66 | 47581,64 | 47912,25 | 46104,34 | 42972,83 |
| **UBR5_E2121Kfs*28_** | NRQKKKGKN | 47513,2 | 48780,06 | 47414,6 | 47736,85 | 48124,23 | 45348,86 | 33512,86 | 25685,19 | 44544,05 | 48243,11 | 46684,63 | 46872,91 |
| **UBR5_E2121Kfs*28_** | RQKKKGKNS | 46082,9 | 43641,92 | 32779,49 | 44066,12 | 44179,74 | 32173 | 11447,41 | 16980,37 | 42780,76 | 38350,42 | 43316,4 | 17314,69 |
| **UBR5_E2121Kfs*28_** | QKKKGKNSP | 48139,34 | 48383,2 | 47558,48 | 47986,44 | 47878,06 | 41823,3 | 28642,79 | 41121,05 | 42492,43 | 44148,21 | 46602,87 | 41266,82 |
| **UBR5_E2121Kfs*28_** | KKKGKNSPC | 46895,22 | 44092,36 | 41652,13 | 45697,11 | 46179,74 | 23855,51 | 11085,27 | 21498,26 | 34969,37 | 40943,49 | 42856,26 | 33095,95 |
| **UBR5_E2121Kfs*28_** | KKGKNSPCC | 46653,3 | 44405 | 44347,36 | 44100,46 | 48053,98 | 41238,7 | 31343,91 | 33249,21 | 37273,28 | 41818,77 | 40003,76 | 41742,38 |
| **UBR5_E2121Kfs*28_** | KGKNSPCCQ | 45272,39 | 46296,78 | 36096,91 | 47326,95 | 47274,24 | 42146,72 | 39306,99 | 41093,48 | 47342,83 | 47115,94 | 37834,41 | 36704,2 |
| **UBR5_E2121Kfs*28_** | GKNSPCCQK | 43643,83 | 42331,36 | 15993,13 | 45561,33 | 44758,55 | 42059,73 | 40655,21 | 9764,95 | 41933,41 | 40445,93 | 41058,83 | 34640,98 |
| **UBR5_E2121Kfs*28_** | KNSPCCQKK | 37734,24 | 42764,1 | 8188,48 | 39125,8 | 43463,34 | 42070,2 | 42941,22 | 23379,71 | 44849,22 | 44973,62 | 28172,53 | 38279,53 |
| **UBR5_E2121Kfs*28_** | NSPCCQKKL | 31979,36 | 35196,37 | 40861,61 | 29731,86 | 33933,93 | 29119,63 | 24360,74 | 36110,18 | 27444,44 | 38254,26 | 26840,09 | 32561,38 |
| **UBR5_E2121Kfs*28_** | SPCCQKKLR | 40900,07 | 45745,08 | 33508,52 | 45431,38 | 40205,52 | 26906,68 | 31805,79 | 35715,78 | 43036,09 | 45985,77 | 43256,44 | 44253,91 |
| **UBR5_E2121Kfs*28_** | PCCQKKLRV | 41811,08 | 44135,8 | 44709,19 | 44953,67 | 48087,28 | 45198,99 | 36383,14 | 45035,44 | 46963,26 | 47856,3 | 43511,8 | 46613,45 |
| **UBR5_E2121Kfs*28_** | CCQKKLRVQ | 43919,04 | 44654,55 | 39029,38 | 46331,37 | 46300,81 | 40013,28 | 23476,03 | 38830,16 | 45200,45 | 46355,94 | 38928,18 | 40355,02 |
| **UBR5_E2121Kfs*28_** | CQKKLRVQN | 41576,93 | 43139,6 | 33663,69 | 41970,18 | 43702,89 | 36562,3 | 18005,34 | 16820,18 | 40895,22 | 39964,39 | 35615,84 | 14151,34 |
| **UBR5_E2121Kfs*28_** | QKKLRVQNQ | 47691,93 | 47896,71 | 43534,4 | 47808,18 | 45945,98 | 44851,62 | 18281,34 | 37090,63 | 44734,34 | 45602,75 | 45925,6 | 39787,07 |
| **UBR5_E2121Kfs*28_** | KKLRVQNQG | 47145,52 | 45570,71 | 42884,1 | 44541,63 | 47134,82 | 42380,39 | 36206,42 | 15065,9 | 40202,49 | 40998,45 | 29429,1 | 39069,52 |
| **UBR5_E2121Kfs*28_** | KLRVQNQGH | 39495,4 | 38229,03 | 2905,06 | 44890,48 | 40791,81 | 25709,93 | 27027,75 | 27585,56 | 45330,23 | 39803,42 | 35665,58 | 6659,25 |
| **UBR5_E2121Kfs*28_** | LRVQNQGHL | 39385,29 | 36888,53 | 40334,94 | 36599,5 | 33731,86 | 26433,15 | 16594,58 | 882,02 | 3286,88 | 25946,34 | 33132,12 | 21787,22 |
| **UBR5_E2121Kfs*28_** | RVQNQGHLL | 23681,15 | 7524,4 | 13372,35 | 6054,7 | 25582,28 | 788,49 | 7702,99 | 6679,09 | 12940,93 | 15090,05 | 5939,85 | 3848,08 |
| **UBR5_E2121Kfs*28_** | VQNQGHLLM | 15941,65 | 7510,65 | 16071,19 | 8562,97 | 24230,62 | 13912,22 | 10767,75 | 5315,29 | 932,08 | 2256,74 | 7861,79 | 37,86 |
| **UBR5_E2121Kfs*28_** | QNQGHLLMI | 24472,49 | 24502,97 | 31091,93 | 16935,41 | 26373,74 | 28887,73 | 12112,56 | 24069,66 | 16036,1 | 26366,59 | 19338,13 | 26988,6 |
| **UBR5_E2121Kfs*28_** | NQGHLLMIL | 30256,62 | 4043,66 | 32837,7 | 14593,89 | 30162,48 | 23569,95 | 6010,12 | 10547,05 | 529,29 | 5805,85 | 26213,27 | 1582,97 |
| **UBR5_E2121Kfs*28_** | QGHLLMILL | 35180,01 | 19393,66 | 33782,63 | 23384,52 | 38417,7 | 27379,79 | 9154,73 | 25856,66 | 18595,34 | 28149,38 | 13014,92 | 19858,04 |
| **UBR5_E2121Kfs*28_** | GHLLMILLH | 36131,68 | 38007,14 | 24173,53 | 34060,1 | 39385,29 | 42974,69 | 36569,42 | 8997,02 | 23841,31 | 33873,77 | 35292,86 | 27745,1 |
| **UBR5_E2121Kfs*28_** | HLLMILLHN | 36048,5 | 11815,36 | 18323,13 | 39249,61 | 40580,51 | 41684,59 | 21869,41 | 27694,7 | 41653,5 | 42921,7 | 19938,34 | 20106,89 |
|  |  |  |  |  |  |  |  |  |  |  |  |  |  |
| **PROTEIN** | **PEPTIDE** | **A*01:01** | **A*02:01** | **A*03:01** | **A*24:02** | **A*26:02** | **B*07:02** | **B*08:02** | **B*27:05** | **B*39:01** | **B*40:01** | **B*58:01** | **B*15:01** |
| **LARP4B_163wt_** | DPREVLKKT | 45788,17 | 46270,25 | 44593,23 | 47409,46 | 38563,04 | 18709,98 | 19670,5 | 43000,72 | 41816,5 | 46398,59 | 46358,96 | 44477,11 |
| **LARP4B_163wt_** | PREVLKKTL | 44774,54 | 45755,5 | 46244,74 | 42176,39 | 47173,07 | 39839,61 | 27372,97 | 19421,37 | 23806,26 | 41037,49 | 45016,45 | 44664,69 |
| **LARP4B_163wt_** | REVLKKTLE | 43702,89 | 43550,43 | 39357,62 | 44682,58 | 44689,36 | 41228,88 | 29106,73 | 24588,75 | 39635,38 | 15851,52 | 40484,47 | 37005,26 |
| **LARP4B_163wt_** | EVLKKTLEF | 22371,29 | 29594,81 | 24427,79 | 6772,23 | 297,94 | 13110,33 | 901,93 | 26721,58 | 23589,34 | 30175,54 | 9536,39 | 2755,77 |
| **LARP4B_163wt_** | VLKKTLEFC | 38160,84 | 5506,41 | 26725,33 | 32239,9 | 40718,16 | 34775,06 | 13611,76 | 35294,38 | 41055,25 | 40380,35 | 29582,33 | 15311,42 |
| **LARP4B_163wt_** | LKKTLEFCL | 39660,7 | 32373,79 | 40638,94 | 34890,01 | 43278,9 | 30756,01 | 2956,84 | 10613,45 | 6191,17 | 23413,12 | 31825,75 | 19328,3 |
| **LARP4B_163wt_** | KKTLEFCLS | 42748,82 | 39534,75 | 39971,29 | 43189,09 | 45695,63 | 41536,92 | 36113,7 | 19731,04 | 36153,57 | 37476,3 | 34043,88 | 38120,41 |
| **LARP4B_163wt_** | KTLEFCLSR | 24628,43 | 12797,09 | 309,64 | 26490,42 | 30276,59 | 33434,99 | 29868,89 | 8022,48 | 37238,63 | 34628,63 | 12840,92 | 23410,09 |
| **LARP4B_163wt_** | TLEFCLSRE | 27842,54 | 23160,91 | 27365,58 | 42679,52 | 40679,42 | 40327,96 | 36153,2 | 37987,42 | 43594,27 | 44061,34 | 40084,34 | 32844,81 |
|  |  |  |  |  |  |  |  |  |  |  |  |  |  |
| **LARP4B_T163Hfs*47_** | DPREVLKKH | 45791,16 | 47964,66 | 43135,4 | 48323,55 | 32681,38 | 28768,58 | 30552,35 | 43386,27 | 43904,79 | 45841,71 | 46289,29 | 39239,41 |
| **LARP4B_T163Hfs*47_** | PREVLKKHW | 46038,55 | 48175,28 | 47379,23 | 41134,87 | 47571,33 | 46720,5 | 42482,32 | 28986,36 | 44885,13 | 47149,11 | 35548,08 | 45154,01 |
| **LARP4B_T163Hfs*47_** | REVLKKHWN | 44297,48 | 45694,63 | 42144 | 43712,82 | 45654,6 | 42041,53 | 32323,39 | 24697,8 | 42331,36 | 23886,49 | 37890,53 | 41094,38 |
| **LARP4B_T163Hfs*47_** | EVLKKHWNS | 36321,79 | 25870,93 | 32201,57 | 36752,68 | 20924,32 | 35190,26 | 5409,57 | 34119,11 | 33191,68 | 42669,8 | 32707,57 | 37943,45 |
| **LARP4B_T163Hfs*47_** | VLKKHWNSA | 30030,92 | 5226,21 | 14160,07 | 26932,6 | 26226,9 | 6484,76 | 59,58 | 21503,61 | 26517,08 | 34760,4 | 34033,93 | 3090,36 |
| **LARP4B_T163Hfs*47_** | LKKHWNSAY | 28537,93 | 42665,18 | 30941,58 | 40436,75 | 20310,01 | 29466,68 | 20633,17 | 11527,82 | 25076,12 | 33543,69 | 34489,89 | 639,84 |
| **LARP4B_T163Hfs*47_** | KKHWNSAYL | 38462,2 | 21222,31 | 27925,79 | 29597,06 | 37608,3 | 14227,33 | 12613,44 | 5072,45 | 2209,67 | 10845,27 | 20830,8 | 10327,64 |
| **LARP4B_T163Hfs*47_** | KHWNSAYLG | 37681,62 | 25791,84 | 31027,07 | 14990,95 | 44752,26 | 36089,88 | 32003,23 | 6758,91 | 6961,76 | 25267,58 | 13506,86 | 31556,24 |
| **LARP4B_T163Hfs*47_** | HWNSAYLGR | 40849,23 | 37446,29 | 14084,59 | 23870,22 | 37870,03 | 40654,77 | 35942,96 | 21744,6 | 42474,05 | 43676,41 | 38209,61 | 36685,93 |
| **LARP4B_T163Hfs*47_** | WNSAYLGRT | 30435,88 | 36096,51 | 39088,55 | 41558,95 | 36693,07 | 39445,87 | 27117,98 | 31496,89 | 35168,57 | 38573,06 | 32313,95 | 36013,04 |
| **LARP4B_T163Hfs*47_** | NSAYLGRTL | 23229,69 | 23946,8 | 33582,91 | 23230,19 | 13890,72 | 3790,31 | 7411,04 | 22048,56 | 3138,18 | 21443,91 | 4865,7 | 6350,81 |
| **LARP4B_T163Hfs*47_** | SAYLGRTLL | 24221,45 | 9143,93 | 15853,24 | 15389,48 | 16545,64 | 216,94 | 1227,92 | 12544,57 | 2234,53 | 11215,82 | 3558,45 | 2576,08 |
| **LARP4B_T163Hfs*47_** | AYLGRTLLV | 29481,36 | 8214,93 | 18852,83 | 156,98 | 28491,35 | 23982,32 | 11304,5 | 12422,06 | 21496,87 | 32567,03 | 26492,42 | 25319,58 |
| **LARP4B_T163Hfs*47_** | YLGRTLLVT | 20904,4 | 245,57 | 26293,66 | 30397,04 | 36295,07 | 25998,33 | 7657,28 | 26067,9 | 21030,99 | 35801,29 | 28075,15 | 9371,3 |
| **LARP4B_T163Hfs*47_** | LGRTLLVTC | 42519,11 | 30505,11 | 36530,27 | 39163,49 | 46059,96 | 25597,79 | 10692,06 | 34463,79 | 35665,19 | 40436,3 | 20939,03 | 24201,28 |
| **LARP4B_T163Hfs*47_** | GRTLLVTCI | 36385,51 | 26218,96 | 37108,69 | 27098,63 | 39333,36 | 32875,03 | 15629,44 | 152,11 | 4353,47 | 20329,37 | 29780,47 | 27310,55 |
| **LARP4B_T163Hfs*47_** | RTLLVTCIL | 17828,16 | 2403,1 | 10480,4 | 6564,67 | 29213,36 | 4437,31 | 8250,92 | 8474,58 | 10237,53 | 13372,5 | 122,98 | 1777,72 |
| **LARP4B_T163Hfs*47_** | TLLVTCILY | 5713,8 | 15944,23 | 1536,45 | 28037,82 | 19015,7 | 41746,44 | 29476,58 | 29445,33 | 39524,91 | 36283,67 | 16814,91 | 1761,52 |
| **LARP4B_T163Hfs*47_** | LLVTCILYH | 25436,28 | 11129,14 | 1181,2 | 42812,25 | 29125 | 39904,77 | 31711,64 | 27891,38 | 42260,44 | 40689,97 | 25752,53 | 6727,68 |
| **LARP4B_T163Hfs*47_** | LVTCILYHR | 28689 | 15619,64 | 4353,42 | 28956,26 | 18597,96 | 36529,47 | 30496,87 | 17644,31 | 38312,25 | 40079,99 | 20507,88 | 28842,14 |
| **LARP4B_T163Hfs*47_** | VTCILYHRW | 14369,66 | 31471,33 | 25539,42 | 3274,92 | 27506,28 | 39284,03 | 29543,63 | 28032,67 | 41011,3 | 39219,88 | 12,22 | 19106,24 |
| **LARP4B_T163Hfs*47_** | TCILYHRWI | 38077,53 | 26296,22 | 39845,21 | 31835,05 | 41319,52 | 33618,91 | 13312,3 | 36962,45 | 34010,74 | 37193,91 | 21846,94 | 35667,12 |
| **LARP4B_T163Hfs*47_** | CILYHRWIV | 25122,29 | 1045,19 | 15807,67 | 18072,29 | 28341 | 18957,36 | 590,33 | 20914,13 | 23657,07 | 35263,46 | 21676,71 | 20816,6 |
| **LARP4B_T163Hfs*47_** | ILYHRWIVT | 31611,95 | 725,21 | 8340,22 | 29307,71 | 37601,8 | 23006,81 | 1706,37 | 24953,78 | 23260,12 | 35597,36 | 30529,22 | 9139,68 |
| **LARP4B_T163Hfs*47_** | LYHRWIVTS | 37329,38 | 26008,75 | 29286,79 | 4024,71 | 36191,15 | 35086,11 | 19435,46 | 22963,55 | 27613,63 | 41296,29 | 32152,47 | 26515,36 |
| **LARP4B_T163Hfs*47_** | YHRWIVTSM | 27486,64 | 21328,21 | 27782,05 | 9453,38 | 17416,71 | 3029,32 | 2397,29 | 6956,11 | 71,78 | 15675,85 | 23967,54 | 3411,6 |
| **LARP4B_T163Hfs*47_** | HRWIVTSMC | 42530,16 | 32433,39 | 37194,32 | 36683,16 | 43516,96 | 31405,34 | 21040,33 | 416,59 | 8033,16 | 33418,35 | 34046,84 | 36080,89 |
| **LARP4B_T163Hfs*47_** | RWIVTSMCQ | 41812,43 | 36874,57 | 21430,68 | 21941,46 | 43119,52 | 38674,19 | 32220,03 | 22912,42 | 43406,48 | 42919,85 | 31628,71 | 25259,11 |
| **LARP4B_T163Hfs*47_** | WIVTSMCQS | 31430,16 | 13503,21 | 26714,36 | 42806,68 | 17642,22 | 28000,83 | 27154,98 | 32050,01 | 33024,39 | 40448,12 | 26878,73 | 13764 |
| **LARP4B_T163Hfs*47_** | IVTSMCQSQ | 37402,97 | 31765,88 | 27737,3 | 43001,66 | 37966,45 | 33851,79 | 34749,1 | 36220,52 | 42184,14 | 43429,97 | 24038,7 | 20691,98 |
| **LARP4B_T163Hfs*47_** | VTSMCQSQR | 19302,39 | 31266 | 2807,05 | 38742,44 | 25242,99 | 34458,57 | 37062,56 | 26302,21 | 42272,79 | 41738,31 | 19945,46 | 30024,74 |
| **LARP4B_T163Hfs*47_** | TSMCQSQRW | 13234,74 | 36351,27 | 29813,37 | 7442,94 | 18940,96 | 26276,9 | 21594,54 | 23793,12 | 33376,43 | 35661,72 | 10,43 | 10467,14 |
|  |  |  |  |  |  |  |  |  |  |  |  |  |  |
| **PROTEIN** | **PEPTIDE** | **A*01:01** | **A*02:01** | **A*03:01** | **A*24:02** | **A*26:02** | **B*07:02** | **B*08:02** | **B*27:05** | **B*39:01** | **B*40:01** | **B*58:01** | **B*15:01** |
| **SPECC1_303wt_** | SDIDEYKKN | 44605,3 | 46648,27 | 45943,49 | 46326,35 | 43272,37 | 46582,2 | 45441,23 | 44911,85 | 47555,9 | 44884,64 | 43093,89 | 44423,24 |
| **SPECC1_303wt_** | DIDEYKKNI | 30074,49 | 29898,32 | 42008,33 | 41839,13 | 31163,33 | 36603,47 | 22128,14 | 43168,07 | 34265,24 | 43260,65 | 42241,69 | 42942,13 |
| **SPECC1_303wt_** | IDEYKKNIH | 39395,96 | 48059,71 | 43595,2 | 48244,14 | 45938,52 | 45174,05 | 40493,66 | 45073,47 | 46134,29 | 43518,88 | 46436,28 | 43789,03 |
| **SPECC1_303wt_** | DEYKKNIHG | 45123,24 | 46647,77 | 44995,03 | 45206,8 | 43854,93 | 42522,78 | 29472,74 | 38947,56 | 37008,05 | 29531,15 | 44141,52 | 36662,53 |
| **SPECC1_303wt_** | EYKKNIHGN | 45933,07 | 47219,55 | 43069,16 | 30490,93 | 37159,32 | 44552,23 | 30141,27 | 41304,33 | 44047,54 | 47594,5 | 43964,69 | 42049,72 |
| **SPECC1_303wt_** | YKKNIHGNA | 42468,08 | 42433,17 | 40848,78 | 43398,5 | 38095,66 | 27972,37 | 8167,51 | 16670,71 | 22085,09 | 35256,21 | 43243,8 | 24266,83 |
| **SPECC1_303wt_** | KKNIHGNAL | 40369 | 36515,24 | 33749 | 36770,98 | 40422,75 | 5541,08 | 3314,02 | 8061,02 | 1738,61 | 9772,24 | 34836,81 | 9698,4 |
| **SPECC1_303wt_** | KNIHGNALR | 37070,59 | 36376,05 | 10830,73 | 38128,23 | 32722,78 | 39260,65 | 38495,93 | 19636,9 | 39263,18 | 39381,48 | 29087,52 | 33127,83 |
| **SPECC1_303wt_** | NIHGNALRT | 37606,67 | 28253,12 | 35491,98 | 43294,84 | 31527,58 | 32761,4 | 27331,25 | 38514,67 | 34572,09 | 43421,96 | 37610,34 | 34600,53 |
|  |  |  |  |  |  |  |  |  |  |  |  |  |  |
| **SPECC1_N303Tfs*63_** | SDIDEYKKT | 42613,51 | 41658,9 | 44436,22 | 44980,41 | 39480,45 | 43880,56 | 41739,2 | 44019,9 | 43799,45 | 38659,53 | 43963,73 | 43183,5 |
| **SPECC1_N303Tfs*63_** | DIDEYKKTY | 3586,28 | 40252,54 | 33393,42 | 44885,62 | 6555,16 | 38106,79 | 30243,53 | 41047,26 | 35586,57 | 42185,96 | 39712,22 | 21735,19 |
| **SPECC1_N303Tfs*63_** | IDEYKKTYM | 26244,21 | 40937,73 | 40465,21 | 41857,24 | 41261,02 | 33239,13 | 20241,78 | 40895,67 | 36163,76 | 27831,39 | 40170,3 | 35893,21 |
| **SPECC1_N303Tfs*63_** | DEYKKTYME | 43836,44 | 44627,03 | 42763,16 | 44225,18 | 40701,86 | 42679,05 | 23035,96 | 37899,96 | 31922,3 | 31015,32 | 45019,38 | 38652 |
| **SPECC1_N303Tfs*63_** | EYKKTYMEM | 36696,64 | 39850,84 | 35454,73 | 1757,22 | 11189,39 | 25449,49 | 4044,31 | 30267,74 | 19302,6 | 36026,67 | 35795,47 | 15480,84 |
| **SPECC1_N303Tfs*63_** | YKKTYMEMH | 42389,11 | 43637,22 | 40358,95 | 44484,82 | 36861,39 | 40645,99 | 23056,9 | 20430,57 | 30632,13 | 37323,33 | 42219,3 | 17861,18 |
| **SPECC1_N303Tfs*63_** | KKTYMEMHY | 28502,13 | 38528,01 | 21666,85 | 39277,64 | 35032,23 | 40943,04 | 39099,97 | 7329,7 | 33159,74 | 33140,73 | 13396,54 | 5343,66 |
| **SPECC1_N303Tfs*63_** | KTYMEMHYG | 24055,6 | 14429,18 | 11815,11 | 26861,01 | 30881,04 | 32406,38 | 29933,6 | 25060,93 | 38431,01 | 36945,24 | 434,62 | 9953,65 |
| **SPECC1_N303Tfs*63_** | TYMEMHYGH | 32378,34 | 33985,37 | 23710,12 | 6802,64 | 23909,77 | 34316,81 | 24373,4 | 17614,56 | 22877,99 | 35958,13 | 28541,94 | 20401,42 |
| **SPECC1_N303Tfs*63_** | YMEMHYGHQ | 16519,17 | 25180,24 | 24808,14 | 38563,86 | 31388,37 | 33923,64 | 14214,25 | 31391,75 | 34720,16 | 37672,65 | 35952,29 | 11590,23 |
| **SPECC1_N303Tfs*63_** | MEMHYGHQA | 24666,56 | 15159,76 | 31650,26 | 31481,22 | 26438,88 | 15305,79 | 5015,96 | 13302,08 | 2506,84 | 541,51 | 29382,96 | 11163,15 |
| **SPECC1_N303Tfs*63_** | EMHYGHQAP | 39286,13 | 31376,47 | 39186,38 | 38945,88 | 31177,16 | 26852,57 | 9798,39 | 37704,05 | 18551,93 | 31259,24 | 38082,05 | 14895,88 |
| **SPECC1_N303Tfs*63_** | MHYGHQAPQ | 38613,98 | 34602,78 | 23323,87 | 36245,22 | 33265,04 | 26152,37 | 23541,91 | 16999,48 | 2759,98 | 31174,12 | 30568,88 | 21617,68 |
| **SPECC1_N303Tfs*63_** | HYGHQAPQV | 37216,47 | 29088,46 | 35022 | 2640,45 | 38021,95 | 32489,24 | 23749,66 | 29081,85 | 27067,26 | 40414,43 | 35363,55 | 36144,96 |
| **SPECC1_N303Tfs*63_** | YGHQAPQVA | 35653,62 | 31562,04 | 40466,07 | 37679,16 | 41087,7 | 17709,54 | 10061,39 | 32727,38 | 11042,54 | 33804,92 | 25731,08 | 16177,43 |
| **SPECC1_N303Tfs*63_** | GHQAPQVAM | 36535,41 | 31049,57 | 33882,19 | 31046,54 | 37789,4 | 18806,59 | 27816,64 | 20729,16 | 189,52 | 14123,04 | 34851,89 | 18320,15 |
| **SPECC1_N303Tfs*63_** | HQAPQVAML | 30751,01 | 1507,82 | 27522,35 | 15258,83 | 10767,75 | 9257,82 | 7887,26 | 7090 | 80,74 | 1528,14 | 17939,61 | 181,9 |
| **SPECC1_N303Tfs*63_** | QAPQVAMLP | 35871,48 | 32413,4 | 39575,39 | 34913,42 | 38014,55 | 37287,41 | 35907,98 | 41375,89 | 35320,73 | 37410,25 | 20646,12 | 32428,83 |
| **SPECC1_N303Tfs*63_** | APQVAMLPK | 33620,73 | 38675,44 | 9170,98 | 40791,81 | 37841,37 | 4356,48 | 25745,84 | 26668,43 | 35020,49 | 37495,35 | 32224,92 | 33011,19 |
| **SPECC1_N303Tfs*63_** | PQVAMLPKL | 40100,39 | 10775,21 | 39406,61 | 17826,23 | 32192,51 | 37540,01 | 30227,81 | 18667,92 | 15355,38 | 14893,46 | 30400,34 | 11677,59 |
| **SPECC1_N303Tfs*63_** | QVAMLPKLL | 31284,61 | 14561,55 | 27444,74 | 25843,52 | 16315,25 | 13835,97 | 11980,13 | 25383,77 | 20855,37 | 25487,79 | 11416,48 | 11840,7 |
| **SPECC1_N303Tfs*63_** | VAMLPKLLC | 32815,32 | 19949,79 | 28483,63 | 29861,15 | 42585,87 | 17959,43 | 15742,81 | 30637,76 | 28162,48 | 34899,44 | 3716,92 | 25293,57 |
| **SPECC1_N303Tfs*63_** | AMLPKLLCR | 30041,96 | 14339,84 | 289,37 | 29471,79 | 35553,08 | 34336,49 | 17128,72 | 10473,71 | 37280,54 | 37902,82 | 33162,97 | 25659,62 |
| **SPECC1_N303Tfs*63_** | MLPKLLCRQ | 30885,38 | 13740,2 | 20107,11 | 34682,63 | 23803,95 | 35281,39 | 23415,16 | 30579,13 | 39496,27 | 42072,48 | 31837,46 | 15528,81 |
| **SPECC1_N303Tfs*63_** | LPKLLCRQM | 37780,82 | 38730,3 | 37528,22 | 37466,56 | 31340,17 | 247,39 | 338,71 | 26600,7 | 22285,28 | 37341,92 | 34007,07 | 21121,52 |
| **SPECC1_N303Tfs*63_** | PKLLCRQML | 44454,97 | 42906,37 | 43776,71 | 38221,58 | 45968,36 | 34767,17 | 21032,36 | 21033,27 | 27575,71 | 37468,59 | 41365,59 | 40144,66 |
| **SPECC1_N303Tfs*63_** | KLLCRQMLP | 33335,65 | 3154,14 | 8550,84 | 26064,79 | 43197,04 | 32324,79 | 11390,21 | 16451,03 | 36296,64 | 35697,25 | 16632,16 | 19982,4 |
| **SPECC1_N303Tfs*63_** | LLCRQMLPT | 25978,65 | 1565,2 | 17142,81 | 37634,77 | 37411,46 | 29216,2 | 12247,77 | 27617,21 | 27757,11 | 38623,59 | 28245,79 | 12158,38 |
| **SPECC1_N303Tfs*63_** | LCRQMLPTL | 38634,03 | 17094,28 | 35876,92 | 29972,82 | 39284,86 | 3123,34 | 2340,01 | 30003,96 | 25323,68 | 28213,4 | 14165,59 | 12433,23 |
| **SPECC1_N303Tfs*63_** | CRQMLPTLS | 35711,15 | 40518,64 | 36719,3 | 37839,73 | 42904,52 | 35848,59 | 22318,33 | 1167,55 | 21013,48 | 38953,87 | 36668,07 | 36226,01 |
| **SPECC1_N303Tfs*63_** | RQMLPTLST | 32065,62 | 2803,32 | 9881,22 | 18870,99 | 36495,11 | 6527,91 | 9814,2 | 1189,81 | 2250,84 | 4576,57 | 16788,73 | 483,82 |
| **SPECC1_N303Tfs*63_** | QMLPTLSTL | 33515,75 | 69,86 | 17421,61 | 5100,85 | 21890,24 | 7399,42 | 4421,92 | 12907,36 | 1868,46 | 13110,89 | 3917,56 | 259,59 |
| **SPECC1_N303Tfs*63_** | MLPTLSTLQ | 25702,14 | 14684,48 | 16558,54 | 32610,74 | 23111,6 | 33774,22 | 25523,94 | 30021,18 | 37151,7 | 38465,1 | 28152,12 | 12214,29 |
| **SPECC1_N303Tfs*63_** | LPTLSTLQQ | 39143,17 | 44116,7 | 35494,27 | 46668,98 | 41498,74 | 10914,49 | 30372,39 | 36910,09 | 35977,98 | 43796,62 | 31062 | 37356,85 |
| **SPECC1_N303Tfs*63_** | PTLSTLQQR | 33475,9 | 38464,29 | 15249,09 | 40365,48 | 33303,93 | 43616,43 | 43076,16 | 36776,93 | 46009,17 | 46783,21 | 33677,15 | 41373,22 |
| **SPECC1_N303Tfs*63_** | TLSTLQQRH | 24349,68 | 33038,35 | 9057,68 | 44244,32 | 30478,08 | 39673,16 | 35462,42 | 33349,73 | 40497,16 | 40266,04 | 29710,64 | 11912,92 |
| **SPECC1_N303Tfs*63_** | LSTLQQRHP | 38167,44 | 41244,04 | 41090,38 | 45795,61 | 45565,27 | 41466,42 | 35874,19 | 41120,17 | 44514,18 | 44381,43 | 12268,72 | 36614,95 |
| **SPECC1_N303Tfs*63_** | STLQQRHPQ | 33456,34 | 36860,99 | 18160,3 | 43064,05 | 30338,57 | 18810,25 | 19795,83 | 32102,76 | 34729,57 | 40891,24 | 21920,83 | 26195,14 |
| **SPECC1_N303Tfs*63_** | TLQQRHPQG | 39395,1 | 31513,92 | 33784,09 | 37221,29 | 43287,33 | 26705,11 | 2919,96 | 33780,43 | 38978,32 | 42748,36 | 36403,61 | 22470,01 |
| **SPECC1_N303Tfs*63_** | LQQRHPQGP | 42851,17 | 37716,7 | 41246,28 | 40836,85 | 45143,76 | 39643,11 | 24323,88 | 30032,88 | 32096,86 | 29689,11 | 37874,55 | 17981,01 |
| **SPECC1_N303Tfs*63_** | QQRHPQGPC | 42942,61 | 32792,6 | 31622,55 | 41161,11 | 39472,77 | 10287,93 | 15592,28 | 24209,13 | 23976,88 | 23929,96 | 35533,09 | 5024,33 |
| **SPECC1_N303Tfs*63_** | QRHPQGPCP | 46089,89 | 46733,13 | 44490,59 | 45692,66 | 46322,35 | 41081,93 | 34171,21 | 18846,3 | 25232,34 | 39811,61 | 43601,35 | 41207,01 |
| **SPECC1_N303Tfs*63_** | RHPQGPCPP | 43223,23 | 42517,73 | 42595,07 | 37091,44 | 45722,81 | 32785,88 | 37999,74 | 35291,7 | 14925,88 | 34514,55 | 42775,19 | 39655,12 |
| **SPECC1_N303Tfs*63_** | HPQGPCPPP | 41126,86 | 39625,52 | 41230,21 | 42970,03 | 41705,81 | 2573,51 | 23510,09 | 38135,65 | 20676,31 | 37045,33 | 38840,66 | 37221,29 |
| **SPECC1_N303Tfs*63_** | PQGPCPPPV | 40143,81 | 10183,06 | 38417,28 | 30031,56 | 43595,2 | 36584,07 | 35731,63 | 32193,55 | 28839,02 | 31739,09 | 38880,61 | 25410,42 |
| **SPECC1_N303Tfs*63_** | QGPCPPPVT | 42782,61 | 37510,78 | 43273,77 | 41350,39 | 46151,25 | 37452,38 | 38115,86 | 43684,92 | 36986,84 | 42104,8 | 40136,84 | 40284,35 |
| **SPECC1_N303Tfs*63_** | GPCPPPVTP | 44246,23 | 42301,61 | 42833,11 | 46610,41 | 46741,75 | 18948,34 | 40424,93 | 43156,88 | 35337,18 | 39307,82 | 39090,67 | 42512,66 |
| **SPECC1_N303Tfs*63_** | PCPPPVTPL | 43159,19 | 30763,98 | 42296,1 | 33297,81 | 42368,94 | 30323,8 | 36887,34 | 44387,21 | 34707,4 | 38770,14 | 40378,61 | 37827,85 |
| **SPECC1_N303Tfs*63_** | CPPPVTPLR | 31871,23 | 38590,58 | 20548,07 | 33668,42 | 21141,88 | 20052,36 | 29159,36 | 30551,36 | 35577,71 | 41337,4 | 36225,24 | 37725,68 |
| **SPECC1_N303Tfs*63_** | PPPVTPLRV | 39642,67 | 38364,1 | 43013,28 | 37087,04 | 44029,9 | 23022,25 | 33315,1 | 42847,47 | 36540,95 | 44848,73 | 41037,49 | 44368,01 |
| **SPECC1_N303Tfs*63_** | PPVTPLRVQ | 46858,7 | 47043,1 | 42004,7 | 48524,22 | 44989,17 | 26461,2 | 41065,93 | 43840,71 | 44435,25 | 46718,48 | 44294,14 | 43255,96 |
| **SPECC1_N303Tfs*63_** | PVTPLRVQS | 41698,14 | 40741,52 | 37318,88 | 45386,66 | 43831,21 | 38435,57 | 40946,13 | 42650,88 | 45222,46 | 46187,72 | 37996,46 | 40483,14 |
| **SPECC1_N303Tfs*63_** | VTPLRVQSV | 25074,21 | 5615,37 | 32292,99 | 11579,7 | 13997,39 | 18294,01 | 3402,83 | 34334,28 | 29074,93 | 38365,36 | 23298,65 | 16914,54 |
| **SPECC1_N303Tfs*63_** | TPLRVQSVL | 35867,59 | 31516,66 | 35150,32 | 33352,25 | 31727,41 | 26,51 | 1311,98 | 28466,07 | 1412,66 | 25361,26 | 22527,95 | 27254,76 |
| **SPECC1_N303Tfs*63_** | PLRVQSVLL | 34061,2 | 13528,95 | 29523,5 | 25263,47 | 39265,31 | 18466,62 | 5488,1 | 32754,66 | 35830,35 | 34465,66 | 35771,47 | 15775,39 |
| **SPECC1_N303Tfs*63_** | LRVQSVLLL | 32094,44 | 21288,09 | 32058,34 | 18220,71 | 29443,75 | 23813,99 | 10944,53 | 46,92 | 223,95 | 15991,06 | 18453,44 | 16544,21 |
| **SPECC1_N303Tfs*63_** | RVQSVLLLG | 25612,19 | 11853,9 | 10180,09 | 18311,83 | 35033 | 18431,3 | 24331,5 | 9304,91 | 35485,82 | 32527,92 | 1614,19 | 6455,21 |
| **SPECC1_N303Tfs*63_** | VQSVLLLGV | 24721,33 | 139,33 | 21159,27 | 22040,68 | 32165,7 | 33299,96 | 20746 | 6867,57 | 9656,62 | 9701,44 | 12320,21 | 2033,56 |
| **SPECC1_N303Tfs*63_** | QSVLLLGVP | 32470,96 | 30460,27 | 37420,36 | 42381,79 | 34371,05 | 35733,96 | 33015,84 | 36932,47 | 35077 | 32686,69 | 9522,57 | 19713,96 |
| **SPECC1_N303Tfs*63_** | SVLLLGVPQ | 37688,13 | 25144,04 | 18521,86 | 43666,5 | 34962,94 | 24729,35 | 26936,09 | 31109,1 | 35111,92 | 36634,76 | 27730,7 | 17160,07 |
| **SPECC1_N303Tfs*63_** | VLLLGVPQT | 40381,66 | 196,35 | 24248,2 | 38553,44 | 43067,3 | 37182,26 | 26872,93 | 34299 | 36051,24 | 38581,4 | 32324,45 | 24620,96 |
| **SPECC1_N303Tfs*63_** | LLLGVPQTA | 30989,83 | 72,97 | 22955,34 | 35114,21 | 37978,38 | 26596,1 | 14334,88 | 26576,83 | 18500,62 | 31316,45 | 22885,9 | 8993,32 |
|  |  |  |  |  |  |  |  |  |  |  |  |  |  |
| **PROTEIN** | **PEPTIDE** | **A*01:01** | **A*02:01** | **A*03:01** | **A*24:02** | **A*26:02** | **B*07:02** | **B*08:02** | **B*27:05** | **B*39:01** | **B*40:01** | **B*58:01** | **B*15:01** |
| **ARID1A_1850wt_** | LHWRIGGGD | 42981,2 | 42388,64 | 41864,5 | 40327,52 | 45602,28 | 40250,8 | 38061,45 | 24911,97 | 24632,14 | 39521,06 | 35064,08 | 39858,6 |
| **ARID1A_1850wt_** | HWRIGGGDT | 44379,52 | 45985,26 | 40398,69 | 41025,08 | 44707,25 | 27767,03 | 26787,87 | 38796,97 | 44516,59 | 45846,68 | 45786,7 | 35909,93 |
| **ARID1A_1850wt_** | WRIGGGDTT | 42057,45 | 38113,79 | 44153,45 | 41690,02 | 36897,71 | 31857,44 | 30081,01 | 4367,24 | 1333,07 | 27622,89 | 39344,85 | 32946,6 |
| **ARID1A_1850wt_** | RIGGGDTTE | 42459,81 | 35281 | 34455,97 | 44826,4 | 45098,33 | 33253,88 | 39292,95 | 40150,75 | 44137,22 | 44190,25 | 33913,38 | 27415,35 |
| **ARID1A_1850wt_** | IGGGDTTEH | 40000,28 | 45144,72 | 37519,71 | 47318,26 | 44768,74 | 41496,95 | 43514,16 | 44362,23 | 45015 | 46392,57 | 34265,24 | 29877,31 |
| **ARID1A_1850wt_** | GGGDTTEHI | 40681,63 | 33585,47 | 44282,17 | 37920,06 | 46423,7 | 40001,16 | 41388,89 | 41368,29 | 39817,64 | 41253,41 | 26360,32 | 36570,6 |
| **ARID1A_1850wt_** | GGDTTEHIQ | 38846,55 | 45086,64 | 42548,57 | 47574,43 | 47967,75 | 45220,01 | 45521,92 | 44698,55 | 45148,65 | 44713,05 | 37464,12 | 44288,85 |
| **ARID1A_1850wt_** | GDTTEHIQT | 42438,67 | 45071,53 | 45069,58 | 47058,88 | 46768,04 | 45364,07 | 43166,21 | 43595,2 | 44977,5 | 38183,96 | 43456,75 | 46098,86 |
| **ARID1A_1850wt_** | DTTEHIQTH | 20349,61 | 42452 | 32283,55 | 45980,78 | 750 | 39443,3 | 32798,65 | 41628,26 | 36568,24 | 41648,07 | 35350,17 | 27412,38 |
|  |  |  |  |  |  |  |  |  |  |  |  |  |  |
| **ARID1A_D1850Tfs*33_** | LHWRIGGGT | 39345,26 | 37011,27 | 38834,37 | 39757,8 | 43330,93 | 34602,4 | 33659,67 | 17182,18 | 10077,73 | 35856,33 | 38185,22 | 37577,39 |
| **ARID1A_D1850Tfs*33_** | HWRIGGGTP | 44117,64 | 43464,74 | 38374,91 | 33719,81 | 42264,09 | 23115,86 | 20677,65 | 33593,09 | 39711,81 | 41874,9 | 42262,26 | 26099,8 |
| **ARID1A_D1850Tfs*33_** | WRIGGGTPL | 33262,87 | 14204,88 | 33402,81 | 23652,21 | 16911,61 | 4634,47 | 3507,53 | 51,15 | 6,08 | 3835,77 | 29509,75 | 5617,81 |
| **ARID1A_D1850Tfs*33_** | RIGGGTPLS | 35287,51 | 18251,3 | 16163,26 | 35122,95 | 40861,16 | 25561,81 | 37542,43 | 31370,03 | 42275,51 | 40931,53 | 25013,79 | 23067,13 |
| **ARID1A_D1850Tfs*33_** | IGGGTPLSI | 34556,39 | 21163,61 | 33467,57 | 19085,99 | 44558,99 | 24002,82 | 24492,62 | 32985,12 | 24646,81 | 37881,1 | 8403,72 | 22135,81 |
| **ARID1A_D1850Tfs*33_** | GGGTPLSIS | 43559,84 | 40932,4 | 40659,18 | 45535,72 | 46436,75 | 41001,54 | 42268,67 | 41196,78 | 45426,46 | 44537,79 | 35368,91 | 37466,97 |
| **ARID1A_D1850Tfs*33_** | GGTPLSISR | 39617,8 | 38840,23 | 17880,32 | 41149,99 | 42605,68 | 38958,52 | 40141,63 | 27069,03 | 42324,04 | 41233,32 | 29536,9 | 33584 |
| **ARID1A_D1850Tfs*33_** | GTPLSISRP | 38610,22 | 35790,84 | 39460,8 | 41563,9 | 37712,61 | 41423,39 | 41917,51 | 42486,46 | 44713,54 | 42782,61 | 32315,01 | 35342,14 |
| **ARID1A_D1850Tfs*33_** | TPLSISRPT | 40772,4 | 34918,71 | 36923,66 | 44276,89 | 39109,28 | 327,91 | 11106,17 | 31938,55 | 13172,46 | 37752,62 | 37314,04 | 36731,61 |
| **ARID1A_D1850Tfs*33_** | PLSISRPTS | 41455,21 | 33781,54 | 35718,5 | 43743,11 | 46309,32 | 38922,28 | 37439,82 | 41994,7 | 45305,71 | 45527,82 | 39578,4 | 35233,7 |
| **ARID1A_D1850Tfs*33_** | LSISRPTSR | 28319,22 | 33450,2 | 3483,33 | 39336,33 | 22274,43 | 27735,49 | 16291,08 | 20931,11 | 35660,96 | 39787,48 | 20911,87 | 19988,02 |
| **ARID1A_D1850Tfs*33_** | SISRPTSRA | 27631,26 | 19931,01 | 14045,48 | 40690,86 | 23437,2 | 9829,08 | 19814,9 | 32741,21 | 34297,88 | 39050,5 | 24487,85 | 17632,1 |
| **ARID1A_D1850Tfs*33_** | ISRPTSRAR | 36985,65 | 38997,73 | 5153,49 | 41003,34 | 34120,96 | 14244,12 | 24223,02 | 26631,24 | 40590,61 | 40846,14 | 27154,98 | 17424,25 |
| **ARID1A_D1850Tfs*33_** | SRPTSRARQ | 45562,33 | 46432,74 | 40938,61 | 44492,02 | 43384,87 | 40176,83 | 35234,09 | 18219,93 | 38910,91 | 45319,45 | 45059,81 | 42036,97 |
| **ARID1A_D1850Tfs*33_** | RPTSRARQS | 43778,61 | 44506,46 | 36802,42 | 45441,7 | 45042,27 | 562,28 | 11352,19 | 31250,09 | 38192,23 | 43675,48 | 38695,52 | 41506,82 |
| **ARID1A_D1850Tfs*33_** | PTSRARQSC | 37568,04 | 43740,26 | 40672,37 | 41306,12 | 44717,89 | 34049,41 | 35744,4 | 43131,2 | 43551,36 | 46528,82 | 27688,72 | 40134,67 |
| **ARID1A_D1850Tfs*33_** | TSRARQSCC | 35164,02 | 41806,55 | 33805,66 | 42478,65 | 41007,33 | 13100,54 | 3165,29 | 36898,5 | 40710,23 | 43802,78 | 23464,11 | 24754,51 |
| **ARID1A_D1850Tfs*33_** | SRARQSCCL | 33169,43 | 32901,71 | 34637,61 | 27637,23 | 33497,28 | 12971,06 | 1307,51 | 545,66 | 349,41 | 23620,76 | 33123,17 | 20131,71 |
| **ARID1A_D1850Tfs*33_** | RARQSCCLP | 34377,39 | 30798,29 | 22185,91 | 34123,91 | 42351,51 | 9626,47 | 15630,29 | 22527,46 | 36100,8 | 33020,48 | 8008,86 | 11421,18 |
| **ARID1A_D1850Tfs*33_** | ARQSCCLPG | 36265,23 | 37155,7 | 32201,92 | 32677,86 | 42680,89 | 29165,68 | 17887,86 | 859,54 | 18612,66 | 30912,12 | 31741,49 | 27439,09 |
| **ARID1A_D1850Tfs*33_** | RQSCCLPGL | 26643,62 | 120,13 | 10924,77 | 8974,56 | 27182,31 | 10789,21 | 13164,06 | 499,05 | 2939,71 | 928,25 | 8446,2 | 477,63 |
| **ARID1A_D1850Tfs*33_** | QSCCLPGLT | 18787,47 | 29894,77 | 29683,33 | 39879,3 | 37963,98 | 38689,66 | 35730,87 | 32598,76 | 37758,34 | 38866,72 | 10853,02 | 29846,92 |
| **ARID1A_D1850Tfs*33_** | SCCLPGLTH | 34039,09 | 39947,09 | 20846,13 | 44965,82 | 42560,53 | 36852,22 | 39938,88 | 38984,65 | 40222,48 | 39955,73 | 27088,95 | 23989,59 |
| **ARID1A_D1850Tfs*33_** | CCLPGLTHP | 42841,89 | 30435,88 | 41768,12 | 44412,18 | 46193,23 | 41153,1 | 39361,02 | 42710 | 43102,28 | 42331,36 | 25828,71 | 38033,48 |
| **ARID1A_D1850Tfs*33_** | CLPGLTHPA | 21540,4 | 94,71 | 24117,89 | 19089,29 | 15016,1 | 18130,65 | 9974,46 | 25225,25 | 12815,66 | 31471,33 | 32775,22 | 7590,47 |
| **ARID1A_D1850Tfs*33_** | LPGLTHPAH | 38337,96 | 44522,36 | 32287,03 | 45757,47 | 38320,55 | 2291,06 | 15752,7 | 34444,41 | 27397,27 | 40071,33 | 37103,48 | 26929,1 |
| **ARID1A_D1850Tfs*33_** | PGLTHPAHQ | 45742,6 | 46596,8 | 42258,61 | 47312,64 | 47932,99 | 45949,48 | 42740,5 | 44627,98 | 47883,74 | 48340,29 | 43141,94 | 44884,14 |
| **ARID1A_D1850Tfs*33_** | GLTHPAHQP | 42493,36 | 20611,97 | 34953,86 | 44400,64 | 45551,49 | 41145,98 | 40129,03 | 40419,7 | 42898,01 | 37445,47 | 36659,74 | 33419,45 |
| **ARID1A_D1850Tfs*33_** | LTHPAHQPL | 18669,53 | 10666,99 | 22776,47 | 11039,07 | 10352,93 | 468,08 | 2731,19 | 22383,62 | 1941,81 | 12791,42 | 1814,33 | 522,55 |
| **ARID1A_D1850Tfs*33_** | THPAHQPLG | 40491,91 | 43628,24 | 41898,94 | 31000,57 | 43441,23 | 40027,55 | 35848,98 | 35571,56 | 19076,49 | 39579,24 | 39752,63 | 39416,85 |
| **ARID1A_D1850Tfs*33_** | HPAHQPLGS | 36405,57 | 42783,52 | 36005,23 | 45707,48 | 36495,11 | 3961,72 | 23249,29 | 35770,32 | 24995,94 | 43099,95 | 34365,49 | 39849,96 |
| **ARID1A_D1850Tfs*33_** | PAHQPLGSM | 36200,93 | 37968,93 | 37337,05 | 39806,01 | 17334,74 | 8252,43 | 22486,31 | 36443,82 | 29962,11 | 36306,07 | 26438,88 | 11814,6 |
|  |  |  |  |  |  |  |  |  |  |  |  |  |  |
| **PROTEIN** | **PEPTIDE** | **A*01:01** | **A*02:01** | **A*03:01** | **A*24:02** | **A*26:02** | **B*07:02** | **B*08:02** | **B*27:05** | **B*39:01** | **B*40:01** | **B*58:01** | **B*15:01** |
| **CTCF_204wt_** | DYQPPAKKT | 45746,08 | 46182,72 | 44377,6 | 35789,29 | 44252,94 | 42728,95 | 37298,69 | 44934,68 | 40896,09 | 46970,38 | 46231,71 | 45835,77 |
| **CTCF_204wt_** | YQPPAKKTK | 41260,11 | 36753,48 | 19124,64 | 34218,56 | 38957,66 | 36734,79 | 35823 | 25341,79 | 35560,39 | 35704,2 | 38812,94 | 19098,39 |
| **CTCF_204wt_** | QPPAKKTKK | 44309,48 | 47067,53 | 33355,5 | 46280,27 | 42132,13 | 29164,74 | 31702,7 | 42699,37 | 45464,82 | 46566,07 | 45678,81 | 45923,62 |
| **CTCF_204wt_** | PPAKKTKKT | 46849,07 | 47723,43 | 45807,99 | 48117,46 | 47585,23 | 24957,03 | 26899,11 | 45730,24 | 45832,78 | 48175,8 | 46859,73 | 47292,14 |
| **CTCF_204wt_** | PAKKTKKTK | 45945,98 | 47510,65 | 29128,78 | 46874,42 | 46441,28 | 43062,66 | 38233,59 | 42976,07 | 48007,73 | 48236,83 | 42566,98 | 41195,88 |
| **CTCF_204wt_** | AKKTKKTKK | 47136,85 | 47933,52 | 31617,76 | 47473,13 | 47223,63 | 45018,89 | 34642,86 | 24396,36 | 47205,24 | 46593,8 | 46883,04 | 42643,04 |
| **CTCF_204wt_** | KKTKKTKKS | 48189,88 | 48148,19 | 44992,1 | 48248,31 | 47928,32 | 44432,87 | 36395,34 | 31775,16 | 46445,29 | 46934,31 | 45423,03 | 45897,29 |
| **CTCF_204wt_** | KTKKTKKSK | 42429,94 | 45286,61 | 1252,01 | 44106,2 | 42929,59 | 31649,59 | 32287,74 | 27264,5 | 47481,86 | 46070,42 | 35244 | 30905,45 |
| **CTCF_204wt_** | TKKTKKSKL | 45455,97 | 46744,76 | 44459,79 | 44813,8 | 44231,39 | 30275,61 | 992,03 | 29490,62 | 31813,01 | 39190,63 | 45462,86 | 36091,44 |
|  |  |  |  |  |  |  |  |  |  |  |  |  |  |
| **CTCF_T204Nfs*26_** | DYQPPAKKN | 46539,38 | 48121,12 | 44627,03 | 38681,3 | 46126,79 | 46287,77 | 42909,63 | 45413,19 | 46636,66 | 48275,98 | 45764,41 | 46036,06 |
| **CTCF_T204Nfs*26_** | YQPPAKKNK | 42259,97 | 38423,52 | 17083,56 | 35995,11 | 39762,52 | 39741,45 | 38771,39 | 28056,63 | 40027,14 | 39360,18 | 40609,49 | 23766,89 |
| **CTCF_T204Nfs*26_** | QPPAKKNKE | 47591,92 | 47921,06 | 46109,33 | 47778,19 | 46773,11 | 32614,97 | 28909,31 | 45851,14 | 45071,99 | 47407,41 | 47066,02 | 47246,11 |
| **CTCF_T204Nfs*26_** | PPAKKNKEN | 47365,88 | 48590,96 | 46916,04 | 48627,76 | 48751,57 | 38633,61 | 39746,19 | 45845,67 | 47897,24 | 48723,09 | 46054,98 | 47174,11 |
| **CTCF_T204Nfs*26_** | PAKKNKENQ | 46694,21 | 48327,22 | 45583,52 | 48336,11 | 47857,35 | 45971,83 | 41197,66 | 46713,42 | 48608,82 | 48663,55 | 43866,33 | 45436,8 |
| **CTCF_T204Nfs*26_** | AKKNKENQK | 46897,77 | 47194,52 | 33272,59 | 47590,91 | 46726,05 | 45243,49 | 35896,71 | 25732,19 | 46881,01 | 45599,31 | 46505,14 | 40957,22 |
| **CTCF_T204Nfs*26_** | KKNKENQKE | 47594,5 | 47563,11 | 45572,68 | 48215,96 | 47912,25 | 46685,11 | 44512,75 | 38797,4 | 45367,03 | 44607,23 | 43283,12 | 44199,83 |
| **CTCF_T204Nfs*26_** | KNKENQKEQ | 46811,07 | 47503,45 | 41460,59 | 48095,09 | 46564,05 | 44786,18 | 37024,48 | 43638,15 | 47515,77 | 46611,95 | 42232,1 | 40192,04 |
| **CTCF_T204Nfs*26_** | NKENQKEQT | 46837,4 | 48583,06 | 48068,02 | 48403,08 | 48582,53 | 45882,88 | 39822,8 | 43282,2 | 37020,46 | 46132,29 | 47347,44 | 47574,43 |
| **CTCF_T204Nfs*26_** | KENQKEQTA | 42603,37 | 43100,39 | 42650,88 | 44937,12 | 44955,61 | 38318,9 | 24998,36 | 36431,6 | 32722,44 | 6613,79 | 40059,19 | 30159,87 |
| **CTCF_T204Nfs*26_** | ENQKEQTAL | 35167,44 | 41658,9 | 42811,32 | 40037,52 | 29698,42 | 23521,3 | 8678,63 | 39457,82 | 7316,07 | 29945,27 | 39069,09 | 30255,96 |
| **CTCF_T204Nfs*26_** | NQKEQTALY | 9354,88 | 33891,36 | 16460,46 | 29705,16 | 2770,99 | 34042,78 | 19198,24 | 17214,56 | 23179,21 | 21504,08 | 28402,99 | 58,38 |
| **CTCF_T204Nfs*26_** | QKEQTALYR | 39727,28 | 44024,67 | 32580,41 | 45620,05 | 44334,41 | 43740,73 | 41136,64 | 24248,73 | 39283,6 | 41070,38 | 42572,96 | 41033,5 |
| **CTCF_T204Nfs*26_** | KEQTALYRG | 41725,66 | 41664,76 | 41587,28 | 35080,41 | 43692,02 | 42238,94 | 35038,67 | 25984,84 | 38996,04 | 13216,29 | 31089,58 | 33753,77 |
| **CTCF_T204Nfs*26_** | EQTALYRGG | 41984,25 | 40276,95 | 42912,87 | 41973,8 | 36399,29 | 41069,47 | 32044,47 | 26116,18 | 32613,92 | 35102,05 | 33833,1 | 22292,99 |
| **CTCF_T204Nfs*26_** | QTALYRGGQ | 32152,47 | 39945,37 | 25364,01 | 45989,74 | 21125,41 | 31752,83 | 33177,69 | 37084,22 | 42847 | 43797,56 | 29712,88 | 27419,81 |
| **CTCF_T204Nfs*26_** | TALYRGGQR | 35224,18 | 38229,45 | 13654,24 | 42792,79 | 26095 | 30906,44 | 24317,55 | 26295,66 | 40315,3 | 42060,62 | 34511,92 | 32671,83 |
| **CTCF_T204Nfs*26_** | ALYRGGQRC | 37054,55 | 3617,73 | 16894,78 | 34816,84 | 38195,54 | 25037,08 | 24154,97 | 24310,19 | 31984,89 | 32374,14 | 28050,57 | 10691,95 |
| **CTCF_T204Nfs*26_** | LYRGGQRCR | 43351,56 | 44157,75 | 25546,88 | 32909,54 | 40149,45 | 37387,2 | 31324,91 | 28543,17 | 44417 | 45880,9 | 43145,19 | 38468,43 |
| **CTCF_T204Nfs*26_** | YRGGQRCRC | 42930,06 | 42834,95 | 44630,4 | 41969,25 | 45113,49 | 34342,07 | 20150,66 | 5305,17 | 20785,76 | 43524,04 | 43585,78 | 42419,85 |
| **CTCF_T204Nfs*26_** | RGGQRCRCV | 40785,18 | 38272,89 | 39269,99 | 34854,54 | 46089,89 | 26948,34 | 6187,02 | 30002,35 | 38937,02 | 43549,95 | 36036,02 | 36143,4 |
| **CTCF_T204Nfs*26_** | GGQRCRCVC | 41811,54 | 41297,2 | 39524,91 | 42149,01 | 47525,55 | 29422,09 | 21525,03 | 32408,13 | 38443,05 | 42509,45 | 33288,79 | 36537,79 |
| **CTCF_T204Nfs*26_** | GQRCRCVCL | 35089,14 | 11716,96 | 26850,55 | 22242,16 | 36442,25 | 10134,04 | 201,2 | 6251,15 | 12702,45 | 10411,9 | 34551,89 | 757,08 |
| **CTCF_T204Nfs*26_** | QRCRCVCLR | 36903,71 | 34797,63 | 22424,11 | 34456,32 | 38704,33 | 40297,42 | 31570,92 | 94,17 | 32247,25 | 40397,39 | 33857,29 | 38268,35 |
| **CTCF_T204Nfs*26_** | RCRCVCLRF | 30077,09 | 30643,73 | 21997,09 | 12056,2 | 35304,3 | 17799,63 | 11900,81 | 18415,74 | 39973,9 | 31149,51 | 5391,51 | 3637,47 |
|  |  |  |  |  |  |  |  |  |  |  |  |  |  |
|  |  |  |  |  |  |  |  |  |  |  |  |  |  |
| **PROTEIN** | **PEPTIDE** | **A*01:01** | **A*02:01** | **A*03:01** | **A*24:02** | **A*26:02** | **B*07:02** | **B*08:02** | **B*27:05** | **B*39:01** | **B*40:01** | **B*58:01** | **B*15:01** |
| **CSMD3_3640wt_** | AIAILVPFF | 26071 | 9722,46 | 12236,24 | 6864,6 | 10388,84 | 25652,96 | 23476,55 | 563,7 | 27139,12 | 37799,22 | 28358,48 | 3810,5 |
| **CSMD3_3640wt_** | IAILVPFFA | 20431,68 | 755,67 | 18533,88 | 29363,58 | 34784,84 | 29549,38 | 15921,65 | 18630,19 | 27082,51 | 19716,1 | 28299,63 | 1642 |
| **CSMD3_3640wt_** | AILVPFFAL | 35013,29 | 307,4 | 13990,73 | 16005,25 | 30258,57 | 6705,01 | 3478,51 | 6712,27 | 20581 | 10096,61 | 18517,04 | 16781,47 |
| **CSMD3_3640wt_** | ILVPFFALI | 20744,42 | 18,26 | 7822,42 | 1703,44 | 15939,75 | 28494,1 | 9639,71 | 2415,8 | 19928,43 | 21021,89 | 23055,16 | 3791,21 |
| **CSMD3_3640wt_** | LVPFFALIF | 17868,91 | 18242,81 | 20249,89 | 1097,82 | 7609,79 | 30243,53 | 21970,21 | 962,79 | 31622,21 | 31842,63 | 30445,45 | 8237,98 |
| **CSMD3_3640wt_** | VPFFALIFA | 33080,54 | 10892,9 | 23302,69 | 38231,52 | 35601,97 | 5204,88 | 16350,24 | 31985,59 | 33128,9 | 19134,17 | 28939,04 | 27050,88 |
| **CSMD3_3640wt_** | PFFALIFAG | 40923,56 | 28105,86 | 35804,39 | 16404,82 | 42966,76 | 43749,73 | 31195,71 | 35535,77 | 34013,7 | 39697,62 | 43278,46 | 32788,35 |
| **CSMD3_3640wt_** | FFALIFAGF | 24986,74 | 16618,66 | 29210,51 | 879,4 | 8772,85 | 30466,21 | 8103,08 | 3965,02 | 24313,87 | 24557,91 | 28024,17 | 21555,09 |
| **CSMD3_3640wt_** | FALIFAGFG | 31270,05 | 22657,01 | 33016,2 | 41216,39 | 38777,69 | 36942,86 | 23937,99 | 22687,19 | 33142,89 | 36978,04 | 38874,29 | 8024,56 |
|  |  |  |  |  |  |  |  |  |  |  |  |  |  |
| **CSMD3_F3640Lfs*61_** | AIAILVPFL | 30402,65 | 137,5 | 14023,92 | 24418,81 | 20147,83 | 17502,1 | 15397,81 | 6679,38 | 26336,96 | 23993,22 | 24432,81 | 12171,28 |
| **CSMD3_F3640Lfs*61_** | IAILVPFLH | 21396,62 | 26623,17 | 8706,37 | 33788,84 | 27717,2 | 36366,22 | 29228,55 | 11398,72 | 25849,95 | 32214,46 | 32486,44 | 1600,57 |
| **CSMD3_F3640Lfs*61_** | AILVPFLHL | 35950,73 | 1318,47 | 12570,52 | 17822,38 | 30881,72 | 15915,11 | 10508,1 | 6467,52 | 24130,15 | 27563,77 | 27484,55 | 13959,12 |
| **CSMD3_F3640Lfs*61_** | ILVPFLHLY | 1597,23 | 3630,12 | 417,82 | 8255,74 | 2194,64 | 35298,96 | 28287,68 | 109,32 | 16829,47 | 31260,59 | 27962,69 | 924,5 |
| **CSMD3_F3640Lfs*61_** | LVPFLHLYL | 15813,67 | 975,69 | 17907,42 | 2343,99 | 8090,9 | 13107,77 | 5614,34 | 4635,57 | 26391,43 | 15106,71 | 22855,72 | 9241,1 |
| **CSMD3_F3640Lfs*61_** | VPFLHLYLQ | 37875,36 | 36653 | 28918,06 | 40203,35 | 36270,73 | 12798,61 | 13730,98 | 35673,31 | 32912,39 | 32107,63 | 38712,27 | 27842,54 |
| **CSMD3_F3640Lfs*61_** | PFLHLYLQD | 44434,79 | 43366,1 | 42942,13 | 24820,75 | 47340,29 | 47003,44 | 42813,63 | 46096,88 | 43469,45 | 47717,74 | 48085,73 | 41276,64 |
| **CSMD3_F3640Lfs*61_** | FLHLYLQDL | 25302,61 | 34,8 | 31449,88 | 21888,35 | 23408,06 | 16595,48 | 214,57 | 3463,71 | 21527,59 | 5356,62 | 25624,11 | 31899,17 |
| **CSMD3_F3640Lfs*61_** | LHLYLQDLD | 39742,32 | 41093,05 | 42013,31 | 33894,66 | 46412,14 | 43771,49 | 40031,04 | 40361,55 | 31333,05 | 20086,23 | 37326,15 | 27774,22 |
| **CSMD3_F3640Lfs*61_** | HLYLQDLDF | 15842,43 | 6422,47 | 13308,12 | 8235,67 | 15589,42 | 22662,65 | 8261,55 | 143,34 | 16488,27 | 15738,9 | 25673,51 | 8579,01 |
| **CSMD3_F3640Lfs*61_** | LYLQDLDFI | 32640,03 | 12473,65 | 37154,09 | 130,45 | 34802,55 | 35843,15 | 28230,51 | 29563,78 | 27394,61 | 24365,75 | 35017,84 | 15795,53 |
| **CSMD3_F3640Lfs*61_** | YLQDLDFIF | 4977,9 | 61,21 | 24088,16 | 284,22 | 12343,42 | 18992,67 | 5753,82 | 105,48 | 8020,48 | 3955,42 | 9411,74 | 2225,99 |
| **CSMD3_F3640Lfs*61_** | LQDLDFIFI | 10666,41 | 710,33 | 32527,23 | 14863,19 | 39340,16 | 34774,3 | 25802,17 | 14000,41 | 23808,58 | 5481,63 | 5026,56 | 19516,6 |
| **CSMD3_F3640Lfs*61_** | QDLDFIFIN | 36870,57 | 38577,22 | 40172,92 | 39496,27 | 42842,83 | 43964,69 | 40866,04 | 40561,2 | 34692 | 42217,02 | 34317,57 | 32393,76 |
| **CSMD3_F3640Lfs*61_** | DLDFIFINK | 12498,23 | 26242,23 | 3073,43 | 37420,36 | 27888,36 | 42874,84 | 33513,59 | 38451,79 | 34631,25 | 40710,68 | 41590,9 | 38781,86 |
| **CSMD3_F3640Lfs*61_** | LDFIFINKG | 38629,02 | 37912,26 | 40522,58 | 39442,03 | 42291,08 | 43337,95 | 34225,22 | 35392,64 | 35271,08 | 41472,7 | 33116,36 | 30177,5 |
| **CSMD3_F3640Lfs*61_** | DFIFINKGL | 39466,79 | 23777,94 | 37348,38 | 16904,47 | 14304,82 | 33161,9 | 12858,99 | 30424,37 | 30681,56 | 19517,64 | 32068,04 | 38647,41 |
| **CSMD3_F3640Lfs*61_** | FIFINKGLH | 26106,3 | 27146,75 | 6856,28 | 42707,68 | 12343,42 | 30941,24 | 23628,94 | 7754,66 | 27270,39 | 36372,51 | 37229,36 | 29176,4 |
| **CSMD3_F3640Lfs*61_** | IFINKGLHL | 33127,1 | 12659,93 | 24484,68 | 501,97 | 29238,97 | 19095,08 | 3091,94 | 8052,83 | 20037,61 | 18865,69 | 26216,98 | 22003,27 |
| **CSMD3_F3640Lfs*61_** | FINKGLHLK | 15986,38 | 16224,42 | 101,86 | 31253,48 | 15400,48 | 29888,62 | 25187,34 | 20554,07 | 18052,35 | 34607,65 | 39319,31 | 26091,32 |
| **CSMD3_F3640Lfs*61_** | INKGLHLKH | 38440,55 | 45334,65 | 29120,59 | 43240,99 | 37913,5 | 41222,64 | 28772,94 | 25647,96 | 30144,2 | 41797,95 | 41758,64 | 35888,95 |
| **CSMD3_F3640Lfs*61_** | NKGLHLKHS | 46709,39 | 46674 | 46489,55 | 47172,07 | 46330,38 | 43873,43 | 25190,61 | 44191,22 | 35817,94 | 38918,91 | 46215,22 | 45632,38 |
| **CSMD3_F3640Lfs*61_** | KGLHLKHSI | 40532,23 | 19990,41 | 32188,32 | 13701,15 | 44907,48 | 14603,2 | 9297,37 | 25880,73 | 18983,22 | 25844,07 | 31865,72 | 3250,28 |
| **CSMD3_F3640Lfs*61_** | GLHLKHSIQ | 41982,44 | 35550 | 19181,63 | 45131,53 | 42983,5 | 33462,5 | 12015,83 | 16938,71 | 32194,59 | 43231,17 | 41753,66 | 41124,63 |
| **CSMD3_F3640Lfs*61_** | LHLKHSIQD | 44424,21 | 42889,66 | 42892,92 | 38053,64 | 46886,61 | 41581,44 | 34447,01 | 41734,68 | 33998,98 | 21899,71 | 41508,18 | 36494,71 |
| **CSMD3_F3640Lfs*61_** | HLKHSIQDV | 32766 | 3607,41 | 26644,78 | 36466,29 | 23590,12 | 20731,87 | 2148,31 | 7197,43 | 32486,08 | 29876,98 | 37545,68 | 35651,69 |
| **CSMD3_F3640Lfs*61_** | LKHSIQDVQ | 45126,18 | 45691,66 | 42541,18 | 47278,84 | 46151,75 | 42113 | 37652,68 | 32048,28 | 29630,7 | 29076,51 | 36919,28 | 37432,12 |
| **CSMD3_F3640Lfs*61_** | KHSIQDVQF | 37318,09 | 37055,75 | 37197,54 | 11682,14 | 40320,53 | 34127,61 | 34889,25 | 20366,79 | 24857,31 | 6860,96 | 25516,21 | 12300,89 |
| **CSMD3_F3640Lfs*61_** | HSIQDVQFM | 14738,29 | 18837,13 | 29057,01 | 25671,01 | 759,99 | 21303,75 | 21904,46 | 1322,25 | 23356,45 | 11210,72 | 23831,26 | 198,18 |
| **CSMD3_F3640Lfs*61_** | SIQDVQFMK | 23370,1 | 21553,47 | 89,24 | 31466,24 | 28839,33 | 35425,98 | 36299,4 | 27598,69 | 21073,13 | 39249,61 | 39395,53 | 25317,66 |
| **CSMD3_F3640Lfs*61_** | IQDVQFMKI | 11891,67 | 1951,73 | 27474,45 | 6669,56 | 38393,6 | 32516,32 | 16964,38 | 13976,65 | 19470,82 | 4586,68 | 7485,99 | 17601,41 |
| **CSMD3_F3640Lfs*61_** | QDVQFMKIT | 40970,51 | 41482,57 | 42133,52 | 44924,48 | 37686,11 | 41705,81 | 37318,88 | 39177,89 | 39790,07 | 40585,32 | 34914,18 | 40682,94 |
| **CSMD3_F3640Lfs*61_** | DVQFMKITM | 27988,41 | 28155,17 | 34518,64 | 26904,05 | 3395,18 | 16169,04 | 4782,3 | 19282,77 | 34259,67 | 14906,03 | 31859,17 | 32923,09 |
| **CSMD3_F3640Lfs*61_** | VQFMKITMA | 32526,52 | 1136,64 | 17620,66 | 27733,7 | 32962,29 | 29964,39 | 2031,59 | 1620,03 | 7480,56 | 7089,46 | 12911,83 | 28409,77 |
| **CSMD3_F3640Lfs*61_** | QFMKITMAK | 31963,09 | 27036,84 | 367,19 | 13810,84 | 29436,1 | 26078,9 | 15316,55 | 23102,6 | 9866,9 | 29147,7 | 36876,57 | 25209,41 |
| **CSMD3_F3640Lfs*61_** | FMKITMAKQ | 36540,95 | 17566,6 | 16807,27 | 40890,34 | 25735,81 | 32866,49 | 13525,58 | 5593,36 | 27395,49 | 38843,19 | 41214,61 | 34139,05 |
| **CSMD3_F3640Lfs*61_** | MKITMAKQL | 41058,38 | 32138,91 | 38335,48 | 35462,03 | 35588,5 | 21624,24 | 4921,03 | 12041,21 | 4163,39 | 827,4 | 13482,04 | 26609,92 |
| **CSMD3_F3640Lfs*61_** | KITMAKQLL | 35029,59 | 10715,92 | 21356,6 | 18405,39 | 37261,59 | 6092,75 | 16622,8 | 10776,61 | 24659,88 | 28480,54 | 27908,58 | 6499,58 |
| **CSMD3_F3640Lfs*61_** | ITMAKQLLK | 7748,88 | 22712,74 | 9,19 | 23341,54 | 27126,78 | 29749,87 | 20557,19 | 22085,33 | 9741,62 | 38770,96 | 38642,8 | 6751,89 |
| **CSMD3_F3640Lfs*61_** | TMAKQLLKI | 21154,22 | 315,74 | 13385,09 | 4770,26 | 19745,99 | 20804,89 | 4448,99 | 3891,2 | 18403,2 | 14912,49 | 27661,16 | 7493,44 |
| **CSMD3_F3640Lfs*61_** | MAKQLLKIP | 42142,16 | 38848,65 | 40557,25 | 43584,82 | 38801,6 | 29308,66 | 9382,36 | 19947,41 | 37010,87 | 37258,76 | 39608,81 | 20283,22 |
| **CSMD3_F3640Lfs*61_** | AKQLLKIPC | 45443,66 | 39712,22 | 42913,34 | 43461,94 | 47107,8 | 34322,74 | 22014,71 | 36020,82 | 22157,13 | 19112,64 | 27996,9 | 41345,03 |
| **CSMD3_F3640Lfs*61_** | KQLLKIPCM | 36284,07 | 1891,49 | 18092,63 | 10956,85 | 33638,55 | 20730,29 | 1901,01 | 602,47 | 2604,61 | 7263,93 | 7060,22 | 12963,2 |
| **CSMD3_F3640Lfs*61_** | QLLKIPCMT | 34752,11 | 2278,99 | 24580,51 | 39927,21 | 40933,72 | 35720,04 | 19694,35 | 24433,08 | 30508,42 | 32837 | 38379,46 | 30312,65 |
| **CSMD3_F3640Lfs*61_** | LLKIPCMTP | 38690,08 | 10828,74 | 25282,35 | 34970,88 | 41075,7 | 30638,43 | 7852,1 | 8261,1 | 34781,07 | 38618,56 | 38716,04 | 32696,24 |
| **CSMD3_F3640Lfs*61_** | LKIPCMTPT | 41522,99 | 26673,62 | 33746,83 | 43104,14 | 35532,31 | 26808,45 | 24604,98 | 19796,68 | 16607,88 | 13090,77 | 29946,89 | 33016,9 |
| **CSMD3_F3640Lfs*61_** | KIPCMTPTQ | 38839,83 | 26762,95 | 18536,09 | 34747,22 | 37437,79 | 31338,15 | 35631,65 | 20993,02 | 35859,82 | 43092,94 | 42736,34 | 29248,78 |
| **CSMD3_F3640Lfs*61_** | IPCMTPTQS | 40638,52 | 41358,89 | 38251,79 | 45923,11 | 45673,86 | 15057,1 | 30584,11 | 39384,02 | 38570,97 | 35831,53 | 42957,48 | 35124,46 |
| **CSMD3_F3640Lfs*61_** | PCMTPTQSQ | 44729,52 | 44213,23 | 41420,23 | 47053,3 | 47096,07 | 42464,85 | 42785,4 | 42367,11 | 45099,32 | 45737,18 | 47300,35 | 39531,31 |
| **CSMD3_F3640Lfs*61_** | CMTPTQSQW | 23469,18 | 18926,62 | 22067,65 | 3424,25 | 22835,2 | 26377,73 | 23620,5 | 2285,61 | 21837,25 | 30404,61 | 32315,7 | 31,13 |
| **CSMD3_F3640Lfs*61_** | MTPTQSQWK | 14571,96 | 33951,55 | 1476,9 | 31832,63 | 16844,23 | 35730,09 | 34019,95 | 28691,48 | 27282,49 | 41997,87 | 42370,77 | 23024,49 |
| **CSMD3_F3640Lfs*61_** | TPTQSQWKG | 42024,23 | 43920,46 | 42671,21 | 43967,07 | 43927,59 | 23279,75 | 30283,48 | 42553,61 | 38587,67 | 33143,6 | 43452,52 | 23840,54 |
| **CSMD3_F3640Lfs*61_** | PTQSQWKGR | 36157,88 | 44741,61 | 26078,62 | 42811,79 | 38855,36 | 45246,44 | 43205,45 | 44491,08 | 40758,28 | 47574,95 | 47803 | 43111,59 |
| **CSMD3_F3640Lfs*61_** | TQSQWKGRR | 36040,71 | 35780,77 | 14073,31 | 39677,43 | 33075,89 | 37887,25 | 36563,48 | 26661,22 | 10849,38 | 36695,46 | 37281,36 | 38209,61 |
| **CSMD3_F3640Lfs*61_** | QSQWKGRRY | 7032,62 | 43640,99 | 19341,69 | 40031,89 | 20064,3 | 32575,14 | 31694,13 | 5217,96 | 27680,33 | 41447,6 | 40225,55 | 10315,92 |
| **CSMD3_F3640Lfs*61_** | SQWKGRRYD | 42442,8 | 28878,68 | 32809,29 | 36918,88 | 43793,77 | 33178,05 | 25778,45 | 17275,38 | 14400,17 | 33447,29 | 26067,06 | 27384,52 |
| **CSMD3_F3640Lfs*61_** | QWKGRRYDL | 42773,34 | 39363,58 | 37908,98 | 10044,31 | 41365,59 | 22453,48 | 838,6 | 30188,93 | 25030,85 | 33324,11 | 40696,14 | 42097,96 |
| **CSMD3_F3640Lfs*61_** | WKGRRYDLI | 32702,25 | 37693,84 | 42565,14 | 26780,63 | 42631,95 | 35219,98 | 3359,33 | 32542,72 | 13408,58 | 14599,09 | 28147,85 | 33324,47 |
| **CSMD3_F3640Lfs*61_** | KGRRYDLIP | 43918,11 | 42232,1 | 37623,36 | 39938,03 | 47379,23 | 33703,77 | 27970,55 | 33325,91 | 32169,87 | 43712,34 | 41976,07 | 23789,78 |
| **CSMD3_F3640Lfs*61_** | GRRYDLIPT | 43977,05 | 38165,79 | 38351,66 | 42470,82 | 42990,49 | 28976,64 | 23410,86 | 34482,06 | 3432,3 | 26827,6 | 37360,9 | 41821,92 |
|  |  |  |  |  |  |  |  |  |  |  |  |  |  |
| **PROTEIN** | **PEPTIDE** | **A*01:01** | **A*02:01** | **A*03:01** | **A*24:02** | **A*26:02** | **B*07:02** | **B*08:02** | **B*27:05** | **B*39:01** | **B*40:01** | **B*58:01** | **B*15:01** |
| **PTEN_267wt_** | HKQNKMLKK | 41398,29 | 43638,62 | 15035,29 | 44533,94 | 42714,62 | 40659,62 | 29428,77 | 5999,54 | 40102,12 | 43021,66 | 41304,79 | 38264,62 |
| **PTEN_267wt_** | KQNKMLKKD | 46074,91 | 42191,89 | 41138,88 | 42164,07 | 45842,69 | 45160,85 | 38275,8 | 24565,34 | 44280,71 | 37531,47 | 30856,65 | 29227,27 |
| **PTEN_267wt_** | QNKMLKKDK | 45042,76 | 47797,32 | 35225,71 | 47305,96 | 45499,26 | 45785,69 | 35809,42 | 39097,02 | 47205,24 | 47857,35 | 45043,25 | 43234,46 |
| **PTEN_267wt_** | NKMLKKDKM | 41159,79 | 44072,31 | 43697,68 | 40521,28 | 41237,34 | 28708,56 | 1749,82 | 20761,49 | 6738,97 | 31923,68 | 40543,65 | 33890,25 |
| **PTEN_267wt_** | KMLKKDKMF | 39262,78 | 16217,57 | 19114,3 | 3996,9 | 36604,25 | 25596,95 | 4274,21 | 10994,85 | 36193,51 | 28180,16 | 4096,72 | 849,23 |
| **PTEN_267wt_** | MLKKDKMFH | 29797,88 | 29352,14 | 2134,61 | 39569,39 | 25813,05 | 29519,98 | 10263,59 | 24290,2 | 37792,28 | 37689,37 | 29853,06 | 3400,29 |
| **PTEN_267wt_** | LKKDKMFHF | 39343,99 | 39370,39 | 40462,13 | 23359,23 | 28201,51 | 37006,86 | 4631,16 | 10461,47 | 27549,47 | 32707,57 | 27309,97 | 5116,49 |
| **PTEN_267wt_** | KKDKMFHFW | 32147,95 | 35637,44 | 37848,32 | 7471,5 | 40969,19 | 37093,45 | 31294,1 | 11135,41 | 24170,4 | 26374,59 | 1196,18 | 30125,62 |
| **PTEN_267wt_** | KDKMFHFWV | 29903,18 | 23749,66 | 31238,61 | 22003,51 | 40975,4 | 33034,41 | 12379,8 | 26173,88 | 33690,27 | 17654,43 | 28221,36 | 32178,59 |
|  |  |  |  |  |  |  |  |  |  |  |  |  |  |
| **PTEN_K267Rfs*9_** | HKQNKMLKR | 43108,34 | 43549 | 28775,42 | 44482,41 | 41650,33 | 40544,07 | 29946,89 | 7839,62 | 38229,45 | 43616,43 | 43177,43 | 41635,02 |
| **PTEN_K267Rfs*9_** | KQNKMLKRT | 43176 | 31790,98 | 32113,88 | 40574,36 | 42567,45 | 39069,52 | 29373,43 | 12292,37 | 38314,33 | 32756,43 | 34160,11 | 18172,48 |
| **PTEN_K267Rfs*9_** | QNKMLKRTK | 43395,67 | 46258,73 | 23766,1 | 45184,3 | 42271,86 | 40971,41 | 24911,7 | 30621,19 | 44169,71 | 46002,2 | 40712,89 | 37240,62 |
| **PTEN_K267Rfs*9_** | NKMLKRTKC | 45012,56 | 43861,1 | 43990,84 | 42749,29 | 45373,9 | 29227,59 | 1213,38 | 20446,72 | 16150,33 | 40236 | 43460,98 | 39851,68 |
| **PTEN_K267Rfs*9_** | KMLKRTKCF | 34131,3 | 20284,97 | 9595,48 | 2337,08 | 32339,48 | 11419,45 | 257,38 | 8080,85 | 29834,01 | 29603,13 | 5841,57 | 203,45 |
| **PTEN_K267Rfs*9_** | MLKRTKCFT | 31723,29 | 14759,98 | 19689,88 | 38685,49 | 37585,53 | 19946,77 | 957,42 | 25874,02 | 34064,89 | 39904,77 | 29928,1 | 11959,93 |
| **PTEN_K267Rfs*9_** | LKRTKCFTF | 37371 | 39686,46 | 36215,04 | 14950,13 | 36316,68 | 21152,17 | 599,98 | 5949,63 | 19677,1 | 29148,96 | 24388,17 | 3268,41 |
| **PTEN_K267Rfs*9_** | KRTKCFTFG | 42024,7 | 39040,79 | 31713,68 | 34898,31 | 43901,45 | 34902,84 | 27499,73 | 486,22 | 33912,64 | 37592,85 | 24823,71 | 33570,57 |
|  |  |  |  |  |  |  |  |  |  |  |  |  |  |
| **PROTEIN** | **PEPTIDE** | **A*01:01** | **A*02:01** | **A*03:01** | **A*24:02** | **A*26:02** | **B*07:02** | **B*08:02** | **B*27:05** | **B*39:01** | **B*40:01** | **B*58:01** | **B*15:01** |
| **APC_1556wt_** | NQEKEAEKT | 42188,25 | 42972,36 | 46827,29 | 45173,55 | 46726,05 | 44868,63 | 41052,16 | 42285,14 | 28768,58 | 36432,77 | 44958,51 | 41102,38 |
| **APC_1556wt_** | QEKEAEKTI | 43766,3 | 42241,69 | 44876,39 | 40172,04 | 41387,98 | 33194,21 | 30872,02 | 39439,46 | 28240,6 | 1709,68 | 38931,54 | 24775,96 |
| **APC_1556wt_** | EKEAEKTID | 48188,86 | 48936,54 | 48446,05 | 48531,05 | 48752,61 | 47500,36 | 44334,89 | 45665,96 | 43643,36 | 46755,38 | 46409,15 | 47359,75 |
| **APC_1556wt_** | KEAEKTIDS | 43036,56 | 41695,88 | 41788,92 | 45173,55 | 44323,37 | 40971,41 | 37494,53 | 37726,5 | 39095,74 | 14796,28 | 41370,98 | 36795,65 |
| **APC_1556wt_** | EAEKTIDSE | 36832,69 | 44841,44 | 44614,48 | 47225,67 | 37138,43 | 41025,08 | 38498,41 | 44538,76 | 37233,78 | 44853,09 | 38003,03 | 41063,7 |
| **APC_1556wt_** | AEKTIDSEK | 40241,22 | 43326,71 | 24725,34 | 45351,32 | 39591,24 | 40120,36 | 38345,43 | 29149,58 | 43563,61 | 23046,93 | 40906,72 | 27486,95 |
| **APC_1556wt_** | EKTIDSEKD | 48291,14 | 48591,99 | 48509,52 | 48497,97 | 48552,05 | 48115,91 | 46161,76 | 45246,94 | 44301,34 | 47414,08 | 44290,31 | 46968,86 |
| **APC_1556wt_** | KTIDSEKDL | 37679,16 | 21611,61 | 34513,05 | 38301,9 | 35376,58 | 27566,16 | 37637,2 | 35587,32 | 36768,99 | 31575,03 | 5139,13 | 18083,83 |
| **APC_1556wt_** | TIDSEKDLL | 10293,62 | 15716,77 | 36890,93 | 35641,67 | 37068,99 | 28363,69 | 30280,84 | 39584,4 | 18897,15 | 29860,49 | 32359,1 | 36045,38 |
|  |  |  |  |  |  |  |  |  |  |  |  |  |  |
| **APC_T1556Nfs*3_** | NQEKEAEKN | 43720,38 | 47072,12 | 47209,84 | 46058,96 | 47141,96 | 46919,57 | 44494,43 | 43075,69 | 42690,58 | 43630,61 | 42946,32 | 42301,15 |
| **APC_T1556Nfs*3_** | QEKEAEKNY | 36662,91 | 46740,21 | 43542,4 | 46590,25 | 34098,81 | 42956,09 | 42270,03 | 40509,88 | 43640,04 | 21797,84 | 39567,68 | 10884,78 |
|  |  |  |  |  |  |  |  |  |  |  |  |  |  |
| **PROTEIN** | **PEPTIDE** | **A*01:01** | **A*02:01** | **A*03:01** | **A*24:02** | **A*26:02** | **B*07:02** | **B*08:02** | **B*27:05** | **B*39:01** | **B*40:01** | **B*58:01** | **B*15:01** |
| **GLI1_274wt_** | KEFVCHWGG | 39965,68 | 32882,5 | 33907,5 | 32872,89 | 42327,7 | 41688,2 | 30466,52 | 15320,87 | 32776,3 | 3806,38 | 21578,43 | 29591,61 |
| **GLI1_274wt_** | EFVCHWGGC | 41540,54 | 37697,12 | 40669,3 | 33763,63 | 26251,88 | 38825,53 | 25631,87 | 38493 | 37593,24 | 42946,32 | 43652,79 | 38125,77 |
| **GLI1_274wt_** | FVCHWGGCS | 25816,68 | 21521,08 | 30936,89 | 42559,6 | 27932,44 | 33171,23 | 30654,68 | 36433,95 | 38381,12 | 41808,36 | 35505,81 | 21670,61 |
| **GLI1_274wt_** | VCHWGGCSR | 38317,64 | 37112,3 | 20964,19 | 42388,21 | 39758,65 | 31651,65 | 34588,57 | 33801,28 | 39293,78 | 42352,44 | 35609,69 | 33231,93 |
| **GLI1_274wt_** | CHWGGCSRE | 41380,38 | 38593,09 | 37985,36 | 32891,74 | 43101,81 | 38635,7 | 35355,53 | 24780,51 | 22230,13 | 40298,72 | 37960,29 | 38014,55 |
| **GLI1_274wt_** | HWGGCSREL | 40420,55 | 33376,8 | 36838,27 | 5910,17 | 39268,73 | 16289,67 | 13010,84 | 23031,73 | 24353,63 | 35993,56 | 34371,43 | 23702,93 |
| **GLI1_274wt_** | WGGCSRELR | 30606,95 | 38065,18 | 29698,42 | 37822,95 | 41147,31 | 38785,64 | 31145,79 | 26257,28 | 39776,3 | 43329,51 | 35021,62 | 38720,23 |
| **GLI1_274wt_** | GGCSRELRP | 44551,29 | 44162,53 | 43259,26 | 45727,77 | 48251,98 | 45635,35 | 40067,43 | 42067 | 45836,24 | 46398,08 | 35577,32 | 44299,88 |
| **GLI1_274wt_** | GCSRELRPF | 36739,95 | 36858,61 | 38120,8 | 32240,96 | 35870,7 | 28915,24 | 28249,76 | 34917,95 | 38986,34 | 34263,76 | 11016,41 | 9907,66 |
|  |  |  |  |  |  |  |  |  |  |  |  |  |  |
| **GLI1_G274Afs*6_** | KEFVCHWGA | 34242,27 | 14556,82 | 26056,34 | 30612,58 | 39137,64 | 34857,94 | 22228,21 | 11037,04 | 19397,85 | 739,43 | 24245,3 | 22287,2 |
| **GLI1_G274Afs*6_** | EFVCHWGAA | 34429,49 | 28488,26 | 33682,25 | 29415,72 | 12137,09 | 25164,17 | 8891,25 | 30113,56 | 18552,34 | 37110,32 | 41104,61 | 26716,37 |
| **GLI1_G274Afs*6_** | FVCHWGAAP | 29578,17 | 9075,83 | 32111,11 | 39151,62 | 25543,01 | 24826,93 | 25227,69 | 34489,89 | 29233,28 | 34922,49 | 29951,43 | 12514,2 |
| **GLI1_G274Afs*6_** | VCHWGAAPG | 40905,85 | 34625,62 | 38335,06 | 42087,95 | 45003,3 | 28664,5 | 31197,05 | 38619,83 | 36321,39 | 37752,62 | 22697,51 | 23529,19 |
| **GLI1_G274Afs*6_** | CHWGAAPGS | 40604,23 | 32196,69 | 36862,2 | 35983,44 | 43507,57 | 36803,22 | 38431,01 | 22213,3 | 12127,24 | 36160,24 | 32527,23 | 37954,95 |
|  |  |  |  |  |  |  |  |  |  |  |  |  |  |
| **PROTEIN** | **PEPTIDE** | **A*01:01** | **A*02:01** | **A*03:01** | **A*24:02** | **A*26:02** | **B*07:02** | **B*08:02** | **B*27:05** | **B*39:01** | **B*40:01** | **B*58:01** | **B*15:01** |
| **B2M_15wt_** | LAVLALLSL | 30287,41 | 5858,66 | 29929,71 | 26611,65 | 23115,86 | 6522,34 | 3814,95 | 25827,59 | 5795,37 | 19583,64 | 1232,57 | 1948,68 |
| **B2M_15wt_** | AVLALLSLS | 33143,96 | 7768,27 | 12419,11 | 39422,83 | 27134,71 | 31004,58 | 31474,74 | 24480,96 | 39973,9 | 35457,43 | 16723,65 | 14974,9 |
| **B2M_15wt_** | VLALLSLSG | 30040,03 | 5033,47 | 21676,25 | 31703,39 | 40282,16 | 27780,85 | 13391,17 | 28978,82 | 30302,16 | 35842 | 14908,94 | 2308,78 |
| **B2M_15wt_** | LALLSLSGL | 31540,19 | 3972,15 | 29386,77 | 28236,61 | 26948,63 | 6400,9 | 5337,18 | 21343,21 | 10069,99 | 22070,99 | 2222,76 | 4561,39 |
| **B2M_15wt_** | ALLSLSGLE | 35899,04 | 11014,14 | 16965,12 | 41098,38 | 39846,08 | 37339,89 | 33822,87 | 29900,59 | 41826,91 | 37806,59 | 32690,59 | 18118,49 |
| **B2M_15wt_** | LLSLSGLEA | 13752,25 | 1211,08 | 18086,18 | 36620,5 | 38282,84 | 18720,11 | 12496,35 | 28840,26 | 19794,33 | 32289,84 | 24265 | 5250,18 |
| **B2M_15wt_** | LSLSGLEAI | 21318,06 | 2555,48 | 31427,44 | 16755,88 | 28202,42 | 17269,22 | 18640,87 | 23209,58 | 10936,13 | 21373,03 | 325,61 | 4888,55 |
| **B2M_15wt_** | SLSGLEAIQ | 35614,3 | 17354,44 | 21914,18 | 44417,48 | 38743,29 | 36849,04 | 38472,2 | 36733,2 | 39314,21 | 39042,07 | 29391,87 | 16349 |
| **B2M_15wt_** | LSGLEAIQR | 25791,29 | 38311,44 | 20817,05 | 42679,96 | 41210,59 | 43205,92 | 41830,07 | 36445,39 | 45230,77 | 44699,5 | 25404,38 | 38929,43 |
|  |  |  |  |  |  |  |  |  |  |  |  |  |  |
| **B2M_L15Ffs*41_** | LAVLALLSF | 26149,82 | 24174,33 | 29493,81 | 12629,28 | 13769,96 | 17272,96 | 12021,3 | 26712,62 | 23204,8 | 27379,49 | 219,7 | 148,96 |
| **B2M_L15Ffs*41_** | AVLALLSFW | 28532,06 | 17037,23 | 14041,83 | 7605,84 | 14377,44 | 32131,97 | 27812,12 | 23003,58 | 40835,53 | 35299,34 | 32,16 | 5276,1 |
| **B2M_L15Ffs*41_** | VLALLSFWP | 34860,21 | 2370 | 24831,24 | 34782,22 | 44741,61 | 42226,14 | 29427,18 | 35349,41 | 39232,61 | 36667,29 | 24531,87 | 26527,12 |
| **B2M_L15Ffs*41_** | LALLSFWPG | 28584,29 | 9192,94 | 27664,46 | 22918,36 | 39036,98 | 24011,66 | 3868,45 | 22076,97 | 20404,5 | 31141,09 | 1190,82 | 13396,1 |
| **B2M_L15Ffs*41_** | ALLSFWPGG | 36804,81 | 1614,12 | 19162,13 | 29584,89 | 38689,26 | 36536,59 | 29098,53 | 19082,27 | 39086,03 | 33272,95 | 19691,15 | 13526,31 |
| **B2M_L15Ffs*41_** | LLSFWPGGY | 615,73 | 14871,88 | 802,7 | 28314,33 | 2613,16 | 32238,51 | 31683,16 | 21872,73 | 34927,4 | 35590,79 | 12138,53 | 191,83 |
| **B2M_L15Ffs*41_** | LSFWPGGYP | 21483,38 | 17314,69 | 15662,62 | 35311,18 | 27264,5 | 25954,49 | 32628,75 | 28071,2 | 32080,89 | 27210,57 | 4175,08 | 11674,94 |
| **B2M_L15Ffs*41_** | SFWPGGYPA | 29150,22 | 1228,04 | 14455,9 | 7702,65 | 30536,49 | 9682,47 | 8133,56 | 14251,22 | 4042,39 | 18731,45 | 24520,47 | 13821,16 |
| **B2M_L15Ffs*41_** | FWPGGYPAY | 18226,64 | 27607,64 | 31358,14 | 4798,41 | 6305,62 | 29825,29 | 26284,56 | 27292,24 | 32406,38 | 35505,81 | 35040,57 | 6520,85 |
| **B2M_L15Ffs*41_** | WPGGYPAYS | 36074,64 | 33252,08 | 36781,73 | 41947,93 | 38942,49 | 6273,64 | 19570,3 | 33671,7 | 25366,2 | 40378,61 | 33633,46 | 36940,05 |
| **B2M_L15Ffs*41_** | PGGYPAYSK | 39715,65 | 43659,88 | 19987,16 | 39139,76 | 46619,01 | 45200,94 | 44040,38 | 40745,48 | 47020,2 | 47111,87 | 37748,94 | 42185,07 |
| **B2M_L15Ffs*41_** | GGYPAYSKD | 44183,55 | 41447,14 | 40942,14 | 41206,13 | 46273,77 | 44416,02 | 43071,96 | 39704,92 | 45387,16 | 43771,49 | 26134,55 | 36557,16 |
| **B2M_L15Ffs*41_** | GYPAYSKDS | 45200,94 | 44851,62 | 43691,54 | 32191,81 | 45735,2 | 43836,44 | 42487,83 | 42765,95 | 45548,02 | 46406,64 | 45901,26 | 44456,43 |
| **B2M_L15Ffs*41_** | YPAYSKDSG | 38891,97 | 41362,02 | 40810,78 | 42564,68 | 40497,61 | 3013,23 | 14326,35 | 34584,06 | 17139,84 | 39638,4 | 26878,16 | 30278,23 |
| **B2M_L15Ffs*41_** | PAYSKDSGL | 37771 | 33492,57 | 39037,41 | 40042,73 | 38518,85 | 25794,91 | 19681,78 | 38297,33 | 33327,01 | 37895,86 | 27806,11 | 28574,08 |
| **B2M_L15Ffs*41_** | AYSKDSGLL | 35507,34 | 32217,96 | 34679,61 | 1967,66 | 32996,19 | 24056,38 | 28446,99 | 26457,48 | 19958,85 | 27793,16 | 26885,43 | 22157,13 |
| **B2M_L15Ffs*41_** | YSKDSGLLT | 12983,56 | 27151,74 | 30638,75 | 38949,67 | 25178,07 | 25169,9 | 21717,33 | 30397,04 | 30072,22 | 34954,24 | 14259,55 | 12217,19 |
| **B2M_L15Ffs*41_** | SKDSGLLTS | 36684,34 | 38571,79 | 40786,95 | 43446,89 | 44561,4 | 37275,7 | 36561,12 | 33339,99 | 16014,08 | 31076,45 | 39975,62 | 38630,27 |
| **B2M_L15Ffs*41_** | KDSGLLTSS | 39773,3 | 38340,45 | 37587,95 | 43680,18 | 41446,69 | 39347,82 | 39579,68 | 35568,46 | 42435 | 34665,75 | 35392,28 | 35713,09 |
| **B2M_L15Ffs*41_** | DSGLLTSSS | 29268,72 | 41025,95 | 40127,3 | 43981,82 | 33067,66 | 34649,61 | 26934,35 | 40053,56 | 36317,05 | 44255,33 | 31040,5 | 34027,68 |
| **B2M_L15Ffs*41_** | SGLLTSSSR | 37913,09 | 35670,99 | 11311,6 | 39114,38 | 39113,95 | 28815,61 | 27123,84 | 23280,5 | 37644,94 | 41505,48 | 28782,59 | 26726,19 |
| **B2M_L15Ffs*41_** | GLLTSSSRE | 42236,65 | 22559,18 | 25163,08 | 45044,21 | 44790,51 | 39628,54 | 35997,06 | 34442,17 | 44848,23 | 42736,8 | 37780 | 25716,6 |
| **B2M_L15Ffs*41_** | LLTSSSREW | 29491,24 | 22776,22 | 25954,21 | 12929,17 | 30414,5 | 23441,27 | 24333,09 | 28747,12 | 34287,5 | 34082,21 | 120,25 | 2809,9 |
| **B2M_L15Ffs*41_** | LTSSSREWK | 14278,07 | 32814,62 | 359,93 | 38247,64 | 27063,16 | 33395,58 | 34428,38 | 22302,15 | 41539,17 | 40475,26 | 10790,03 | 26803,23 |
| **B2M_L15Ffs*41_** | TSSSREWKV | 11945,58 | 12523,68 | 29179,88 | 26600,7 | 30512,06 | 32108,33 | 12772,05 | 33196,01 | 25510,7 | 36561,9 | 3367,45 | 33117,09 |
| **B2M_L15Ffs*41_** | SSSREWKVK | 22378,79 | 40139,9 | 2474,34 | 40532,23 | 39130,02 | 36195,84 | 36053,57 | 25197,41 | 42270,03 | 41659,79 | 14352,42 | 28593,56 |
| **B2M_L15Ffs*41_** | SSREWKVKF | 25727,46 | 36553,6 | 23418,45 | 14274,36 | 13385,67 | 8280,79 | 13149,25 | 21546,93 | 35264,6 | 33026,55 | 1574,67 | 2243,78 |
| **B2M_L15Ffs*41_** | SREWKVKFP | 44255,82 | 45192,14 | 43307,49 | 44372,79 | 46493,57 | 42331,82 | 30567,23 | 19067,41 | 32303,11 | 40656,97 | 43624 | 45047,14 |
| **B2M_L15Ffs*41_** | REWKVKFPE | 41573,79 | 32302,42 | 33374,99 | 37243,87 | 44098,55 | 34035,77 | 26857,53 | 7076,51 | 29540,43 | 4801,43 | 33435,72 | 29372,16 |
| **B2M_L15Ffs*41_** | EWKVKFPEL | 41200,79 | 34927,02 | 38226,14 | 4659,21 | 30373,06 | 28123,5 | 386,96 | 29593,53 | 28187,18 | 37793,91 | 42621,81 | 28232,95 |
| **B2M_L15Ffs*41_** | WKVKFPELL | 34807,06 | 24528,15 | 40249,91 | 26750,51 | 34261,15 | 29736,37 | 13486,56 | 3406,33 | 232,4 | 5128,57 | 24092,07 | 14032,56 |
| **B2M_L15Ffs*41_** | KVKFPELLC | 38609,8 | 20479,94 | 14139,86 | 33344,31 | 41417,11 | 23308,75 | 28959,71 | 31031,09 | 41569,29 | 37887,25 | 11376,9 | 21049,88 |
| **B2M_L15Ffs*41_** | VKFPELLCV | 36083,22 | 11926,59 | 34467,9 | 33068,02 | 38365,78 | 36930,85 | 25731,91 | 17242,15 | 8992,55 | 23663,99 | 27785,35 | 21854,99 |
| **B2M_L15Ffs*41_** | KFPELLCVW | 35236 | 28962,21 | 32887,48 | 101,03 | 29502,11 | 31595,52 | 28498,42 | 26585,16 | 36587,63 | 33433,18 | 1518,35 | 14762,07 |
| **B2M_L15Ffs*41_** | FPELLCVWV | 27850,37 | 15770,43 | 39135,11 | 38815,04 | 40450,75 | 10733,32 | 8248,15 | 33724,92 | 9279,48 | 31775,86 | 33936,48 | 39386,16 |
| **B2M_L15Ffs*41_** | PELLCVWVS | 42469,45 | 39677,02 | 42660,59 | 39488,98 | 45948,98 | 44940,04 | 39122,82 | 36988,44 | 39619,95 | 23896,59 | 41548,17 | 41697,23 |
| **B2M_L15Ffs*41_** | ELLCVWVSS | 33588,74 | 7949,81 | 28503,05 | 40591,48 | 27542,61 | 37230,56 | 18367,79 | 33475,9 | 31632,81 | 38118,76 | 35042,09 | 25499,38 |
| **B2M_L15Ffs*41_** | LLCVWVSSI | 25088,6 | 137,08 | 16848,96 | 16074,67 | 28501,2 | 25909,32 | 5508,26 | 24100,42 | 21021,89 | 33154,38 | 13628,56 | 2625,23 |
| **B2M_L15Ffs*41_** | LCVWVSSIR | 36947,65 | 30068,3 | 19222,56 | 42653,65 | 34525,37 | 36878,16 | 37416,31 | 32439,02 | 40392,15 | 40583,15 | 28233,57 | 30266,1 |
| **B2M_L15Ffs*41_** | CVWVSSIRH | 26777,73 | 30972,73 | 3021,36 | 41599,43 | 28186,56 | 32990,82 | 32754,32 | 23201,8 | 42163,14 | 38751,66 | 22508,21 | 13718,66 |
|  |  |  |  |  |  |  |  |  |  |  |  |  |  |
| **PROTEIN** | **PEPTIDE** | **A*01:01** | **A*02:01** | **A*03:01** | **A*24:02** | **A*26:02** | **B*07:02** | **B*08:02** | **B*27:05** | **B*39:01** | **B*40:01** | **B*58:01** | **B*15:01** |
| **BRD3_24wt_** | PGPVNPPPP | 46227,71 | 45425,49 | 46440,29 | 43723,22 | 48692,53 | 45176 | 43733,15 | 47172,07 | 46122,8 | 47004,44 | 44117,16 | 45912,18 |
| **BRD3_24wt_** | GPVNPPPPE | 46075,92 | 43875,81 | 41783,93 | 47755,95 | 44368,96 | 7801,12 | 37185,88 | 42194,64 | 36569,42 | 40381,21 | 41791,62 | 40936,39 |
| **BRD3_24wt_** | PVNPPPPEV | 30806,96 | 10460 | 31865,02 | 35421,76 | 37898,74 | 32704,38 | 38657,04 | 42082,49 | 38284,91 | 39227,09 | 29332,14 | 35549,61 |
| **BRD3_24wt_** | VNPPPPEVS | 43595,2 | 43578,7 | 44333,46 | 43473,68 | 45730,24 | 42954,68 | 43503,8 | 45796,59 | 41408,14 | 44182,13 | 43061,71 | 42462,55 |
| **BRD3_24wt_** | NPPPPEVSN | 45956,92 | 45918,17 | 45098,33 | 46864,78 | 44962,89 | 27248,87 | 38781,04 | 45212,2 | 38977,49 | 45633,85 | 43293,89 | 44286,48 |
| **BRD3_24wt_** | PPPPEVSNP | 47014,62 | 45156,95 | 46339,89 | 46438,76 | 47488,02 | 39821,52 | 44261,55 | 47408,98 | 45739,15 | 47171,55 | 45740,15 | 46197,73 |
| **BRD3_24wt_** | PPPEVSNPS | 44766,31 | 41435,04 | 44659,88 | 45777,28 | 43676,89 | 27205,55 | 38318,48 | 44385,76 | 37776,73 | 45220,01 | 45037,39 | 42876,22 |
| **BRD3_24wt_** | PPEVSNPSK | 41820,57 | 46958,69 | 33245,97 | 46845,53 | 46879,5 | 32127,1 | 40960,76 | 43497,69 | 45016,94 | 46698,27 | 44227,09 | 44428,54 |
| **BRD3_24wt_** | PEVSNPSKP | 47385,9 | 48371,69 | 47586,79 | 47633,65 | 47897,24 | 46482 | 45542,12 | 46513,7 | 45712,43 | 38637,37 | 46702,82 | 45737,68 |
|  |  |  |  |  |  |  |  |  |  |  |  |  |  |
| **BRD3_P24Rfs*24_** | PGPVNPPPR | 42230,25 | 45292,98 | 36101,98 | 41998,33 | 46174,74 | 44131,95 | 42458,88 | 43200,32 | 46810,05 | 47530,7 | 44770,18 | 44807,01 |
| **BRD3_P24Rfs*24_** | GPVNPPPRR | 44125,75 | 45513,05 | 28843,39 | 46925,69 | 39635,38 | 25739,71 | 41635,02 | 39463,38 | 44404,51 | 44318,6 | 42675,36 | 43922,35 |
| **BRD3_P24Rfs*24_** | PVNPPPRRS | 43233,99 | 44532,96 | 39431,78 | 47342,34 | 45555,43 | 43669,31 | 44941,98 | 45958,9 | 47869,76 | 48014,48 | 41766,77 | 43565,96 |
| **BRD3_P24Rfs*24_** | VNPPPRRSP | 45847,15 | 44538,76 | 43905,73 | 42157,68 | 45918,64 | 36499,85 | 35210,83 | 44098,55 | 40966,52 | 44225,18 | 42665,18 | 41103,26 |
| **BRD3_P24Rfs*24_** | NPPPRRSPT | 44189,8 | 42209,26 | 41792,07 | 44688,38 | 38393,18 | 1277,92 | 2645,1 | 38381,96 | 19145,35 | 43729,37 | 45555,43 | 42244,88 |
| **BRD3_P24Rfs*24_** | PPPRRSPTP | 46868,86 | 46177,71 | 45971,83 | 44775,5 | 47898,26 | 31164,68 | 30406,59 | 45444,66 | 43405,52 | 46746,79 | 46107,34 | 45852,62 |
| **BRD3_P24Rfs*24_** | PPRRSPTPA | 42749,29 | 44641,51 | 38615,65 | 46110,82 | 45118,35 | 598,03 | 9811,02 | 38934,91 | 35541,93 | 44211,77 | 43271,42 | 37559,92 |
| **BRD3_P24Rfs*24_** | PRRSPTPAS | 46746,79 | 46774,62 | 42162,68 | 46056,49 | 45981,29 | 33267,55 | 34322,74 | 22283,82 | 39482,16 | 45537,18 | 45634,85 | 41056,61 |
| **BRD3_P24Rfs*24_** | RRSPTPASP | 44579,25 | 41903,91 | 40288,71 | 41798,41 | 45332,2 | 32164,66 | 34089,97 | 6167,57 | 19199,7 | 31178,17 | 34410,89 | 35203,61 |
| **BRD3_P24Rfs*24_** | RSPTPASPA | 23773,57 | 14496,15 | 17813,69 | 29626,21 | 30486,65 | 5056,23 | 22298,54 | 24882,33 | 21464,8 | 29401,72 | 13790,39 | 8981,16 |
| **BRD3_P24Rfs*24_** | SPTPASPAA | 36619,71 | 30880,04 | 32935,55 | 42861,84 | 33705,59 | 82,17 | 13525,58 | 35074,33 | 5050 | 32530,4 | 33767,64 | 30248,11 |
| **BRD3_P24Rfs*24_** | PTPASPAAR | 31207,54 | 40885,04 | 23331,7 | 41273,07 | 28394,71 | 41048,61 | 43659,4 | 40282,61 | 44884,64 | 46164,73 | 42117,54 | 39808,17 |
| **BRD3_P24Rfs*24_** | TPASPAARP | 42417,11 | 41685,96 | 39914,26 | 44495,87 | 39877,14 | 8205,43 | 32150,04 | 41023,73 | 34452,23 | 41567,5 | 38364,1 | 40631,91 |
| **BRD3_P24Rfs*24_** | PASPAARPT | 41336,52 | 40930,63 | 41670,16 | 45944,97 | 45444,66 | 31508,47 | 38921,85 | 42480,48 | 39771,58 | 43865,37 | 30736,71 | 37957 |
| **BRD3_P24Rfs*24_** | ASPAARPTS | 37278,93 | 37055,34 | 33349,73 | 37511,58 | 39800,83 | 27826,58 | 36809,18 | 38919,74 | 39847,37 | 41425,63 | 26321,84 | 29324,52 |
| **BRD3_P24Rfs*24_** | SPAARPTSC | 39338,03 | 38975,38 | 35591,96 | 42539,81 | 39192,73 | 58,76 | 1031,16 | 36128,56 | 11640,37 | 38384,05 | 36409,12 | 33866,81 |
| **BRD3_P24Rfs*24_** | PAARPTSCS | 37695,08 | 43652,79 | 40462,57 | 45601,78 | 44087,09 | 36336,71 | 38778,09 | 42840,98 | 44247,69 | 46126,29 | 31985,24 | 38902,9 |
| **BRD3_P24Rfs*24_** | AARPTSCST | 38264,62 | 33804,92 | 29312,46 | 43966,59 | 38011,66 | 1507,4 | 11179,35 | 34833,06 | 32047,6 | 36716,5 | 21793,82 | 10461,7 |
| **BRD3_P24Rfs*24_** | ARPTSCSTC | 44256,76 | 39308,68 | 43450,17 | 34750,6 | 42950,03 | 35670,6 | 31529,27 | 17346,74 | 22502,13 | 36455,64 | 42463,94 | 38143,08 |
| **BRD3_P24Rfs*24_** | RPTSCSTCR | 37522,54 | 43164,34 | 21053,53 | 42121,64 | 40193,78 | 7729,7 | 27180,26 | 25053,88 | 36142,24 | 41533,79 | 34355,08 | 39031,09 |
| **BRD3_P24Rfs*24_** | PTSCSTCRM | 7612,51 | 32297,17 | 32658,05 | 33436,07 | 21796,42 | 31915,74 | 31380,55 | 37434,15 | 37170,18 | 39904,33 | 12632,42 | 19159,44 |
| **BRD3_P24Rfs*24_** | TSCSTCRMW | 19926,27 | 36883,75 | 31036,47 | 17277,63 | 30391,12 | 35618,92 | 32413,4 | 28924,01 | 39449,71 | 40500,66 | 30,06 | 14105,48 |
| **BRD3_P24Rfs*24_** | SCSTCRMWW | 28230,82 | 35237,14 | 32638,62 | 22089,63 | 37987 | 30257,59 | 24820,75 | 31889,51 | 39381,48 | 39335,06 | 209,5 | 27693,22 |
|  |  |  |  |  |  |  |  |  |  |  |  |  |  |
| **PROTEIN** | **PEPTIDE** | **A*01:01** | **A*02:01** | **A*03:01** | **A*24:02** | **A*26:02** | **B*07:02** | **B*08:02** | **B*27:05** | **B*39:01** | **B*40:01** | **B*58:01** | **B*15:01** |
| **SALL4_995wt_** | ISVIQSGGV | 23541,91 | 14541,08 | 31283,26 | 37987 | 24534,26 | 26096,4 | 27903,76 | 32180,33 | 33542,61 | 35664,82 | 7749,13 | 14152,41 |
| **SALL4_995wt_** | SVIQSGGVP | 37515,23 | 31222,74 | 33812,62 | 43737,89 | 23688,58 | 19606,76 | 31848,48 | 38362,05 | 32892,46 | 30955,63 | 28094,01 | 9950,85 |
| **SALL4_995wt_** | VIQSGGVPT | 34612,51 | 12951,01 | 28225,32 | 40824,92 | 39244,07 | 11976,37 | 27728 | 35773,02 | 26626,05 | 35605,83 | 34687,12 | 12464,61 |
| **SALL4_995wt_** | IQSGGVPTL | 34428,76 | 179,86 | 28070,91 | 9995,31 | 29296,92 | 15618,45 | 20121,91 | 10370,53 | 117,03 | 1506,01 | 12286,93 | 295,2 |
| **SALL4_995wt_** | QSGGVPTLP | 32917,74 | 39071,21 | 39769,42 | 42332,29 | 44531,52 | 41780,32 | 41762,25 | 42747,45 | 42415,71 | 41105,04 | 12573,51 | 37460,47 |
| **SALL4_995wt_** | SGGVPTLPV | 28209,13 | 9061,2 | 26378,29 | 26889,8 | 40736,23 | 12959,42 | 15876,58 | 29669,51 | 20903,95 | 34023,64 | 18892,45 | 19686,68 |
| **SALL4_995wt_** | GGVPTLPVS | 43226,96 | 31782,04 | 38967,79 | 43563,16 | 44283,11 | 37699,55 | 41051,26 | 38478,43 | 40201,17 | 39080,51 | 25324,52 | 27922,48 |
| **SALL4_995wt_** | GVPTLPVSL | 37810,68 | 7182,03 | 32715,35 | 24213,58 | 24890,68 | 15386,48 | 23692,94 | 32252,13 | 20231,72 | 24594,6 | 29215,26 | 11842,49 |
| **SALL4_995wt_** | VPTLPVSLG | 41020,64 | 39586,95 | 37042,91 | 40816,1 | 41218,17 | 6864,97 | 29481,68 | 34828,91 | 34892,64 | 40995,78 | 26058,88 | 36970,05 |
|  |  |  |  |  |  |  |  |  |  |  |  |  |  |
| **SALL4_V995Ffs*14_** | ISVIQSGGF | 18573,03 | 32941,25 | 28125,93 | 16953,01 | 9594,97 | 21989 | 26868,27 | 26360,03 | 35916,92 | 33395,94 | 701,19 | 403,99 |
| **SALL4_V995Ffs*14_** | SVIQSGGFL | 28132,03 | 7935,2 | 18712,82 | 25117,12 | 893,57 | 2918,73 | 16592,25 | 21079,97 | 11283,61 | 16372,9 | 15784,61 | 1102,2 |
| **SALL4_V995Ffs*14_** | VIQSGGFLP | 30485,64 | 17108,72 | 27402,31 | 30173,26 | 42165,89 | 33616,71 | 35460,89 | 35985,36 | 38523,42 | 36519,2 | 24582,09 | 26389,71 |
| **SALL4_V995Ffs*14_** | IQSGGFLPS | 31386,32 | 5184,93 | 20738,36 | 29078,7 | 35764,51 | 25652,96 | 27587,94 | 15829,24 | 7186,62 | 17217,36 | 24328,61 | 2000,29 |
| **SALL4_V995Ffs*14_** | QSGGFLPSR | 30916,49 | 33181,99 | 7306,5 | 36847,85 | 27230,58 | 37006,05 | 38289,06 | 26971,67 | 40795,34 | 41027,73 | 20796,34 | 31589,02 |
| **SALL4_V995Ffs*14_** | SGGFLPSRF | 28864,3 | 38852,86 | 33045,85 | 9181,01 | 34335,76 | 28066,66 | 21679,76 | 28005,38 | 37837,27 | 37860,21 | 7378,96 | 7805,85 |
| **SALL4_V995Ffs*14_** | GGFLPSRFP | 43694,84 | 32451,3 | 35840,82 | 42656,87 | 46534,85 | 39320,17 | 38451,79 | 36941,64 | 42088,87 | 38162,91 | 24124,67 | 33825,79 |
| **SALL4_V995Ffs*14_** | GFLPSRFPW | 34880,58 | 24208,09 | 22958,08 | 183,53 | 35208,17 | 25246 | 20572,76 | 15480,33 | 27337,76 | 26398,85 | 780,33 | 15673,82 |
| **SALL4_V995Ffs*14_** | FLPSRFPWG | 33084,85 | 1786,52 | 33402,08 | 14768,29 | 26208,74 | 30509,09 | 4377,98 | 29705,49 | 33162,61 | 38201,75 | 34866,23 | 17005,37 |
| **SALL4_V995Ffs*14_** | LPSRFPWGP | 37428,47 | 31211,58 | 37400,53 | 39123,25 | 42854,41 | 15978,78 | 19519,34 | 33668,05 | 24044,67 | 36096,51 | 18386,68 | 37924,17 |
| **SALL4_V995Ffs*14_** | PSRFPWGPP | 35520,39 | 39238,15 | 35096,73 | 40660,49 | 42611,67 | 30488,95 | 33230,86 | 38572,64 | 43991,32 | 43189,57 | 30129,21 | 32266,78 |
| **SALL4_V995Ffs*14_** | SRFPWGPPP | 41857,24 | 29743,12 | 33128,55 | 34017,36 | 38942,09 | 29647,38 | 28326,58 | 1040,1 | 4800,18 | 22665,36 | 35602,36 | 28995,76 |
| **SALL4_V995Ffs*14_** | RFPWGPPPL | 33097,38 | 10256,26 | 21192,03 | 317,43 | 34405,3 | 6086,17 | 10558,47 | 20123,21 | 7813,11 | 15057,43 | 25877,38 | 14945,6 |
|  |  |  |  |  |  |  |  |  |  |  |  |  |  |
| **PROTEIN** | **PEPTIDE** | **A*01:01** | **A*02:01** | **A*03:01** | **A*24:02** | **A*26:02** | **B*07:02** | **B*08:02** | **B*27:05** | **B*39:01** | **B*40:01** | **B*58:01** | **B*15:01** |
| **ARID1A2_2141wt_** | DLILATPPF | 25452,8 | 13422,07 | 29734,1 | 8301,96 | 424,6 | 15206,08 | 5610,27 | 28979,46 | 8988,56 | 19962,53 | 23609,76 | 468,63 |
| **ARID1A2_2141wt_** | LILATPPFS | 33710,7 | 6045,47 | 17205,62 | 39677,02 | 41292,72 | 33642,57 | 32861,15 | 33570,57 | 40775,03 | 40818,31 | 20738,59 | 24255,55 |
| **ARID1A2_2141wt_** | ILATPPFSR | 28007,8 | 12457,19 | 242,56 | 27166,14 | 31143,1 | 31317,11 | 29792,4 | 17097,43 | 34527,99 | 38659,53 | 28918,69 | 22918,36 |
| **ARID1A2_2141wt_** | LATPPFSRL | 26799,75 | 14123,19 | 27427,52 | 16424,71 | 16900,26 | 3941,71 | 11272,01 | 24362,86 | 11429,46 | 22055 | 1750,71 | 7519,19 |
| **ARID1A2_2141wt_** | ATPPFSRLE | 29360,4 | 32242,71 | 23947,05 | 33498,36 | 29545,86 | 37066,17 | 38043,34 | 39283,6 | 43151,26 | 41125,95 | 27153,21 | 30181,41 |
| **ARID1A2_2141wt_** | TPPFSRLEK | 34044,62 | 41668,82 | 16612,19 | 40135,11 | 32900,99 | 10122,32 | 27259,19 | 35272,61 | 36640,71 | 42211,08 | 38884,82 | 39104,21 |
| **ARID1A2_2141wt_** | PPFSRLEKL | 42726,17 | 36015,36 | 40363,74 | 34276,36 | 39298,04 | 4642,3 | 6439,8 | 37409,43 | 24899,02 | 38589,34 | 34846,25 | 39492,83 |
| **ARID1A2_2141wt_** | PFSRLEKLY | 19710,34 | 44344,96 | 28456,82 | 22741,99 | 35118,39 | 45291,98 | 41570,66 | 35166,3 | 45957,93 | 45112,5 | 28620,48 | 25776,77 |
| **ARID1A2_2141wt_** | FSRLEKLYS | 24527,36 | 31174,79 | 26964,09 | 42622,75 | 38299,82 | 23828,17 | 14030,14 | 31761,41 | 38568,88 | 41933,84 | 13818,47 | 20792,06 |
|  |  |  |  |  |  |  |  |  |  |  |  |  |  |
| **ARID1A2_F2141Sfs*59_** | DLILATPPS | 35157,17 | 9619,6 | 33009,77 | 40077,41 | 15780,16 | 29390,27 | 18690,76 | 37052,14 | 20950,37 | 36679,59 | 38338,38 | 18906,76 |
| **ARID1A2_F2141Sfs*59_** | LILATPPSA | 33866,44 | 860,94 | 19052,36 | 38777,69 | 37135,22 | 14228,56 | 12771,78 | 27968,75 | 24050,92 | 34852,27 | 20329,37 | 9615,34 |
| **ARID1A2_F2141Sfs*59_** | ILATPPSAA | 25439,57 | 815,94 | 12295,7 | 37774,27 | 33982,06 | 6757,08 | 10848,44 | 28795,68 | 15948,2 | 32203,31 | 29775,96 | 4841,86 |
| **ARID1A2_F2141Sfs*59_** | LATPPSAAW | 25302,61 | 28993,26 | 29795,95 | 14419,97 | 17309,44 | 7602,14 | 24297,04 | 28801,9 | 18275,8 | 27769,73 | 7,45 | 2881,67 |
| **ARID1A2_F2141Sfs*59_** | ATPPSAAWR | 22405,43 | 27130,02 | 2969,6 | 31350,36 | 13734,99 | 35067,88 | 38129,48 | 29135,71 | 41680,53 | 39860,73 | 28535,46 | 28488,57 |
| **ARID1A2_F2141Sfs*59_** | TPPSAAWRS | 39555,71 | 36491,16 | 40357,21 | 38059 | 38707,68 | 21127,24 | 30230,76 | 39395,96 | 30230,76 | 41801,13 | 38191,82 | 42062,9 |
| **ARID1A2_F2141Sfs*59_** | PPSAAWRSC | 44423,73 | 43828,84 | 41663,84 | 43311,71 | 46696,73 | 10977,85 | 31815,08 | 41090,38 | 36945,64 | 43798,52 | 38436,41 | 43022,13 |
| **ARID1A2_F2141Sfs*59_** | PSAAWRSCI | 23221,14 | 34437,33 | 35899,04 | 25192,51 | 37012,06 | 25925,01 | 26222,35 | 35500,04 | 37493,73 | 42194,18 | 11366,08 | 29092,24 |
| **ARID1A2_F2141Sfs*59_** | SAAWRSCIA | 19475,88 | 16558 | 22257,81 | 36712,15 | 24476,73 | 2637,28 | 891,77 | 27178,49 | 9393,73 | 27579,88 | 10532 | 8903,28 |
| **ARID1A2_F2141Sfs*59_** | AAWRSCIAL | 31323,56 | 2427,98 | 21903,28 | 21554,63 | 34114,31 | 1769,36 | 2659,48 | 12171,41 | 3103,1 | 10051,16 | 3881,02 | 3360,93 |
| **ARID1A2_F2141Sfs*59_** | AWRSCIALW | 36085,97 | 31763,83 | 24277,33 | 265,15 | 27467,01 | 28628,55 | 18906,36 | 13697,15 | 40429,76 | 37062,56 | 4105,82 | 8713,91 |
| **ARID1A2_F2141Sfs*59_** | WRSCIALWC | 37345,14 | 33399,56 | 40354,14 | 37073,39 | 44418,43 | 37403,38 | 22884,17 | 2273,75 | 7406,79 | 30762 | 32346,14 | 36251,51 |
| **ARID1A2_F2141Sfs*59_** | RSCIALWCA | 16312,25 | 7836,99 | 16910,87 | 23380,72 | 39338,89 | 31445,12 | 20594,14 | 17339,05 | 28411,6 | 32619,56 | 1295,94 | 18583,08 |
| **ARID1A2_F2141Sfs*59_** | SCIALWCAS | 33747,55 | 20958,3 | 32500,5 | 40831,55 | 37701,6 | 28322,91 | 23724,74 | 33128,55 | 28673,48 | 33293,13 | 21776,85 | 22129,81 |
| **ARID1A2_F2141Sfs*59_** | CIALWCASS | 29571,44 | 5720,17 | 23197,03 | 36428,83 | 27824,15 | 30529,56 | 19750,27 | 32448,49 | 37225,73 | 41276,19 | 29696,81 | 13624,28 |
| **ARID1A2_F2141Sfs*59_** | IALWCASSV | 26226,04 | 903,19 | 20801,29 | 24330,45 | 31951,35 | 5790,16 | 8908,39 | 21555,57 | 13049,89 | 25996,35 | 2267,31 | 7923,27 |
| **ARID1A2_F2141Sfs*59_** | ALWCASSVT | 32397,27 | 547,91 | 19644,12 | 38296,94 | 38466,37 | 21250,81 | 21494,78 | 24265,25 | 27134,13 | 26450,03 | 29318,16 | 5720,36 |
| **ARID1A2_F2141Sfs*59_** | LWCASSVTE | 43094,33 | 36275,04 | 34489,89 | 22806,31 | 44719,34 | 42218,38 | 35670,23 | 35989,26 | 43274,24 | 43798,97 | 36086,36 | 31975,2 |
| **ARID1A2_F2141Sfs*59_** | WCASSVTER | 33140,37 | 34451,86 | 25089,15 | 42837,72 | 31694,13 | 34992,83 | 35887,77 | 31487,69 | 37612,79 | 39679,14 | 31944,07 | 33400,64 |
| **ARID1A2_F2141Sfs*59_** | CASSVTERT | 25488,07 | 27847,35 | 37732,2 | 40804,62 | 36170,01 | 32863,29 | 32856,19 | 34451,86 | 33250,64 | 38985,91 | 7557,6 | 30080,35 |
| **ARID1A2_F2141Sfs*59_** | ASSVTERTR | 32818,88 | 39626,39 | 11755,94 | 43949,47 | 39356,35 | 37878,65 | 41353,96 | 33547,7 | 43589,54 | 42444,64 | 20021,36 | 32463,23 |
| **ARID1A2_F2141Sfs*59_** | SSVTERTRC | 35084,21 | 38589,76 | 36986,44 | 42527,39 | 39605,82 | 29881,51 | 31765,2 | 36839,46 | 37540,4 | 41802,94 | 13400,45 | 30673,59 |
| **ARID1A2_F2141Sfs*59_** | SVTERTRCA | 34494,74 | 24988,9 | 30575,84 | 42613,51 | 23643,77 | 11991,42 | 3222,58 | 35370,05 | 31303,91 | 40518,22 | 32740,13 | 23274,96 |
| **ARID1A2_F2141Sfs*59_** | VTERTRCAG | 21274,27 | 39427,5 | 34208,19 | 42603,37 | 42630,11 | 21783,69 | 23073,13 | 35130,18 | 37752,62 | 42297,03 | 17921,19 | 24188,19 |
| **ARID1A2_F2141Sfs*59_** | TERTRCAGR | 40789,6 | 42893,39 | 28949,05 | 43491,55 | 34138,3 | 37362,11 | 24150,27 | 25371,68 | 39584,8 | 35083,06 | 43553,72 | 33999,34 |
| **ARID1A2_F2141Sfs*59_** | ERTRCAGRW | 39284,43 | 45675,36 | 42685,96 | 34803,29 | 35313,85 | 40500,66 | 32122,56 | 6372,57 | 32610,74 | 42310,29 | 18157,55 | 36231,11 |
| **ARID1A2_F2141Sfs*59_** | RTRCAGRWL | 24074,88 | 19569,87 | 8849,4 | 16592,79 | 23481,88 | 189,14 | 5663,02 | 7937,69 | 27271,57 | 17555,01 | 3949,18 | 2880,8 |
| **ARID1A2_F2141Sfs*59_** | TRCAGRWLW | 31360,18 | 35181,53 | 33434,62 | 6384,99 | 32907,76 | 34582,19 | 22341,05 | 593,3 | 14007,99 | 33332,06 | 2643,28 | 26214,41 |
| **ARID1A2_F2141Sfs*59_** | RCAGRWLWY | 9018,07 | 31891,93 | 4565,34 | 28064,23 | 21991,61 | 23673,72 | 24893,92 | 16036,1 | 39480,45 | 35520,39 | 2776,09 | 4475,88 |
| **ARID1A2_F2141Sfs*59_** | CAGRWLWYC | 29243,08 | 12050,86 | 32577,59 | 27670,46 | 42014,7 | 42375,82 | 23282,27 | 31372,74 | 37820,9 | 42953,3 | 9110,65 | 39116,89 |
| **ARID1A2_F2141Sfs*59_** | AGRWLWYCW | 33737,34 | 37348,38 | 30480,04 | 6972,24 | 39863,77 | 29667,91 | 18977,88 | 22948,39 | 39723,39 | 35331,82 | 674,78 | 22144,19 |
| **ARID1A2_F2141Sfs*59_** | GRWLWYCWP | 42088,87 | 30241,89 | 38548,44 | 36420,56 | 45472,18 | 44051,34 | 31630,41 | 776,31 | 29423,99 | 31852,28 | 40052,71 | 41359,78 |
| **ARID1A2_F2141Sfs*59_** | RWLWYCWPT | 33307,18 | 7608,89 | 19680,08 | 2574,35 | 40984,7 | 32628,39 | 11567,81 | 8793,95 | 26807,88 | 31027,41 | 29597,37 | 29159,36 |
| **ARID1A2_F2141Sfs*59_** | WLWYCWPTW | 21634,77 | 3748,02 | 18367,99 | 2087,63 | 26795,99 | 25484,76 | 12865,39 | 10551,28 | 25351,92 | 22729,94 | 357,09 | 6598,78 |
| **ARID1A2_F2141Sfs*59_** | LWYCWPTWL | 28091,27 | 4036,23 | 17462,94 | 395,08 | 29887,96 | 22849,53 | 5611,48 | 8517,78 | 16755,34 | 26785,85 | 19771,42 | 13422,65 |
| **ARID1A2_F2141Sfs*59_** | WYCWPTWLR | 21189,28 | 20408,47 | 9842,7 | 5383,35 | 24160,98 | 40314 | 32639,7 | 12037,7 | 32079,85 | 38579,73 | 27726,5 | 35875,74 |
| **ARID1A2_F2141Sfs*59_** | YCWPTWLRG | 30534,83 | 22640,11 | 34993,21 | 30812,29 | 43967,53 | 35302,39 | 29885,71 | 27988,41 | 35382,3 | 38018,67 | 9180,21 | 32122,91 |
| **ARID1A2_F2141Sfs*59_** | CWPTWLRGT | 40896,09 | 31985,24 | 36918,09 | 15030,73 | 35144,22 | 40365,93 | 32351,38 | 34734,83 | 43646,65 | 46029,59 | 44316,2 | 38970,73 |
| **ARID1A2_F2141Sfs*59_** | WPTWLRGTA | 30948,95 | 31140,75 | 33196,01 | 38810,41 | 28260,77 | 40,77 | 2436,27 | 21143,94 | 4030,38 | 30553,35 | 30280,2 | 28520,63 |
| **ARID1A2_F2141Sfs*59_** | PTWLRGTAW | 24353,63 | 37381,53 | 33300,32 | 16476,86 | 27775,73 | 26842,41 | 19993,64 | 27886,54 | 41060,6 | 40910,26 | 489,72 | 18218,55 |
| **ARID1A2_F2141Sfs*59_** | TWLRGTAWQ | 42682,27 | 40719,5 | 30579,8 | 25001,07 | 41250,75 | 40794,47 | 34472,75 | 33017,96 | 44330,09 | 45357,7 | 39655,96 | 37509,54 |
| **ARID1A2_F2141Sfs*59_** | WLRGTAWQL | 25459,96 | 65,54 | 18830 | 9975,21 | 16682,62 | 2052,27 | 226,76 | 7277,85 | 3186,59 | 13700,26 | 16825,46 | 1269,7 |
| **ARID1A2_F2141Sfs*59_** | LRGTAWQLV | 31056,62 | 29153,38 | 37188,69 | 20624,91 | 40030,6 | 31115,48 | 20940,4 | 618,05 | 11981,68 | 32546,94 | 29638,72 | 31951,35 |
| **ARID1A2_F2141Sfs*59_** | RGTAWQLVP | 37615,21 | 32711,11 | 33105,25 | 33606,9 | 45886,88 | 33309,34 | 36104,31 | 28037,82 | 34782,57 | 35430,96 | 8076,56 | 29483,9 |
| **ARID1A2_F2141Sfs*59_** | GTAWQLVPL | 17175,3 | 400,55 | 15228,65 | 19565,86 | 7345,34 | 4877,51 | 14310,24 | 18009,82 | 5494,45 | 11971,84 | 4602,44 | 3197,61 |
| **ARID1A2_F2141Sfs*59_** | TAWQLVPLQ | 36611 | 31293,07 | 28224,39 | 41053,93 | 34495,88 | 35036,41 | 32500,85 | 29577,85 | 38005,92 | 37379,1 | 10622,41 | 30704,12 |
| **ARID1A2_F2141Sfs*59_** | AWQLVPLQC | 42424,91 | 30845,99 | 38742,04 | 14964,53 | 46690,68 | 41531,54 | 31309,66 | 35099,4 | 40821,84 | 41445,35 | 38523,84 | 38773,06 |
| **ARID1A2_F2141Sfs*59_** | WQLVPLQCR | 37004,07 | 25785,42 | 16486,84 | 34221,52 | 35775,74 | 37134 | 26354,34 | 4498,12 | 28898,67 | 29554,82 | 35338,32 | 22091,06 |
| **ARID1A2_F2141Sfs*59_** | QLVPLQCRR | 34281,93 | 24341,51 | 4919,48 | 39024,32 | 25970,78 | 38704,33 | 37440,62 | 22766,62 | 40530,48 | 41449,39 | 33151,85 | 29540,43 |
| **ARID1A2_F2141Sfs*59_** | LVPLQCRRA | 36394,57 | 22373,22 | 36014,2 | 38167,02 | 33385,11 | 31856,06 | 19224,02 | 36913,68 | 40602,03 | 43716,12 | 38789,84 | 28190,83 |
| **ARID1A2_F2141Sfs*59_** | VPLQCRRAV | 38163,72 | 27665,36 | 32700,84 | 37369,79 | 37915,14 | 9,78 | 1049,08 | 24823,99 | 8803,18 | 33282,68 | 34020,68 | 30561,94 |
| **ARID1A2_F2141Sfs*59_** | PLQCRRAVS | 40919,12 | 37664,9 | 37466,56 | 43024,93 | 45362,11 | 34229,66 | 13931,36 | 38695,95 | 43542,88 | 45600,78 | 43864,89 | 35470,08 |
| **ARID1A2_F2141Sfs*59_** | LQCRRAVSA | 34942,52 | 14230,72 | 27974,19 | 35392,64 | 38783,56 | 24942,98 | 1461,47 | 12468,79 | 8844,81 | 21659,13 | 30218,34 | 4066,56 |
| **ARID1A2_F2141Sfs*59_** | QCRRAVSAT | 43422,91 | 41455,21 | 37904,88 | 45510,09 | 42814,1 | 10840,23 | 15156,97 | 39358,05 | 40281,28 | 42290,62 | 36538,16 | 28625,13 |
| **ARID1A2_F2141Sfs*59_** | CRRAVSATS | 41941,11 | 41233,32 | 33674,24 | 40105,18 | 40428,43 | 22075,53 | 15920,27 | 2614,04 | 24621,76 | 39787,07 | 37925,8 | 28101,29 |
| **ARID1A2_F2141Sfs*59_** | RRAVSATSW | 38550,93 | 37618,48 | 31275,82 | 12209,4 | 36269,54 | 19727,62 | 20699,59 | 364,47 | 14965,66 | 26938,14 | 1949,2 | 14434,33 |
| **ARID1A2_F2141Sfs*59_** | RAVSATSWA | 28712,92 | 11547,17 | 17733,89 | 34788,23 | 26158,59 | 3697,79 | 20041,73 | 17305,13 | 20727,6 | 24582,63 | 3256,19 | 7673,46 |
| **ARID1A2_F2141Sfs*59_** | AVSATSWAS | 26410,57 | 7894,61 | 17160,45 | 36730,81 | 28499,96 | 18633,82 | 28440,2 | 29586,49 | 24010,62 | 29829,18 | 11874,83 | 10932,57 |
|  |  |  |  |  |  |  |  |  |  |  |  |  |  |
| **PROTEIN** | **PEPTIDE** | **A*01:01** | **A*02:01** | **A*03:01** | **A*24:02** | **A*26:02** | **B*07:02** | **B*08:02** | **B*27:05** | **B*39:01** | **B*40:01** | **B*58:01** | **B*15:01** |
| **NPM1_317wt_** | DQEAIQDLW | 20923,19 | 39571,12 | 42211,54 | 18440,46 | 31916,09 | 40529,17 | 37667,36 | 32427,79 | 26046,48 | 24267,09 | 5353,61 | 23747,86 |
| **NPM1_317wt_** | QEAIQDLWQ | 41188,3 | 45690,18 | 43845,46 | 47278,84 | 44431,89 | 44388,65 | 42867,87 | 39147,81 | 43411,18 | 29488,7 | 41495,15 | 39831,44 |
| **NPM1_317wt_** | EAIQDLWQW | 28894,92 | 32651,7 | 40994,45 | 11170,15 | 4576,71 | 36087,92 | 28794,12 | 32325,49 | 29233,6 | 35160,98 | 32,59 | 22169,6 |
| **NPM1_317wt_** | AIQDLWQWR | 31990,42 | 25377,46 | 5663,27 | 33865,34 | 26724,76 | 35905,25 | 35106,23 | 22101,35 | 42943,55 | 40771,06 | 34191,91 | 33260 |
| **NPM1_317wt_** | IQDLWQWRK | 8737,04 | 15946,13 | 1926,5 | 25887,74 | 41139,3 | 41854,97 | 37593,65 | 8644,79 | 30492,25 | 29382,96 | 23070,38 | 30111,93 |
| **NPM1_317wt_** | QDLWQWRKS | 39701,05 | 43524,52 | 40394,76 | 44119,09 | 43958,51 | 42970,03 | 37345,14 | 35386,53 | 42992,34 | 39912,54 | 41621,06 | 43907,17 |
| **NPM1_317wt_** | DLWQWRKSL | 34297,52 | 7267,39 | 33836,77 | 22838,91 | 14441,99 | 7001,34 | 318,57 | 18556,55 | 8526,82 | 29928,1 | 38333,41 | 17531,47 |
|  |  |  |  |  |  |  |  |  |  |  |  |  |  |
| **NPM1_W317Cfs*12_** | DQEAIQDLC | 35292,47 | 38304,39 | 45302,3 | 42028,33 | 44126,72 | 43487,79 | 39142,74 | 41248,51 | 22555,27 | 28365,23 | 39919 | 39722,54 |
| **NPM1_W317Cfs*12_** | QEAIQDLCL | 30848,66 | 34422,43 | 41270,84 | 35759,09 | 37113,12 | 29965,03 | 22056,44 | 32844,81 | 10747,27 | 179,74 | 33397,75 | 18934,19 |
| **NPM1_W317Cfs*12_** | EAIQDLCLA | 25681,84 | 13479,12 | 37553,82 | 40840,82 | 3339,25 | 27403,49 | 21446,92 | 33557,5 | 17650,62 | 33146,12 | 17420,48 | 22600,21 |
| **NPM1_W317Cfs*12_** | AIQDLCLAV | 21271,74 | 116,61 | 21269,9 | 30420,73 | 26199,96 | 14985,43 | 13619,56 | 26209,32 | 19450,82 | 26490,98 | 29023,08 | 7520,57 |
| **NPM1_W317Cfs*12_** | IQDLCLAVE | 31903,66 | 22490,21 | 34255,23 | 39328,67 | 43146,58 | 38449,73 | 36034,09 | 31375,46 | 27314,99 | 24612,17 | 35945,68 | 22107,8 |
| **NPM1_W317Cfs*12_** | QDLCLAVEE | 42058,34 | 41549,05 | 42667,05 | 44574,42 | 45669,91 | 43798,97 | 40995,78 | 40599,83 | 42935,18 | 36379,2 | 41703,09 | 42127,59 |
| **NPM1_W317Cfs*12_** | DLCLAVEEV | 28752,4 | 5975,18 | 38444,3 | 36368,97 | 26708,86 | 38443,05 | 22436,49 | 39831,85 | 25128,8 | 37519,71 | 38229,03 | 28771,69 |
| **NPM1_W317Cfs*12_** | LCLAVEEVS | 42287,43 | 33708,5 | 43330,93 | 45984,79 | 47803 | 42708,62 | 41049,93 | 43835,03 | 41364,26 | 42129,41 | 21585,2 | 38776,41 |
| **NPM1_W317Cfs*12_** | CLAVEEVSL | 25888,57 | 380,1 | 28641,86 | 26518,52 | 35193,33 | 19566,28 | 7162,71 | 30414,16 | 10460,12 | 24793,65 | 20492,12 | 4234,59 |
| **NPM1_W317Cfs*12_** | LAVEEVSLR | 31596,54 | 30279,87 | 19713,11 | 41897,13 | 14186,6 | 37183,46 | 39002,38 | 29243,41 | 39576,69 | 39935,86 | 20996,44 | 27741,5 |
| **NPM1_W317Cfs*12_** | AVEEVSLRK | 14406,71 | 33427,4 | 396,2 | 42525,55 | 35193,33 | 36763,42 | 42072,02 | 31792,36 | 43770,56 | 39899,57 | 30710,44 | 31145,13 |
|  |  |  |  |  |  |  |  |  |  |  |  |  |  |
| **PROTEIN** | **PEPTIDE** | **A*01:01** | **A*02:01** | **A*03:01** | **A*24:02** | **A*26:02** | **B*07:02** | **B*08:02** | **B*27:05** | **B*39:01** | **B*40:01** | **B*58:01** | **B*15:01** |
| **BLM_515wt_** | ETPRLGKKN | 40655,21 | 47094,55 | 45516,48 | 46064,96 | 32395,51 | 44698,55 | 41493,36 | 43501,93 | 46976,98 | 48181,02 | 41512,66 | 44117,16 |
| **BLM_515wt_** | TPRLGKKNE | 47333,61 | 47556,91 | 42709,53 | 47625,41 | 46093,87 | 6596,28 | 19585,98 | 43175,56 | 45239,09 | 46831,33 | 46075,41 | 44678,23 |
| **BLM_515wt_** | PRLGKKNES | 47874,94 | 47631,59 | 46770,06 | 47562,06 | 48402,58 | 45287,08 | 40534,87 | 32036,15 | 44698,55 | 47914,32 | 47453,61 | 47703,8 |
| **BLM_515wt_** | RLGKKNESS | 41593,59 | 23839,51 | 29904,47 | 40512,5 | 45374,4 | 29299,15 | 12311,67 | 29725,42 | 41366,95 | 42463,94 | 36434,75 | 22160,96 |
| **BLM_515wt_** | LGKKNESSY | 21374,18 | 43256,91 | 25076,93 | 41395,16 | 28229,29 | 30288,05 | 17282,86 | 34288,61 | 40332,75 | 41364,26 | 12379 | 481,46 |
| **BLM_515wt_** | GKKNESSYF | 41939,3 | 44355,05 | 40198,57 | 37253,93 | 32849,43 | 35824,55 | 22518,68 | 16486,49 | 33703,05 | 29339,44 | 36543,32 | 3183,25 |
| **BLM_515wt_** | KKNESSYFP | 44523,8 | 39264,9 | 40908,49 | 41297,2 | 46400,11 | 41687,31 | 39115,64 | 28946,55 | 30009,49 | 26648,25 | 30698,14 | 34611,4 |
| **BLM_515wt_** | KNESSYFPG | 35758,33 | 37923,75 | 35697,64 | 34038,36 | 45144,25 | 39461,23 | 33055,87 | 31159,61 | 34547,42 | 38346,27 | 23738,1 | 36371,73 |
| **BLM_515wt_** | NESSYFPGN | 40349,77 | 41961,08 | 42937,04 | 42348,31 | 40843,48 | 43017,95 | 36150,45 | 33709,96 | 36061,38 | 22256,12 | 35694,92 | 35206,65 |
|  |  |  |  |  |  |  |  |  |  |  |  |  |  |
| **BLM_N515Mfs*16_** | ETPRLGKKM | 32030,61 | 41461,95 | 42523,25 | 33885,48 | 2306,2 | 29708,07 | 26268,93 | 36972,44 | 34920,22 | 41054,83 | 33200,68 | 29339,75 |
| **BLM_N515Mfs*16_** | TPRLGKKMK | 44720,32 | 46464,41 | 28275,44 | 46439,27 | 41959,73 | 8278,46 | 23054,17 | 37405,79 | 44709,68 | 45676,34 | 44601,91 | 42549,03 |
| **BLM_N515Mfs*16_** | PRLGKKMKA | 46082,9 | 45615,61 | 44094,74 | 45712,93 | 47233,34 | 43073,35 | 31993,2 | 15628,09 | 39188,91 | 46649,27 | 45919,14 | 46582,71 |
| **BLM_N515Mfs*16_** | RLGKKMKAL | 37939,36 | 4913,95 | 17446,7 | 23404,77 | 35480,83 | 2060,05 | 125,78 | 15815,89 | 22780,91 | 27141,76 | 30931,87 | 3956,11 |
| **BLM_N515Mfs*16_** | LGKKMKALI | 37144,85 | 34294,18 | 37150,09 | 23447,1 | 42840,04 | 22619,3 | 2531,89 | 27313,51 | 35620,85 | 38198,44 | 15519,07 | 20976,45 |
| **BLM_N515Mfs*16_** | GKKMKALIS | 46401,1 | 46216,71 | 42808,53 | 46163,25 | 46777,66 | 41524,79 | 15839,35 | 23836,16 | 39733,28 | 41681,9 | 44391,54 | 36224,45 |
| **BLM_N515Mfs*16_** | KKMKALISQ | 45267,98 | 37957,42 | 29538,83 | 44309,96 | 43401,3 | 32878,23 | 30051,4 | 8830,65 | 32902,44 | 34234,12 | 30216,04 | 24398,47 |
| **BLM_N515Mfs*16_** | KMKALISQE | 42639,35 | 22071,23 | 6283,42 | 39243,66 | 40506,8 | 28147,56 | 13973,48 | 19544,27 | 41981,52 | 39281,04 | 28220,43 | 6715,1 |
| **BLM_N515Mfs*16_** | MKALISQEM | 28856,49 | 23544,46 | 33726,03 | 28694,9 | 23141,63 | 11925,17 | 5438,91 | 7815,23 | 85,03 | 6667,54 | 16450,32 | 1612,63 |
| **BLM_N515Mfs*16_** | KALISQEMF | 32033,73 | 28451,91 | 30910,47 | 6552,4 | 37016,07 | 23615,38 | 27026,01 | 23787,2 | 33954,13 | 26392,01 | 23,69 | 3627,61 |
| **BLM_N515Mfs*16_** | ALISQEMFS | 30009,49 | 2032,09 | 14316,28 | 40656,97 | 37697,51 | 37101,49 | 34377,75 | 32005,67 | 39571,53 | 37828,68 | 27263,9 | 18442,46 |
| **BLM_N515Mfs*16_** | LISQEMFSQ | 34824,01 | 25924,73 | 25750,02 | 44090,45 | 33424,15 | 36332,8 | 37454,41 | 37702,81 | 41184,75 | 43338,42 | 21426,27 | 20253,84 |
| **BLM_N515Mfs*16_** | ISQEMFSQA | 15224,69 | 11105,08 | 26927,35 | 28874,3 | 32154,56 | 22464,67 | 14388,49 | 27638,14 | 27418,02 | 32636,16 | 7270,54 | 11406,73 |
| **BLM_N515Mfs*16_** | SQEMFSQAL | 19555,9 | 5648,15 | 32409,2 | 18990 | 30963,02 | 10069,77 | 4312,17 | 13846,75 | 35,43 | 502,2 | 32799,71 | 665,11 |
| **BLM_N515Mfs*16_** | QEMFSQALL | 22677,13 | 13243,2 | 29433,86 | 17547,22 | 22241,2 | 16224,59 | 14413,57 | 14435,26 | 2075,4 | 8,02 | 18951,82 | 5364,22 |
|  |  |  |  |  |  |  |  |  |  |  |  |  |  |
| **PROTEIN** | **PEPTIDE** | **A*01:01** | **A*02:01** | **A*03:01** | **A*24:02** | **A*26:02** | **B*07:02** | **B*08:02** | **B*27:05** | **B*39:01** | **B*40:01** | **B*58:01** | **B*15:01** |
| **ZMYM2_1044wt_** | QPRPRSKKK | 44379,06 | 47048,2 | 28329,34 | 46389,04 | 42439,14 | 9419,89 | 18704,31 | 38067,64 | 45377,82 | 46349,42 | 44919,62 | 42790,95 |
| **ZMYM2_1044wt_** | PRPRSKKKG | 48625,66 | 49141,85 | 48544,16 | 47990,62 | 48832,34 | 47679,54 | 45960,41 | 41193,64 | 48054,51 | 48921,71 | 48373,78 | 48426,65 |
| **ZMYM2_1044wt_** | RPRSKKKGA | 44550,31 | 45697,11 | 38868,82 | 46043,51 | 45457,43 | 120,41 | 1848,77 | 32708,98 | 39017,98 | 44194,55 | 44271,15 | 41155,78 |
| **ZMYM2_1044wt_** | PRSKKKGAK | 46661,39 | 47658,91 | 38195,12 | 46921,12 | 47014,11 | 43208,73 | 39017,15 | 13316,04 | 46220,23 | 47461,32 | 46746,27 | 45920,15 |
| **ZMYM2_1044wt_** | RSKKKGAKR | 35490,45 | 41431,88 | 4494,37 | 40367,67 | 37227,75 | 28130,5 | 20621,12 | 15324,67 | 44264,44 | 43396,15 | 31666,37 | 25482 |
| **ZMYM2_1044wt_** | SKKKGAKRK | 46949,03 | 47285 | 30398,04 | 47497,27 | 45710,46 | 43003,52 | 34208,56 | 21917,74 | 46257,75 | 44659,39 | 45656,07 | 38355,39 |
| **ZMYM2_1044wt_** | KKKGAKRKA | 47899,84 | 47416,16 | 43948,99 | 47203,2 | 47033,94 | 35561,54 | 29990,66 | 25850,5 | 44245,75 | 44984,32 | 45418,11 | 40572,16 |
| **ZMYM2_1044wt_** | KKGAKRKAV | 45546,05 | 44629,91 | 41804,74 | 45178,94 | 45873,95 | 20231,5 | 5603,35 | 16264,49 | 32695,52 | 40591,92 | 43949,47 | 37961,12 |
| **ZMYM2_1044wt_** | KGAKRKAVS | 43966,59 | 43590,5 | 35758,72 | 44000,38 | 46511,68 | 25553,51 | 15420,82 | 36192,71 | 42873,43 | 44894,86 | 32223,53 | 34220,41 |
|  |  |  |  |  |  |  |  |  |  |  |  |  |  |
| **ZMYM2_K1044Rfs*33_** | QPRPRSKKR | 44461,22 | 47317,25 | 35297,82 | 46510,7 | 40726,09 | 14670,19 | 19429,37 | 39149,07 | 44839,01 | 46684,12 | 45632,38 | 44106,66 |
| **ZMYM2_K1044Rfs*33_** | PRPRSKKRE | 48601,45 | 49052,62 | 48258,24 | 48088,83 | 48522,66 | 47734,79 | 45334,65 | 40856,73 | 48123,2 | 48983,68 | 48699,88 | 48563,61 |
| **ZMYM2_K1044Rfs*33_** | RPRSKKREP | 45728,77 | 46479,99 | 41493,81 | 46465,9 | 46839,94 | 629,65 | 9045,33 | 37851,6 | 43095,75 | 44507,92 | 44103,33 | 42360,22 |
| **ZMYM2_K1044Rfs*33_** | PRSKKREPR | 45915,18 | 46909,44 | 41496,06 | 45248,39 | 46631,61 | 43612,18 | 36628,43 | 11894,12 | 43713,78 | 47213,95 | 46202,23 | 46208,73 |
| **ZMYM2_K1044Rfs*33_** | RSKKREPRE | 43617,86 | 44932,25 | 28119,85 | 45165,25 | 46034,55 | 34931,19 | 19693,07 | 31693,44 | 46866,8 | 45455,97 | 28299,32 | 32958,71 |
| **ZMYM2_K1044Rfs*33_** | SKKREPRER | 46697,76 | 46793,36 | 38607,28 | 47195,04 | 45132,03 | 42426,28 | 33853,99 | 27494,68 | 43991,8 | 45488,43 | 46268,75 | 41810,62 |
| **ZMYM2_K1044Rfs*33_** | KKREPRERL | 44717,41 | 40997,12 | 36682,35 | 40449,45 | 43406 | 8823,3 | 13615,58 | 10437,96 | 26969,63 | 25584,23 | 36731,21 | 23560,26 |
| **ZMYM2_K1044Rfs*33_** | KREPRERLY | 31226,44 | 43494,38 | 30545,75 | 40899,2 | 38359,55 | 41001,54 | 36191,55 | 2544,64 | 36558,34 | 38979,17 | 31481,22 | 26459,19 |
| **ZMYM2_K1044Rfs*33_** | REPRERLYQ | 41667,02 | 44461,22 | 38731,97 | 43202,66 | 43880,08 | 40723,02 | 37763,23 | 29931,65 | 42938,89 | 28528,67 | 41407,25 | 36581,69 |
| **ZMYM2_K1044Rfs*33_** | EPRERLYQD | 46378 | 46334,39 | 44726,61 | 45566,77 | 42564,21 | 21103,71 | 7787,54 | 40930,63 | 42503,48 | 46500,62 | 43348,73 | 43663,18 |
| **ZMYM2_K1044Rfs*33_** | PRERLYQDT | 45921,13 | 48324,07 | 47808,18 | 46468,42 | 48529,48 | 47012,59 | 42681,36 | 36723,25 | 45464,82 | 47886,34 | 47448,49 | 48091,43 |
| **ZMYM2_K1044Rfs*33_** | RERLYQDTS | 41537,83 | 42507,62 | 39940,61 | 41335,19 | 43890,07 | 32377,3 | 24529,75 | 25453,34 | 35977,2 | 7620,34 | 37816 | 28414,06 |
| **ZMYM2_K1044Rfs*33_** | ERLYQDTSL | 40679,87 | 36682,35 | 41868,12 | 36740,36 | 34397,11 | 27020,75 | 6794,4 | 2538,94 | 159,82 | 26936,97 | 38346,68 | 31917,82 |
| **ZMYM2_K1044Rfs*33_** | RLYQDTSLM | 19606,33 | 230,18 | 1441,58 | 9310,15 | 11138,17 | 3041,96 | 8865,79 | 3857,83 | 7592,6 | 12327 | 1962,68 | 32,62 |
| **ZMYM2_K1044Rfs*33_** | LYQDTSLMM | 19472,09 | 18674,99 | 27828,68 | 399,67 | 21814,35 | 20182,53 | 16547,43 | 20238,29 | 9938,69 | 25838,2 | 18706,34 | 7181,8 |
| **ZMYM2_K1044Rfs*33_** | YQDTSLMMI | 2378,56 | 50,68 | 27599,28 | 6661,62 | 30416,46 | 21304,22 | 12623,68 | 8557,78 | 194,02 | 1272,41 | 13439,8 | 4033,96 |
| **ZMYM2_K1044Rfs*33_** | QDTSLMMIV | 23166,93 | 20474,39 | 34216,33 | 30770,99 | 28578,41 | 30776,98 | 27449,78 | 29440,87 | 23540,65 | 13992,39 | 23295,13 | 29748,28 |
| **ZMYM2_K1044Rfs*33_** | DTSLMMIVL | 14802,36 | 14968,42 | 34375,16 | 28379,04 | 2959,47 | 24107,71 | 10807,55 | 34890,39 | 9327,49 | 27696,51 | 14948,34 | 21213,83 |
| **ZMYM2_K1044Rfs*33_** | TSLMMIVLT | 22335,25 | 10760,65 | 26789,02 | 34787,49 | 38128,23 | 38753,36 | 21623,54 | 30245,48 | 32027,14 | 36580,89 | 5233,11 | 26097,81 |
| **ZMYM2_K1044Rfs*33_** | SLMMIVLTI | 22112,58 | 20 | 6134,36 | 6061,39 | 25902,87 | 19120,09 | 838,48 | 13606,6 | 8797,27 | 20576,55 | 6866,75 | 1646,95 |
| **ZMYM2_K1044Rfs*33_** | LMMIVLTIQ | 35818,73 | 10488,33 | 9872,14 | 40907,62 | 38179,43 | 35713,48 | 16261,14 | 28403,3 | 37276,52 | 39237,29 | 22871,05 | 5737,71 |
| **ZMYM2_K1044Rfs*33_** | MMIVLTIQN | 32041,35 | 8489,08 | 16347,59 | 32071,87 | 33808,59 | 37134,81 | 16487,39 | 25578,96 | 30245,81 | 35877,29 | 8677,41 | 4814,8 |
| **ZMYM2_K1044Rfs*33_** | MIVLTIQNA | 32271,32 | 1347,92 | 22726,26 | 37188,69 | 11385,28 | 21979,49 | 12015,05 | 30655,67 | 23691,92 | 34681,49 | 18938,29 | 6384,85 |
| **ZMYM2_K1044Rfs*33_** | IVLTIQNAA | 32832,72 | 4845,37 | 24248,98 | 39536,02 | 35684,12 | 17915,36 | 15612,88 | 31108,07 | 22350,48 | 32281,1 | 18331,85 | 14694,5 |
| **ZMYM2_K1044Rfs*33_** | VLTIQNAAF | 23646,08 | 9697,57 | 23909,26 | 8209,33 | 19375,62 | 13115,29 | 9134,93 | 26238,81 | 22449,11 | 25633,26 | 10759,14 | 134,44 |
| **ZMYM2_K1044Rfs*33_** | LTIQNAAFL | 14011,48 | 1698,62 | 23031,22 | 10817,74 | 1864,32 | 13664,29 | 10903,99 | 21604,83 | 10533,25 | 17635,16 | 570,74 | 1726,15 |
| **ZMYM2_K1044Rfs*33_** | TIQNAAFLS | 23148,14 | 14130,22 | 21127,92 | 31222,74 | 34409,76 | 33101,67 | 33321,59 | 32018,14 | 35257,34 | 38883,96 | 22431,4 | 24581,03 |
| **ZMYM2_K1044Rfs*33_** | IQNAAFLSN | 33643,29 | 23718,33 | 22833,71 | 28346,21 | 38887,75 | 35779,23 | 29399,81 | 18029,9 | 24842,52 | 26632,96 | 11960,83 | 1385,66 |
| **ZMYM2_K1044Rfs*33_** | QNAAFLSNI | 25257,46 | 12975,27 | 26775,41 | 16274 | 16359,79 | 26039,43 | 17451,23 | 23957,41 | 14370,75 | 22968,76 | 9507,75 | 16915,08 |
| **ZMYM2_K1044Rfs*33_** | NAAFLSNIR | 26663,24 | 33969,55 | 17284,54 | 37125,98 | 16870,67 | 31248,74 | 30353,01 | 26568,49 | 30281,18 | 37410,25 | 24242,69 | 29509,75 |
| **ZMYM2_K1044Rfs*33_** | AAFLSNIRM | 26725,62 | 13440,24 | 22319,54 | 32610,74 | 22474,64 | 12884,76 | 20572,76 | 23431,88 | 13906,51 | 16088,93 | 1964,55 | 3390,81 |
| **ZMYM2_K1044Rfs*33_** | AFLSNIRMA | 40236 | 14690,36 | 28209,75 | 22673,21 | 37296,29 | 32602,27 | 18590,32 | 30777,63 | 34367,35 | 38860 | 35669,05 | 29396,62 |

Overlapping peptides have been designed to have the wt and mutated aminoacid residue in all nine positions. In addition, neoepitopes have been designed from the newly generated sequence derived from the alternative reading frame. Values of binding affinity (nM) to each haplotypes are reported. Green, neoepitopes with affinity <100nM; Red, neoepitopes with affinity between 100-200 nM; Gold, neoepitopes with affinity between 200-400 nM.
