# Supplementary figures and images for: Lack of shared neoantigens in prevalent mutations in cancer
Source: J Transl Med. 2024 Apr 10;22:344. doi: 10.1186/s12967-024-05110-0 (PMC11005154; doi:10.1186/s12967-024-05110-0)

## Slide 1
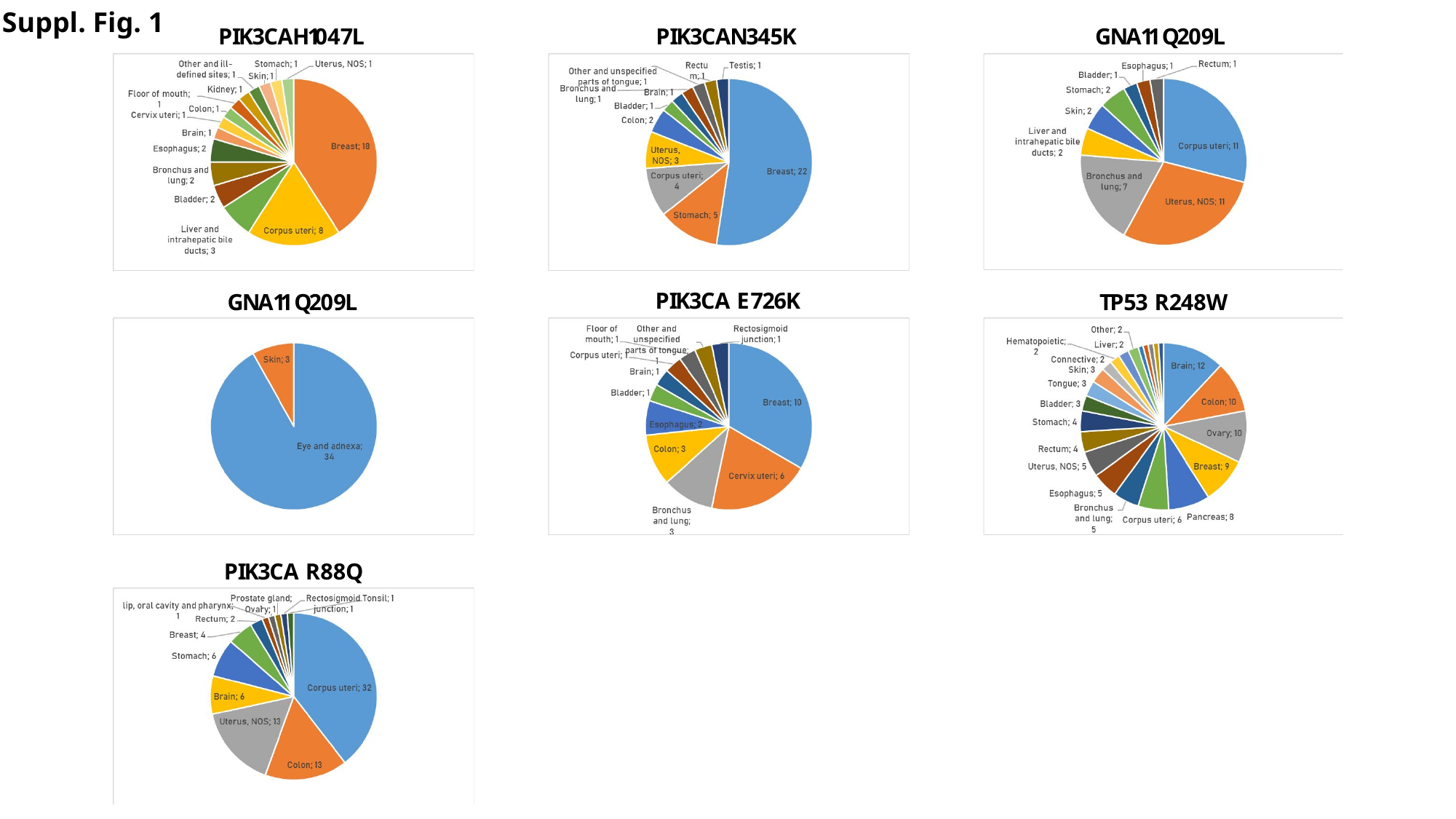

Suppl. Fig. 1

## Slide 2
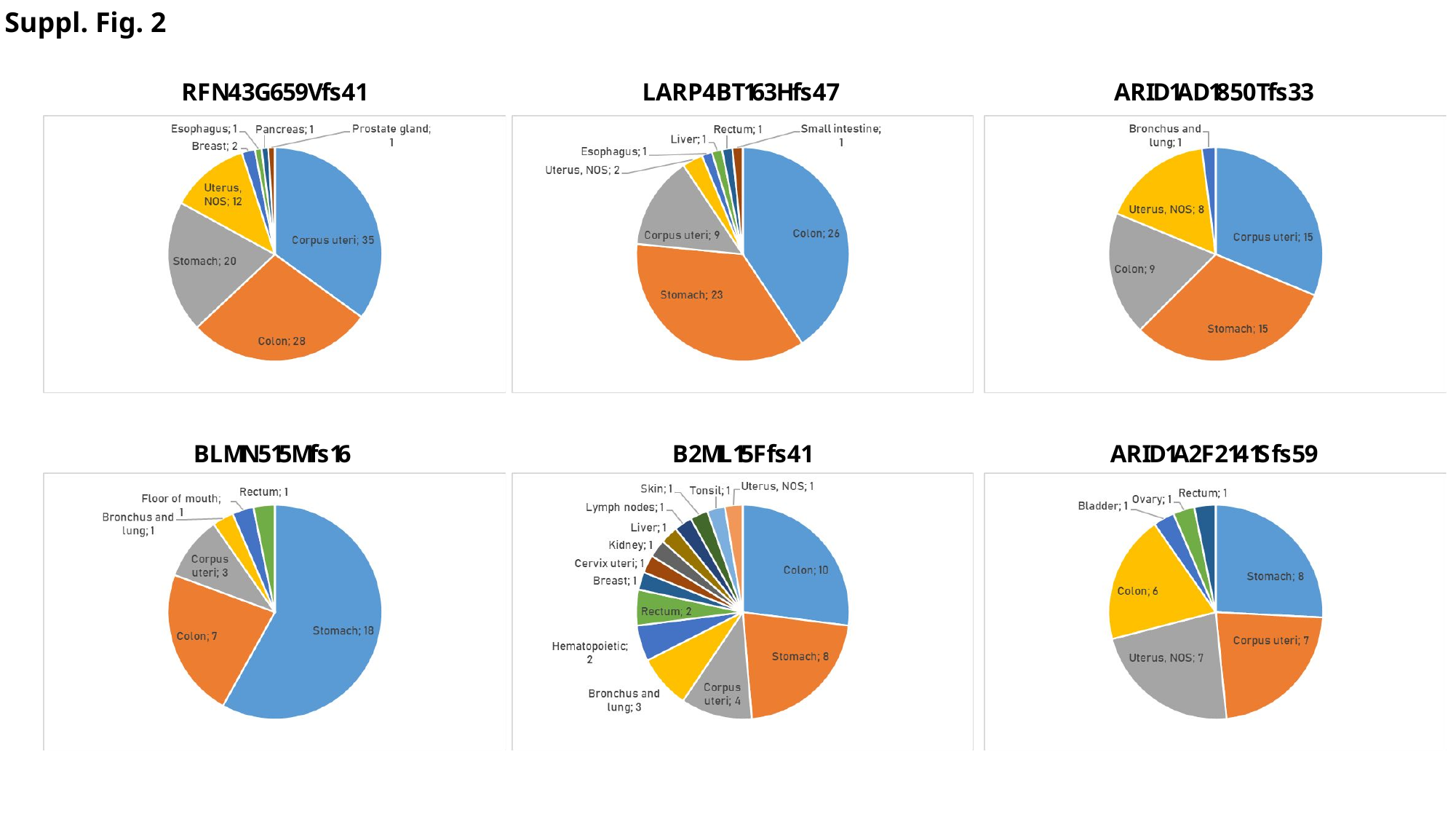

Suppl. Fig. 2

Supplement: Supplementary file 2 — Additional file 2: Fig. S1. Percentage of mutated samples, for each tumor type, presenting the indicated missense mutation giving rise to neoantigens. Fig. S2. Percentage of mutated samples, for each tumor type, presenting the indicated frameshift mutation giving rise to neoantigens. [file 12967_2024_5110_MOESM2_ESM.pptx]
